# Straight Forward and Versatile Differentiation of the l-*glycero* and d-*glycero*-d-*manno* Heptose Scaffold

**DOI:** 10.3389/fchem.2020.00625

**Published:** 2020-07-31

**Authors:** Christoph Suster, Ian R. Baxendale, Marko D. Mihovilovic, Christian Stanetty

**Affiliations:** ^1^Faculty of Technical Chemistry, Institute of Applied Synthetic Chemistry, TU Wien, Vienna, Austria; ^2^Department of Chemistry, University of Durham, Durham, United Kingdom

**Keywords:** heptose, higher carbon sugars, orthoester derivatives, lipopolysaccharides (LPS), carbohydrate chemistry, synthetic methodology

## Abstract

Bacterial lipopolysaccharides (LPS) are important bio-medical structures, playing a major role in the interaction with human immune systems. Their core regions, containing multiple units of l-*glycero*-d-*manno* heptoses (l,d-heptose), are highly conserved structurally (with *O*3 and *O*7 glycosidic bonds), making them an epitope of high interest for the potential development of new antibiotics and vaccines. Research in this field has always been restricted by the limited availability of the parent l,d-heptose as well as its biochemical epimeric precursor d-*glycero*-d-*manno* heptose (d,d-heptose). This problem of availability has recently been solved by us, through a rapid and efficient practical synthesis of l,d-*manno*-heptose peracetate demonstrated at scale. Herein we report an optimized, technically simple and versatile synthetic strategy for the differentiation of both the l-*glycero* and d-*glycero*-d-*manno* heptose scaffolds. Our approach is based on an orthoester methodology for the differentiation of all three positions of the sugar core using a *O*6, *O*7-tetraisopropyl disiloxyl (TIPDS) protecting group for the exocyclic positions. Furthermore, the regioselective opening toward 7-OH acceptors (6*O*-FTIPDS ethers) differentiates the exocyclic diol which has been demonstrated with a broader set of substrates and for both *manno*-heptoses for the first time.

## Introduction

**Figure 1:**
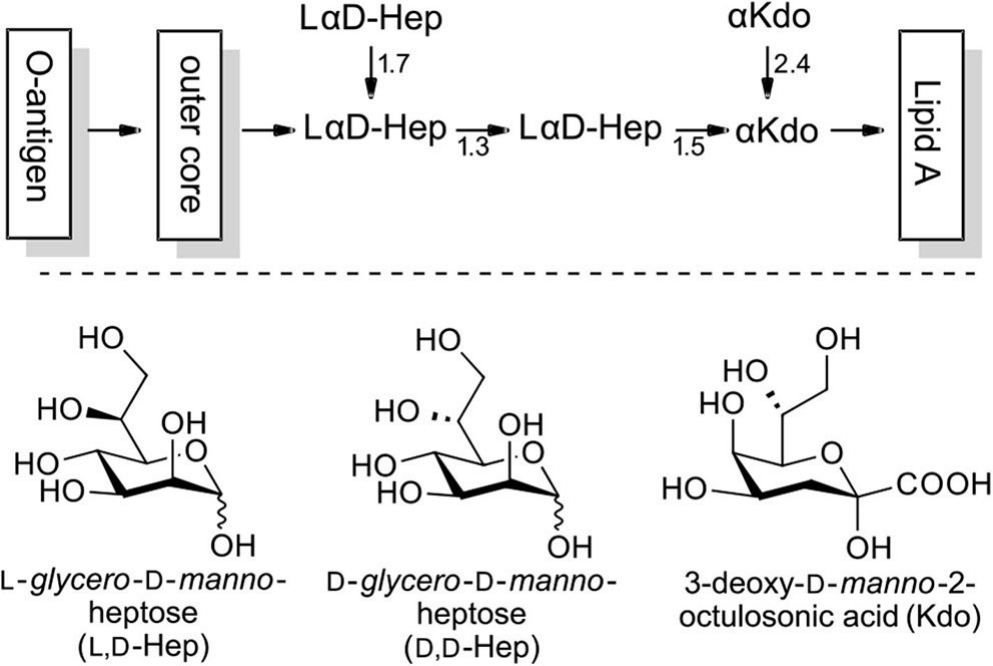
Exemplary structure of LPS with the l,d-heptose containing inner core region (phosphorylation not shown).

Microorganisms are able to generate a variety of sugars which, to our current knowledge are absent in vertebrate organisms. Among these, l-*glycero*-d-*manno*-heptose (l,d-heptose) has been identified as a major constituent of the lipopolysaccharide (LPS) of Gram-negative bacteria, an important mediator for numerous interactions with the native and adaptive immune system of the host (Holst, 2007; Kosma, 2008). The inner core region is based on l,d-heptoses and 3-deoxy-d-*manno*-2-octulosonic acid (Kdo) and is exhibited in a highly conserved manner in many enterobacterial strains (exemplified in Figure 1; Holst, 2007). In LPS-substructures *O*3 and *O*7 glycosylations are the most important with *O*4 phosphorylation being a widespread motif. However, *O*6 and *O*2 modifications (e.g., deoxy) have also been targeted for specific biological purposes in the past (Kosma, 2008; Tikad and Vincent, 2013).

In Nature, l,d-heptose substructures are produced from the 6-epimeric structure d-*glycero*-d-*manno*-heptose. Thus, structures containing both d-*manno*-heptoses have been targeted and have for example been studied in terms of binding to the cross-reactive antibacterial monoclonal antibody WN1 222-5 (Di Padova et al., 1993; Gomery et al., 2012), for their interactions with C-type lectins (Jaipuri et al., 2008; Wang et al., 2008; Šulák et al., 2011; Marchetti et al., 2012) and in respect to their roles as potential diagnostic tools for bacterial infections (Anish et al., 2013).

Historically, access to the parent l,d- and d,d-heptoses has required 6-8 step synthesis from commercially available shorter chain sugars, thus creating a substantial entry-burden to this increasingly important research area. In the light of increasing antibiotic resistance, the awareness of the importance of heptoside containing fragments has grown rapidly and as such synthetic efforts in the field have evolved from niche-existence into more mainstream focus, reflected by recent reports across a wider application base. However, these new approaches generally stuck with the principle strategy of long synthetic routes, establishing complex protecting group designs alongside the chain elongation steps toward the heptose backbone. These synthetic efforts, although chemically elegant, do not constitute a general solution to the lack of easy access to common heptose motifs being applicable for multiple purposes (Brimacombe and Kabir, 1986; Segerstedt et al., 2004; Ohara et al., 2010; Anish et al., 2013; Inuki et al., 2017).

We strongly believed that what was required was a fundamental alteration in mind-set and revolutionary change of approach to create a simplified entry to this important field. Ideally, the desired heptoses should be available directly off the shelf as are standard hexoses constituting mammalian glycans, for which efficient and partly automated approaches are already state of the art today. This would allow for an entirely different level of sophistication in terms of questions posed and streamlining the methods applied in answering them.

**Scheme 1:**
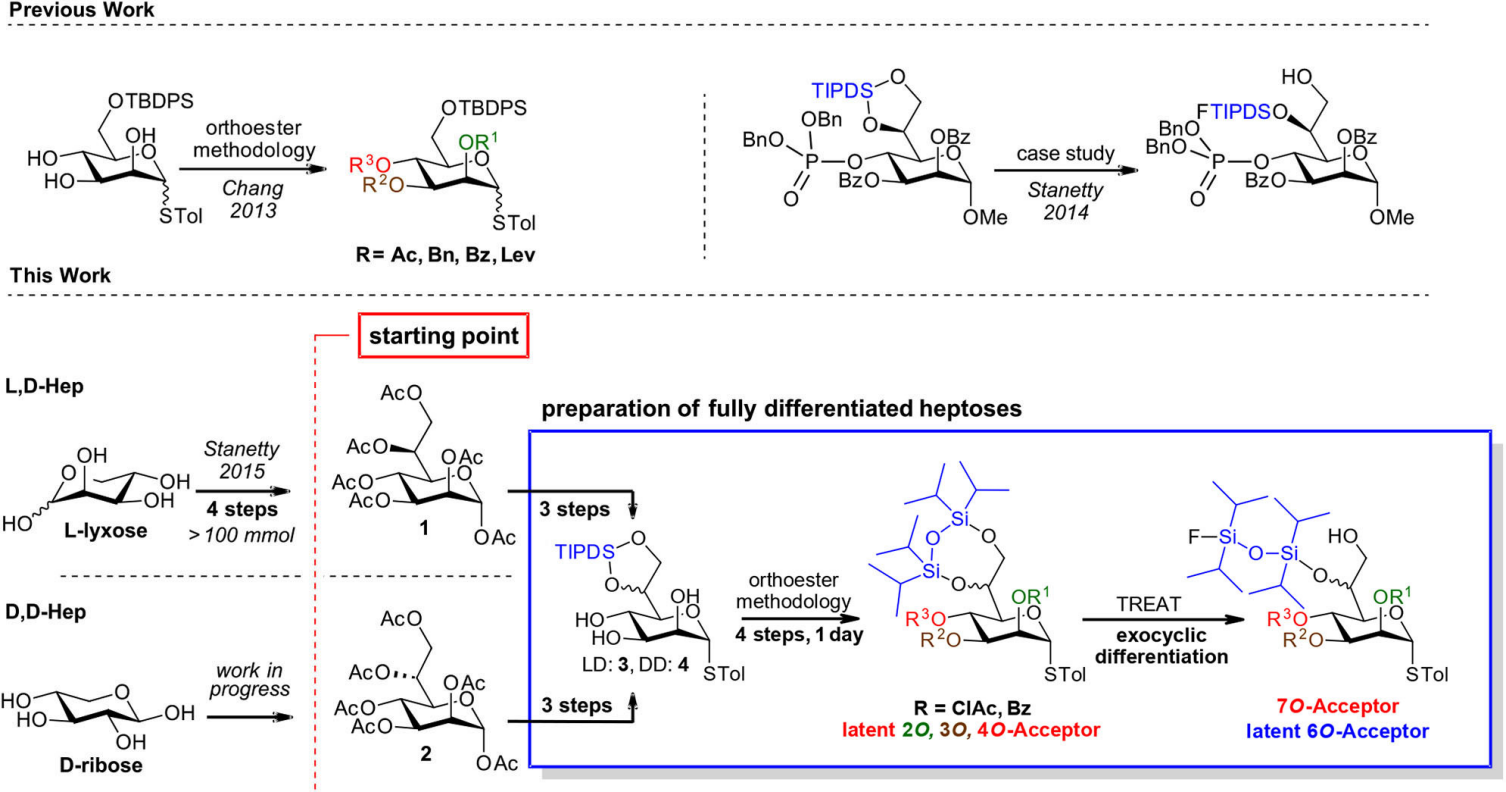
General strategy based on bulk *manno*-heptose starting pools 3 and 4 and a common differentiation methodology for both d,d and l,d-*manno* heptosides.

Toward our aspirations in this area we recently reported the first short and fully scalable synthetic approach to l,d-heptose peracetate based on indium mediated acyloxyallylation of l-lyxose and demonstrated it on a >100 mmol (45 g) scale (Stanetty and Baxendale, 2015). We are currently developing methodology for the analogous preparation of d,d-heptose peracetate (Aronow et al., 2019), with the intention of paving the way to make both d-*manno*-heptoses commercially available as their crystalline, bench-stable pyranose peracetate derivatives. Availability, be it commercial or at least through synthetically straightforward chemistry, is however only the first piece of a much larger puzzle (Scheme 1, bottom-left).

Thus, the focus of this publication is to underline the suitability of the peracetates **1** and **2** as starting points for different types of decorated heptose structures required for molecular probes or as donor/acceptors for oligoheptoside assembly. Despite tremendous advances in glycosylation techniques, it is still common to resort to a systematic trial and error search to come up with optimized conditions to install in high yield a specific glycosidic bond in a stereoselective fashion. Rapid change of decoration pattern can be a decisive element toward ultimate success in high yielding oligosaccharide formation.

We therefore believe that common starting materials for the late-stage decoration of the d,d- as well as the l,d-scaffolds are required and to this need we suggest the tetraisopropyl disiloxyl (TIPDS) protected *manno*-heptoses (**3** and **4**) as suitable examples of such compounds. We suggest diversification based on orthoester methodology and a recently introduced regioselective TIPDS-cleavage.

In *cis*-*trans*-triols, the *cis*-diol can be selectively addressed to form orthoesters which upon acidic hydrolysis yield the axial esters in high selectivity (King and Allbutt, 1970). In the presence of the intermediate orthoester several protecting groups can be installed under basic conditions and after orthoester cleavage another group can be installed at the equatorial hydroxyl group. Such an approach was recently demonstrated comprehensively on mannosides (Chang et al., 2013), and has also been used in target oriented syntheses of heptosides (Stanetty et al., 2014; Walter et al., 2017). The regioselective TIPDS cleavage on the other hand was originally introduced by the Ziegler group (Ziegler and Eckhardt, 1992; Ziegler et al., 1994) on standard hexoses and was successfully transferred to the exocyclic diol of the *manno*-heptose scaffold within a case study with an exceptionally bulky *O*4 group in place (see Scheme 1, top-right) and demonstrated there at preparative scale (Stanetty et al., 2014).

Within this current work, we set out to demonstrate the general value of unifying these two methods, exemplifying the orthoester methodology by permutating the labile chloroacetate group over the heptose core, and showcasing that the regioselective partial cleavage of the TIPDS at the exocyclic diol is applicable with other smaller 4*O*-substitutents as well as for both the l,d- and d,d-heptose scaffold (see Scheme 1, bottom-right).

## Results and Discussion

### Preparation of Starting Materials for Diversification

**Scheme 2:**
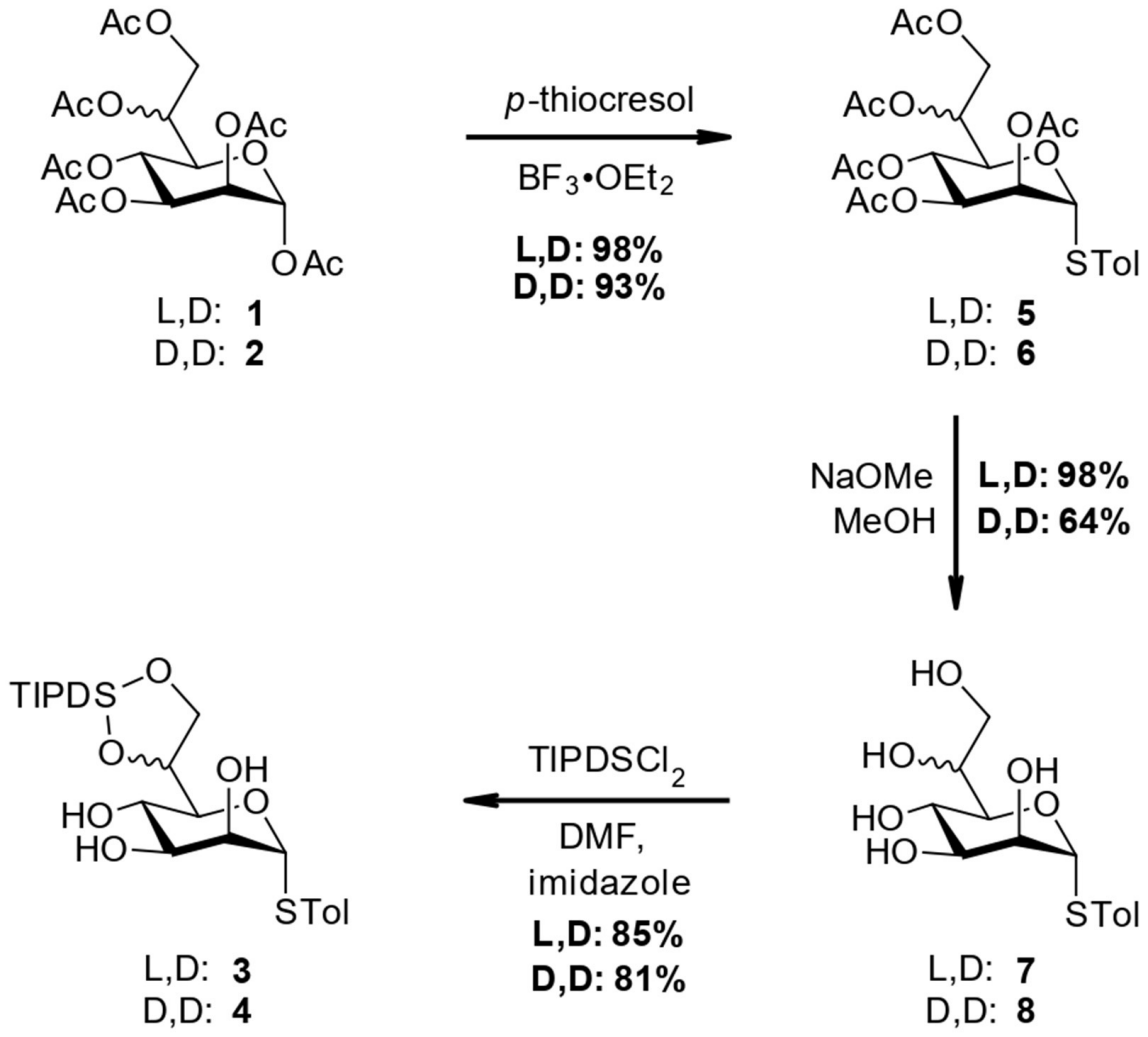
Preparation of triol-intermediates **3** and **4**.

Starting from the peracetylated heptose species **1** and **2** standard reaction conditions were used to introduce the STol group at the anomeric center (**5** and **6**). Further deacetylation using Zemplén conditions gave the pentaols **7** and **8**. At this stage the l,d-material **7** was obtained in near quantitative yield after recrystallization, while for the recrystallization of d,d-material only moderate recoveries (64%) were accomplished (see Scheme 2).

**Scheme 3:**
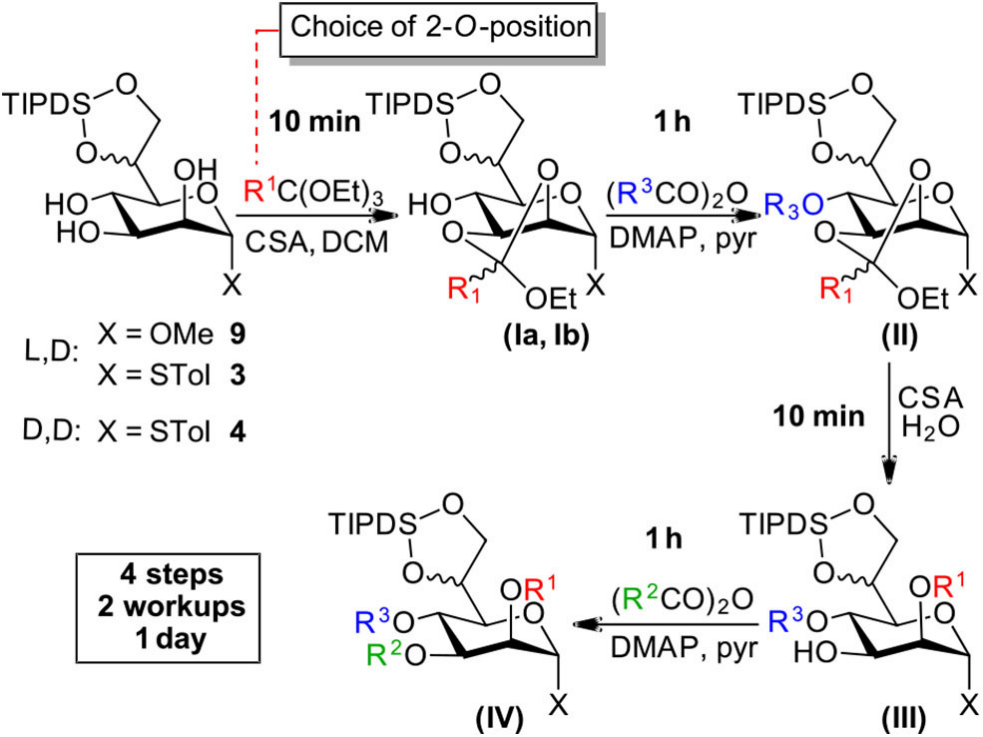
Efficient and versatile orthoester methodology.

Next, the TIPDS group was selectively introduced using a combination of TIPDSCl_2_ and imidazole at −78 °C, following conditions originally developed by Corey and Venkateswarlu (1972). Alternative conditions as described by Ziegler et al. (1994) employing pyridine at 0 °C are operationally simpler but gave lower yields for our compounds (ca. 60%). In addition to the thioglycosides **3** and **4**, we also included the TIPDS protected methyl heptoside **9**, which had also been used in the original case study to evaluate potential effects arising from the anomeric position (Stanetty et al., 2014; see Scheme 3 for structure **9**).

### Core Differentiation by an Orthoester Methodology

Starting from compounds **3**, **4**, and **9** we set out to develop a robust protocol for the rapid differentiation of the *manno*-configured triol based on an orthoester methodology and to exemplify this chemistry by the installation of the labile chloroacetate ester on all endocyclic ring-positions. Herein, we present a reliable and well-understood protocol specifically optimized for this class of the bacterial *manno*-heptoses (Scheme 3).

All four reaction steps of the protocol can be performed in dry DCM and intermediate aqueous workup is only required after the protection at *O*4 (structure II) and *O*3 (structure IV). The overall process can be accomplished within a working day with subsequent purification by standard column chromatography. However, steps 2 and 4 can also be safely left running overnight and intermediate purification is possible after step 2 and/or 3 if desired.

#### Step 1—Orthoester Formation

The formation of the orthoester intermediates is highly selective and is completed within minutes. Subsequently the sulfonic acid catalyst reacts readily with the orthoester reagents to form the sulfonic acid esters^1^. Generally, the two diastereomers (**Ia** and **Ib**) are observed as separate spots on TLC (e.g., LP/EtOAc 4:1, R_f_ 0.55 and 0.60). We found the addition of a small amount of Et_3_N prior to evaporation of the solvent is advisable to prevent any undesired orthoester cleavage. Co-evaporation from toluene facilitates removal of residual ethanol, present from the orthoester introduction, which if left, would consume acylating agent in the subsequent step^2^.

#### Step 2—Acylation at *O*4

At the *O*4 position acetates, benzoates and chloroacetates were introduced via the corresponding anhydrides using pyridine [Et_3_N is not compatible with (ClAc)_2_O!] and DMAP as a catalyst, which proved necessary for the sterically restricted position close to the TIPDS-group. Interestingly, while 2*O*/3*O*-orthoacetates and orthobenzoates with unprotected 4-OH group (**I**) were instantaneously hydrolysed in the presence of water and acid, upon 4*O*-protection the orthoester moieties (**II**) was stable to washing with 1 N HCl during extractive workup and column chromatography (Stanetty et al., 2014).

#### Step 3—Orthoester Hydrolysis

Under homogenous conditions in the presence of catalytic amounts of CSA or TsOH and with only a few equivalents of water the orthoester moiety was cleaved within minutes to provide 2*O*-ester (**III**).

#### Step 4—Acylation at *O*3

The final acylation follows the same principles as in step 2 and can generally follow step 3 directly, but evaporation and re-dissolution in DCM prevents hydrolysis of the acylating agent which gives a cleaner reaction.

We have applied this 4-step protocol at 1 mmol scale to starting materials **3**, **4**, and **9** to prepare a set of compounds (see Table 1) with overall yields of 60–81% reflecting at least 88% yield per step. The process can equally be applied to the methyl glycoside (entries 1 and 2) and thioglycosides and allows the installation for example the labile chloroacetyl group on position *O*3 and *O*4 but proved troublesome at the *O*2-position. We included a 4*O*-chloroacetate (entry 7) as an example with d,d-configuration for comparison in particular in the later TIPDS-cleavage.

**Table 1:** Decoration of L,D-Hep (1 mmol scale).

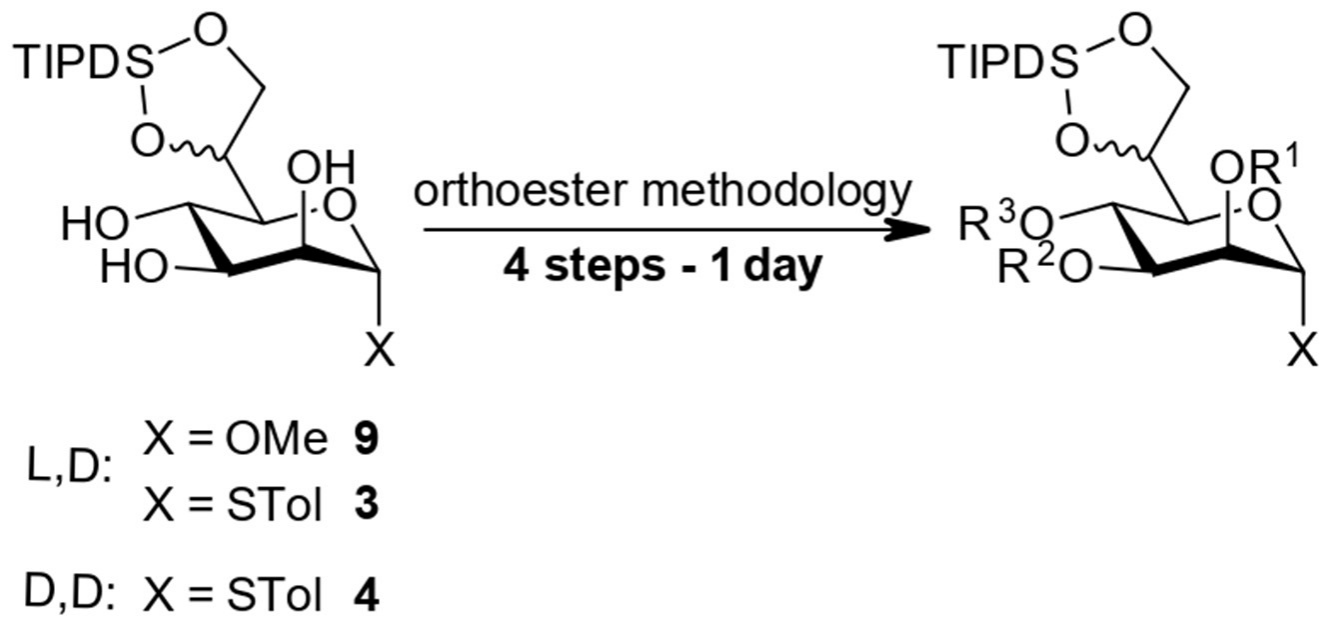						
**Entry**	**Start. mat**.	**R^**1**^**	**R^**2**^**	**R^**3**^**	**Product**	**Yield%**
1	**9**	Ac	Ac	ClAc	**10**	81
2	**9**	Bz	ClAc	Bz	**11**	81
3	**3**	Bz	Bz	ClAc	**12**	75
4	**3**	Bz	ClAc	Bz	**13**	69
5^a^	**3**	ClAc orthoester		H	**14a,b**	64
6^a^	**3**	ClAc orthoester		Bz	**15a,b**	55
7	**4**	Bz	Bz	ClAc	**16**	60

a *Only orthoester formation on smaller scale (entry 5) and subsequent benzoylation (entry 6, 2-step yield) was performed*.

### Stability of the Ortho Chloroacetate

The aim of introducing the labile chloroacetate ester at the *O*2-position could not be directly accomplished as the intermediate ortho chloroacetate exhibited surprising stability and withstood the standard conditions both as 4*O-*Bz (**15**) as well as the 4-OH (**14**) intermediate. Under harsher, previously reported conditions (80% AcOH or CF_3_COOH) (Oscarson and Tedebark, 1996; Ueki et al., 2010) multiple unidentified products were observed with both orthoesters **14a,b** and **15a,b**. The problem was identified as arising from the presence of the TIPDS group (see Supplementary Material).

Nevertheless, the high acid stability of the ortho chloroacetate moieties allowed chromatographic separation of both diastereomeric pairs **14a,b** and **15a,b** and full characterization thereof. We suggest, that the ortho chloroacetate can be considered as a selective long-term protecting group for the 2*O*,3*O*-diol of the *manno*-scaffold. It tolerates transformations under slightly acidic conditions which cannot be performed with the usual orthoesters which we also demonstrated by including **15** in our TIPDS cleavage study (*vide infra*). To also be able to include the originally targeted 3*O*,4*O*-dibenzoyl-2*O*-chloroacetyl species **16** (see Table 2 for structure) in the survey we have synthesized it by a lengthier synthetic route (see Supplementary Material).

**Table 2:** Regioselective cleavage of the TIPDS group.

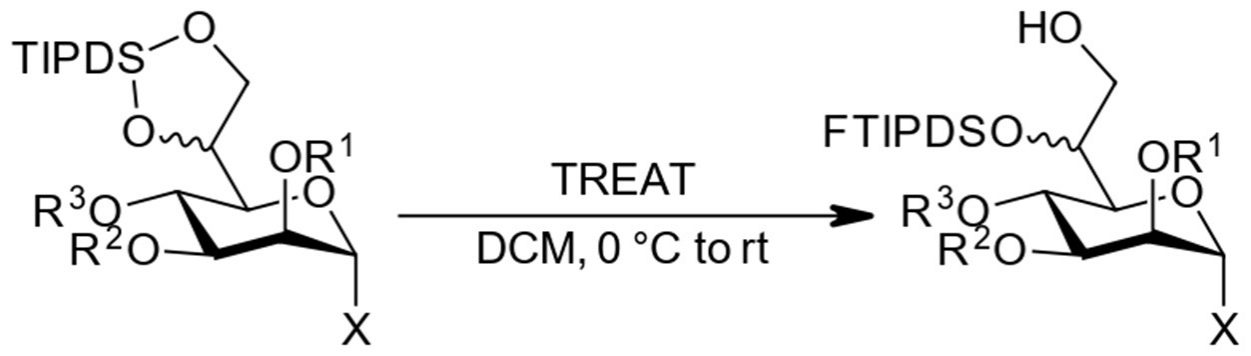							
	**Start. mat**.	**X**	**R^**1**^**	**R^**2**^**	**R^**3**^**	**Product**	**Yield%**
l,d-series	**11**	OMe	Bz	ClAc	Bz	**20**	49
	**21**	STol	Bz	Bz	Bz	**22**	69
	**23**	STol	Ac	Ac	Ac	**24**	64
	**12**	STol	Bz	Bz	ClAc	**25**	Mig
	**13**	STol	Bz	ClAc	Bz	**26**	70
	**15a**	STol	orthoester		Bz	**27**	75
	**17**	STol	ClAc	Bz	Bz	**28**	24^a^
d,d-series	**29**	STol	Bz	Bz	Bz	**30**	57
	**31**	STol	Ac	Ac	Ac	**32**	68
	**16**	STol	Bz	Bz	ClAc	**33**	68

a *The low yield is due losses in purification at the substantially smaller scale, not selectivity as judged by crude ^1^H-NMR*.

### Regioselective TIPDS-Cleavage to Provide 7-OH Acceptors

With the compounds obtained via the orthoester protocol and the additional per-benzoylated and per-acetylated model compounds (**21**, **23**, **29**, **31** in Table 2), we set out to systematically investigate the regioselective TIPDS cleavage toward 7-OH acceptors using triethylamine trihydrofluoride (TREAT) as a mild fluoride source. We commenced the investigation with the l,d-configured methyl and STol glycosides (see Table 2, top entries) under the conditions from the case study. The targeted compound could generally be obtained as the major product in yields between 50–75% independent of the ester group at *O*4 (confirming that the bulky dibenzyl phosphate group of the case study was not a prerequisite for this attractive transformation, see Scheme 1). Successful TIPDS cleavage at the *O*7 position can be confirmed in the NMR by a new COSY signal between 7-OH and the H7 protons. Further, a diagnostic shift in the ^13^C-NMR by 5 ppm highfield for C7 is observed. Experimentally, the reactions were performed in dry DCM at 0 °C in a teflon vessel to prevent uncontrolled quenching of the fluoride reagent on glass. There is an inherent over-reaction toward complete desilylation (there was no indication of opening toward 6-OH acceptors) which requires TLC monitoring of the three main components (starting material, target compound, and fully deprotected by-product) for optimal results. Sufficient stirring combined with slow addition of the TREAT prevents overreaction. When prolonged stirring at 0 °C led to sluggish conversion more TREAT was added in portions until completion as determined by TLC.

When studying this reaction for the d,d-heptoses, a simple modification of conditions was applied. Slow warming of the reaction to room temperature following addition of the TREAT led to complete conversion in a short time frame; comparable yields to other protocol were also obtained. In Table 2 we have summarized our findings.

Independent of the protocol, most transformations performed as expected and with good selectivity toward initial attack at the “primary” side of the disiloxane ring system. However, the reaction toward l,d-configured compound **25**, where the labile chloroacetate group is attached to the 4*O*-position, partial migration during workup, and/or purification to the 7*O*-position was observed. Interestingly, in the reaction with the d,d-analog **16** this migration was not observed and a good isolated yield was obtained. We assume that the stereochemistry at 6*O*-position with the bulky FTIPDSO-group leads to a conformational change in the side-chain and thus prevents proximity between the 7-OH and the particularly reactive chloroacetate at *O*4 which tentatively favors migration in the l,d-case. Also, the other two examples with d,d-configuration, perbenzoate **29**, and peracetate **31** gave comparable selectivities and isolated yields to their l,d-counterparts, confirming the independence of the size of the *O*4-substitutent also with this configuration. When the ortho chloroacetate **15a** was submitted to the reaction conditions, we were pleased to note that the acid stability was sufficient to cleanly prepare the corresponding 7-OH acceptor **27**.

Despite the somewhat lower yields compared to the original example in our case study, the simultaneous *O*6/*O*7-protection together with the differentiation between those two positions in one step is an attractive feature of the overall approach. Noteworthy, performance in this interesting transformation was comparable between the two epimeric families, which supports this methodology for future use in both *manno*-heptose families alike.

## Conclusion

We are convinced that the field of preparative *manno*-heptose chemistry would benefit greatly from a shift of paradigm toward common parent starting materials (e.g., peracetates) and differentiation thereof. In this light, we present short and scalable protocols to both (l,d, d,d) crystalline STol-heptosides and promote the utilization of the TIPDS-group as a protection for their exocyclic diols. This is due to its attractive partial regioselective cleavage which was proven to be a generally applicable solution for the differentiation between *O*6/*O*7. Additionally, we report an efficient 4-steps/1-purification protocol based on orthoester methodology to achieve the prior differentiation of the sugar core triol. The unforeseen stability of the ortho chloroacetates in this context was exploited by showing its applicability as stable protecting group during the TIPDS cleavage. We hope that the presented approach will pave the way towards a more unified starting point into biologically relevant structures based on *manno*-heptoses.

## Data Availability Statement

The original contributions presented in the study are included in the article/Supplementary Material, further inquiries can be directed to the corresponding author/s.

## Author Contributions

CSu and CSt have been preparing and analyzing the materials. MM and IB have contributed with advice. All authors contributed to the article and approved the submitted version.

## Conflict of Interest

The authors declare that the research was conducted in the absence of any commercial or financial relationships that could be construed as a potential conflict of interest.

## Footnotes

^1^Due to this side reaction the choice of sulfonic acid catalyst was made based on the chromatographic properties of the resultant ester in respect to the final target compound which is to be columned.

^2^Formation of 2*O*-esters is also partly observed on TLC, which should not be mistaken for an incomplete orthoester formation. After addition of TEA such decomposition on TLC is not observed any more.
