# Supplementary material for: Straight Forward and Versatile Differentiation of the l-glycero and d-glycero-d-manno Heptose Scaffold
Source: Front Chem. 2020 Jul 31;8:625. doi: 10.3389/fchem.2020.00625 (PMC7411327; doi:10.3389/fchem.2020.00625)

Straight forward and versatile differentiation of the L-*glycero* and D-*glycero*-D-*manno* heptose scaffold

Christoph Suster[1], Ian R. Baxendale[2], Marko D Mihovilovic[1], Christian Stanetty[1]*

[1]*Institute of Applied Synthetic Chemistry, TU Wien, Getreidemarkt 9, 1060 Vienna, Austria*
[2]*Department of Chemistry, Durham University, Durham, United Kingdom*

e-mail: christian.stanetty@tuwien.ac.at

Content

A. General Information 5
B. Experimental 5

B.1. Proving the incompatibility of the TIPDS-Group with chloracetate orthoester cleaving conditions. 5

B.2. Preparation of triol starting materials 3 and 4 6

B.2.1. Synthesis of tol-4-yl 2,3,4,6,7-penta-*O*-acetyl-1-thio-L-*glycero*-α-D-*manno*-heptopyranoside (5) 6
B.2.2. Synthesis of tol-4-yl 1-thio-L-*glycero*-α-D-*manno*-heptopyranoside (7) 6
B.2.3. Synthesis of tol-4-yl 6,7-*O*-(1,1,3,3-tetraisopropyl-1,3-disiloxane-1,3-diyl)-1-thio-L-*glycero*-α-D-*manno*-heptopyranoside (3) 7
B.2.4. Synthesis of tol-4-yl 2,3,4,6,7-penta-*O*-acetyl-1-thio-D-*glycero*-α-D-*manno*-heptopyranoside (6) 8
B.2.5. Synthesis of tol-4-yl 1-thio-D-*glycero*-α-D-*manno*-heptopyranoside (8) 8
B.2.6. Synthesis of tol-4-yl 6,7-*O*-(1,1,3,3-tetraisopropyl-1,3-disiloxane-1,3-diyl)-1-thio-D-*glycero*-α-D-*manno*-heptopyranoside (4) 9

B.3. Decoration of endocyclic ring positions 10

B.3.1. Synthesis of methyl 2,3-di-*O*-acetyl-4-*O*-chloroacetyl-6,7-*O*-(1,1,3,3-tetraisopropyl-1,3-disiloxane-1,3-diyl)-L-*glycero*-α-D-*manno*-heptopyranoside (10) 10
B.3.2. Synthesis of methyl 2,4-di-*O*-benzoyl-3-*O*-chloroacetyl-6,7-*O*-(1,1,3,3-tetraisopropyl-1,3-disiloxane-1,3-diyl)-L-*glycero*-α-D-*manno*-heptopyranoside (11) 11
B.3.3. Synthesis of tol-4-yl 2,3-di-*O*-benzoyl-4-*O*-chloroacetyl-6,7-*O*-(1,1,3,3-tetraisopropyl-1,3-disiloxan-1,3-diyl)-1-thio-L-*glycero*-α-D-*manno*-heptopyranoside (12) 12
B.3.4. Synthesis of tol-4-yl 2,4-di-*O*-benzoyl-3-*O*-chloroacetyl-6,7-*O*-(1,1,3,3-tetraisopropyl-1,3-disiloxane-1,3-diyl)-1-thio-L-*glycero*-α-D-*manno*-heptopyranoside (13) 13

B.3.5. Synthesis of tol-4-yl 2,3-*O*-(2-chloro-1-ethoxyethyliden-1,1-diyl)-6,7-*O*-(1,1,3,3-tetraisopropyl-1,3-disiloxane-1,3-diyl)-1-thio-L-*glycero*-α-D-*manno*-heptopyranoside . (14a), (14b) ... 14

B.3.6. Synthesis of tol-4-yl 4-*O*-benzoyl-2,3-*O*-(2-chloro-1-ethoxyethyliden-1,1-diyl)-6,7-O(1,1,3,3-tetraisopropyl-1,3-disiloxane-1,3-diyl)-1-thio-L-*glycero*-α-D-*manno*-heptopyranoside (15a), (15b) ... 15

B.3.7. Synthesis of tol-4-yl 2,3-di-*O*-benzoyl-4-*O*-chloroacetyl-6,7-*O*-(1,1,3,3-tetraisopropyl-1,3-disiloxan-1,3-diyl)-1-thio-D-*glycero*-α-D-*manno*-heptopyranoside (16) ... 16

B.3.8. Synthesis of tol-4-yl 3-*O*-benzoyl-6,7-*O*-(1,1,3,3-tetraisopropyl-1,3-disiloxan-1,3-diyl)-1-thio-L-*glycero*-α-D-*manno*-heptopyranoside (18) ... 18

B.3.9. Synthesis of tol-4-yl 3-*O*-benzoyl-2-*O*-chloroacetyl-6,7-*O*-(1,1,3,3-tetraisopropyl-1,3-disiloxan-1,3-diyl)-1-thio-L-*glycero*-α-D-*manno*-heptopyranoside (19) ... 19

B.3.10. Synthesis of tol-4-yl 3,4-di-*O*-benzoyl-2-*O*-chloroacetyl-6,7-*O*-(1,1,3,3-tetraisopropyl-1,3-disiloxan-1,3-diyl)-1-thio-L-*glycero*-α-D-*manno*-heptopyranoside (17) ... 20

B.3.11. Synthesis of tol-4-yl 2,3,4-tri-*O*-benzoyl-6,7-*O*-(1,1,3,3-tetraisopropyl-1,3-disiloxane-1,3-diyl)-1-thio-L-*glycero*-α-D-*manno*-heptopyranoside (21) ... 21

B.3.12. Synthesis of tol-4-yl 2,3,4-tri-*O*-benzoyl-6,7-*O*-(1,1,3,3-tetraisopropyl-1,3-disiloxane-1,3-diyl)-1-thio-D-*glycero*-α-D-*manno*-heptopyranoside (29) ... 21

B.3.13. Synthesis of tol-4-yl 2,3,4-tri-*O*-acetyl-6,7-*O*-(1,1,3,3-tetraisopropyl-1,3-disiloxane-1,3-diyl)-1-thio-D-*glycero*-α-D-*manno*-heptopyranoside (31) ... 22

B.4. Regioselective partial cleavage of the TIPDS group ... 23

B.4.1. Synthesis of methyl 2,4-di-*O*-benzoyl-3-*O*-chloroacetyl-6-*O*-(3-fluoro-1,1,3,3-tetraisopropyl-1,3-disiloxane-1-yl)-L-*glycero*-α-D-*manno*-heptopyranoside (20) ... 23

B.4.2. Synthesis of tol-4-yl 2,3,4-tri-*O*-benzoyl-6-*O*-(3-fluoro-1,1,3,3-tetraisopropyl-1,3-disiloxane-1-yl)- 1-thio-L-*glycero*-α-D-*manno*-heptopyranoside (22) and tol-4-yl 2,3,4-tri-*O*-benzoyl-1-thio-L-*glycero*-α-D-*manno*-heptopyranoside (22a) ... 24

B.4.3. Synthesis of tol-4-yl 2,3,4-tri-*O*-acetyl-6-*O*-(3-fluoro-1,1,3,3-tetraisopropyl-1,3-disiloxane-1-yl)-1-thio-L-*glycero*-α-D-*manno*-heptopyranoside (24) and tol-4-yl 2,3,4-tri-*O*-acetyl-1-thio-L-*glycero*-α-D-*manno*-heptopyranoside (24a) ... 25

B.4.4. Synthesis of tol-4-yl 2,4-di-*O*-benzoyl-3-*O*-chloroacetyl-6-*O*-(3-fluoro-1,1,3,3-tetraisopropyl-1,3-disiloxane-1-yl)- 1-thio-L-*glycero*-α-D-*manno*-heptopyranoside (26) ... 26

B.4.5. Synthesis of tol-4-yl 2,3-*O*-(2-chloro-1-ethoxy-ethyliden-1,1-diyl)- 6-*O*-(3-fluoro-1,1,3,3-tetraisopropyl-1,3-disiloxane-1-yl)-1-thio-L-*glycero*-α-D-*manno*-heptopyranoside (27) ... 26

B.4.6. Synthesis of tol-4-yl 3,4-di-*O*-benzoyl-2-*O*-chloroacetyl-6-*O*-(3-fluoro-1,1,3,3-tetraisopropyl-1,3-disiloxane-1-yl)- 1-thio-L-*glycero*-α-D-*manno*-heptopyranoside (28) ... 27

B.4.7. Synthesis of tol-4-yl 2,3,4-tri-*O*-benzoyl-6-*O*-(3-fluoro-1,1,3,3-tetraisopropyl-1,3-disiloxane-1-yl)-1-thio-D-*glycero*-α-D-*manno*-heptopyranoside (30) ... 28

B.4.8. Synthesis of tol-4-yl 2,3,4-tri-*O*-acetyl-6-*O*-(3-fluoro-1,1,3,3-tetraisopropyl-1,3-disiloxane-1-yl)- 1-thio-D-*glycero*-α-D-*manno*-heptopyranoside (32) ... 29

B.4.9. Synthesis of tol-4-yl 2,3-di-*O*-benzoyl-4-*O*-chloroacetyl-6-*O*-(3-fluoro-1,1,3,3-tetraisopropyl-1,3-disiloxane-1-yl)- 1-thio-D-*glycero*-α-D-*manno*-heptopyranoside (33) ... 29

C. Recorded NMR-spectra ... 30

C.1. Preparation of triol starting materials ... 30

C.1.1. Tol-4-yl 2,3,4,6,7-penta-*O*-acetyl-1-thio-L-*glycero*-α-D-*manno*-heptopyranoside (5) ... 30

C.1.2. Tol-4-yl 1-thio-D-*glycero*-α-D-*manno*-heptopyranoside (7) ... 31

C.1.3. Tol-4-yl 6,7-*O*-(1,1,3,3-tetraisopropyl-1,3-disiloxane-1,3-diyl)-1-thio-L-*glycero*-α-D-*manno*-heptopyranoside (3) ... 32

C.1.4. Tol-4-yl 2,3,4,6,7-penta-*O*-acetyl-1-thio-D-*glycero*-α-D-*manno*-heptopyranoside (6) ... 33

C.1.5. Tol-4-yl 1-thio-D-*glycero*-α-D-*manno*-heptopyranoside (8) ... 34

C.1.6. Tol-4-yl 6,7-*O*-(1,1,3,3-tetraisopropyl-1,3-disiloxane-1,3-diyl)-1-thio-D-*glycero*-α-D-*manno*-heptopyranoside (4) 35

C.2. Fully Decorated TIPDS sugars 36

C.2.1. Methyl 2,3-di-*O*-acetyl-4-*O*-chloroacetyl-6,7-*O*-(1,1,3,3-tetraisopropyl-1,3-disiloxane-1,3-diyl)-L-*glycero*-α-D-*manno*-heptopyranoside (10) 36

C.2.2. Methyl 2,4-di-*O*-benzoyl-3-*O*-chloroacetyl-6,7-*O*-(1,1,3,3-tetraisopropyl-1,3-disiloxane-1,3-diyl)-L-*glycero*-α-D-*manno*-heptopyranoside (11) 38

C.2.3. Tol-4-yl 2,3-di-*O*-benzoyl-4-*O*-chloroacetyl-6,7-*O*-(1,1,3,3-tetraisopropyl-1,3-disiloxane-1,3-diyl)-1-thio-L-*glycero*-α-D-*manno*-heptopyranoside (12) 39

C.2.4. Tol-4-yl 2,4-di-*O*-benzoyl-3-*O*-chloroacetyl-6,7-*O*-(1,1,3,3-tetraisopropyl-1,3-disiloxane-1,3-diyl)-1-thio-L-*glycero*-α-D-*manno*-heptopyranoside (13) 40

C.2.5. Tol-4-yl 2,3-*O*-(2-chloro-1-ethoxyethyliden-1,1-diyl)-6,7-*O*-(1,1,3,3-tetraisopropyl-1,3-disiloxane-1,3-diyl)-1-thio-L-*glycero*-α-D-*manno*-heptopyranoside – Isomere A (14a) 42

C.2.6. Tol-4-yl 2,3-*O*-(2-chloro-1-ethoxyethyliden-1,1-diyl)-6,7-*O*-(1,1,3,3-tetraisopropyl-1,3-disiloxane-1,3-diyl)-1-thio-L-*glycero*-α-D-*manno*-heptopyranoside – Isomere B (14b) 43

C.2.7. Tol-4-yl 4-*O*-benzoyl-2,3-*O*-(2-chloro-1-ethoxyethyliden-1,1-diyl)-6,7-*O*-(1,1,3,3-tetraisopropyl-1,3-disiloxane-1,3-diyl)-1-thio-L-*glycero*-α-D-*manno*-heptopyranoside – Isomere A (15a) 44

C.2.8. Tol-4-yl 4-*O*-benzoyl-2,3-*O*-(2-chloro-1-ethoxyethyliden-1,1-diyl)-6,7-*O*-(1,1,3,3-tetraisopropyl-1,3-disiloxane-1,3-diyl)-1-thio-L-*glycero*-α-D-*manno*-heptopyranoside – Isomere B (15b) 46

C.2.9. Tol-4-yl 2,3-di-*O*-benzoyl-4-*O*-chloroacetyl-6,7-*O*-(1,1,3,3-tetraisopropyl-1,3-disiloxan-1,3-diyl)-1-thio-D-*glycero*-α-D-*manno*-heptopyranoside (16) 48

C.2.10. Tol 4-yl 3-*O*-benzoyl-6,7-*O*-(1,1,3,3-tetraisopropyl-1,3-disiloxan-1,3-diyl)-1-thio-L-*glycero*-α-D-*manno*-heptopyranoside (18) 49

C.2.11. Tol 4-yl 3-*O*-benzoyl-2-*O*-chloroacetyl-6,7-*O*-(1,1,3,3-tetraisopropyl-1,3-disiloxan-1,3-diyl)-1-thio-L-*glycero*-α-D-*manno*-heptopyranoside (19) 50

C.2.12. Tol 4-yl 3-*O*-benzoyl-2,4-*O*-dichloroacetyl-6,7-*O*-(1,1,3,3-tetraisopropyl-1,3-disiloxan-1,3-diyl)-1-thio-L-*glycero*-α-D-*manno*-heptopyranoside (by-product of 19) 51

C.2.13. Tol-4-yl 3,4-di-*O*-benzoyl-2-*O*-chloroacetyl-6,7-*O*-(1,1,3,3-tetraisopropyl-1,3-disiloxan-1,3-diyl)-1-thio-L-*glycero*-α-D-*manno*-heptopyranoside (17) 52

C.2.14. Tol-4-yl 2,3,4-tri-*O*-benzoyl-6,7-*O*-(1,1,3,3-tetraisopropyl-1,3-disiloxane-1,3-diyl)-1-thio-L-*glycero*-α-D-*manno*-heptopyranoside (21) 53

C.2.15. Tol-4-yl 2,3,4-tri-*O*-benzoyl-6,7-*O*-(1,1,3,3-tetraisopropyl-1,3-disiloxane-1,3-diyl)-1-thio-D-*glycero*-α-D-*manno*-heptopyranoside (29) 54

C.2.16. Tol-4-yl 2,3,4-tri-*O*-acetyl-6,7-*O*-(1,1,3,3-tetraisopropyl-1,3-disiloxane-1,3-diyl)-1-thio-D-*glycero*-α-D-*manno*-heptopyranoside (31) 55

C.3. Regioselective partial cleavage of TIPDS 56

C.3.1. Methyl 2,4-di-*O*-benzoyl-3-*O*-chloroacetyl-6-*O*-(3-fluoro-1,1,3,3-tetraisopropyl-1,3-disiloxane-1-yl)-L-*glycero*-α-D-*manno*-heptopyranoside (20) 56

C.3.2. Tol-4-yl 2,3,4-tri-*O*-benzoyl-6-*O*-(3-fluoro-1,1,3,3-tetraisopropyl-1,3-disiloxane-1-yl)-1-thio-L-*glycero*-α-D-*manno*-heptopyranoside (22) 57

C.3.3. Tol-4-yl 2,3,4-tri-*O*-benzoyl-1-thio-L-*glycero*-α-D-*manno*-heptopyranoside (22a) 58

C.3.4. Tol-4-yl 2,3,4-tri-*O*-acetyl-6-*O*-(3-fluoro-1,1,3,3-tetraisopropyl-1,3-disiloxane-1-yl)-1-thio-L-*glycero*-α-D-*manno*-heptopyranoside (24) 59

C.3.5. Tol-4-yl 2,3,4-tri-*O*-acetyl-1-thio-L-*glycero*-α-D-*manno*-heptopyranoside (24a) 60

C.3.6. Tol-4-yl 2,4-di-*O*-benzoyl-3-*O*-chloroacetyl-6-*O*-(3-fluoro-1,1,3,3-tetraisopropyl-1,3-disiloxane-1-yl)-1-thio-L-*glycero*-α-D-*manno*-heptopyranoside (26) 61

C.3.7. Tol-4-yl 2,3-*O*-(2-chloro-1-ethoxyethyliden-1,1-diyl)- 6-*O*-(3-fluoro-1,1,3,3-tetraisopropyl-1,3-disiloxane-1-yl)-1-thio-L-*glycero*-α-D-*manno*-heptopyranoside (27) 63

C.3.8. Tol-4-yl 3,4-di-*O*-benzoyl-2-*O*-chloroacetyl-6-*O*-(3-fluoro-1,1,3,3-tetraisopropyl-1,3-disiloxane-1-yl)- 1-thio-L-*glycero*-α-D-*manno*-heptopyranoside (28) 64
C.3.9. Tol-4-yl 2,3,4-tri-*O*-benzoyl-6-*O*-(3-fluoro-1,1,3,3-tetraisopropyl-1,3-disiloxane-1-yl)- 1-thio-D-*glycero*-α-D-*manno*-heptopyranoside (30) 65
C.3.10. Tol-4-yl 2,3,4-tri-*O*-acetyl-6-*O*-(3-fluoro-1,1,3,3-tetraisopropyl-1,3-disiloxane-1-yl)-1-thio-D-*glycero*-α-D-*manno*-heptopyranoside (32) 66
C.3.11. Tol-4-yl 2,3-di-*O*-benzoyl-4-*O*-chloroacetyl-6-*O*-(3-fluoro-1,1,3,3-tetraisopropyl-1,3-disiloxane-1-yl)- 1-thio-D-*glycero*-α-D-*manno*-heptopyranoside (33) 67

A. General Information

All chemicals were directly used from commercial sources without further purification. Water-free solvents were available at the institute from a PureSolv EN 1-4 Enclosed solvent drying plant. Light petrol (LP) for column chromatography was distilled prior to use.

TLC analysis for reaction monitoring and analysing fractions from column chromatography was performed on silica gel 60 F254-plates or HPTLC-plates (silica gel 60 with concentration zone 20 × 2.5 cm). Visualisation on TLC plates was done, using UV light (254 nm) followed by staining with anisaldehyde solution (180 mL EtOH, 10 mL anisaldehyde, 10 mL H_2SO_4 conc., 2 mL AcOH).

NMR spectra were recorded at 297 K with the solvent indicated with an Avance Ultra Shield 400 MHz, an Avance III HD 600 MHz or a Varian V NMR S-700 MHz spectrometer. All spectra were calibrated to the solvent residual peaks. Chemical shifts (δ) and coupling constants (*J*) were expressed in ppm and Hz, respectively. Assignments are based on APT, COSY, HSQC, and HMBC spectra.

Low- and high-resolution mass spectrometry was performed by use of the indicated techniques on Waters LCT Premier XE or Waters TQD instruments equipped with Acquity UPLC and a lock-mass electrospray ion source.

Optical rotation was measured on an Anton Paar MCP 500 at the specified conditions, [α]D values are given in 10^{-1} deg cm^2 g^{-1}.

B. Experimental

B.1. Proving the incompatibility of the TIPDS-Group with chloracetate orthoester cleaving conditions.

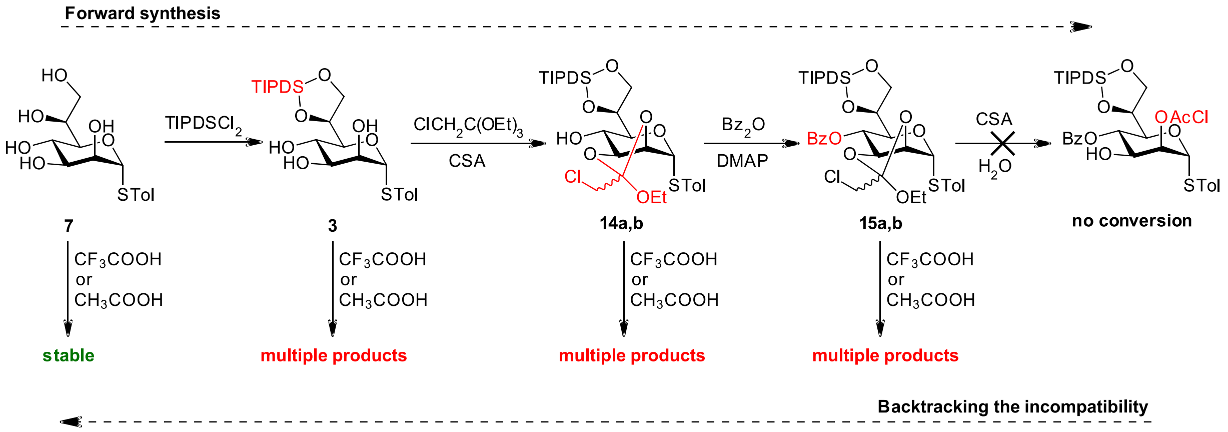

Scheme 1. Investigation for the incompatibility of the chloroacetate cleaving protocol, by backtracking the cause of starting material degradation.

Under standard treatment with CSA and water, no conversion was observed for both orthoester pairs **14** and **15**. Applying harsher conditions that had literature precedence for successful ortho chloroacetate cleavage (CF_3COOH or 80% CH_3COOH), gave multiple products on TLC. To investigate this observation, the precursors **3** and **7** were also subjected to the same conditions to see what parts of the molecule were not stable. Using this approach, it was possible to conclude, that the TIPDS group was degrading under those harsh acidic conditions, as only thioglycoside **7** proved stable, while **3** (with TIPDS group) gave multiple products too (see *Scheme 1*).

B.2. Preparation of triol starting materials 3 and 4

B.2.1. Synthesis of tol-4-yl 2,3,4,6,7-penta-*O*-acetyl-1-thio-L-*glycero*-α-D-*manno*-heptopyranoside (5)

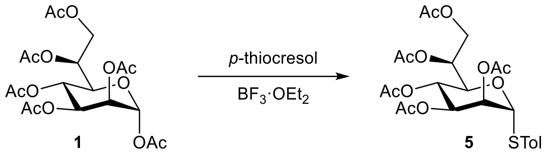

Procedure

The peracetate **1** (0.925 g, 2.00 mmol, 1.00 eq.) was dissolved in dry DCM (9.2 mL), *p*-thiocresol (0.348 g, 2.80 mmol, 1.40 eq.) and $BF_3 \cdot OEt_2$ (4.62 g, 10.0 mmol, 5.00 eq.) was added dropwise over a minute keeping the temperature at rt. The reaction mixture was stirred and monitored by TLC (LP/EtOAc 1:1, 1:2). After 20 h complete conversion of the starting material was achieved, and the reaction mixture was distributed between DCM and sat. $NaHCO_3$, the combined organic layer was washed with brine, dried over Na_2SO_4 and evaporated to give the crude material (1.3 g).

Purification

The crude material was purified *via* column chromatography (25 g SiO_2, 25 mL/min, gradient LP/EtOAc 2:1 to 1:2) to give target compound **5** with an anomeric ratio of α:β = 20:1 (1.03 g, 98 %).

Analytics

R_f-value: 0.32 (LP/EtOAc = 8:1)

$[\alpha]_D^{20}$: +45° (*c* 1.1, $CHCl_3$)

^1H NMR (700 MHz, $CDCl_3$) δ 7.32-7.28 (m, 2H, STol-H2/H6), 7.15-7.10 (m, 2H, STol-H3/H5), 5.53 (d, *J* = 1.4 Hz, 1H, H1), 5.51 (dd, *J* = 3.2, 1.4 Hz, 1H, H2), 5.36 (t, *J* = 10.1 Hz, 1H, H4), 5.30 (dd, *J* = 10.1, 3.4 Hz, 1H, H3), 5.31-5.26 (m, 1H, H6), 4.54 (dd, *J* = 9.9, 2.0 Hz, 1H, H5), 4.05 (dd, *J* = 11.4, 5.6 Hz, 1H, H7a), 4.00 (dd, *J* = 11.4, 7.6 Hz, 1H, H7b), 2.32 (s, 3H, STol-CH_3), 2.17, 2.12, 2.04, 2.00, 1.94 (5 × s, 5 × 3H, 5 × $COCH_3$)

^{13}C NMR (176 MHz, $CDCl_3$) δ 170.5, 170.3, 170.1, 169.9, 169.7 (5 × *C*OCH₃), 138.5 (STol-C), 131.6 (STol-C2/C6), 130.3 (STol-C), 128.8 (STol-C3/C5), 86.3 (C1), 71.1 (C2), 69.83 (C5), 69.76 (C3), 67.2 (C6), 65.2 (C4), 62.0 (C7), 21.2 (STol-CH_3), 21.1, 20.84, 20.78, 20.77, 20.74 (5 × $COCH_3$)

HRMS (+ESI) m/z: calc. for $C_{24}H_{30}O_{11}S$ $[M+Na]^+$: 549.1407, $[M+NH4]^+$: 544.1853 found: m/z = 549.1418; 544.1842

B.2.2. Synthesis of tol-4-yl 1-thio-L-*glycero*-α-D-*manno*-heptopyranoside (7)

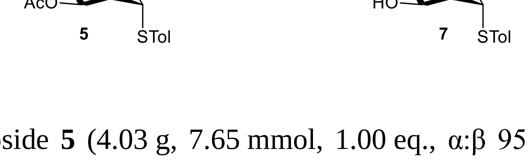

Procedure

The peracetylated thio-glycoside **5** (4.03 g, 7.65 mmol, 1.00 eq., α:β 95:5) was dissolved in MeOH (ca. 100 mL), NaOMe was added until pH remained basic (ca.200 mg) and the reaction mixture was stirred at rt under TLC monitoring ($CHCl_3$:MeOH:H_2O 14:6:1). Soon an increasing amount of white precipitate formed. A heterogenous sample was taken, diluted with MeOH/H_2O/AcOH until homogenous and analysed via TLC to show complete conversion to two new more polar spots. After 3 h the reaction mixture was neutralised by addition of freshly washed QP-SA leading to a clear solution. Filtration and evaporation gave a crude material (2.7 g).

Purification
The crude material was recrystallised from ^{i}PrOH (ca. 23 mL) in the microwave oven, to furnishing crystalline α-anomer (1.90 g, 79%) and an anomeric mixture as white solid foam (470 mg) upon evaporation. Overall, the procedure yielded 2.37 g (98 %) of target compound **7**.

Analytics
R_f-value: 0.50 ($CHCl_3$/MeOH/H_2O = 14:6:1)

m.p.: 166.3-167.5 (^{i}PrOH)

$[\alpha]_D^{20}$: +270° (*c* 1.0, MeOH)

^{1}H NMR (400 MHz, MeOD) δ 7.45-7.33 (m, 2H, 2 × STol-H2/H6), 7.20-7.12 (m, 2H, 2 × STol-H3/H5), 5.39 (d, *J* = 1.5 Hz, 1H, H1), 4.05 (dd, *J* = 3.3, 1.5 Hz, 1H, H2), 4.03-3.95 (m, 2H, H5, H6), 3.92 (t, *J* = 9.5 Hz, 1H, H4), 3.69 (dd, *J* = 9.3, 3.3 Hz, 1H, H3), 3.48 (dd, *J* = 11.0, 7.5 Hz, 1H, H7a), 3.41 (dd, *J* = 11.0, 5.4 Hz, 1H, H7b), 2.32 (s, 3H, STol-CH_3)

^{13}C NMR (151 MHz, MeOD) δ 139.0 (STol-C2/C6), 133.6 (STol-CH), 131.7 (STol-C3/C5), 130.8 (STol-CH), 90.7 (C1), 74.1 (C5), 73.6 (C2), 73.4 (C3), 71.0 (C6), 68.0 (C4), 65.0 (C7), 21.1 (STol-CH_3)

HRMS (+ESI) m/z: calc. for $C_{14}H_{20}O_6S$ $[M+H]^+$: 317.1059, found: m/z = 317.1062

B.2.3. Synthesis of tol-4-yl 6,7-*O*-(1,1,3,3-tetraisopropyl-1,3-disiloxane-1,3-diyl)-1-thio-L-*glycero*-α-D-*manno*-heptopyranoside (3)

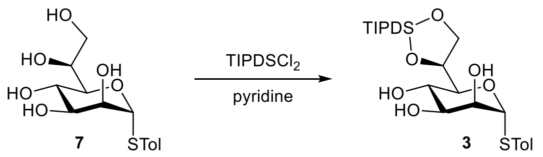

Procedure
A solution of thioglycoside **7** (1.00 g, 3.16 mmol, 1.00 eq.) in DMF (5 mL) was cooled to -60 °C after the addition of 1*H*-imidazole (538 mg, 7.90 mmol, 2.50 eq.). Then TIPDSCl_2 (997 mg, 3.16 mmol, 1.00 eq.) was added dropwise over 15 min. The reaction mixture was allowed to slowly warm to rt and was stirred over night. After TLC analysis (LP/EtOAc 1:1 and $CHCl_3$:MeOH:H_2O 14:6:1) showed complete conversion, the reaction mixture was distributed between $NaHCO_3$ and EtOAc. The aqueous layer was extracted with fresh EtOAc twice. Finally, the combined organic layers were washed with sat. $NaHCO_3$, water and brine, were dried over Na_2SO_4 and evaporated, to give the crude material.

Purification
The crude material was purified *via* column chromatography (40 g SiO_2, gradient LP/EtOAc 4:1 to 1:4) to give pure compound **3** as white solid foam (1.5 g, 85%)

Analytics
R_f-value: 0.43 (TLC, LP/EtOAc = 2:1)

$[\alpha]_D^{20}$: +150 (*c* 0.8, $CHCl_3$)

^{1}H NMR (700 MHz, $CDCl_3$) δ 7.35-7.30 (m, 2H, STol-H2/H6), 7.14-7.09 (m, 2H,STol-H3/H5), 5.49 (d, *J* = 1.1 Hz, 1H, H1), 4.23 (dt, *J* = 6.5, 3.5 Hz, 1H, H6), 4.16 (dd, *J* = 3.2, 1.1 Hz, 1H, H2), 4.09 (dd, *J* = 9.7, 2.9 Hz, 1H, H5), 3.93 (app. t, *J* = 9.5 Hz, 1H, H4), 3.83 (dd, *J* = 9.2, 3.3 Hz, 1H, H3), 3.84-3.79 (m, 2H, H7a/7b) 2.33 (s, 3H, STol-CH_3), 1.16-0.85 (m, 28H, TIPDS)

^{13}C NMR (176 MHz, $CDCl_3$) δ 138.1 (STol-C), 132.5 (STol-C2/C6), 130.0 (STol-C3/C5), 129.5 (STol-C), 88.0 (H1), 75.1 (H4), 72.7 (H3), 72.0 (H5), 71.7 (H2), 68.6 (H6), 67.4 (H7), 21.3 (STol-CH_3), 17.6, 17.6, 17.6, 17.5, 17.5, 17.4, 17.4, 17.3 (8 × TIPDS-CH_3), 13.1, 12.8, 12.5, 12.5 (4 × TIPDS-CH)

HRMS (+ESI) m/z: calc. for $C_{26}H_{46}O_7SSi_2$ $[M+Na]^+$: 581.2401, found: m/z = 581.2401

B.2.4. Synthesis of tol-4-yl 2,3,4,6,7-penta-*O*-acetyl-1-thio-D-*glycero*-α-D-*manno*-heptopyranoside (6)

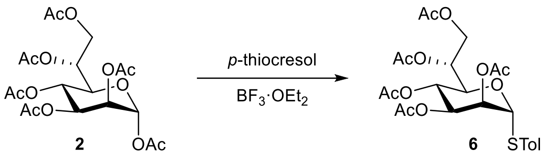

Procedure
The peracetate **2** (2.35 g, 5.08 mmol, 1.00 eq.) was dissolved in dry DCM (23.5 mL). First *p*-thiocresol (0.880 g, 7.12 mmol, 1.40 eq.) was added followed by dropwise addition of $BF_3·OEt_2$ (4.62, 10.0 mmol, 5.00 eq.) over a minute. Stirring at rt for 18 h lead to full conversion according to HPTLC (LP/EtOAc 1:1). The mixture was distributed between DCM and sat. $NaHCO_3$, the combined organic layer was washed with brine, dried over Na_2SO_4 and was evaporated to give crude material.

Purification
The crude material was purified *via* column chromatography (50 g SiO_2, 25 mL/min gradient LP/EtOAc 3:1 to 1:1) to yield target compound **6** with α:β 20:1 (2.50 g, 93.2%).

Analytics
^{1}H NMR (700 MHz, $CDCl_3$) δ 7.38-7.32 (m, 2H, STol-H2/H6), 7.14-7.08 (m, 2H, STol-H3/H5), 5.44 (dd, *J* = 3.2, 1.8 Hz, 1H, H2), 5.36 (d, *J* = 1.6 Hz, 1H, H1), 5.31 (t, *J* = 9.9 Hz, 1H, H4), 5.25 (dd, *J* = 9.7, 3.3 Hz, 1H, H3), 5.20 (dt, *J* = 6.8, 3.3, Hz, 1H, H6), 4.52 (dd, *J* = 10.0, 2.9 Hz, 1H, H5), 4.38 (dd, *J* = 12.0, 3.6 Hz, 1H, H7a), 4.19 (dd, *J* = 12.0, 7.6 Hz, 1H, H7b), 2.32 (s, 3H, STol-CH_3), 2.12, 2.10, 2.03, 2.02, 2.00 (5 × s, 5 × 3H, 5 × $COCH_3$)

^{13}C NMR (176 MHz, $CDCl_3$) δ 170.7, 169.93, 169.91 (2 ×), 169.8 (5 × $COCH_3$), 138.5 (STol-C1), 132.5 (STol-C2/C6), 130.0 (STol-C3/C5), 129.0 (STol-C4), 85.9 (C1), 70.9 (C5), 70.8 (C2), 70.1 (C6), 69.6 (C3), 66.9 (C4), 61.8 (C7), 21.2, 20.95, 20.93, 20.86, 20.83, 20.7 (5 × $COCH_3$)

B.2.5. Synthesis of tol-4-yl 1-thio-D-*glycero*-α-D-*manno*-heptopyranoside (8)

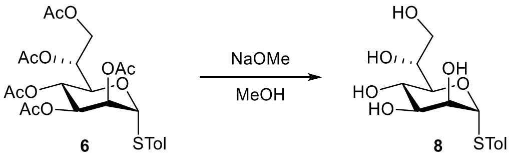

Procedure
Peracetylated thio-pyranoside **6** (2.45 g, 4.65 mmol, 1.00 eq., α:β=20:1) was dissolved in MeOH (50 mL) and NaOMe was added until pH remained basic (ca. 50 mg). An increasing amount of white precipitate was observed and the reaction mixture was stirred at rt and was monitored *via* TLC (EtOAc pure, $CHCl_3$/MeOH/H_2O 14:6:1). After 1 h the reaction was complete, the white solid was collected *via* filtration and washed with MeOH and Et_2O. The washings were combined with the mother liquor, neutralised with QP-SA and evaporated to the give the crude material (470 mg, anomeric mixture). The solid was suspended in MeOH, neutralised and evaporated to give another fraction of crude material (1.05 g only α-anomere).

Purification
The solid that was obtained from the mother liquor and washings was recrystallised from EtOH (ca. 5 mL) to give 200 mg of pure crystalline α-anomere of target compound **8**. The primary solid was recrystallised from EtOH (ca. 40 mL) to give 750 mg of crystalline α-anomere of target compound **8**. Overall, the procedure gave 950 mg (64%) of crystalline anomerically pure target compound **8**.

Analytics
R_f-value: 0.60 ($CHCl_3$/MeOH/H_2O = 14:6:1)

m.p. (EtOH): 176.9-177.8 °C

$[\alpha]_D^{20}$: +209° (*c* 0.5, MeOH)

^{1}H NMR (700 MHz, MeOD) δ 7.43-7.39 (m, 2H, STol-H2/H6), 7.17-7.13 (m, 2H, STol-H3/H5), 5.33 (dd, *J* = 1.7, 0.6 Hz, 1H, H1), 4.07-4.03 (m, 2H, H2, H5), 3.91 (ddd, *J* = 6.7, 4.3, 3.8 Hz, 1H, H6), 3.87 (app. t, *J* = 9.5 Hz, 1H, H4), 3.70 (dd, *J* = 11.6, 3.7 Hz, 1H, H7a), 3.68 (dd, *J* = 9.3, 3.3 Hz, 1H, H3), 3.57 (dd, *J* = 11.6, 6.8 Hz, 1H, H7b), 2.32 (s, 3H, STol-CH_3)

^{13}C NMR (176 MHz, MeOD) δ 139.0 (STol-C1), 133.6 (STol-C2/C6), 131.9 (STol-C4), 130.8 (STol-C3/C5), 90.7 (C1), 74.9 (C6), 74.6 (C5), 73.4 (C2), 73.1 (C3), 70.1 (C4), 64.2 (C7), 21.1 (STol-CH_3)

HRMS (+ESI) m/z: calc. for $C_{14}H_{20}O_6S$ $[M+Na]^+$: 339.0878, found: m/z = 339.0875

B.2.6. Synthesis of tol-4-yl 6,7-*O*-(1,1,3,3-tetraisopropyl-1,3-disiloxane-1,3-diyl)-1-thio-D-*glycero*-α-D-*manno*-heptopyranoside (4)

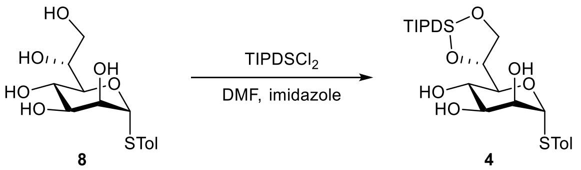

8 4

Procedure
A solution of thioglycoside **8** (1.00 g, 3.16 mmol, 1.00 eq.) in dry DMF (ca.5 mL) was cooled to -55 °C after the addition of 1*H*-imidazole (0.538 g, 7.90 mmol, 2.50 eq.). Pure $TIPDSCl_2$ (0.997 g, 3.16 mmol, 1.00 eq.) was added dropwise keeping the internal temperature below -45 °C, upon complete addition stirring was continued for 1 h. Incomplete conversion according to TLC (LP/EtOAc 1:1, $CHCl_3$:MeOH:H_2O =14:6:1) was observed. The reaction mixture was warmed to 0 °C, which drove the reaction to completion within 1 h. The reaction mixture was distributed between sat. $NaHCO_3$ and EtOAc and the aqueous layer was once reextracted with EtOAc. The combined organic layers were washed with sat. $NaHCO_3$ and brine, drying over Na_2SO_4, filtration and subsequent evaporation gave the crude material (2.20 g).

Purification
The crude material was purified *via* column chromatography (90 g SiO_2, 120 mL CV, 60 mL/min, gradient LP/EtOAc 4:1 to 1:4) to give pure target compound **4** (1.43 g, 81%).

Analytics
R_f-value: 0.26 (TLC, LP/EtOAc 1:1)

$[\alpha]_D^{20}$: +72 (*c* 1.1, $CHCl_3$).

^{1}H NMR (400 MHz, $CDCl_3$) δ 7.36 (d, *J* = 8.1 Hz, 2H, STol-H2/H6), 7.12 (d, *J* = 8.0 Hz, 2H, STol-H3/H5), 5.49 (d, *J* = 1.4 Hz, 1H, H1), 4.23 – 4.17 (m, 1H, H2), 4.14 (td, *J* = 8.6, 7.3, 1.7 Hz, 1H, H6), 4.09 (s, 1H, 2-OH), 4.03 – 3.92 (m, 1H, H4), 3.90 (dd, *J* = 8.6, 2.8 Hz, 2H, H3), 3.85 (t, *J* = 8.5 Hz, 1H, H5), 3.80 (dd, *J* = 12.5, 1.7 Hz, 1H, H7a), 3.59 (dd, *J* = 12.5, 7.2 Hz, 1H, H7b), 2.98 (s, 1H, 3-OH), 2.76 (s, 1H, 4-OH), 2.33 (s, 3H, STol-CH_3),1.44 – 0.55 (m, 28H, TIPDS-H)

^{13}C-NMR (101 MHz, $CDCl_3$) δ = 138.0 (Stol-C1), 132.4 (Stol-C2/C6), 130.1 (Stol-C3/C5), 129.7 (Stol-C4), 87.6 (C1), 79.9 (C6), 72.0 (C3), 71.7 (C4), 71.3 (C2), 69.8 (C5), 67.1 (C7), 21.3 (STol-CH_3), 17.5, 17.45, 17.42, 17.39, 17.30, 17.27 (8 × TIPDS-CH_3), 13.3, 12.8, 12.5, 12.4 (4 × TIPDS-CH)

HRMS (+ESI) m/z: calc. for $C_{26}H_{46}O_7SSi_2$ $[M+Na]^+$: 581.2401, found: m/z = 581.2402

B.3. Decoration of endocyclic ring positions

B.3.1. Synthesis of methyl 2,3-di-*O*-acetyl-4-*O*-chloroacetyl-6,7-*O*-(1,1,3,3-tetraisopropyl-1,3-disiloxane-1,3-diyl)-L-*glycero*-α-D-*manno*-heptopyranoside (10)

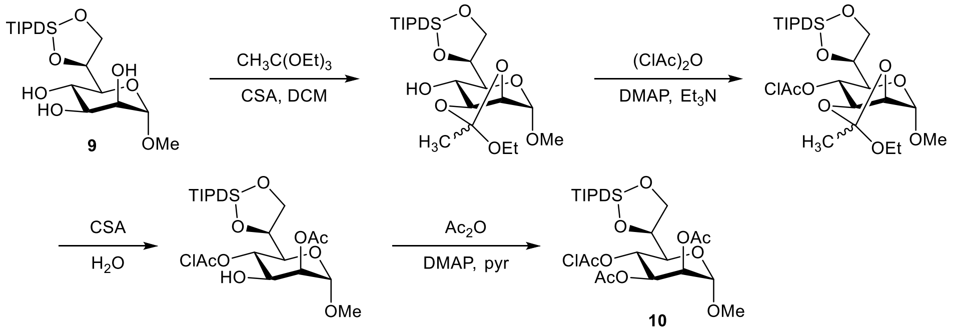

Procedure

Step 1: The starting material **10** (466,7 mg, 1.00 mmol, 1.00 eq.) was dissolved in dry DCM (9.3 mL), first ethyl orthoacetate (386.0 µL, 2.0 mmol, 2 eq.) and then CSA (23.2 mg, 0.100 mmol, 0.10 eq.) was added and the mixture was stirred under TLC (LP/EtOAc = 2:1) monitoring. After 10 min all starting material was converted to the orthoester. A small amount of Et_3N (41.9 µL, 0.30 mmol, 0.30 eq.) was added and the reaction mixture was evaporated, coevaporated from toluene and dried in HV for 30 min (leaving a white solid). **Step 2:** The reaction mixture was taken up in dry DCM (ca. 9 mL), cooled with an ice bath and first Et_3N (680 µL, 5.00 mmol, 5.00 eq.) followed by $(ClAc)_2O$ (512.9 mg, 3.00 mmol, 3.00 eq.) and then DMAP (37 mg, 0.3 mmol, 0.3 eq.) were added within a minute. Stirring was continued under ice bath cooling and then at rt. TLC monitoring (LP/EtOAc = 4:1) indicated low conversion rates so that after 60 min more $(ClAc)_2O$ (50mg, 0.29 mmol, 0.29 eq.) and DMAP (10 mg, 0.08 mmol, 0.08 eq.) were added at rt to drive the reaction to completion. Then ^{i}PrOH was added to quench excess reagent – stirring was continued for 30 min. The reaction mixture was pipetted into a stirred cold mixture of HCl (0.5 M)/Et_2O and transferred into a separatory funnel. Upon shaking of the two layers the organic layer took an orange color and had to be filtered from a bit of black solid before being washed with fresh HCl, water and $NaHCO_3$ dried over Na_2SO_4 and evaporated. **Step 3:** The residue was again taken up in DCM (ca. 10 mL) and first CSA (23 mg, 0.10 mmol, 0.10 eq.) and then water (90µL, 5.0 mmol, 5.0 eq.) was added. After 5 min TLC (LP/EtOAc = 4:1) showed complete conversion to a more polar spot. **Step 4:** After 10 more min first Ac_2O (1.0 mL, 10.5 mmol, 10.5 eq.) and pyridine (1.0 mL, 12.3 mmol, 12.3 eq.) were added simultaneously followed by DMAP (36 mg, 0.3 mmol, 0.3 eq., excess to CSA). After 2h TLC (LP/EtOAc = 4:1) indicated complete conversion to the target compound. Again ^{i}PrOH (1.2 mL, 15.7 mmol, 15.7 eq.) was added and the reaction mixture was stirred at rt for 30 min. The reaction mixture was diluted with Et_2O, and chilled HCl (0.5 M) was added, phases were separated and the organic layer was washed with fresh HCl (pH 1), water and $NaHCO_3$, filtered through a passage of cotton and evaporated to give (ca. 760 mg) of dark crude material.

Purification

The crude material was submitted to column chromatography (25 g SiO_2, 20 mL/min, gradient LP/EtOAc 9:1 – 2:1) to give 510 mg (81%) of pure material and 60 mg (9%) of less pure material.

Analytics

^{1}H-NMR (400 MHz, $CDCl_3$) δ = 5.58 (app. t, *J* = 9.9 Hz, 1H, H4), 5.35 (dd, *J* = 10.0, 3.4 Hz, 1H, H3), 5.16 (dd, *J* = 3.3, 1.8 Hz, 1H, H2), 4.68 (d, *J* = 1.7 Hz, 1H, H1), 4.17 (app. d, *J* = 8.8 Hz, 1H, H6), 4.09 (dd, *J* = 12.1, 8.7 Hz, 1H, H7a), 3.97 (s, 2H, ClAc-CH_2), 3.85 (dd, *J* = 12.1, 1.2 Hz, 1H, H7b), 3.81 (dd, *J* = 9.8, 1.5 Hz, 2H, H5), 3.36 (s, 3H, OCH_3), 2.12 (s, 3H, $COCH_3$), 1.99 (s, 3H, $COCH_3$), 1.18 – 0.92 (m, 28H, TIPDS)

^{13}C NMR (176 MHz, $CDCl_3$) δ = 170.4, 170.2 (2 × CH_3CO) 166.1 (ClAc-C=O), 98.4 (C1), 73.8 (C6), 70.8 (C5), 69.9 (C2), 69.6 (C3), 68.2 (C4), 67.6 (C7), 55.4 (OCH_3), 40.7 (ClAc-CH_2), 20.92, 20.90 (2 × $COCH_3$), 17.61, 17.59 (2 × TIPDS-CH_3), 17.57 (2 × TIPD-CH_3), 17.47, 17.45, 17.42, 17.19 (4 × TIPDS-CH_3), 13.3 (2 × TIPDS-CH), 12.81, 12.73 (2 × TIPDS-CH)

HRMS (+ESI) m/z: calc. for $C_{26}H_{48}ClO_{11}Si_2$ $[M+Na]^+$: 649.2245, found: m/z = 649.2252

B.3.2. Synthesis of methyl 2,4-di-*O*-benzoyl-3-*O*-chloroacetyl-6,7-*O*-(1,1,3,3-tetraisopropyl-1,3-disiloxane-1,3-diyl)-L-*glycero*-α-D-*manno*-heptopyranoside (11)

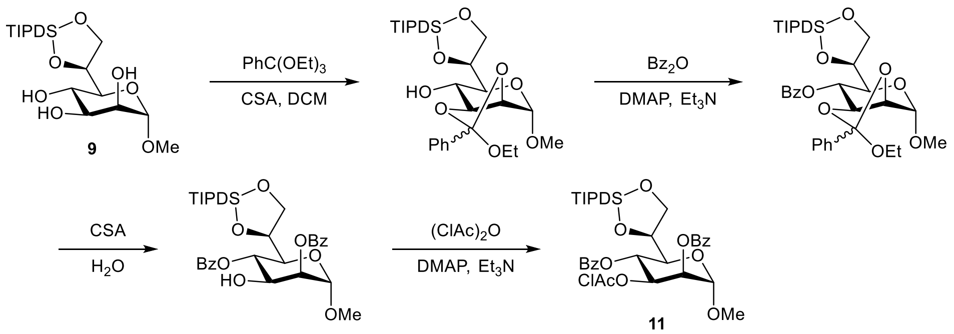

Procedure

Step 1: The starting material **9** (466.7 mg, 1.00 mmol, 1.00 eq.) was dissolved in dry DCM (9.3 mL), first ethyl orthobenzoate and then CSA (23 mg) was added and the mixture was stirred under TLC monitoring (LP/EtOAc = 1:1). After 10 min all starting material was converted to the orthoester. The reaction mixture was treated with Et_3N (40 µL, 0.3 mmol, 0.30 eq.) and was evaporated, coevaporated form toluene and shortly dried at high vacuum. **Step 2:** The reaction mixture was taken up in dry DCM (ca. 9 mL), first Et_3N (697.9 µL, 5.0 mmol, 5.0 eq.) then Bz_2O (679 mg, 3.00 mmol, 3.0 eq.) and last DMAP (33 mg, 0.3 mmol, 0.3 eq.) was added at rt. After 30 min TLC (LP/EtOAc = 4:1) indicated only little conversion, therefore Some more DMAP (35 mg, 0.28 mmol, 0.28 eq.) was added and the mixture was stirred at rt under TLC (LP/EtOAc = 9:1) monitoring. Complete conversion was observed after 20 h. The reaction mixture was cooled with an ice bath and was treated with MeOH (200 µL, 5.0 mmol, 5.00 eq.) and stirred at rt for 30 min, then it was added to a stirred mixture of Et_2O, ice and 1 M HCl. The layers were separated, and the organic layer was washed with water, $NaHCO_3$, brine, dried over Na_2SO_4 and was evaporated. **Step 3:** The residue was taken up in DCM (ca. 10 mL) and 4 drops of water and then CSA (26 mg, 0.11 mmol, 0.11 eq.) was added and the reaction mixture was stirred at rt. After 10 min TLC (LP/EtOAc = 9:1) indicated complete cleavage of the orthoester, therefore, the reaction mixture was distributed between DCM and $NaHCO_3$ and the organic layer was washed with diluted acetic acid, $NaHCO_3$ and brine. The combined organic layer was then further dried over Na_2SO_4, filtered, and evaporated. **Step 4:** The resulting residue was taken up in dry DCM (ca. 10 mL), first Et_3N (558 µL, 4.00 mmol, 4.00 eq.) then chloroacetic anhydride (342 mg, 2.00 mmol, 2.00 eq.) was added and stirred at rt for 1h. According to TLC (LP/EtOAc = 6:1) complete conversion was achieved. ^{i}PrOH (300 µL, 5.00 mmol, 5.00 eq.) was added, and was stirred for 30 min before it was diluted with Et_2O and was worked up the same way as the benzoylation before.

Purification

Crude material was submitted to column chromatography (40 g SiO_2, 30 mL/min, gradient LP/EtOAc 20:1 – 4:1) to yield 606 mg (81%) of pure target compound **11** as solid foam.

Analytics

^{1}H NMR (400 MHz, $CDCl_3$) δ = 8.16 - 8.08, 8.02 - 7.93 (2 × m, 2 × 2H, 2 × Bz-H2/H6), 7.69 - 7.59, 7.59 - 7.54 (2 × m, 2 × 1H, Bz-H4), 7.52 - 7.40 (m, 4H, 2 × Bz-H3/H5), 5.95 (t, J = 10.0 Hz, 1H, H4), 5.63 (dd, J = 10.1, 3.4 Hz, 1H, H3), 5.52 (dd, J = 3.4, 1.8 Hz, 1H, H2), 4.88 (d, J = 1.5 Hz, 1H, H1), 4.33-4.27 (m, 1H, H6), 4.15 (dd, J = 12.2, 8.7 Hz, 1H, H7a), 4.05 (dd, J = 9.9, 1.7 Hz, 1H, H5), 3.93

(dd, *J* = 12.1, 1.2 Hz, 1H, H7b), 3.89 (d, *J* = 15.2 Hz, 1H, ClAc-CH_2a), 3.84 (d, *J* = 15.2 Hz, 1H, ClAc-CH_2b), 3.45 (s, 3H, OCH_3), 1.18-0.75 (m, 28H, TIPDS).

^{13}C NMR (176 MHz, $CDCl_3$) δ = 166.9 (ClAc-C=O), 165.8, 165.1 (2 × Bz-C=O), 133.7, 133.5 (2 × Bz-C4), 130.2, 129.8 (2 × Bz-C2/C6), 129.5, 129.3 (2 × Bz-C1), 128.7, 128.6 (Bz-C3/C5), 98.8 (C1), 73.8 (C6), 72.2 (C3), 71.3 (C5), 70.1 (C2), 67.9 (C7), 66.5 (C4), 55.5 (OCH_3), 40.7 (ClAc-CH_2), 17.60 (2 × TIPDS-CH_3), 17.56 (2 × TIPDS-CH_3), 17.50, 17.47, 17.41, 17.14 (4 × TIPDS-CH_3), 13.3, 13.1, 12.8, 12.7 (4 × TIPDS-CH).

B.3.3. Synthesis of tol-4-yl 2,3-di-*O*-benzoyl-4-*O*-chloroacetyl-6,7-*O*-(1,1,3,3-tetraisopropyl-1,3-disiloxan-1,3-diyl)-1-thio-L-*glycero*-α-D-*manno*-heptopyranoside (12)

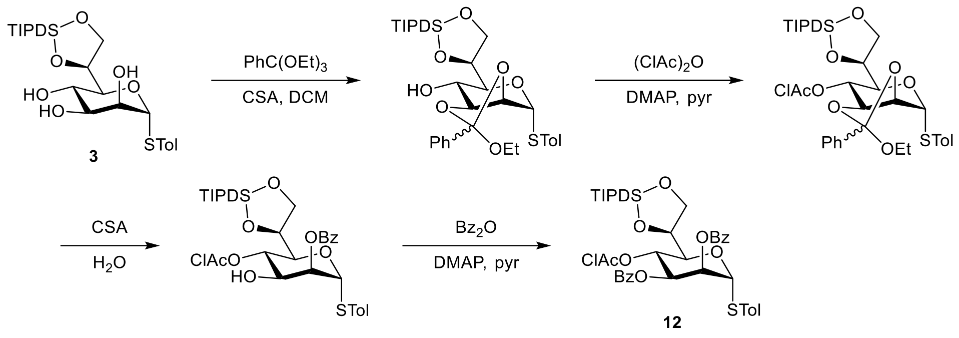

Procedure

Step 1: Starting triol **3** (563 mg, 1.01 mmol, 1.00 eq.) was dissolved in dry DCM (3 mL) before ethyl orthobenzoate (457 µL, 2.02 mmol, 2.00 eq.) and CSA (23.4 mg, 0.101 mmol, 0.100 eq.) were added and the reaction mixture was stirred at rt. Within the first 15 min TLC (LP/EtOAC = 4:1, partial hydrolysis to 2-*O*-benzoate on TLC) indicated full conversion. To quench excess of reagent, Et_3N (42 µL, 0.3 mmol, 0.30 eq.) was pipetted into the mixture and the solution was evaporated and twice coevaporated from dry toluene. **Step 2:** The colourless residue was taken up in dry DCM (1 mL) and was treated with dry pyridine (813 µL, 10.1 mmol, 10.0 eq.) before DMAP (74 mg, 0.30 mmol, 0.30 eq.) and $(ClAc)_2O$ (689 mg, 4.03 mmol, 4.00 eq.) were added. The reaction mixture was stirred at rt and was monitored *via* TLC (LP/EtOAC = 8:1) after 30 min another addition of pyridine (407 µL, 5.05 mmol, 5.00 eq.), DMAP (37 mg, 0.30 mmol, 0.3 eq.) and $(ClAc)_2O$ (344 mg, 2.02 mmol, 2.00 eq.) lead to completion of the reaction within the next 30 min. The excess of reagent was quenched by adding ^{i}PrOH (1.01 mL, 13.1 mmol, 13.0 eq.) and stirring the mixture at rt for 30 min. The mixture was diluted with Et_2O and treated with chilled 0.5 M HCl. Further, it was washed with sat. $NaHCO_3$, water and brine followed by drying over Na_2SO_4 and evaporated of solvent. **Step 3:** The residue was taken up in dry DCM (3 mL), followed by addition of water (55 µL, 3.0 mmol, 3.0 eq.) and CSA (117 mg, 0.504 mmol, 0.500 eq.). The reaction mixture was stirred at rt and TLC monitoring (LP/EtOAc = 8:1) indicated full conversion within 10 min. The solvent was evaporated and coevaporated twice from toluene. **Step 4:** The colourless residue was taken up in dry DCM (3 mL), before dry pyridine (325 µL, 4.03 mmol, 4.00 eq.), Bz_2O (455 mg, 2.02 mmol, 2.00 eq.) and DMAP (37 mg, 0.30 mmol, 0.30 eq.) were added. The reaction mixture was stirred at rt and was monitored *via* TLC (LP/EtOAc = 8:1). After 1 h approximately half of starting material was consumed, and the reaction was not proceeding further. Therefore, pyridine (162 µL, 2.02 mmol, 2.00 eq.), DMAP (37 mg, 0.30 mmol, 0.30 eq.) and Bz_2O (455 mg, 2.02 mmol, 2.00 eq.) were added and the reaction was completed within the next 1 h. The excess of reagent was quenched *via* addition of ^{i}PrOH (1.01 mL, 13.1 mmol, 13.0 eq.) and stirring at rt for 30 min. The mixture was diluted with Et_2O, treated with chilled 0.5 M HCl and was washed with sat. $NaHCO_3$ solution, water and brine. The organic phases were collected, dried over Na_2SO_4 and evaporated to yield the crude material of compound **12** (1.15 g).

Purification
The crude material was purified *via* column chromatography (40 g SiO_2, 60 mL CV, 25 mL/min, gradient LP/EtOAc 30:1 to 15:1) to give pure target compound **12** (630 mg, 75%) as colourless glass.

Analytics
R_f-value: 0.30 (LP/EtOAc = 8:1)

$[\alpha]_D^{20}$: +14.5° (*c* 1.0, $CHCl_3$)

^{1}H-NMR (400 MHz, $CDCl_3$) δ = 8.11 – 8.01 (m, 2H, Bz-H2/H6), 7.95 – 7.85 (m, 2H, Bz-H3/H5), 7.62 – 7.56 (m, 1H, Bz-H4), 7.53 – 7.48 (m, 1H, Bz-H4), 7.48 – 7.32 (m, 6H, 2 × Bz-H2/H6, STol-H2/H6), 7.15 (d, *J* = 8.0 Hz, 2H, STol-H3/H5), 6.02 (t, *J* = 10.0 Hz, 1H, H4), 5.85 (dd, *J* = 3.3, 1.5 Hz, 1H, H2), 5.70 (d, *J* = 1.4 Hz, 1H, H1), 5.65 (dd, *J* = 10.2, 3.3 Hz, 1H, H3), 4.41 (dd, *J* = 9.9, 1.8 Hz, 1H, H5), 4.26 (dt, *J* = 8.9, 1.7 Hz, 1H, H6), 4.02 – 3.90 (m, 2H, $ClCH_2$), 3.76 (dd, *J* = 12.5, 8.9 Hz, 1H, H7a), 3.54 (dd, *J* = 12.5, 1.6 Hz, 1H, H7b), 2.33 (s, 3H, STol-CH_3), 1.11 – 0.82 (m, 28H, TIPDS)

^{13}C-NMR (101 MHz, $CDCl_3$) δ = 166.8, 165.7, 165.1 (3 × C=O), 138.5 (STol-C), 133.8, 133.6 (2 × Bz-C4), 132.5 (STol-C2/C6), 130.2 (STol-C3/C5, Bz-C2/C6), 129.9 (Bz-C2/C6), 129.4, 129.2 (2 × Ar-C1), 128.6 (2 × Bz-C3/C5), 128.5 (Ar-C1), 85.6 (C1), 73.3 (C6), 72.5 (C5), 72.3 (C3), 71.3 (C2), 67.7 (C7), 66.5 (C4), 40.6 (ClAc-CH_2), 21.2 (STol-CH_3), 17.6, 17.4, 17.3, 17.2, 17.1 (8 × TIPDS-CH_3), 13.2, 12.9, 12.7, 12.5 (4 × TIPDS-CH)

B.3.4. Synthesis of tol-4-yl 2,4-di-*O*-benzoyl-3-*O*-chloroacetyl-6,7-*O*-(1,1,3,3-tetraisopropyl-1,3-disiloxane-1,3-diyl)-1-thio-L-*glycero*-α-D-*manno*-heptopyranoside (13)

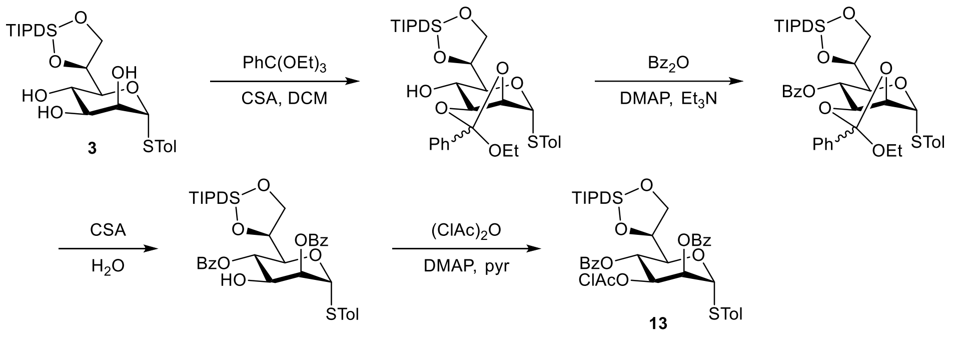

Procedure
Step 1: Triol **3** (543 mg, 0.971 mmol, 1.00 eq.) was dissolved in dry DCM (5 mL) before ethyl orthobenzoate (436 µL, 1.94 mmol, 2.00 eq.) and CSA (22.6 mg, 0.097 mmol, 0.100 eq.) were added in quick succession. The reaction showed full conversion on TLC (LP/EtOAc = 4:1, partial hydrolysis to 2-*O*-benzoate on TLC) after 15 min of stirring at rt when dry Et_3N (40.0 µL, 0.291 mmol, 0.300 eq.) was pipetted into the mixture to quench the excess of reagent and the solution was evaporated and coevaporated from dry toluene twice. **Step 2:** The colourless residue was dissolved in dry DCM (1 mL) and Et_3N (1.48 mL, 10.7 mmol, 11.0 eq.) was added, followed by the addition of Bz_2O (1.10 g, 4.86 mmol, 5.00 eq.) and DMAP (142 mg, 1.17 mmol, 1.20 eq.). The reaction mixture was stirred at rt and monitored *via* TLC (LP/EtOAc = 8:1). After 2 h, additional Bz_2O (219.7 mg, 0.971 mmol, 1 eq.), Et_3N (269 µL, 1.94 mmol, 2.00 eq.) and DMAP (35.6 mg, 0.291 mmol, 0.300 eq.) were added into the reaction mixture to drive the reaction to completeness within the next 1 h. The excess of reagents was quenched by adding ^{i}PrOH (972.8 µL, 12.6 mmol, 13.0 eq.) and stirring for 30 min at rt. The mixture was diluted with Et_2O and treated with chilled 0.5 M HCl. Further, it was washed with sat. $NaHCO_3$, water and brine followed by drying over Na_2SO_4 and evaporated of solvent. **Step 3:** The residue was taken up in dry DCM (3 mL) and first water (52.4 µL, 2.91 mmol, 1.00 eq.) and then CSA (22.6 mg, 0.097 mmol, 0.100 eq.) were added. TLC monitoring (LP/EtOAc = 8:1) indicated full conversion within 10 min of stirring at rt. The solvent was evaporated and coevaporated twice from toluene. **Step 4:** The colourless residue was dissolved in dry DCM (3 mL), dry pyridine (627 µL, 7.77 mmol, 8.00 eq.) was

pipetted in, followed by the addition of DMAP (35.6 mg, 0.291 mmol, 0.30 eq.) and $(ClAc)_2O$ (689 mg, 4.03 mmol, 4.00 eq.). The reaction was stirred at rt and monitored via TLC (LP/EtOAc = 8:1). The reaction mixture slowly turned brownish and showed full conversion within 40 min. The excess of reagent was quenched by the addition of MeOH (512 µL, 12.6 mmol, 13.0 eq.) and stirring for 30 min at rt. The mixture was diluted with Et_2O, treated with chilled 0.5 M HCl and was washed with sat. $NaHCO_3$ solution, water and brine. The organic phases were collected, dried over Na_2SO_4 and evaporated to yield the crude material (1.1 g).

Purification
The crude material was purified *via* column chromatography (40 g SiO_2, 60 mL CV, 25 mL/min, gradient LP/EtOAc 30:1 to 15:1) to give pure **13** as colourless glass (574 mg, 69%).

Analytics
R_f-value: 0.32 (TLC, LP/EtOAc = 8:1)

$[\alpha]_D^{20}$: +45° (*c* 1.1, $CHCl_3$)

^1H NMR (400 MHz, $CDCl_3$) δ = 8.16 – 8.09 (m, 2H, BzH2/H6), 8.04 – 7.98 (m, 2H, BzH2/H6), 7.65 – 7.55 (m, 2H, 2 × BzH4), 7.52 – 7.37 (m, 6H, STol-H2/H6, 2 × BzH3/H5), 7.16 (d, *J* = 7.9 Hz, 2H, STol-H3/H5), 6.02 (t, *J* = 10.0 Hz, 1H, H4), 5.79 (dd, *J* = 3.3, 1.5 Hz, 1H, H2), 5.68 (d, *J* = 1.4 Hz, 1H, H1), 5.66 (dd, *J* = 10.1, 3.3 Hz, 1H, H3), 4.47 (dd, *J* = 9.9, 1.8 Hz, 1H, H5), 4.27 (dt, *J* = 9.0, 1.7 Hz, 1H, H6), 3.96 – 3.82 (m, 2H, ClAc-CH_2), 3.78 (dd, *J* = 12.5, 8.9 Hz, 1H, H7a), 3.61 – 3.53 (m, 1H, H7b), 2.34 (s, 3H, STol-CH_3), 1.18 – 0.75 (m, 28H, TIPDS)

^{13}C NMR (101 MHz, $CDCl_3$) δ = 166.9, 165.7, 165.1 (3 × C=O), 138.5 (STol-C4), 133.8, 133.6 (2 × Bz-C4), 132.5 (STol-C2/C6), 130.2 (STol-C3/C5, Bz-C2/C6), 129.9 (Bz-C2/C6), 129.4, 129.2 (2 × Ar-C1), 128.6 (2 × Bz-C3/C5), 128.5 (Ar-C1), 85.6 (C1), 73.3 (C6), 72.5 (C5), 72.3 (C3), 71.3 (C2), 67.7 (C7), 66.5 (C4), 40.6 (ClAc-CH_2), 21.2 (STol-CH_3), 17.5, 17.4, 17.3, 17.2, 17.1 (8 × TIPDS-CH_3), 13.2, 12.9, 12.7, 12.5 (4 × TIPDS-CH)

B.3.5. Synthesis of tol-4-yl 2,3-*O*-(2-chloro-1-ethoxyethyliden-1,1-diyl)-6,7-*O*-(1,1,3,3-tetraisopropyl-1,3-disiloxane-1,3-diyl)-1-thio-L-*glycero*-α-D-*manno*-heptopyranoside . (14a), (14b)

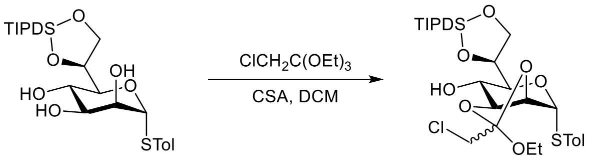

3 14a, 14b

Procedure
Starting triol **3** (29 mg, 0.052 mmol, 1.00 eq.) was dissolved in dry DCM (0.7 mL). First CSA (1 mg, 0.005 mmol. 0.1 eq.) then 1,1,1-tris(hydroxyethyl) chloroacetate (20 µL, 0.10 mmol, 2.00 eq.) was added and the mixture was stirred at rt under TLC monitoring (LP/EtOAC = 4:1). Within the first 10 min the reaction was complete, and the solvent was evaporated to give the crude material.

Purification
The crude material was purified *via* column chromatography (4 g SiO_2, 18 mL/min, gradient 15:1 – 8:1) to give pure compound **14a** (12 mg) and pure compound **14b** (10 mg), which corresponds to an overall yield of 64%

Analytics – Compound **14a**
R_f-value: 0.44 (LP/EtOAc = 4:1)

$[\alpha]_D^{20}$: +8.1° (*c* 0.4, $CHCl_3$)

^{1}H-NMR (400 MHz, $CDCl_3$) δ = 7.31 – 7.22 (m, 2H, STol-H2/H6), 7.08 – 7.02 (m, 2H, STol-H3/H5), 5.64 (d, *J* = 2.69 Hz, 1H, H1), 4.43 (dd, *J* = 6.9, 2.7 Hz, 1H, H2), 4.31 (t, *J* = 7.3 Hz, 1H, H3), 4.21 (ddd, *J* = 10.5, 7.6, 2.9 Hz, 1H, H4), 4.12 (dt, *J* = 8.5, 2.0 Hz, 1H, H6), 3.80 (dd, *J* = 10.2, 2.3 Hz, 1H, H5), 3.69 – 3.56 (m, 5H, ClAc-CH_2, OEt-CH_2, H7a), 3.53 (dd, *J* = 12.4, 1.8 Hz, 1H, H7b), 2.52 (d, *J* = 3.1 Hz, 1H, C4-OH), 2.26 (s, 3H, STol-CH3), 1.14 (t, *J* = 7.1 Hz, 3H, OEt-CH3), 1.03 – 0.80 (m, 28H, TIPDS)

^{13}C NMR (101 MHz, $CDCl_3$) δ = 138.1 (STol-C1), 132.5 (2 × STol-C2/C6), 129.9 (2 × STol-C3/C5), 128.3 (STol-C4), 120.8 (orthoester-C), 83.5 (C1), 79.7 (C3), 77.8 (C2), 74.4 (C6), 71.5 (C5), 68.4 (C4), 67.6 (C7), 59.0 (OEt-CH_2), 21.1 (STol-CH_3), 17.5, 17.4, 17.32, 17.29, 17.24, 17.21, 17.1 (8 × TIPDS-CH_3) 15.1 (OEt-CH_3), 13.0, 12.6, 12.5, 12.3 (4 × TIPDS-CH)

Analytics – Compound **14b**
R_f-value: 0.27 (LP/EtOAc = 4:1)

$[\alpha]_D^{20}$: +10.4° (*c* 0.4, $CHCl_3$)

^{1}H NMR (400 MHz, $CDCl_3$) δ = 7.37 – 7.32 (m, 2H, STol-H2/6), 7.14 – 7.07 (m, 2H, STol-H3/5), 5.71 (d, *J* = 2.8 Hz, 1H, H1), 4.51 (dd, *J* = 6.8, 2.8 Hz, 1H, H2), 4.43 (t, *J* = 7.2 Hz, 1H, H3), 4.23 – 4.15 (m, 2H, H4, H5), 3.86 (dd, *J* = 10.2, 2.4 Hz, 1H, H6), 3.77 – 3.56 (m, 6H, ClAc-CH_2, OEt-CH_2, H7a, H7b), 2.63 (d, *J* = 3.2 Hz, 1H, C4-OH), 2.33 (s, 3H, STol-CH_3), 1.22 (t, *J* = 7.0 Hz, 3H, OEt-CH_3), 1.10 – 0.94 (m, 28H, TIPDS)

^{13}C NMR (101 MHz, $CDCl_3$) δ = 138.2 (STol-C1), 132.6 (2 × STol-C2/C6), 129.9 (2 × STol-C3/C5), 128.2 (STol-C4), 120.1 (orthoester-C), 83.0 (C1), 80.3 (C3), 76.6 (C2), 74.5 (C6), 71.4 (C5), 68.6 (C4), 67.5 (C7), 59.1 (OEt-CH_2), 44.2 (ClAc-CH_2), 21.1 (STol-CH_3), 17.5, 17.4, 17.33 (2 ×), 17.29, 17.27, 17.21, 17.16 (8 × TIPDS-CH_3), 15.2 (OEt-CH_3), 13.0, 12.6, 12.3 (4 × TIPDS-CH)

B.3.6. Synthesis of tol-4-yl 4-*O*-benzoyl-2,3-*O*-(2-chloro-1-ethoxyethyliden-1,1-diyl)-6,7-*O*-(1,1,3,3-tetraisopropyl-1,3-disiloxane-1,3-diyl)-1-thio-L-*glycero*-α-D-*manno*-heptopyranoside (15a), (15b)

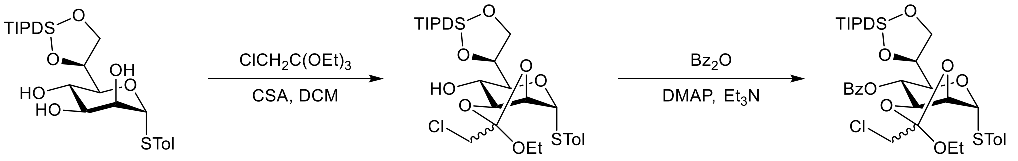

3 15a, 15b

Procedure
Step 1: Starting triol **3** (46 mg, 0.08 mmol, 1.00 eq.) was dissolved in dry DCM (2 mL). First CSA (2 mg, 0.008 mmol. 0.1 eq.) then 1,1,1-tris(hydroxyethyl) chloroacetate (34 µL, 0.16 mmol, 2.00 eq.) was added and the mixture was stirred at rt under TLC monitoring (LP/EtOAC = 4:1). Within the first 10 min the reaction was complete, and the solvent was evaporated. **Step 2:** The colourless residue was taken up in dry DCM (1 mL) and Et_3N (136 µL, 0.984 mmol, 11.0 eq.) was added together with Bz_2O (58 mg, 0.26 mmol, 3.0 eq.) and DMAP (cat. amount ca. 10 mg). The reaction mixture was stirred at rt under TLC monitoring (LP/EtOAc = 8:1). After 3 h the reaction was completed and excess reagent was quenched by adding ^{i}PrOH (87 µL, 1.2 mmol, 13 eq.) and further stirring for 30 min. Afterwards the mixture was diluted with Et_2O and extracted with chilled 0.5 M HCl. Further it was washed with sat. $NaHCO_3$, water and brine and the resulting organic layer was dried over Na_2SO_4, filtered, and evaporated to give the crude material.

Purification
The crude material was purified *via* column chromatography (4 g SiO_2, 15 mL/min, isocratic 30:1) to give pure compound **15a** (21 mg) and pure compound **15b** (14 mg), which corresponds to an overall yield of 55%

<u>Analytics – Compound **15a**</u>
Rf-value: 0.66 (LP/EtOAc = 8:1)

$[\alpha]_D^{20}$: +12° (*c* 0.6, $CHCl_3$)

^{1}H NMR (600 MHz, $CDCl_3$) δ = 8.04 (dd, *J* = 8.4, 1.4 Hz, 2H, Bz-H2/H6), 7.57 (ddt, *J* = 8.8, 7.3, 1.3 Hz, 1H, Bz-H4), 7.49 – 7.41 (m, 2H, Bz-H3/5), 7.39 – 7.33 (m, 2H, STol-H3/5), 7.18 – 7.07 (m, 2H, STol-H2/H6), 5.93 (d, *J* = 1.7 Hz, 1H, H1), 5.74 (dd, *J* = 10.3, 6.8, 1H, H4), 4.64 (dd, *J* = 7.2, 1.7 Hz, 2H, H2), 4.61 (d, *J* = 7.1, 1H, H3), 4.19 (dd, *J* = 10.3, 1.8 Hz, 1H, H5), 4.12 (dt, *J* = 8.8, 1.7 Hz, 1H, H6), 4.04 – 3.85 (m, 2H, OEt-CH_2), 3.75 – 3.66 (m, 2H, ClAc-CH_2), 3.59 (dd, *J* = 12.7, 8.9 Hz, 1H, H7a), 3.40 (dd, *J* = 12.6, 1.5 Hz, 1H, H7b), 2.33 (s, 3H, STol-CH_3), 1.28 (t, *J* = 7.0 Hz, 3H, OEt-CH_3), 1.06 – 0.73 (m, 28H, TIPDS)

^{13}C NMR (151 MHz, $CDCl_3$) δ = 164.8 (Bz-C=O), 137.9 (STol-C1), 133.2 (Bz-C4), 131.7 (STol-C3/C5), 130.0 (STol-C2/C6), 129.8 (Bz-C2/C6), 129.7 (Ar-C), 128.4 (Bz-C3/C5), 128.4 (Ar-C), 120.9 (orthoester-C), 83.7 (C1), 77.6 (C2/C3), 77.4 (C2/C3), 73.6 (C6), 70.0 (C5), 69.6 (C4), 67.9 (C7), 58.8 (OEt-CH_2), 46.5 (ClAc-CH_2), 21.1 (STol-CH_3), 17.4 17.3, 17.2, 17.1, 17.0 (8 × TIPDS-CH_3), 14.9 (OEt-CH_3), 13.0, 12.8, 12.5, 12.4 (4 × TIPDS-CH)

<u>Analytics – Compound **15b**</u>
Rf-value: 0.57 (LP/EtOAc = 8:1).

$[\alpha]_D^{20}$: +10° (*c* 0.5, $CHCl_3$)

^{1}H NMR (600 MHz, $CDCl_3$) δ = 8.07 – 8.01 (m, 2H, BzH2/H6), 7.57 (t, *J* = 7.5 Hz, 1H, BzH4), 7.48 – 7.42 (m, 2H, BzH3/H5), 7.37 – 7.32 (m, 2H, STol-H2/H6), 7.19 – 7.11 (m, 2H, STol-H3/H5), 5.98 – 5.94 (m, 1H, H1), 5.57 (dd, *J* = 10.0, 7.3 Hz, 1H, H4), 4.65 (dd, *J* = 7.3, 6.1 Hz, 1H, H3), 4.61 (dd, *J* = 6.2, 1.2 Hz, 1H, H2), 4.18 (dd, *J* = 10.0, 1.9 Hz, 1H, H5), 4.12 (dt, *J* = 9.1, 1.7 Hz, 1H, H6), 3.89 – 3.78 (m, 2H, ClAc-CH_2), 3.67 – 3.60 (m, 2H, OEt-CH_2), 3.56 (dd, *J* = 12.6, 9.0 Hz, 1H, H7a), 3.35 (dd, *J* = 12.6, 1.6 Hz, 1H, H7b), 2.33 (s, 3H, STol-CH_3), 1.24 – 1.18 (m, 3H, OEt-CH_3), 1.05 – 0.80 (m, 28H, TIPDS)

^{13}C NMR (151 MHz, $CDCl_3$) δ = 164.8 (C=O), 138.0 (STol-C1), 133.2 (Bz-C4), 131.9 (2 × STol-C3/C5), 130.0 (2 × STol-C2/C6), 129.8 (2 × Bz-C2/C6), 129.7 (2 × Ar-C), 128.4 (2 × Bz-C3/C5), 128.2 (Ar-C), 120.1 (orthoester-C), 83.0 (C1), 78.1 (C3), 76.9 (C2), 73.4 (C6), 69.6 (C5), 69.1 (C4), 67.5 (C7), 58.8 (OEt-CH_2), 45.0 (ClAc-CH_2), 21.1 (STol-CH_3), 17.4, 17.3, 17.2, 17.1, 17.0 (8 × TIPDS-CH_3), 15.1 (OEt-CH_3), 13.1, 13.0, 12.5, 12.4 (4 × TIPDS-CH)

B.3.7. Synthesis of tol-4-yl 2,3-di-*O*-benzoyl-4-*O*-chloroacetyl-6,7-*O*-(1,1,3,3-tetraisopropyl-1,3-disiloxan-1,3-diyl)-1-thio-D-*glycero*-α-D-*manno*-heptopyranoside (16)

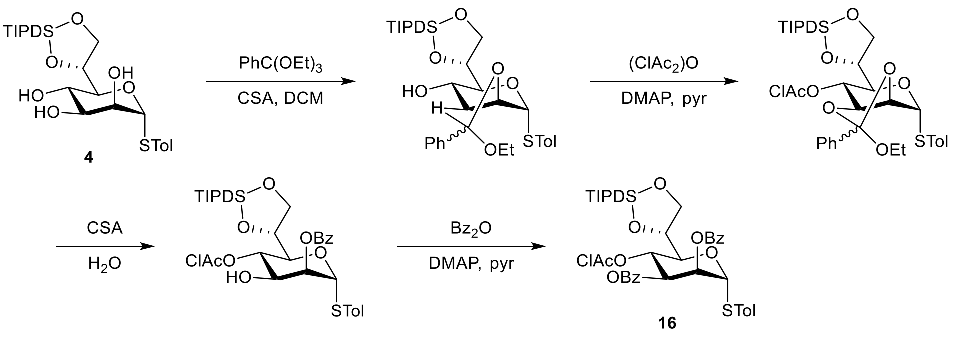

<u>Procedure</u>
Step 1: Starting triol **4** (565 mg, 1.01 mmol, 1.00 eq.) was dissolved in dry DCM (6 mL) before ethyl orthobenzoate (457 µL, 2.02 mmol, 2.00 eq.) and CSA (23.4 mg, 0.101 mmol, 0.100 eq.) were added and the reaction mixture was stirred at rt. Within the first 15 min TLC (LP/EtOAC = 4:1, partial

hydrolysis to 2-*O*-benzoate on TLC) indicated full conversion. To quench excess of reagent, Et_3N (42 µL, 0.3 mmol, 0.30 eq.) was pipetted into the mixture and the solution was evaporated and twice coevaporated from dry toluene. **Step 2:** The colourless residue was taken up in dry DCM (6 mL) and was treated with dry pyridine (816 µL, 10.1 mmol, 10.0 eq.) before DMAP (74 mg, 0.30 mmol, 0.30 eq.) and $(ClAc)_2O$ (689 mg, 4.03 mmol, 4.00 eq.) were added. The reaction mixture was stirred at rt and was monitored *via* TLC (LP/EtOAC = 8:1) after 30 min another addition of DMAP (74 mg, 0.60 mmol, 0.60 eq.) and $(ClAc)_2O$ (691 mg, 4.04 mmol, 4.00 eq.) led to completion of the reaction within the next 2 h. The excess of reagent was quenched by adding ^{i}PrOH (1.01 mL, 13.1 mmol, 13.0 eq.) and stirring the mixture at rt for 30 min. The mixture was diluted with Et_2O and treated with chilled 0.5 M HCl. Further, it was washed with sat. $NaHCO_3$, water and brine followed by drying over Na_2SO_4 and evaporated of solvent. **Step 3:** The residue was taken up in dry DCM (6 mL) then water (55 µL, 3.03 mmol, 3.00 eq.) and CSA (117 mg, 0.505 mmol, 0.500 eq.) was added and the reaction mixture was stirred at rt. Within the first 15 min full conversion was achieved according to TLC (LP/EtOAc = 4:1). **Step 4** The solvent was evaporated, and the reaction mixture was again taken up in dry DCM (6 mL). First pyridine (326 µL, 4.04 mmol, 4.00 eq.) was added, followed by DMAP (74 mg, 0.61 mmol, 0.60 eq.) and portion wise addition of Bz_2O (457 mg, 2.02 mmol, 2.00 eq.) within 1 min. The reaction mixture was stirred at rt and monitored *via* TLC (LP/EtOAc = 8:1) and the reaction was completed within 2 h. The excess of reagent was quenched with addition of MeOH (324 µL, 10.0 mmol, 10.0 eq.) and stirring at rt for 1 h. The mixture was diluted with Et_2O, treated with chilled 0.5 M HCl, and was washed with sat. $NaHCO_3$ solution, water, and brine. The organic phases were collected, dried over Na_2SO_4, and evaporated to yield the crude material of compound **16**.

<u>Purification</u>
The crude material was purified *via* column chromatography (90 g SiO_2, 60 mL/min, gradient LP/EtOAc 20:1 to 4:1) to give mostly pure (>90%) target compound **16** (506 mg, 60%) as colourless glass.

<u>Analytics</u>
R_f-value: 0.45 (LP/EtOAc = 4:1)

$[\alpha]_D^{20}$: +11° (*c* 0.5, MeOH)

^{1}H NMR (400 MHz, $CDCl_3$) δ = 8.03 (d, 2H, Bz-H2/H6), 7.89 (d, 2H, Bz-H2/H6), 7.59 (t, 1H, Bz-H4), 7.51 (t, 1H, Bz-H4), 7.48 – 7.39 (m, 4H, Bz-H3/H5, STol-H2/H6), 7.36 (t, *J* = 7.7 Hz, 2H, Bz-H3/H5), 7.14 (d, *J* = 7.9 Hz, 2H, STol-H3/H5), 6.00 (t, *J* = 9.9 Hz, 1H, H4), 5.84 (dd, *J* = 3.2, 1.7 Hz, 1H, H2), 5.61 (dd, *J* = 9.9, 3.2 Hz, 1H, H3), 5.58 (d, *J* = 1.6 Hz, 1H, H1), 4.55 (dd, *J* = 10.0, 1.8 Hz, 1H, H5), 4.18 (d, *J* = 9.0, 1H, H6), 4.00 (dd, *J* = 12.1, 1.4 Hz, 1H, H7a), 3.94 (s, 2H, ClAc-CH_2), 3.79 (dd, *J* = 12.1, 8.8 Hz, 1H, H7b), 2.34 (s, 3H, STol-CH_3), 1.18 – 0.89 (m, 28H, TIPDS)

^{13}C NMR (101 MHz, $CDCl_3$) δ = 166.0, 165.6, 165.4 (3 × C=O), 138.5 (STol-C), 133.7, 133.6 (2 × Bz-C4), 132.7 (STol-C2/C6), 130.1 (STol-C3/C5), 130.0 (Bz-C2/C6), 129.4, 129.3 (2 × Bz-C1), 128.9 (STol-C), 128.7 (Bz-C3/C5), 86.3 (C1), 76.8 (C6), 72.4 (C5), 72.0 (C2), 71.1 (C3), 68.3 (C4), 67.7 (C7), 40.6 (ClAc-CH_2), 21.3 (STol-CH_3), 17.52, 17.46, 17.43, 17.39 (8 × TIPDS-CH_3), 12.98, 12.94, 12.5, 12.4 (4 × TIPDS-CH)

B.3.8. Synthesis of tol-4-yl 3-*O*-benzoyl-6,7-*O*-(1,1,3,3-tetraisopropyl-1,3-disiloxan-1,3-diyl)-1-thio-L-*glycero*-α-D-*manno*-heptopyranoside (18)

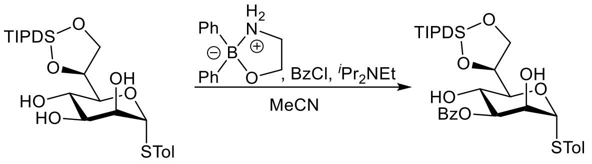

3 18

Procedure

First diphenyl borinate (24 mg, 0.11 mmol, 0.33 eq.) was transferred into a round bottom flask and was placed under an argon atmosphere. The starting sugar triole **3** (187 mg, 0.335 mmol, 1.00 eq.) was dissolved in dry MeCN (2 mL) and was added to the borinate. Next ^{i}Pr$_2$NEt (62 µL, 0.356, 1.06 eq.) followed by BzCl (42 µL, 0.361 mmol, 1.08 mmol) was added and the mixture was stirred at rt and was monitored *via* TLC (LP/EtOAc = 5:1). After 1 h another addition of ^{i}Pr$_2$NEt (17 µL, 0.10 mmol, 0.30 eq.) and BzCl (11 µl, 0.10 mmol, 0.30 eq.) was added and the reaction was stirred for 1 h. Then TLC indicated good conversion but still some starting material was present, therefore a last addition of ^{i}Pr$_2$NEt (26 µL, 0.15 mmol, 0.45 eq.) and benzoylchloride (17 µL, 0.15 mmol, 0.44 eq.) was conducted, to finally achieve full conversion after overall 3.5 h reaction time. The reaction mixture was distributed between Et_2O and water. The aqueous phase was extracted with EtOAc and then the combined organic layers were washed with brine (reextracted with EtOAc and recombined), dried over $MgSO_4$ and evaporated to give crude the material (279 mg).

Purification

The crude material was submitted to column chromatography (10 g SiO_2, 12 mL/min, gradient LP/EtOAc 15:1 – 2:1) to give pure target compound **18** (147 mg, 66%) as viscous light-yellow liquid.

Analytics

R_f-value: 0.56 (LP/EtOAc = 5:1)

$[\alpha]_D^{20}$: +120° (*c* 1.0, $CHCl_3$)

^{1}H NMR (400 MHz, $CDCl_3$) δ = 8.12 (d, *J* = 7.2 Hz, 2H, Bz-H2/H6), 7.60 (t, *J* = 7.4 Hz, 1H, Bz-H4), 7.47 (t, *J* = 7.7 Hz, 2H, Bz-H3/H5), 7.35 (d, *J* = 8.1 Hz, 2H, STol-H2/H6), 7.13 (d, *J* = 8.0 Hz, 2H, STol-H3/H5), 5.53 – 5.49 (m, 1H, H1), 5.33 (dd, *J* = 9.6, 3.0 Hz, 1H, H3), 4.53 – 4.25 (m, 3H, H2, H4, H6), 4.20 (dd, *J* = 9.6, 2.2 Hz, 1H, H5), 3.94 – 3.65 (m, 2H, H7a, H7b), 2.89 (s, 1H, OH), 2.34 (s, 3H, STol-CH_3), 2.28 (s, 1H, OH), 1.20 – 0.86 (m, 28H, TIPDS)

^{13}C NMR (101 MHz, $CDCl_3$) δ = 166.8 (C=O), 138.2 (STol-C1), 133.7 (Bz-C4), 132.4 (STol-C2/C6), 130.11 (STol-C3/C5), 130.06 (Bz-C2/C6), 129.6, 129.4 (2 × Ar-C), 128.6 (Bz-C3/C5), 88.0 (C1), 75.8 (C3), 74.5 (C2/C6), 73.4 (C5), 70.7 (C2/C6), 67.7 (C7), 66.1 (C4), 21.3 (STol-CH_3), 17.61, 17.58, 17.51, 17.46, 17.39, 17.33 (8 × TIPDS-CH_3), 13.1, 12.8, 12.55, 12.49 (4 × TIPDS-CH)

B.3.9. Synthesis of tol-4-yl 3-*O*-benzoyl-2-*O*-chloroacetyl-6,7-*O*-(1,1,3,3-tetraisopropyl-1,3-disiloxan-1,3-diyl)-1-thio-L-*glycero*-α-D-*manno*-heptopyranoside (19)

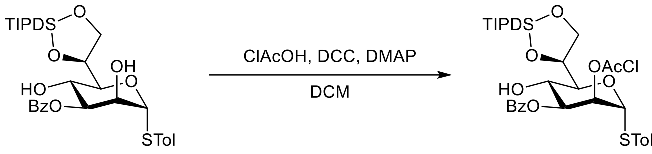

18 **19**

Procedure

Starting material **18** (183 mg, 0.276 mmol, 1.00 eq.) was dissolved in dry DCM (2 mL). Next DMAP (ca. 5 mg) was added followed by a 100 mg/mL solution of chloroacetic acid (250 µL, 0.286 mmol, 1.04 eq.) in dry DCM. Afterwards a 200 mg/mL solution of DCC in dry DCM (290 µL, 0.28 mmol, 1.0. eq.) was added in 4 equal portions within 30 min. The reaction was stirred at rt and was monitored *via* TLC (LP/EtOAc = 7:1). After 2 h another addition of DCC-solution (73 µL,0.072 mmol, 0.25 eq.) was performed, as the reaction proceeded slowly. Further stirring for 1 h made the reaction proceed to about half conversion, therefore again DCC solution (73 µL, 0.072 mmol, 0.25 eq.) and chloroacetic acid solution (125 µL, 0.143 mmol, 0.52 eq.) was added to the reaction mixture. After overall 4.5 h of reaction time the amount of di-chloroacetate by-product became significant, therefore the reaction was diluted with DCM (30 mL) and was filtered through a cotton plug. The solution was then washed with H_2O, $NaHCO_3$, and brine and all organic layers were re-extracted with 5 mL of DCM. The combined organic layer was dried over Na_2SO_4 and was stripped of solvent under reduced pressure. To give the crude material that was analysed by NMR to be a 4:1 mixture of target compound and 2,4-*O*-dichloroacetylated by-product.

Purification

The crude material was submitted to column chromatography (45 g SiO_2, CV=70 mL, 30 ml/min, gradient LP/EtOAc 30:1 – 3:1) to give pure target compound **19** (93 mg, 46%) and 40 mg as 3:1 mixture of **19** and di-chloroacetylated by-product. Also, minor amounts of the by-product were isolated in pure form and were characterized.

Analytics – **19**

R_f-value: 0.46 (LP/EtOAc = 7:1)

$[\alpha]_D^{20}$: +66° (*c* 1.0, $CHCl_3$)

^{1}H NMR (400 MHz, $CDCl_3$) δ = 8.03 (d, *J* = 7.2 Hz, 2H, Bz-H2/H6), 7.59 (t, *J* = 7.4 Hz, 1H, Bz-H4), 7.46 (t, *J* = 7.7 Hz, 2H, Bz-H3/H5), 7.36 (d, *J* = 8.1 Hz, 2H, STol-H2/H6), 7.14 (d, *J* = 7.9 Hz, 2H, STol-H3/H5), 5.63 (dd, *J* = 3.0, 1.4 Hz, 1H, H2), 5.51 – 5.44 (m, 2H, H1, H3), 4.40 – 4.29 (m, 2H, H6, H4), 4.19 (dd, *J* = 9.6, 1.9 Hz, 1H, H5), 4.13 – 4.03 (m, 2H, ClAc-CH_2), 3.84 (dd, *J* = 12.2, 8.7 Hz, 1H, H7a), 3.80 – 3.71 (m, 1H, H7b), 2.81 (d, *J* = 3.8 Hz, 1H, OH), 2.34 (s, 3H, STol-CH_3), 1.13 – 0.93 (m, 28H, TIPDS)

^{13}C NMR (101 MHz, $CDCl_3$) δ = 166.54, 166.49 (2 × C=O), 138.6 (STol-C1), 133.7 (Bz-C4), 132.9 (STol-C2/C6), 130.1 (STol-C3/C5), 130.1 (Bz-C2/C6), 129.3, 128.8 (2 × Ar-C), 128.7 (Bz-C3/C5), 85.7 (C1), 74.1 (C6), 73.6 (C5), 73.19 (C3), 73.16 (C2), 67.7 (C7), 66.2 (C4), 40.6 (ClAc-CH_2), 21.3 (STol-CH_3), 17.62, 17.62, 17.58, 17.52, 17.46, 17.45, 17.39, 17.33 (8 × TIPDS-CH_3), 13.2, 12.75, 12.74, 12.5 (4 × TIPDS-CH)

Analytics – (byproduct)

R_f-value: 0.54 (LP/EtOAc = 7:1)

$[\alpha]_D^{20}$: +42° (*c* 1.0, $CHCl_3$)

^{1}H NMR (400 MHz, $CDCl_3$) δ = 7.96 (d, *J* = 7.4 Hz, 2H, Bz-H2/H6), 7.58 (t, *J* = 7.4 Hz, 1H, Bz-H4), 7.44 (t, *J* = 7.7 Hz, 2H, Bz-H3/H5), 7.36 (d, *J* = 8.1 Hz, 2H, STol-C2/C6), 7.15 (d, *J* = 7.9 Hz, 2H, STol-C3/C5), 5.85 (t, *J* = 10.0 Hz, 1H, H4), 5.69 (dd, *J* = 3.0, 1.4 Hz, 2H, H2), 5.59 – 5.52 (m, 2H, H3, H1), 4.38 – 4.30 (m, 1H, H5), 4.21 (d, *J* = 8.9 Hz, 1H, H6), 4.13 – 4.02, 4.00 – 3.90 (2 × m, 2 × 2H, 2 × ClAc-CH_2), 3.77 (dd, *J* = 12.3, 9.0 Hz, 1H, H7a), 3.61 – 3.53 (m, 1H, H7b), 2.34 (s, 3H, STol-CH_3), 1.13 – 0.90 (m, 28H, TIPDS)

^{13}C NMR (101 MHz, $CDCl_3$) δ 166.5, 166.1, 165.6 (3 × C=O), 138.7 (STol-C1), 133.8 (Bz-C4), 132.7 (STol-C2/C6), 130.2 (STol-C3/C5), 130.0 (Bz-C2/C6), 128.8 (Ar-C), 128.7 (Bz-C3/C5), 128.4 (Ar-C), 85.2 (C1), 73.4 (C6), 73.0 (C2), 71.9 (C5), 70.8 (C3), 67.7 (C4), 67.4 (C7), 40.6, 40.5 (2 × ClAc-CH2), 21.3 (STol-CH_3), 17.64, 17.61, 17.56, 17.54, 17.44, 17.41, 17.3, 17.2 (8 × TIPDS-CH_3), 13.4, 13.2, 12.7, 12.6 (4 × TIPDS-CH)

B.3.10. Synthesis of tol-4-yl 3,4-di-*O*-benzoyl-2-*O*-chloroacetyl-6,7-*O*-(1,1,3,3-tetraisopropyl-1,3-disiloxan-1,3-diyl)-1-thio-L-*glycero*-α-D-*manno*-heptopyranoside (17)

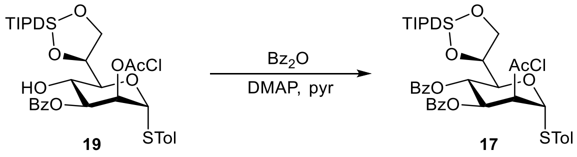

Procedure

Starting material **19** (39 mg, 0.053 mmol, 1.00 eq.) was dissolved in dry DCM (0.5 mL). Then pyridine (43 µL, 0.53 mmol, 10.0 eq.), Bz_2O (70 mg, 0.31 mmol, 5.9 eq.) and DMAP (spatula tip) was added to the solution. The reaction mixture was stirred at rt for 15 h, showing complete conversion according to TLC analysis (LP/EtOAc = 10:1) afterwards. The excess of reagent was quenched by adding THF (0.5 mL) and water (18 µL, 1.0 mmol, 18 eq.) for 6 h. Next, the mixture was diluted with Et_2O and was washed with chilled 1 N HCl. The aqueous layer was reextracted with Et_2O and the combined organic layer was then washed with sat. $NaHCO_3$ and brine. Drying over $MgSO_4$ and evaporation of solvent gave the crude material (31 mg), that already indicated decomposition of material upon NMR analysis.

Purification

The crude material was purified *via* column chromatography (4 g SiO_2, CV=7 mL, 7 mL/min, gradient LP/EtOAc 30:1 – 3:1) to give target compound **17** (12 mg, 27%) but mostly unwanted 2-OH by-product.

Analytics

R_f: 0.44 (LP/EtOAc = 5:1)

^{1}H NMR (600 MHz, $CDCl_3$) δ 7.97 – 7.91 (m, 4H, 2 × Bz-H2/H6), 7.56 – 7.48 (m, 2H, 2 × Bz-H4), 7.41 – 7.35 (m, 6H, 2 × Bz-H3/H5, STol-H2/H6), 7.16 (d, *J* = 7.9 Hz, 2H, STol-H3/H5), 6.06 (t, *J* = 10.0 Hz, 1H, H4), 5.74 (dd, *J* = 3.1, 1.6 Hz, 1H, H2), 5.67 (dd, *J* = 10.1, 3.2 Hz, 1H, H3), 5.59 (d, *J* = 1.4 Hz, 1H, H1), 4.45 (dd, *J* = 9.8, 1.4 Hz, 1H, H5), 4.26 (d, *J* = 9.0 Hz, 1H, H6), 4.13 (d, *J* = 15.0 Hz, 1H, ClAc-CH_2a), 4.08 (d, *J* = 15.0 Hz, 1H, ClAc-CH_2b), 3.81 (dd, *J* = 12.4, 9.0 Hz, 1H, H7a), 3.61 (dd, *J* = 12.4, 1.5 Hz, 1H, H7b), 2.35 (s, 3H, STol-CH_3), 1.16 – 0.84 (m, 28H, TIPDS)

13C NMR (151 MHz, $CDCl_3$) δ 166.6, 165.7, 165.2 (3 × C=O), 133.5, 133.4 (2 × Bz-C4), 132.7 (STol-C2/C6), 130.7 (2 × Ar-C), 130.0, 129.8 (2 × Bz-C2/C6), 129.0 (STol-C2/C6), 128.6, 128.5 (2 × Bz-C3/C5), 85.4 (C1), 73.4 (C6), 73.2 (C2), 72.4 (C5), 71.1 (C3), 67.6 (C7), 66.2 (C4), 40.6 (ClAc-CH_2), 21.3 (STol-CH_3), 17.56, 17.55, 17.53, 17.434, 17.431, 17.28, 17.23 (8 × TIPDS-CH_3), 13.3, 13.1, 12.7, 12.6 (4 × TIPDS-CH)

B.3.11. Synthesis of tol-4-yl 2,3,4-tri-*O*-benzoyl-6,7-*O*-(1,1,3,3-tetraisopropyl-1,3-disiloxane-1,3-diyl)-1-thio-L-*glycero*-α-D-*manno*-heptopyranoside (21)

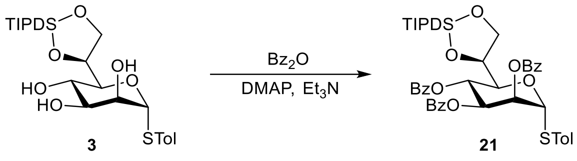

Procedure

Starting triol **3** (388 mg, 0.694 mmol, 1.00 eq.) was dissolved in DCM (5 mL) before first Et_3N (1.0 mL, 7.2 mmol, 10 eq.) and next Bz_2O (945 mg, 4.18 mmol, 6.00 eq.) was added within 5 min followed by catalytic amounts of DMAP (ca. 50 mg). The reaction mixture was stirred at rt under TLC monitoring (LP/EtOAc = 20:1). After 22 h full conversion to a new apolar spot was observed, THF (4.2 mL) and water (75 µL, 4.2 mmol, 6.0 eq.) were added to quench excess of reagent by stirring at rt for further 30 min. The reaction mixture was diluted with Et_2O and washed with 2 N HCl, sat. $NaHCO_3$, and water. The combined organic layer was dried over Na_2SO_4, filtered and was evaporated to give the crude product (590 mg) as sticky solid.

Purification

The crude material was purified *via* column chromatography (45 g SiO_2, CV = 60 mL, 60 mL/min, isocratic LP/EtOAC = 15:1) to give pure target compound **21** (493 mg, 81%) as colourless glass.

Analytics

R_f-value: 0.27 (LP/EtOAc = 8:1)

$[\alpha]_D^{20}$: +5.7° (*c* 1.0, $CHCl_3$)

^{1}H-NMR (600 MHz, $CDCl_3$) δ = 8.12 – 8.08 (m, 2H, Bz-H2/H6), 7.98 – 7.94, 7.89 – 7.85 (2 × m, 2 × 2H, 2 × Bz-H2/H6), 7.61 – 7.57, 7.53 – 7.49 (2 × m, 2 × 1H, 2 × Bz-H4), 7.47 – 7.36 (m, 7H, Bz-H4, 2 × Bz-H3/H5, STol-H2/H6), 7.30 – 7.26 (m, 2H, Bz-H3/H5), 7.16 (d, *J* = 7.9 Hz, 2H, STol-H3/H5), 6.22 (t, *J* = 10.1 Hz, 1H, H4), 5.90 (dd, *J* = 3.3, 1.5 Hz, 1H, H2), 5.79 – 5.73 (m, 2H, H1, H3), 4.53 (dd, *J* = 9.9, 1.8 Hz, 1H, H5), 4.31 (dt, *J* = 9.0, 1.7 Hz, 1H, H6), 3.80 (dd, *J* = 12.5, 9.0 Hz, 1H, H7a), 3.59 (dd, *J* = 12.5, 1.6 Hz, 1H, H7b), 2.34 (s, 3H, STol-CH_3), 1.64 – 0.73 (m, 28H, TIPDS)

^{13}C NMR (101 MHz, $CDCl_3$) δ = 165.7, 165.6, 165.2 (3 × C=O), 138.3 (Ar-C), 133.6 (Bz-C4), 133.3, 133.3 (2 × Bz-C4), 132.5 (2 × STol-C), 130.1 (2 × STol-C, 2 × Bz-C2/C6), 130.0, 129.8 (4 × Bz-C2/C6), 129.6, 129.4, 129.2, 128.9 (4 × Ar-C1), 128.6, 128.5, 128.5 (6 × Bz-C3/C5), 85.7 (C1), 73.4 (C6), 72.5 (C5), 71.9 (C2), 71.5 (C3), 67.8 (C7), 66.5 (C4), 21.3 (STol-CH_3), 17.6, 17.5,17.4, 17.3, 17.2 (8 × TIPDS-CH_3), 13.3, 13.1, 12.7, 12.5 (4 × TIPDS-CH)

B.3.12. Synthesis of tol-4-yl 2,3,4-tri-*O*-benzoyl-6,7-*O*-(1,1,3,3-tetraisopropyl-1,3-disiloxane-1,3-diyl)-1-thio-D-*glycero*-α-D-*manno*-heptopyranoside (29)

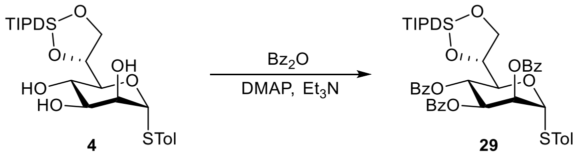

Procedure

Starting triol **4** (100 mg, 0.179 mmol, 1.00 eq.) was dissolved in dry DCM (ca. 1 mL) before first Et_3N (248 µL, 1.79 mmol, 10.0 eq.) and then Bz_2O (243 mg, 1.07 mmol, 6.00 eq.) was added slowly and the mixture was stirred at rt. Reaction monitoring *via* TLC (LP/EtOAc = 7:1) indicated incomplete conversion after 2 h, therefore DMAP (13 mg, 0.11 mmol, 0.60 eq.) was added. The reaction was completed after 2 h. To quench the excess of reagent water (20 µL, 1.1 mmol, 6.00 eq.) and THF (ca. 3

mL) was added and the mixture was stirred for further 30 min. The reaction mixture was diluted with Et_2O and washed with 2 N HCl, sat. $NaHCO_3$, and water. The combined organic layer was dried over Na_2SO_4, filtered and was evaporated to give the crude product as sticky solid.

Purification
The crude material was purified *via* column chromatography (9 g SiO_2, gradient LP/EtOAC = 20:1 – 4:1). To yield pure target compound **29** (107 mg, 69%) as colourless glass.

Analytics
R_f-value 0.32 (LP/EtOAc = 4:1)

$[\alpha]_D^{20}$: -2.0° (*c* 1.0, $CHCl_3$)

^{1}H NMR (400 MHz, $CDCl_3$) δ = 8.07 (dd, *J* = 8.3, 1.2 Hz, 2H, Bz-H2/H6), 7.98 (dd, *J* = 8.4, 1.3 Hz, 2H, Bz-H2/H6), 7.86 (dd, *J* = 8.3, 1.2 Hz, 2H, Bz-H2/H6), 7.64 – 7.55 (m, 1H, Bz-H4), 7.57 – 7.48 (m, 1H, Bz-H4), 7.51 – 7.35 (m, 8H, Bz-H4, STol-H2/H6,2 × Bz-H3/H5), 7.33 – 7.26 (m, 2H, Bz-H3/H5), 7.15 (d, *J* = 7.9 Hz, 2H, STol-H3/H5), 6.22 (t, *J* = 10.0 Hz, 1H, H4), 5.90 (dd, *J* = 3.1, 1.7 Hz, 1H, H2), 5.74 (dd, *J* = 10.0, 3.2 Hz, 1H, H3), 5.62 (d, *J* = 1.5 Hz, 1H, H1), 4.67 (dd, *J* = 10.0, 1.1 Hz, 1H, H5), 4.27 – 4.17 (m, 2H, H6), 4.02 (dd, *J* = 12.0, 1.2 Hz, 1H, H7a), 3.76 (dd, *J* = 12.0, 9.0 Hz, 1H, H7b), 2.35 (s, 3H, STol-CH_3), 1.15 – 0.64 (m, 28H, TIPDS)

^{13}C NMR (101 MHz, $CDCl_3$) δ = 165.7, 165.5, 164.9 (3 × C=O), 138.4 (STol-C1), 133.6, 133.4, 133.3 (3 × Bz-C4), 132.6 (STol-C2/C6), 130.1 (STol-C3/C5), 130.02, 129.96, 129.87 (3 × Bz-C2/C6), 129.7 (STol-C4), 129.5, 129.4, 129.2 (3 × Bz-C1), 128.7, 128.6, 128.5 (3 × Bz-C3/C5), 86.5 (C1), 76.7 (C6), 73.1 (C5), 72.1 (C2), 71.3 (C3), 67.7 (C7), 66.6 (C4), 21.3 (STol-CH_3), 17.50, 17.47, 17.39 (2×), 17.35 (2×), 17.15, 17.13 (8 × TIPDS-CH3), 12.9, 12.7, 12.42, 12.40 (4 × TIPDS-CH)

B.3.13. Synthesis of tol-4-yl 2,3,4-tri-*O*-acetyl-6,7-*O*-(1,1,3,3-tetraisopropyl-1,3-disiloxane-1,3-diyl)-1-thio-D-*glycero*-α-D-*manno*-heptopyranoside (31)

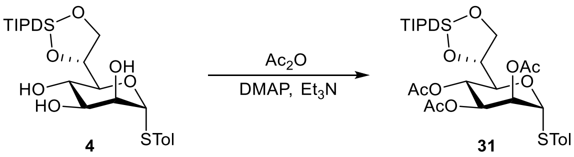

Procedure
Starting triol **4** (203 mg, 0.363 mmol, 1.00 eq.) was dissolved in dry DCM (ca. 4 mL) before first Et_3N (504 µL, 3.63 mmol. 10.0 eq.), Ac_2O (206 µL, 2.18 mmol, 6.00 eq.) and then DMAP (27 mg, 0.218 mmol, 0.6 eq.) was added. The reaction mixture was stirred at rt for 90 min until TLC monitoring (LP/EtOAc = 7:1) indicated full conversion. The mixture was treated with MeOH (147 µl, 3.63 mmol, 10.0 eq.) and THF (ca. 1 mL) to quench the excess of reagent by stirring at rt for 30 min. Then the reaction mixture was diluted with Et_2O, washed with 2 N HCl, water, $NaHCO_3$ and brine and the combined organic layer was dried over Na_2SO_4 and evaporated of solvent to give the crude material

Purification
The crude material was purified *via* column chromatography (45 g SiO_2, gradient LP/EtOAc 20:1 **-** 4:1) to give pure target compound **31** (220 mg, 88%) as colourless resin.

Analytics
R_f-value: 0.29 (LP/EtOAc = 4:1)

$[\alpha]_D^{20}$: +72° (*c* 1.1, $CHCl_3$)

^{1}H NMR (400 MHz, $CDCl_3$) δ = 7.40 (d, *J* = 8.0 Hz, 2H, STol-H2/H6), 7.10 (d, *J* = 7.9 Hz, 2H, STol-H3/H5), 5.47 (t, *J* = 9.8 Hz, 1H, H4), 5.40 (dd, *J* = 3.2, 1.9 Hz, 1H, H2), 5.32 (d, *J* = 1.8 Hz, 1H, H1), 5.27 (dd, *J* = 9.6, 3.2 Hz, 1H, H3), 4.39 (dd, *J* = 10.0, 1.7 Hz, 1H, H5), 4.04 (d, 1H, H6), 3.96 (dd,

J = 12.1, 1.4 Hz, 1H, H7a), 3.73 (dd, *J* = 12.1, 8.6 Hz, 1H, H7b), 2.33 (s, 3H, STol-CH_3), 2.10, 2.04, 2.01 (3 × s, 3 × 3H, 3 × $COCH_3$), 1.15 – 0.88 (m, 28H, TIPDS).

^{13}C NMR (101 MHz, $CDCl_3$) δ = 170.14, 170.08, 169.3 (3 × C=O), 138.4 (STol-C), 132.8 (STol-C2/C6), 130.0 (STol-C3/C5), 129.6 (STol-C), 86.1 (C1), 76.0 (C6), 73.0 (C5), 71.2 (C2), 70.1 (C3), 67.1 (C7), 66.7 (C4), 21.3 (STol-CH_3), 20.96, 20.92, 20.87 (3 × $COCH_3$), 17.6, 17.5, 17.43, 17.40 (8 × TIPDS-CH_3), 13.0, 12.9, 12.53, 12.47 (4 × TIPDS-CH).

B.4. Regioselective partial cleavage of the TIPDS group

B.4.1. Synthesis of methyl 2,4-di-*O*-benzoyl-3-*O*-chloroacetyl-6-*O*-(3-fluoro-1,1,3,3-tetraisopropyl-1,3-disiloxane-1-yl)-L-*glycero*-α-D-*manno*-heptopyranoside (20)

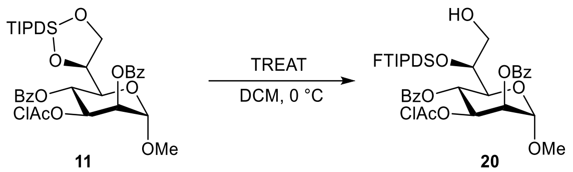

Procedure
Starting material **11** (60 mg, 0.080 mmol, 1.00 eq., 90% pure) was dissolved in dry DCM (1.4 mL), transferred to a teflon vessel and was cooled to 0 °C. Treatment with TREAT (240 µL, 0.240 mmol, 3.00 eq.) and stirring for 60 min with ice bath cooling led to full conversion according to TLC (Hex:EtOAc = 6:1). The reaction mixture was pipetted into ice/$NaHCO_3$/Et_2O, phases were separated, and the organic layer was washed with $NaHCO_3$, brine, dried over Na_2SO_4 and evaporated to give a crude material (48 mg). According to ^1H-NMR the targeted compound was present as main component (75%) together with around 10% of fully desilylated material.

Purification
The crude material was purified *via* column chromatography (20 g SiO_2, 20 mL/min, gradient LP/EtOAc 9:1 – 4:1) to give pure target compound **20** (30 mg, 49%).

Analytics
^1H NMR (400 MHz, $CDCl_3$) δ 8.16 – 8.06 (m, 2H, Bz-H2/H6), 8.02 – 7.92 (m, 2H, Bz-H2/H6), 7.66 – 7.51 (m, 2H, 2×Bz-H4), 7.51 – 7.36 (m, 4H, 2×Bz-H3/H5), 5.97 (t, *J* = 9.9 Hz, 1H, H4), 5.67 (dd, *J* = 9.9, 3.4 Hz, 1H, H3), 5.53 (dd, *J* = 3.4, 1.8 Hz, 1H, H2), 4.91 (d, *J* = 1.7 Hz, 1H, H1), 4.25 – 4.16 (m, 2H, H5, H6), 3.99 – 3.85 (m, 2H, H7a, H7b), 3.89 (d, *J* = 15.1 Hz, 1H, ClAc-CH_2a), 3.83 (d, *J* = 15.1 Hz, 1H, ClAc-CH_2b), 3.49 (s, 3H, OCH_3), 1.08 – 0.80 (m, 28H, FTIPDS)

^{13}C NMR (101 MHz, $CDCl_3$) δ 166.8, 165.9, 165.3 (3 × C=O), 133.7, 133.5 (2 × Bz-C4), 130.2, 129.9 (2 × Bz-C2/C6), 129.5 129.3 (2 × Bz-C1), 128.7, 128.6 (2 × Bz-C3/C5), 98.7 (C1), 72.3 (C3), 71.6 (C6), 70.3 (C5), 70.1 (C2), 66.8 (C4), 63.9 (C7), 55.6 (OCH_3), 40.7 (ClAc-CH_2), 17.46, 17.41, 17.34, 17.31 (4 × FTIPDS-CH_3), 16.79 ($^3J_{C\text{-}F}$, J = 1.0 Hz, FTIPDS-CH_3), 16.77 (d, $^3J_{C\text{-}F}$ = 1.0 Hz, FTIPDS-CH_3), 16.73 (d, J = 1.2 Hz, FTIPDSCH_3), 16.71 (d, J = 1.3 Hz, FTIPDSCH_3), 13.6, 13.2 (2 × FTIPDS-CH), 12.69 (d, $^2J_{C\text{-}F}$ = 16.4 Hz, FTIPDS-CH), 12.66 (d, $^2J_{C\text{-}F}$ = 16.5 Hz, FTIPDS-CH)

B.4.2. Synthesis of tol-4-yl 2,3,4-tri-*O*-benzoyl-6-*O*-(3-fluoro-1,1,3,3-tetraisopropyl-1,3-disiloxane-1-yl)- 1-thio-L-*glycero*-α-D-*manno*-heptopyranoside (22) and tol-4-yl 2,3,4-tri-*O*-benzoyl-1-thio-L-*glycero*-α-D-*manno*-heptopyranoside (22a)

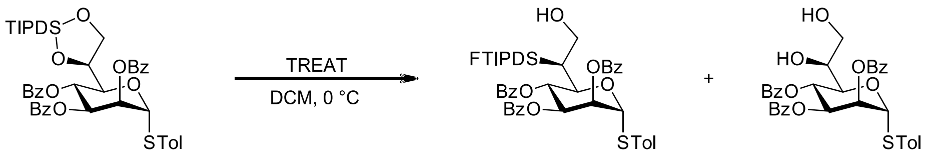

21 **22** **22a**

Procedure

The decorated thioglycoside **21** (110 mg, 0.126 mmol, 1.00 eq.) was dissolved in dry DCM (2.5 mL) and was transferred into a teflon vessel before the mixture was cooled to 0 °C and stirred. After the addition of TREAT (123 µL, 0.757 mmol, 6.00 eq.) in two portions within 30 min, the reaction was closely monitored *via* TLC (LP/EtOAc = 4:1) until after further 40 min, significant amounts of the more polar 6,7-OH by-product were present in the mixture. The reaction was stopped by pouring it into a mixture of $NaHCO_3$, ice and Et_2O, phases were separated, and the organic layer was washed with sat. $NaHCO_3$ and brine. Drying over Na_2SO_4, and filtration gave the crude target compound **22** (110 mg) after evaporation of solvent.

Purification

The crude material was purified *via* column chromatography (10 g SiO_2, CV=15 mL, 10 mL/min, gradient LP/EtOAc 9:1 – 1:1) to give pure target compound **22** (78 mg, 69%). Also, minor amounts of the fully desilylate by-product **22a** were isolated.

Analytics – **22**

R_f-value: 0.52 (LP/EtOAc = 4:1)

$[\alpha]_D^{20}$: -4.5° (*c* 1.1, $CHCl_3$)

^{1}H NMR (400 MHz, $CDCl_3$) δ 8.12 – 8.06 (m, 2H, Bz-H2/H6), 7.99 – 7.92 (m, 2H, Bz-H2/H6), 7.89 – 7.84 (m, 2H, Bz-H2/H6), 7.59 (ddt, *J* = 8.8, 7.0, 1.4 Hz, 1H, Bz-H4), 7.53 – 7.48 (m, 1H, Bz-H4), 7.48 – 7.40 (m, 5H, Bz-H4 STol-H2/H6 Bz-H3/H5), 7.37 (t, *J* = 7.8 Hz, 2H, Bz-H3/H5), 7.32 – 7.25 (m, 2H, Bz-H3/H5), 7.20 – 7.13 (m, 2H, STol-H3/H5), 6.25 (t, *J* = 10.0 Hz, 1H, H4), 5.92 (dd, *J* = 3.3, 1.6 Hz, 1H, H2), 5.82 – 5.76 (m, 2H, H1/H3), 4.73 (dd, *J* = 9.9, 2.2 Hz, 1H, H5), 4.22 (td, *J* = 5.9, 2.1 Hz, 1H, H6), 3.82 – 3.63 (m, 2H, H7a/H7b), 2.35 (s, 3H, STol-CH_3), 1.05 – 0.89 (m, 28H, FTIPDS).

^{13}C NMR (101 MHz, $CDCl_3$) δ 165.72, 165.69, 165.36 (3x C=O), 138.35 (STol-C1), 133.61, 133.35 (2x Bz-C1), 133.30 (BZ-C1), 132.07 (2x STol-C2/C6), 130.24 (2x STol-C3/C5), 130.12, 129.95, 129.90 (3x Bz-C2/C6), 129.58 (STol-C4), 129.41, 129.27, 129.18 (3x Bz-C4), 128.62 (2xBz-C3/C5), 128.45 (4x Bz-C3/C5), 85.87 (C1), 72.12 (C2), 71.62, 71.57, 71.54 (3x C3/C5/C6), 66.80 (C4), 63.73 (C7), 21.29 (STol-CH_3), 17.48, 17.45, 17.36, 17.33, 16.76, 16.70 (8 × FTIPDS-CH_3), 13.61, 13.31 (2 × FTIPDS-CH), 12.66 (d, $^2J_{C\text{-}F}$ = 16.3 Hz, FTIPDS-CH), 12.63 (d, $^2J_{C\text{-}F}$ = 16.4 Hz, FTIPDS-CH).

Analytics – by-product **22a**

R_f-value 0.12 (LP/EtOAc = 1:1)

^{1}H NMR (400 MHz, $CDCl_3$) δ = 8.10 (d, *J* = 6.6 Hz, 2H, Bz-H2/H6), 8.01 (d, *J* = 7.1 Hz, 2H, Bz-H2/H6), 7.84 (d, *J* = 7.1 Hz, 2H, Bz-H2/H6), 7.61 (ddt, *J* = 7.9, 6.9, 1.3 Hz, 1H, Bz-H4), 7.55 (ddt, *J* = 7.9, 6.9, 1.3 Hz, 1H, Bz-H4), 7.51 – 7.36 (m, 7H, Bz-H4, 2 × Bz-H3/H5, STol-H2/H6), 7.32 – 7.20 (m, 3H, Bz-H3/H5), 7.17 (d, 2H, STol-H3/H5), 6.07 – 5.89 (m, 3H, H2, H3, H4), 5.79 (d, *J* = 1.5 Hz, 1H, H1), 4.53 (dd, *J* = 9.3, 1.4 Hz, 1H, H5), 3.80 (d, *J* = 6.0 Hz, 1H, H6), 3.68 (dd, *J* = 11.4, 7.1 Hz, 1H, H7a), 3.49 (dd, *J* = 11.4, 4.8 Hz, 1H, H7b), 3.19 (s, 1H, 6-OH), 2.35 (s, 3H, STol-CH_3).

^{13}C NMR (101 MHz, $CDCl_3$) δ 166.8, 165.4 (3 × C=O), 138.6, 133.9, 133.7, 133.3, 132.3, 130.7, 130.5, 130.2, 130.1, 130.02, 130.96, 129.85, 129.7, 129.1, 128.9, 128.7, 128.6, 128.43, 128.36, 128.31, 127.0,

124.6 (24 × Ar-C), 85.9 (C1), 71.8, 71.7 (2 × C5/C6), 69.9 (C3), 68.9 (C3), 67.5 (C4), 63.5 (C7), 21.2 (STol-CH_3).

B.4.3. Synthesis of tol-4-yl 2,3,4-tri-*O*-acetyl-6-*O*-(3-fluoro-1,1,3,3-tetraisopropyl-1,3-disiloxane-1-yl)-1-thio-L-*glycero*-α-D-*manno*-heptopyranoside (24) and tol-4-yl 2,3,4-tri-*O*-acetyl-1-thio-L-*glycero*-α-D-*manno*-heptopyranoside (24a)

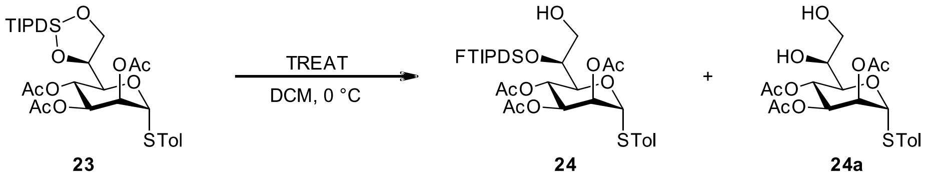

Procedure

Starting material **23** (106 mg, 0.154 mmol, 1.00 eq.) was dissolved in dry DCM (1 mL) and transferred into a teflon vessel. The solution was cooled to 0 °C and TREAT (84 µL, 0.51 mmol, 3.3 eq.) was added dropwise within 5 min. Close TLC monitoring (LP/EtOAc = 4:1) indicated slow progress of the reaction, therefore a second addition of TREAT (84 µL, 0.51 mmol, 3.3 eq.) was needed after 2 h. After further stirring at 0 °C for 2 h, the solution was pipetted into a mixture of Et_2O, $NaHCO_3$ and ice. Layers were separated, the combined organic layer was washed with $NaHCO_3$ and brine, dried over Na_2SO_4 and evaporated to yield the crude material (91 mg).

Purification

The crude material was purified *via* column chromatography (10 g SiO_2, 12 mL CV, 20 mL/min, gradient LP/EtOAc 10:1 – 1:1), giving pure target compound **24** (65 mg, 60%) and also 6,7-OH by-product **24a** (6 mg).

Analytics – **24**

R_f-value: 0.27 (LP/EtOAc = 4:1)

$[\alpha]_D^{20}$: -6.2° (*c* 1.1, $CHCl_3$)

^1H NMR (400 MHz, $CDCl_3$) δ = 7.35 (d, *J* = 8.1 Hz, 2H, STol-H2/H6), 7.12 (d, *J* = 7.9 Hz, 2H, STol-H3/H5), 5.57 (t, *J* = 9.8 Hz, 1H, H4), 5.48 (d, *J* = 1.7 Hz, 1H, H1), 5.43 (dd, *J* = 3.3, 1.7 Hz, 1H, H2), 5.33 (dd, *J* = 9.8, 3.3 Hz, 1H, H3), 4.36 (dd, *J* = 9.9, 1.8 Hz, 1H, H5), 4.07 (td, *J* = 6.0, 1.9 Hz, 1H, H6), 3.67 (dt, *J* = 11.4, 5.7 Hz, 1H, H7a), 3.61 – 3.51 (m, 1H, H7b), 2.32 (s, 3H, STol-CH_3), 2.10, 2.01 (3 × s, 3 × 3H, 3 × $COCH_3$), 1.22 – 0.89 (m, 28H, FTIPDS)

^{13}C NMR (101 MHz, $CDCl_3$) δ = 170.1, 170.0, 169.4 (3 × C=O), 138.2 (STol-C1), 132.0 (2 × STol-C2/C6), 130.0 (STol-C3/C5), 129.0 (STol-C4), 85.4 (C1), 71.1, 71.1 (2 × C5/C6), 71.0 (C2), 70.3 (C3), 66.3 (C4), 63.7 (C7), 21.1 (STol-CH_3), 20.9, 20.8, 20.8 (3 × $COCH_3$), 17.52, 17.45, 17.3, 16.79, 16.75, 16.74, 16.71 (8 × FTIPDS-CH_3), 13.6, 13.4 (2 × FTIPDS-CH), 12.6 (d, $^2J_{C-F}$ = 16.5 Hz, 2 × FTIPDS-CH)

Analytics – by-product **24a**

R_f-value: 0.08 (LP/EtOAc = 1:1)

^1H NMR (600 MHz, $CDCl_3$) δ = 7.33 – 7.30 (m, 2H, STol-H2/H6), 7.17 – 7.12 (m, 2H, STol-H3/H5), 5.53 – 5.49 (m, 2H, H1/H3), 5.44 – 5.38 (m, 2H, H2/H4), 4.26 – 4.19 (m, 1H, H5), 3.68 – 3.62 (m, 1H, H6), 3.60 (dd, *J* = 11.2, 7.3 Hz, 1H, H7a), 3.42 (dd, *J* = 11.2, 4.4 Hz, 1H, H7b), 2.34 (s, 3H, STol-CH_3), 2.15, 2.12, 2.03 (3 × s, 3 × 3H, 3 × $COCH_3$)

^{13}C NMR (151 MHz, $CDCl_3$) δ = 171.4, 170.1, 167.0 (3 × C=O), 138.7 (STol-C1), 132.2 (STol-C2/C6), 130.3 (STol-C3/C5), 128.5 (STol-C4), 85.8 (C1), 71.5 (C5), 70.7 (C2), 69.2 (C3), 68.9 (C6), 66.8 (C4), 63.5 (C7), 21.3 (STol-CH_3), 21.1, 21.0, 20.8 (3 × $COCH_3$)

B.4.4. Synthesis of tol-4-yl 2,4-di-*O*-benzoyl-3-*O*-chloroacetyl-6-*O*-(3-fluoro-1,1,3,3-tetraisopropyl-1,3-disiloxane-1-yl)- 1-thio-L-*glycero*-α-D-*manno*-heptopyranoside (26)

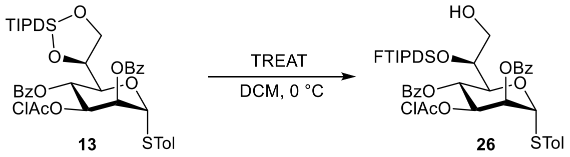

Procedure

Starting material **13** (177 mg, 0.210 mmol, 1.00 eq.) was dissolved in dry DCM (1.5 mL) and transferred into a teflon vessel. The solution was cooled to 0 °C followed by dropwise addition of TREAT (173 µL, 1.05 mmol, 5.00 eq.). The reaction was stirred at 0 °C for 3 h and was monitored *via* TLC (LP/EtOAc = 8:1). After 3 h the reaction was nearly complete with small amounts of starting material and already some 6,7-diole present; at that point the reaction mixture was pipetted into a mixture of Et_2O, $NaHCO_3$ and ice. Layers were separated, the combined organic layer was washed with $NaHCO_3$ and brine, dried over Na_2SO_4 and evaporated to yield the crude material (185 mg).

Purification

An aliquot of 79 mg was purified *via* column chromatography (4 g SiO_2, 5 mL CV, 18 mL/min, gradient LP/EtOAc 15:1 – 2:1) to give pure **26** as colourless glass (54 mg, representing 70%)

Analytics

R_f-value: 0.30 (TLC, LP/EtOAc = 8:1)

$[\alpha]_D^{20}$: -4.5° (*c* 1.1, $CHCl_3$)

^{1}H-NMR (400 MHz, $CDCl_3$) δ = 8.15 – 8.09 (m, 2H, Bz-H2/H6), 8.05 – 7.98 (m, 2H, Bz-H2/H6), 7.64 – 7.55 (m, 2H, 2 × Bz-H4), 7.49 – 7.40 (m, 6H, STol-H2/H6, 2 × Bz-H3/H5), 7.16 (d, *J* = 7.9 Hz, 2H, STol-H3/H5), 6.05 (t, *J* = 9.9 Hz, 1H, H4), 5.81 (dd, *J* = 3.3, 1.6 Hz, 1H, H2), 5.71 (d, *J* = 1.6 Hz, 1H, H1), 5.68 (dd, *J* = 9.9, 3.3 Hz, 1H, H3), 4.67 (dd, *J* = 10.0, 2.2 Hz, 1H, H5), 4.18 (td, *J* = 5.9, 2.3 Hz, 1H, H6), 3.96 – 3.83 (m, 2H, ClAc-CH_2), 3.78 (dt, *J* = 11.5, 5.8 Hz, 1H, H7a), 3.68 (dt, *J* = 11.3, 6.4 Hz, 1H, H7b), 2.35 (s, 3H, STol-CH_3), 1.83 (td, *J* = 6.4, 2.2 Hz, 1H, 7-OH), 1.06 – 0.85 (m, 28H, FTIPDS)

^{13}C-NMR (101 MHz, $CDCl_3$) δ = 166.7, 165.6, 165.1 (3 × C=O), 138.4 (STol-C1), 133.7, 133.5 (4 × Bz-C4), 132.1 (2 × STol-C2/C6), 130.14 (2 × STol-C3/C5), 130.06, 129.9 (4 × Bz-C2/C6), 129.2, 129.0, 128.7 (3 × Ar-C), 128.54, 128.47 (4 × Bz-C3/C5), 85.6 (C1), 72.5 (C3), 71.4, 71.3 (C2, C5, C6), 66.6 (C4), 63.5 (C7), 40.5 (ClAc-CH_2), 21.2 (STol-CH_3), 17.45 (d, $^3J_{C\text{-}F}$ = 3.3 Hz, FTIPS-CH_3), 17.32 (d, $^3J_{C\text{-}F}$ = 2.8 Hz, FTIPS-CH_3), 16.76 (d, $^4J_{C\text{-}F}$ = 1.3 Hz, FTIPS-CH_3), 16.74 (d, $^4J_{C\text{-}F}$ = 1.3 Hz, FTIPS-CH_3), 16.70 (d, $^4J_{C\text{-}F}$ = 1.1 Hz, FTIPS-CH_3), 16.68 (d, $^4J_{C\text{-}F}$ = 1.3 Hz, FTIPDS-CH_3), 13.60, 13.28 (2 × FTIPDS-CH), 12.65 (d, $^2J_{C\text{-}F}$ = 16.3 Hz, FTIPDS-CH), 12.62 (d, $^2J_{C\text{-}F}$ = 16.5 Hz, FTIPDS-CH)

B.4.5. Synthesis of tol-4-yl 2,3-*O*-(2-chloro-1-ethoxy-ethyliden-1,1-diyl)- 6-*O*-(3-fluoro-1,1,3,3-tetraisopropyl-1,3-disiloxane-1-yl)-1-thio-L-*glycero*-α-D-*manno*-heptopyranoside (27)

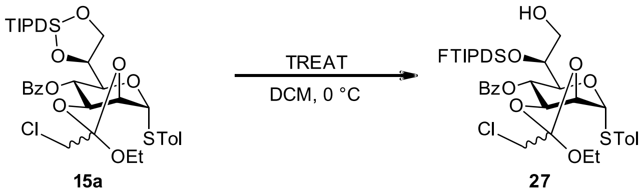

Procedure

Starting material **15a** (20.0 mg, 0.026 mmol, 1.00 eq.) was dissolved in dry DCM (1 mL) and transferred into a teflon vessel. The mixture was cooled to 0 °C. TREAT (21.2 µL, 0.130 mmol, 5.00 eq.) was added

dropwise within 5 min and the reaction was stirred at 0 °C. Monitoring *via* TLC (LP/EtOAc = 8:1) indicated that nearly no conversion was achieved after 1 h, therefore another portion of TREAT (10 µL, 0.065 mmol, 2.50 eq.) was added. A last addition of TREAT (10.0 µL, 0.065 mmol, 2.50 eq.) after overall 2 h of reaction time drove the reaction to convincing conversion within the next 1 h and the reaction mixture was pipetted into a mixture of Et_2O, $NaHCO_3$ and ice. Layers were separated in a separation funnel. The combined organic layer was washed with $NaHCO_3$ and brine, was dried over Na_2SO_4 and was evaporated to give the crude material of compound **27** (22 mg)

Purification

The crude material was purified *via* column chromatography (4 g SiO_2, 5 mL CV, 15 mL/min, gradient LP/EtOAc 20:1 – 8:1) gave pure target compound **27** (15 mg, 75 %)

Analytics

R_f-value: 0.30 (LP/EtOAc = 8:1)

^{1}H NMR (600 MHz, $CDCl_3$) δ = 8.11 – 7.97 (m, 2H, Bz-H2/H6), 7.57 (ddt, J = 8.7, 7.4, 1.3 Hz, 1H, Bz-H4), 7.48 – 7.41 (m, 2H, Bz-H3/H5), 7.41 – 7.35 (m, 2H, STol-H2/H6), 7.17 – 7.13 (m, 2H, STol-H3/H5), 5.89 (d, J = 1.6 Hz, 1H, H1), 5.83 (dd, J = 9.7, 6.4 Hz, 1H, H4), 4.70 – 4.52 (m, 2H, H2, H3), 4.42 (dd, J = 9.7, 2.6 Hz, 1H, H5), 4.06 (ddd, J = 6.5, 5.3, 2.6 Hz, 1H, H6), 3.92 (dq, J = 8.8, 7.0 Hz, 1H, OEt-CH_2a), 3.86 (dq, J = 8.8, 7.1 Hz, 1H, OEt-CH_2b), 3.72 – 3.65 (m, 2H, ClAc-CH_2), 3.62 (dt, J = 11.5, 5.8 Hz, 1H, H7a), 3.55 (dt, J = 11.2, 6.3 Hz, 1H, H7b), 2.35 (s, 3H, STol-CH_3), 1.28 (t, J = 7.1 Hz, 3H, OEt-CH_3), 1.01 – 0.87 (m, 28H, FTIPDS).

^{13}C NMR (151 MHz, $CDCl_3$) δ = 163.9 (Bz-C=O), 136.9 (STol-C1), 132.2 (Bz-C4), 130.2 (2x STol-C2/C6), 129.0 (2 × STol-C3/C5), 128.9 (2 × Bz-C2/C6), 128.6, 127.8 (2 × Ar-C), 127.3 (2 × Bz-C3/C5), 119.8 (orthoester-C), 83.0 (C1), 76.5 (2 × C2/C3), 70.4 (C6), 68.4 (C5), 68.0 (C4), 62.2 (C7), 58.1 (OEt-CH_2), 45.1 (ClAc-CH_2), 20.1 (STol-CH_3), 16.2, 16.1, 16.02, 15.98, 15.62, 15.59, 15.55, 15.53 (8 × FTIPDS-CH_3), 13.9 (OEt-CH_3), 13.40, 13.12 (2 × FTIPDS-CH), 12.63 (d, $^2J_{C\text{-}F}$ = 16.4 Hz, FTIPDS-CH), 12.60 (d, $^2J_{C\text{-}F}$ = 16.5 Hz, FTIPDS-CH).

B.4.6. Synthesis of tol-4-yl 3,4-di-*O*-benzoyl-2-*O*-chloroacetyl-6-*O*-(3-fluoro-1,1,3,3-tetraisopropyl-1,3-disiloxane-1-yl)- 1-thio-L-*glycero*-α-D-*manno*-heptopyranoside (28)

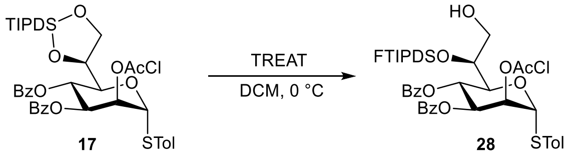

Procedure

Starting material **17** (12 mg, 0.014 mmol, 1.00 eq.) was dissolved in dry DCM (0.2 mL) and was transferred into a teflon vessel. The solution was cooled to 0 °C before TREAT (58 µL, 0.350 mmol, 25 eq.) was added. The solution was stirred for 25 min before a second portion of TREAT (58 µL, 0.350 mmol, 25 eq.) was added. Next, the reaction mixture was allowed to warm up to rt, TLC monitoring (LP/EtOAc = 4:1) indicated complete consumption of the starting material. The reaction mixture was poured into a mixture of $NaHCO_3$ and ice. Et_2O was used to dilute the solution. Layers were sperated and the organic layer was washed with brine and was dried over $MgSO_4$. The solvent was evaporated to give the crude material (9 mg).

Purification

The crude material was purified *via* filtration through a SiO_2 filled Pasteur pipette (LP/EtOAc = 5:1) to give target compound **28** (3 mg, 24%).

Analytics

R_f-value: 0.22 (LP:EtOAc 5:1)

1H NMR (600 MHz, $CDCl_3$) δ 7.98 – 7.93 (m, 2H, Bz-H2/6), 7.94 – 7.89 (m, 2H, Bz-H2/6), 7.53 – 7.49 (m, 2H, 2 × Bz-H4), 7.42 (d, *J* = 8.1 Hz, 2H, STol-H2/H6), 7.40 – 7.34 (m, 4H, 2 × Bz-H3/5), 7.17 (d, *J* = 7.9 Hz, 2H, STol-H3/H5), 6.06 (t, *J* = 9.9 Hz, 1H, H4), 5.76 (dd, *J* = 3.1, 1.7 Hz, 1H, H2), 5.69 (dd, *J* = 9.9, 3.2 Hz, 1H, H3), 5.62 (d, *J* = 1.5 Hz, 1H, H1), 4.64 (dd, *J* = 9.9, 1.8 Hz, 1H, H5), 4.18 (dt, *J* = 6.0, 3.0 Hz, 1H, H6), 4.15 – 4.05 (m, 2H, ClAc-CH_2), 3.75 (dt, *J* = 11.6, 5.9 Hz, 1H, H7a), 3.65 (dt, *J* = 11.3, 6.4 Hz, 1H, H7b), 2.36 (s, 3H, STol-CH_3), 1.03 – 0.92 (m, 28H, TIPDSF)

^{13}C NMR (151 MHz, $CDCl_3$) δ 166.5, 165.5, 165.1 (3 × C=O), 138.5 (STol-C1), 133.4, 133.3 (2 × Bz-C4), 132.2 (STol-CH), 130.1 (STol-CH), 129.83, 129.75 (2 × Bz-C2/6), 129.3, 128.9, 128.8 (2 × Bz-C1 / STol-C4), 128.5, 128.4 (2 × Bz-C3/5), 85.3 (C1), 73.1 (C2), 71.5 (C5), 71.3 (C6), 71.1 (C3), 66.2 (C4), 63.6 (C7), 40.5 (ClAc-CH_2), 21.2 (STol-CH_3), 17.34, 17.29, 17.23, 17.20, 16.64, 16.63, 16.59, 16.57 (8 × FTIPDS-CH_3), 12.57, 12.54, 12.46, 12.43 (4 × FTIPDS-CH).

B.4.7. Synthesis of tol-4-yl 2,3,4-tri-*O*-benzoyl-6-*O*-(3-fluoro-1,1,3,3-tetraisopropyl-1,3-disiloxane-1-yl)-1-thio-D-*glycero*-α-D-*manno*-heptopyranoside (30)

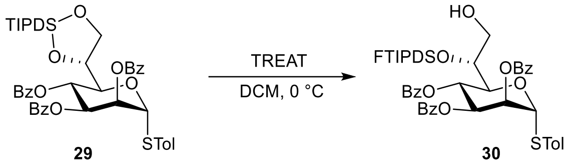

<u>Procedure</u>
The decorated thioglycoside **29** (109 mg, 0.125 mmol, 1.00 eq.) was dissolved in dry DCM (1.5 mL) and was transferred into a teflon vessel before the mixture was cooled to 0 °C and stirred. After the addition of TREAT (123 µL, 0.757 mmol, 6.00 eq.) in two portions over 45 min, the reaction was closely monitored *via* TLC (LP/EtOAc = 4:1) after a further 2 h of stirring at 0 °C, significant amounts of the more polar 6,7-OH by-product were present in the mixture. The reaction was stopped by pouring it into a mixture of $NaHCO_3$, ice and Et_2O, phases were separated, and the organic layer was washed with sat. $NaHCO_3$ and brine. Drying over Na_2SO_4, and filtration gave the curde target compound **30** after evaporation of solvent.

<u>Purification</u>
The crude material was purified *via* column chromatography (9 g SiO_2, 10 mL/min, gradient LP/EtOAC = 9:1 – 2:1) to give pure target compound **30** (64 mg, 57%) as colourless oil.

<u>Analytics</u>
R_f-value 0.47 (LP/EtOAc = 4:1)

$[\alpha]_D^{20}$: +7.2° (*c* 0.5, $CHCl_3$)

^{1}H NMR (400 MHz, $CDCl_3$) δ = 8.07 (dd, *J* = 8.4, 1.4 Hz, 2H, BzH2/H6), 7.98 (dd, *J* = 8.4, 1.4 Hz, 2H, Bz-H2/H6), 7.84 (dd, *J* = 8.4, 1.3 Hz, 2H, Bz-H2/H6), 7.59 (t, 1H, BzH4), 7.55 – 7.33 (m, 7H, 2 × Bz-H3/H5, 2 × Bz-H4, STol-H3/H5), 7.31 – 7.22 (m, 2H, Bz-H3/H5), 7.12 (dd, *J* = 8.4, 0.5 Hz, 2H, STol-H2/H6), 6.17 (t, *J* = 10.2 Hz, 1H, H4), 5.90 (dd, *J* = 3.2, 1.6 Hz, 1H, H2), 5.78 (dd, *J* = 10.0, 3.2 Hz, 1H, H3), 5.64 (d, *J* = 1.6 Hz, 1H, H1), 4.89 (dd, *J* = 10.3, 0.6 Hz, 1H, H5), 4.20 (td, *J* = 5.8, 1.3 Hz, 1H, H6), 3.95 – 3.74 (m, 2H, H7a, H7b), 2.34 (s, 3H, STol-CH_3), 1.14 – 0.77 (m, 28H, FTIPDS)

^{13}C NMR (101 MHz, $CDCl_3$) δ = 165.6, 165.5 (3 × C=O), 138.4 (STol-C1), 133.7, 133.6, 133.4 (3 × Bz-C4), 132.9 (STol-C3/C5), 130.0 (Bz-C2/C6), 130.04 (STol-C2/C6), 130.96, 129.9 (2 × Bz-C2/C6), 129.7 (STol-C4), 129.4, 129.2, 129.1 (3 × Bz-C1), 128.7, 128.6, 128.5 (3 × Bz-C3/C5), 86.8 (C1), 74.1 (C5), 72.5 (C2), 72.3 (C6), 71.1 (C3), 67.5 (C4), 64.1 (C7), 21.3 (STol-CH_3), 17.3, 17.1, 16.7, 16.7 (8 × FTIPDS-CH_3), 13.3 (FTIPDS-CH), 13.1 (FTIPS-CH), 12.63 (d, $^2J_{C\text{-}F}$ = 16.5 Hz, FTIPS-CH), 12.60 (d, $^2J_{C\text{-}F}$ = 16.2 Hz, FTIPDS-CH)

B.4.8. Synthesis of tol-4-yl 2,3,4-tri-*O*-acetyl-6-*O*-(3-fluoro-1,1,3,3-tetraisopropyl-1,3-disiloxane-1-yl)- 1-thio-D-*glycero*-α-D-*manno*-heptopyranoside (32)

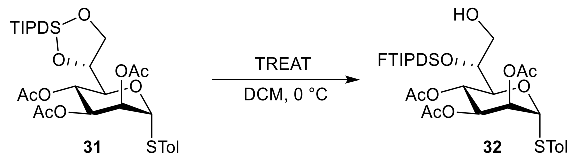

Procedure
Starting material **31** (110 mg, 0.161 mmol, 1.00 eq.) was dissolved in dry DCM (1.5 mL) and transferred into a teflon vessel. The solution was cooled to 0 °C and TREAT (79 µL, 0.48 mmol, 3.0 eq.) was added dropwise within 5 min. Close TLC monitoring (LP/EtOAc = 4:1) indicated about 50% conversion, therefore the reaction mixture was allowed to warm to rt. After stirring for another 30 min, the amount of by-product became significant, therefore the reaction mixture was pipetted into a mixture of Et_2O, $NaHCO_3$ and ice. Layers were separated, the combined organic layer was washed with $NaHCO_3$ and brine, dried over Na_2SO_4 and evaporated to yield the crude material.

Purification
The crude material was purified *via* column chromatography (9 g SiO_2, 12 mL CV, 10 mL/min, gradient LP/EtOAc 9:1 – 2:1), giving pure target compound **32** (77 mg, 68%)

R_f-value: 0.21 (LP/EtOAc = 7:1)

$[\alpha]_D^{20}$: +61° (*c* 1.2, $CHCl_3$)

^{1}H NMR (400 MHz, $CDCl_3$) δ 7.39 (d, *J* = 8.2 Hz, 2H, STol-H2/H6), 7.09 (d, *J* = 7.9 Hz, 2H, STol-H3/H5), 5.50 (t, *J* = 10.0 Hz, 1H, H4), 5.45 (dd, *J* = 3.4, 1.7 Hz, 1H, H2), 5.34 (d, *J* = 1.7 Hz, 1H, H1), 5.27 (dd, *J* = 9.8, 3.3 Hz, 1H, H3), 4.54 (d, *J* = 10.2 Hz, 1H, H5), 4.08 (td, *J* = 5.7, 1.3 Hz, 1H, H6), 3.78 – 3.66 (m, 2H, H7a, H7b), 2.32 (s, 3H, STol-CH_3), 2.11, 2.04, 2.00 (3 × s, 3 × 3H, 3 × $COCH_3$), 1.14 – 0.91 (m, 28H, TIPDS)

^{13}C NMR (101 MHz, $CDCl_3$) δ = 170.08, 170.07, 169.61 (3 × C=O), 138.37 (STol-C1), 132.73 (STol-C2/C6), 129.98 (STol-C3/C5), 129.64 (STol-C4), 86.53 (C1), 73.65 (C5), 72.63 (C6), 71.30 (C2), 70.14 (C3), 66.78 (C4), 64.06 (C7), 21.29 (STol-CH_3), 20.98, 20.92, 20.83 (3 × $COCH_3$), 17.30, 17.24, 17.19, 17.16, 16.86, 16.81, 16.77 (8 × TIPDS-CH_3), 13.37, 13.11 (2 × FTIPDS-CH), 12.69 (d, $^2J_{C\text{-}F}$ = 16.4 Hz, FTIPDS-CH), 12.68 (d, $^2J_{C\text{-}F}$ = 16.3 Hz, FTIPDS-CH)

B.4.9. Synthesis of tol-4-yl 2,3-di-*O*-benzoyl-4-*O*-chloroacetyl-6-*O*-(3-fluoro-1,1,3,3-tetraisopropyl-1,3-disiloxane-1-yl)- 1-thio-D-*glycero*-α-D-*manno*-heptopyranoside (33)

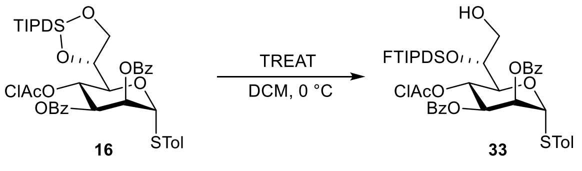

Procedure
Decorated thio-glycoside **16** (125 mg, 0.148 mmol, 1.00 eq.) was dissolved in dry DCM (3 mL), transferred into a teflon vessel and cooled to 0 °C. The mixture was stirred and TREAT (72 µL, 0.445 mmol, 3.00 eq.) was added slowly. The reaction progress was analysed *via* TLC (LP/EtOAc = 4:1). After 30 min, the cooling was removed, and the reaction mixture was allowed to warm to rt. After 3 h the amount of polar by-product visible on TLC became significant, therefore the reaction mixture was pipetted into a mixture of Et_2O, $NaHCO_3$ and ice. Layers were separated, the combined organic layer was washed with $NaHCO_3$ and brine, dried over Na_2SO_4 and evaporated to yield the crude material.

<u>Purification</u>
The crude material was purified *via* column chromatography (9 g SiO_2, 10 mL/min, gradient LP/EtOAC = 20:1 – 2:1) to give mostly pure target compound **33** (87 mg, 58%) as colourless oil.

<u>Analytics</u>
R_f-value: 0.50 (LP/EtOAc = 4:1)

^{1}H NMR (400 MHz, $CDCl_3$) δ = 8.03 (d, *J* = 7.3 Hz, 2H, Bz-H2/H6), 7.91 (d, *J* = 7.3 Hz, 2H, Bz-H2/H6), 7.58 (t, *J* = 7.4 Hz, 1H, Bz-H4), 7.51 (t, *J* = 7.5 Hz, 1H, Bz-H4), 7.48 – 7.41 (m, 4H, Bz-H3/H5, STol-H2/H6), 7.36 (t, *J* = 7.7 Hz, 2H, Bz-H3/H5), 7.12 (d, *J* = 7.9 Hz, 2H, STol-H3/H5), 6.01 (t, *J* = 10.1 Hz, 1H, H4), 5.87 – 5.83 (m, 1H, H2), 5.64 (dd, *J* = 10.0, 3.2 Hz, 1H, H3), 5.61 (s, 1H, H1), 4.77 (d, *J* = 10.2 Hz, 1H, H5), 4.17 (t, *J* = 5.8 Hz, 1H, H6), 4.03 – 3.91 (m, 2H, ClAc-CH_2), 3.87 – 3.74 (m, 2H, H7a, H7b), 2.33 (s, 3H, STol-CH_3), 1.13 – 0.90 (m, 28H, TIPDS).

^{13}C NMR (101 MHz, $CDCl_3$) δ = 166.5, 165.58, 165.56 (3 × C=O), 138.5 (STol-C1), 133.7, 133.6 (2 × Bz-C4), 132.9 (STol-C2/C6), 130.04, 130.00, 129.96 (STol-C3/C5, 2 × Bz-C2/C6), 129.4, 129.3 (2 × Bz-C1), 128.9 (STol-C4), 128.7, 128.6 (2 × Bz-C3/C5), 86.6 (C1), 73.2 (C5), 72.6 (C6), 72.3 (C2), 71.0 (C3), 68.6 (C4), 64.0 (C7), 40.7 (ClAc-CH_2), 21.3 (STol-CH_3), 17.2, 17.1, 16.85, 16.79, 16.75, 16.7 (8 × FTIPDS-CH_3), 13.4, 13.2, 12.8, 12.6 (4 × FTIPDS-CH).

C. Recorded NMR-spectra

C.1. Preparation of triol starting materials

C.1.1. Tol-4-yl 2,3,4,6,7-penta-*O*-acetyl-1-thio-L-*glycero*-α-D-*manno*-heptopyranoside (5)

1H NMR (CDCl3, 700 MHz)

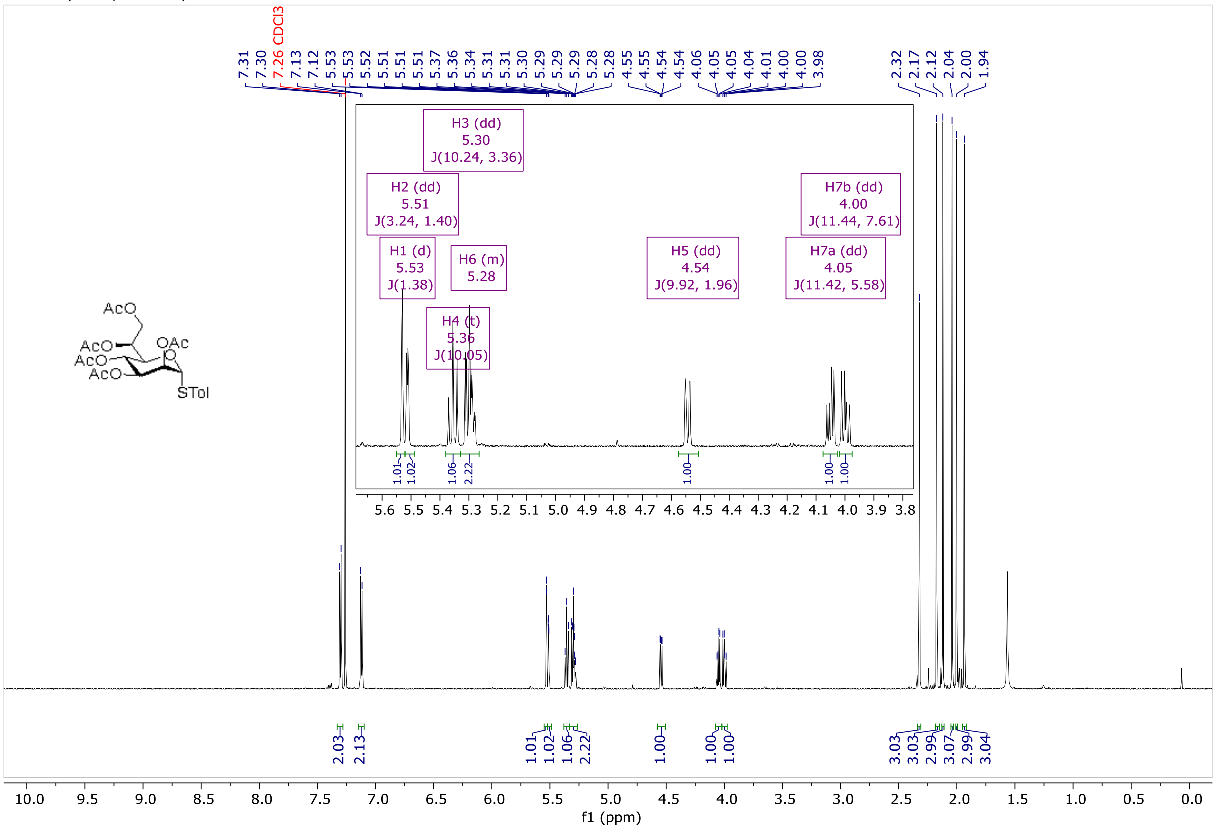

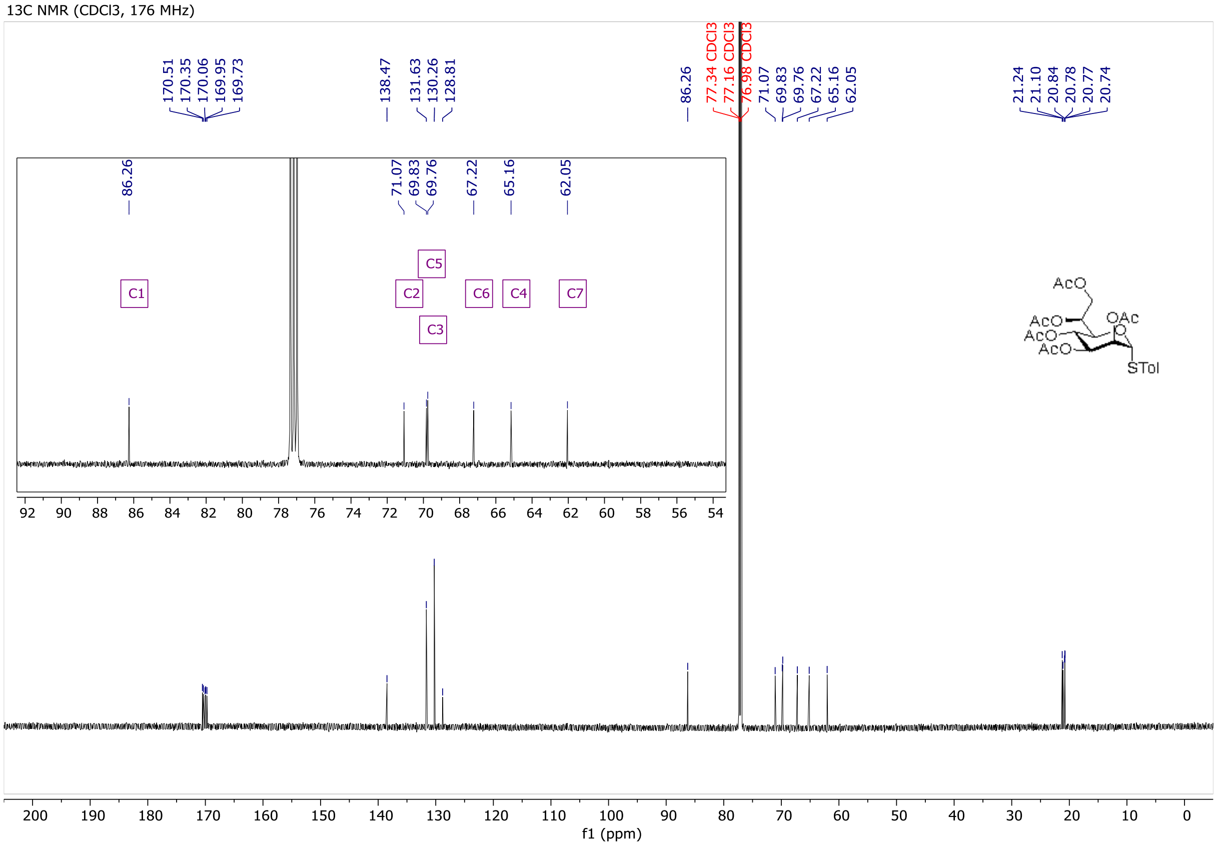

C.1.2. Tol-4-yl 1-thio-D-*glycero*-α-D-*manno*-heptopyranoside (7)

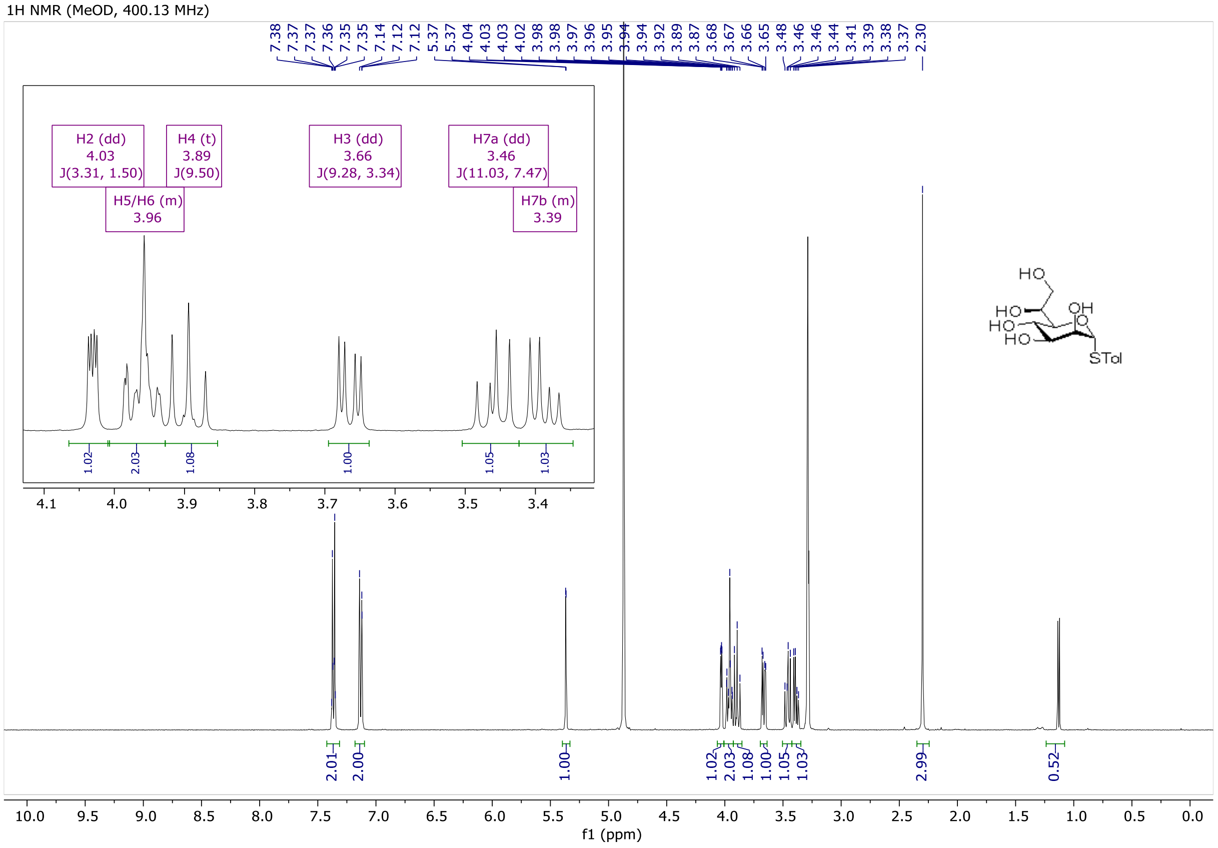

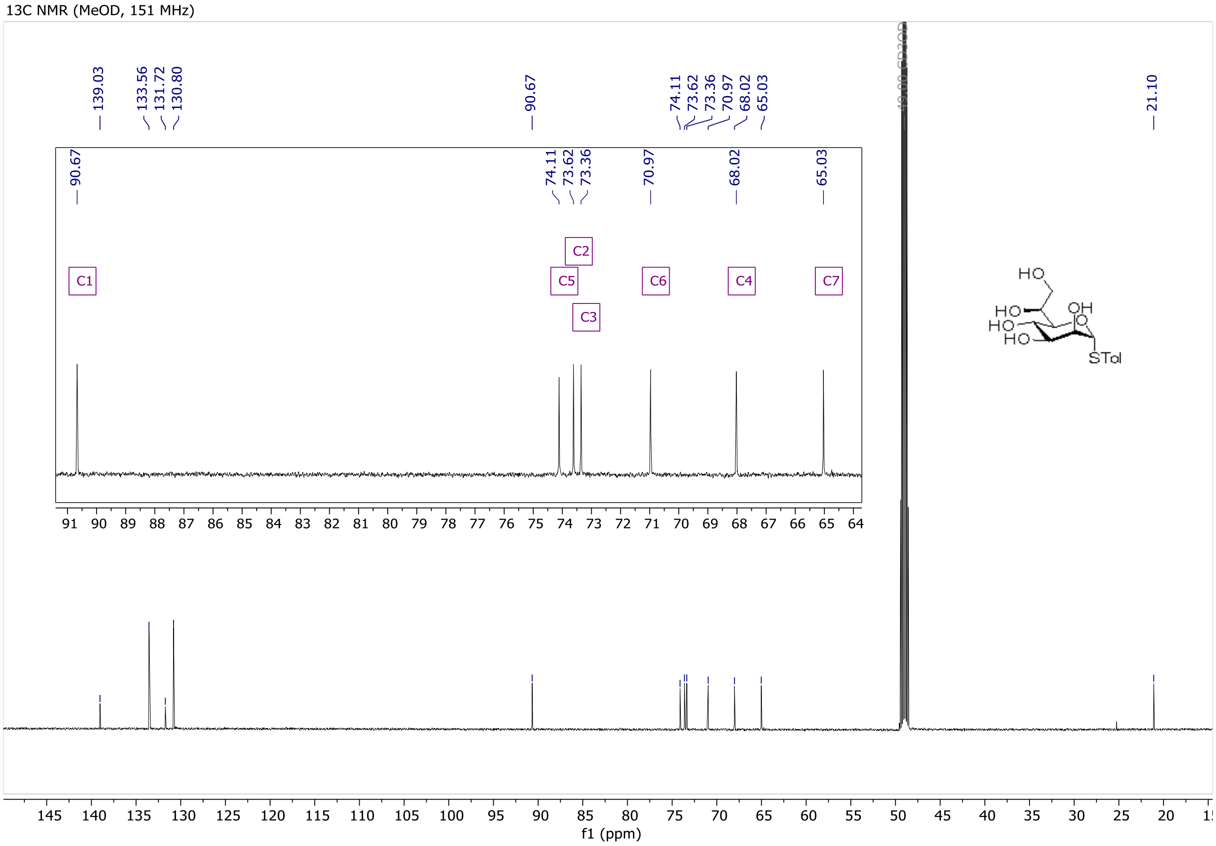

C.1.3. Tol-4-yl 6,7-*O*-(1,1,3,3-tetraisopropyl-1,3-disiloxane-1,3-diyl)-1-thio-L-*glycero*-α-D-*manno*-heptopyranoside (3)

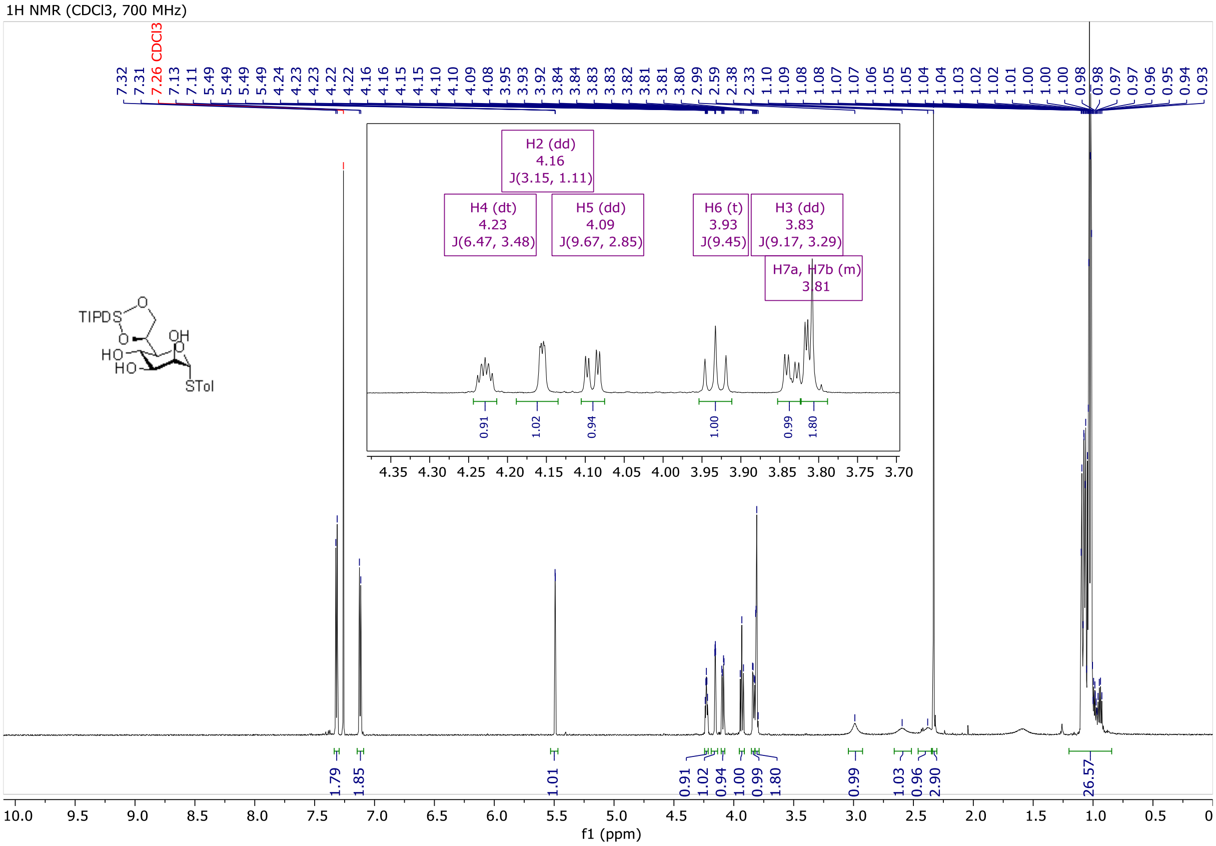

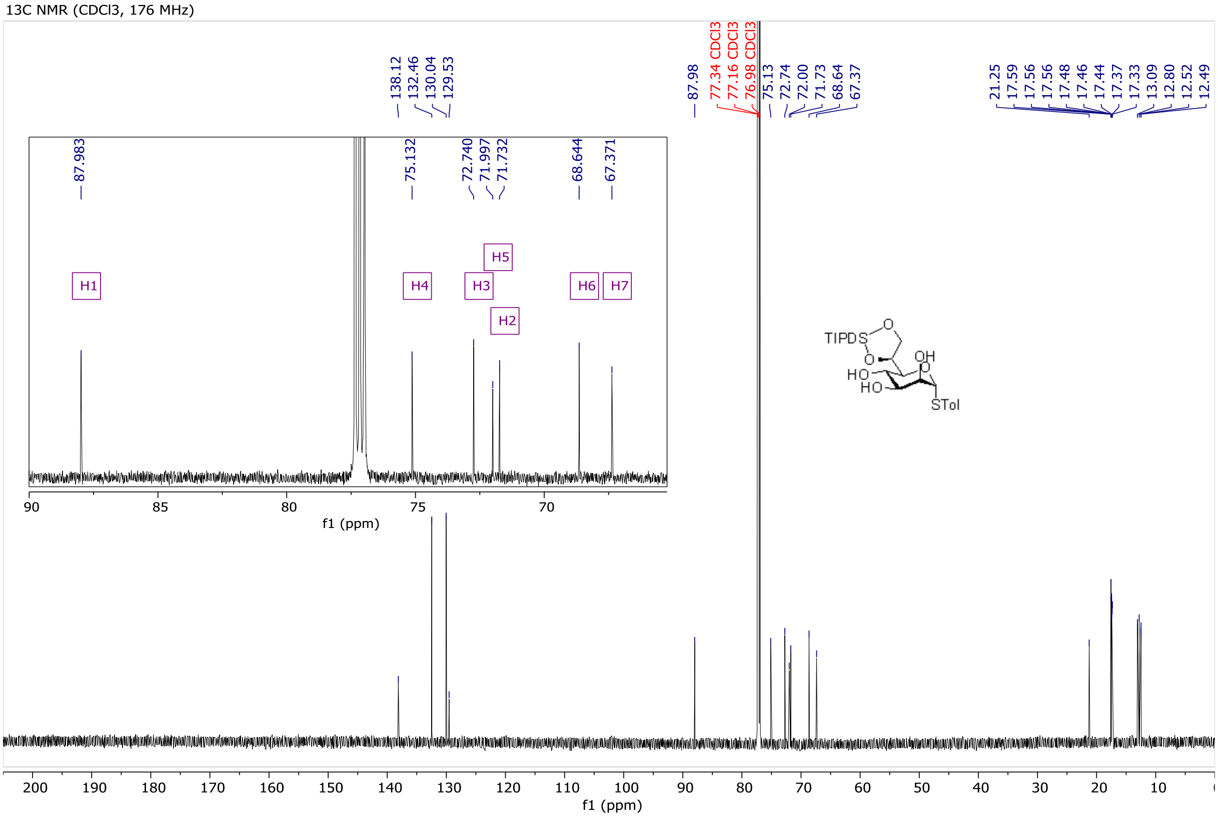

C.1.4. Tol-4-yl 2,3,4,6,7-penta-*O*-acetyl-1-thio-D-*glycero*-α-D-*manno*-heptopyranoside (6)

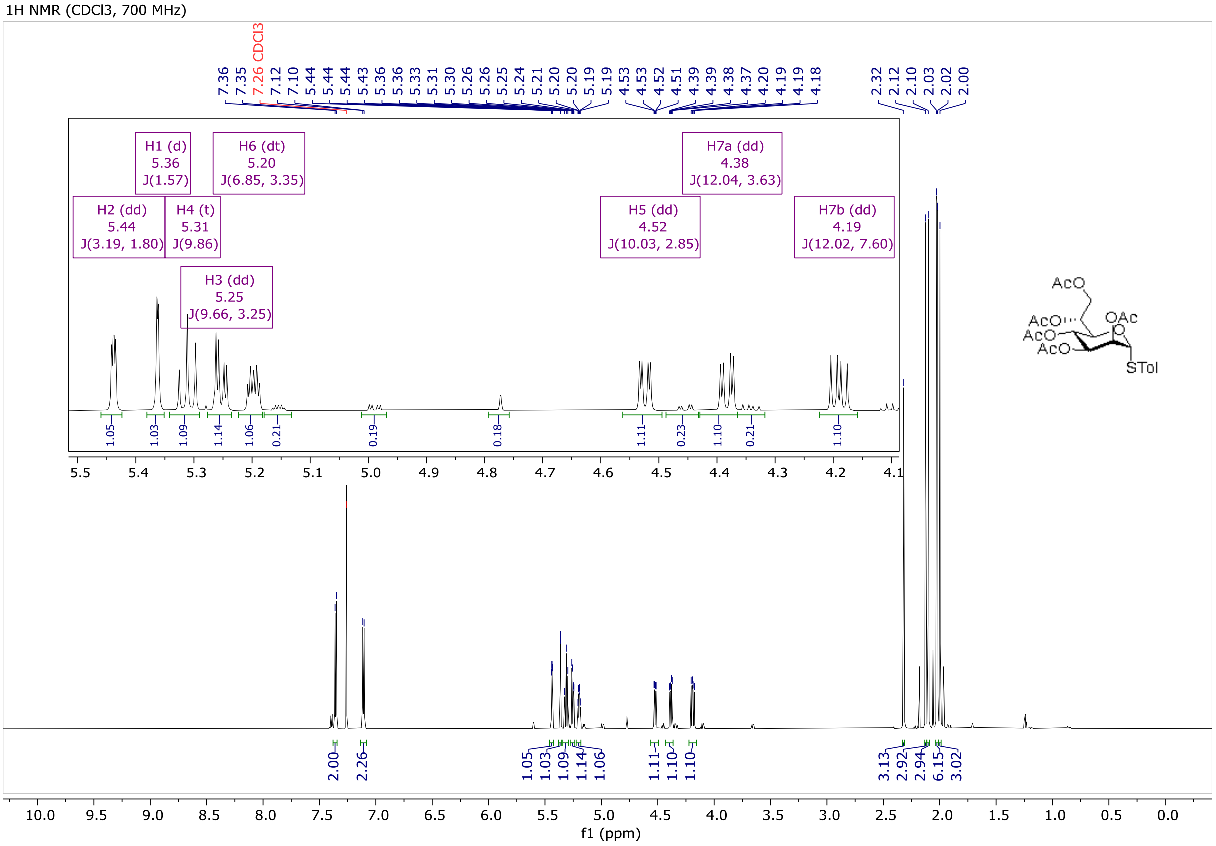

13C NMR (CDCl3, 176 MHz)

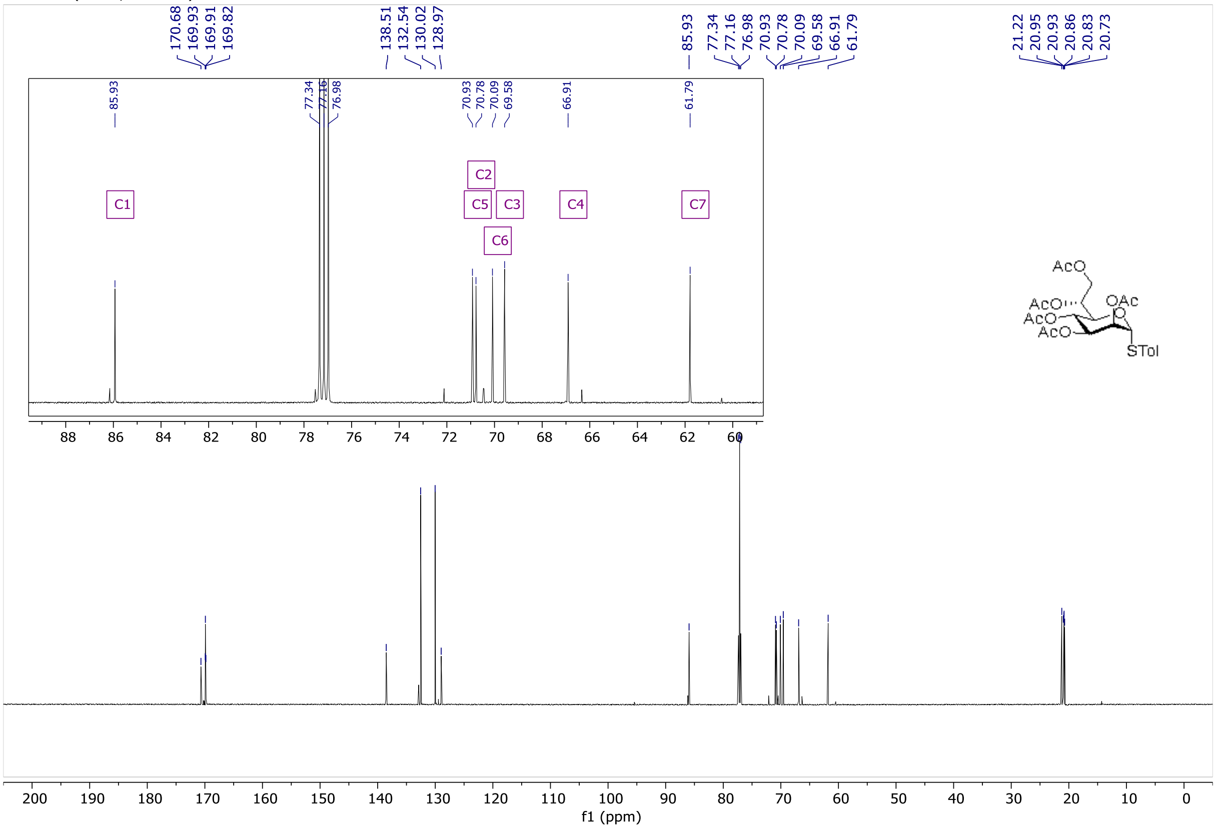

C.1.5. Tol-4-yl 1-thio-D-*glycero*-α-D-*manno*-heptopyranoside (8)

1H NMR (MeOD, 700 MHz)

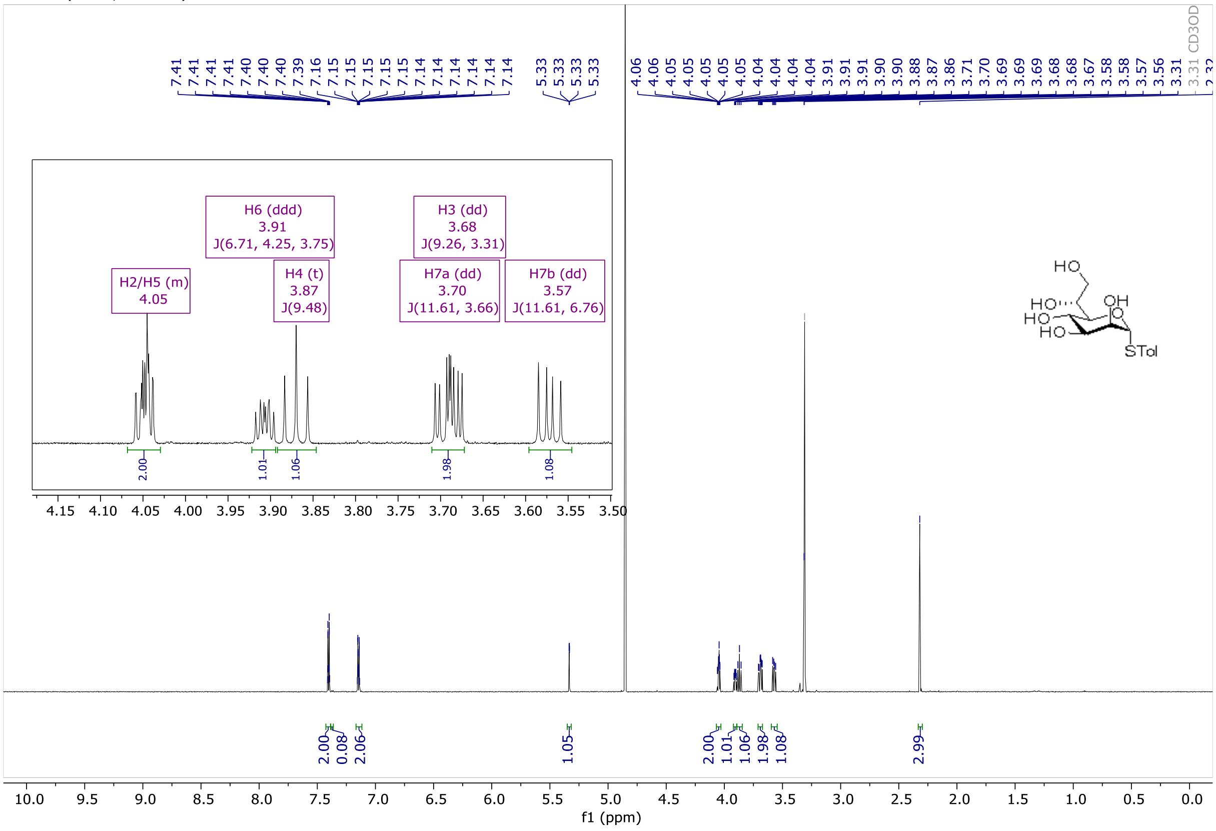

13C NMR (MeOD, 176 MHz)

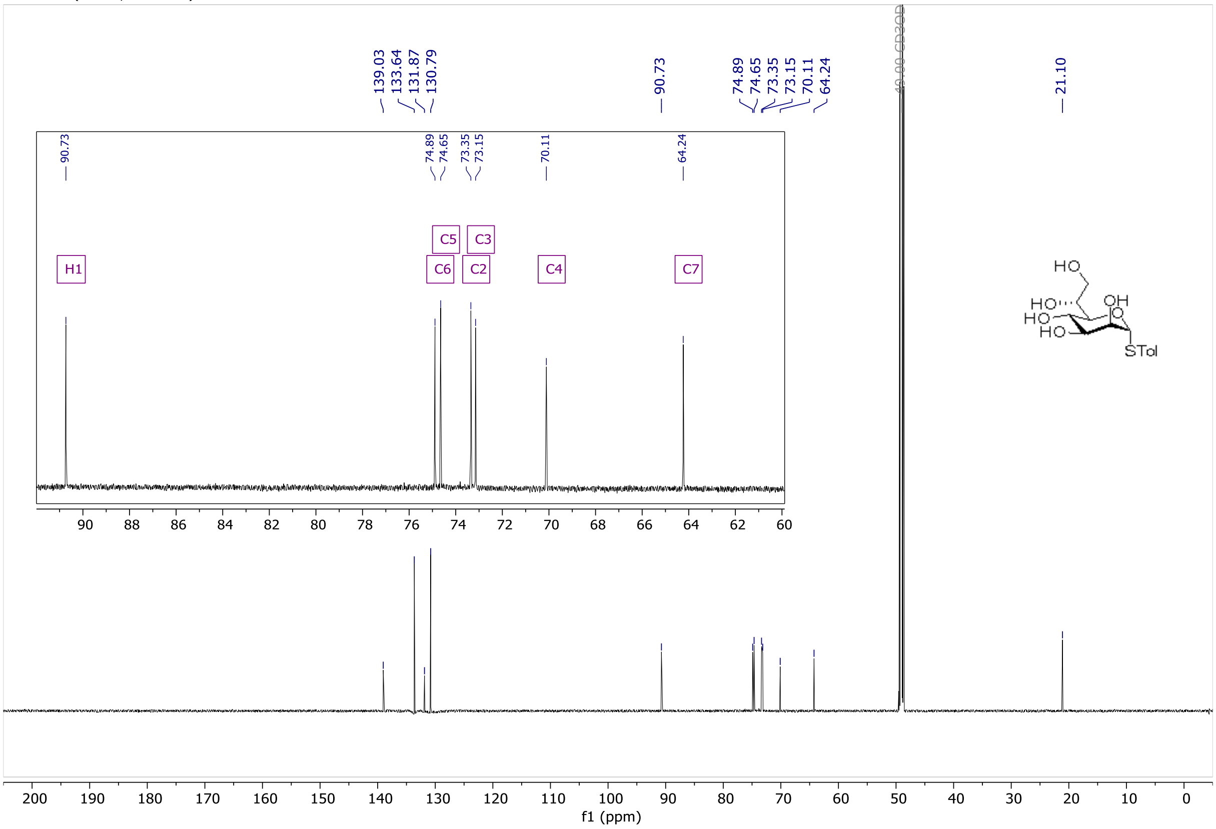

C.1.6. Tol-4-yl 6,7-*O*-(1,1,3,3-tetraisopropyl-1,3-disiloxane-1,3-diyl)-1-thio-D-*glycero*-α-D-*manno*-heptopyranoside (4)

1H NMR (CDCl3, 400 MHz)

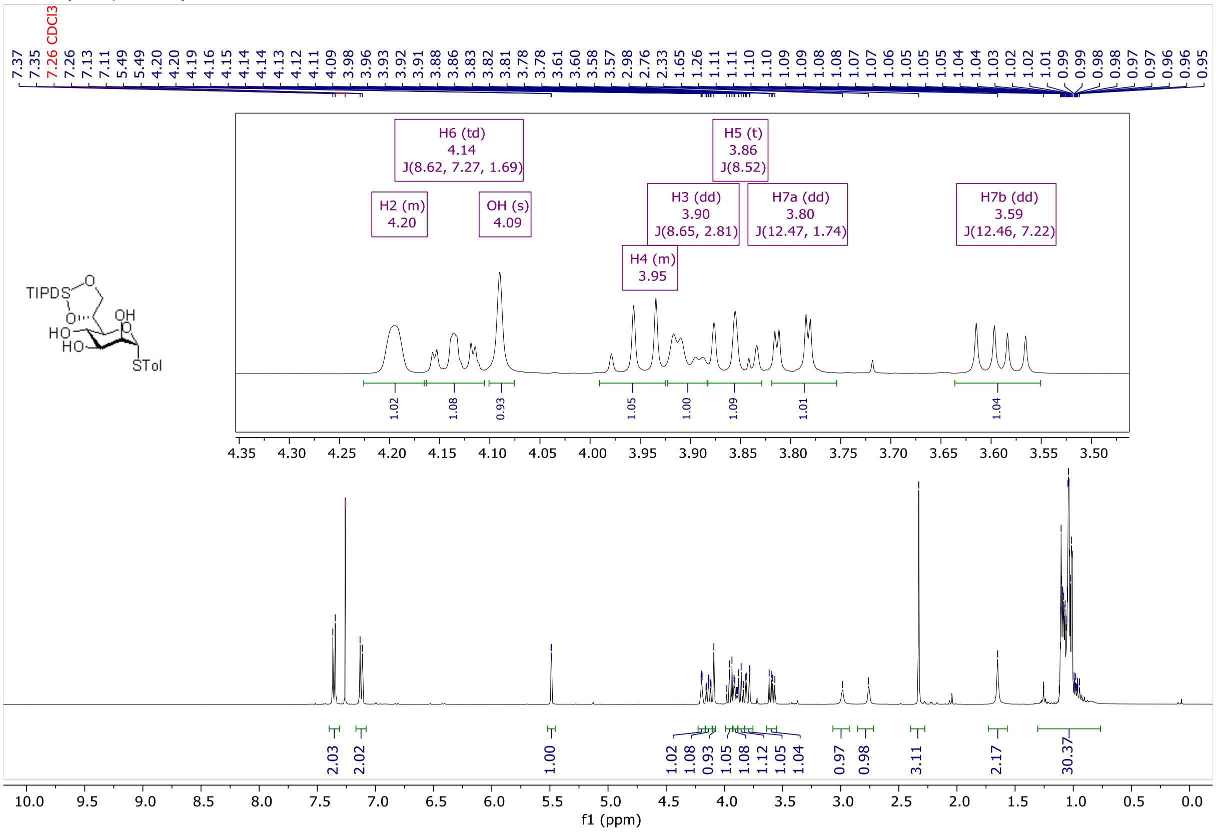

13C NMR (CDCl3, 101 MHz)

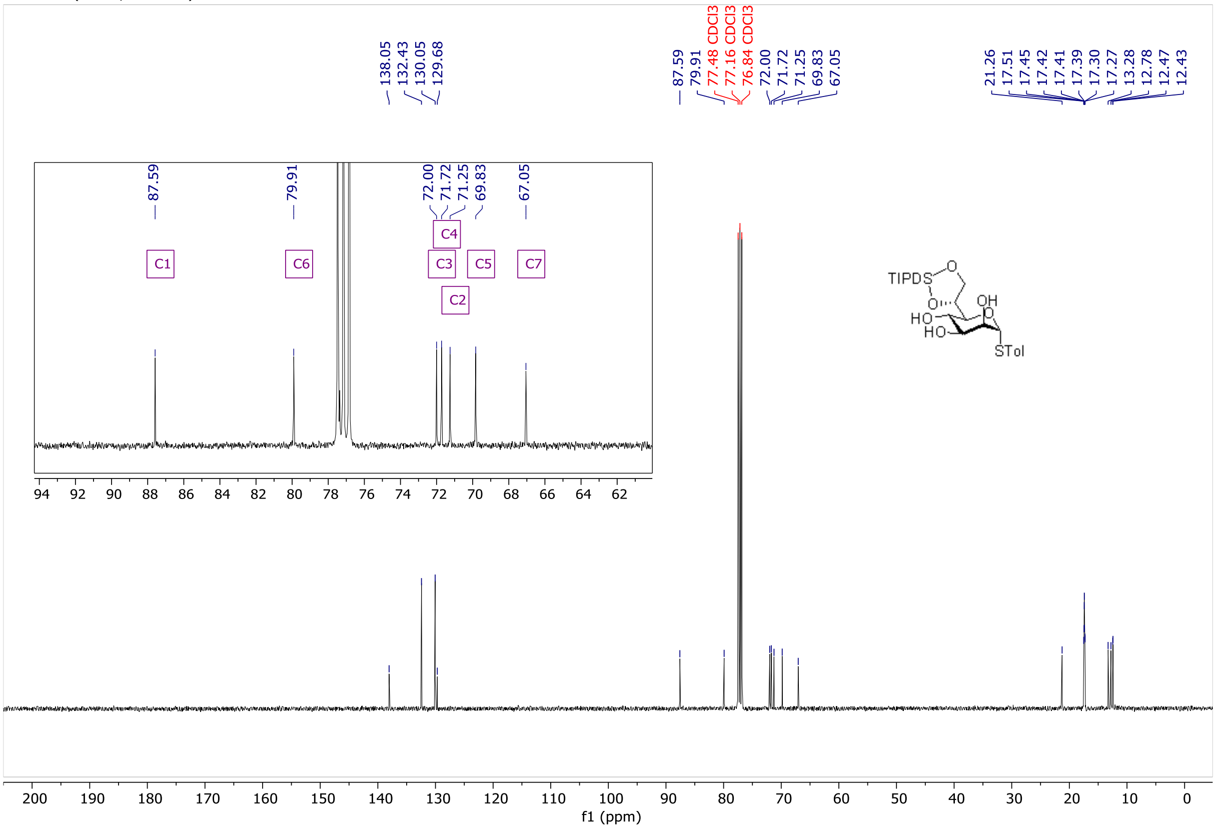

C.2. Fully Decorated TIPDS sugars

C.2.1. Methyl 2,3-di-*O*-acetyl-4-*O*-chloroacetyl-6,7-*O*-(1,1,3,3-tetraisopropyl-1,3-disiloxane-1,3-diyl)-L-*glycero*-α-D-*manno*-heptopyranoside (10)

1H NMR (CDCl3, 400 MHz)

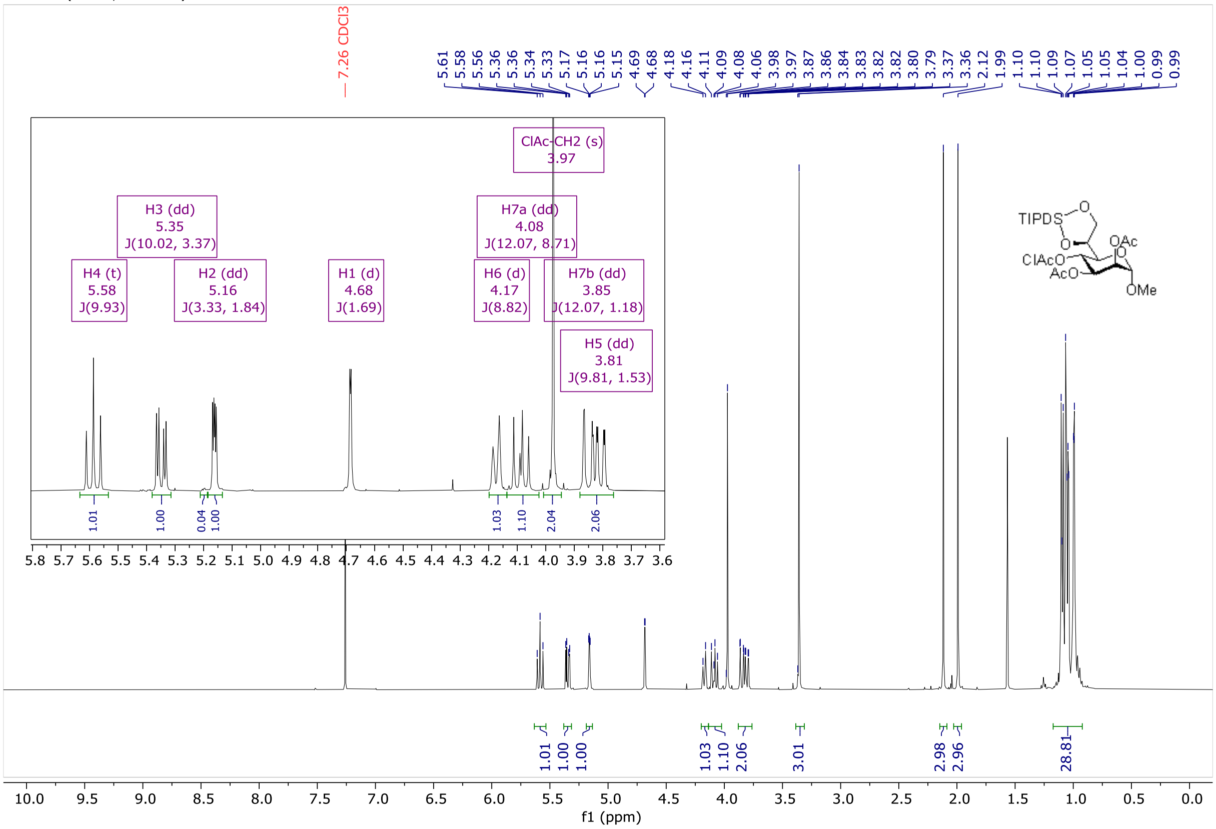

13C NMR (CDCl3, 176 MHz)

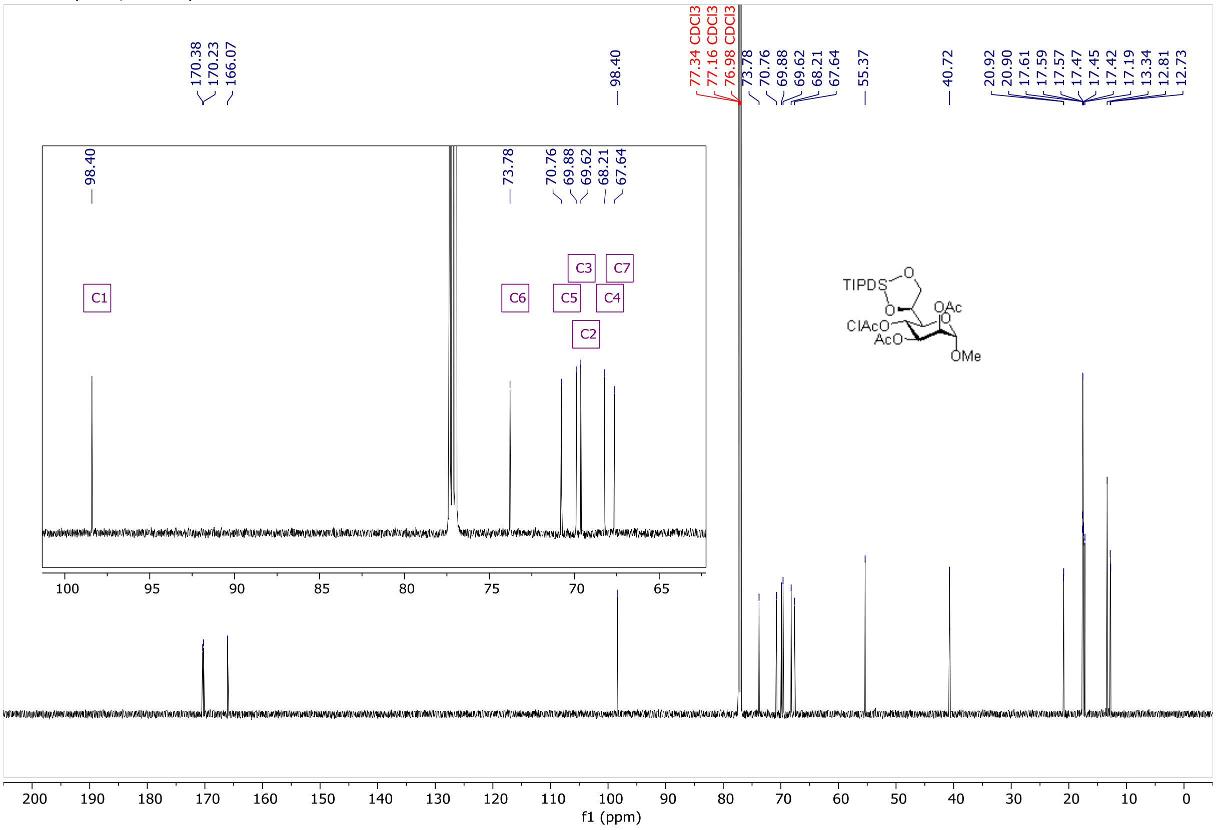

C.2.2. Methyl 2,4-di-*O*-benzoyl-3-*O*-chloroacetyl-6,7-*O*-(1,1,3,3-tetraisopropyl-1,3-disiloxane-1,3-diyl)-L-*glycero*-α-D-*manno*-heptopyranoside (11)

1H NMR (CDCl3, 400.13 MHz)

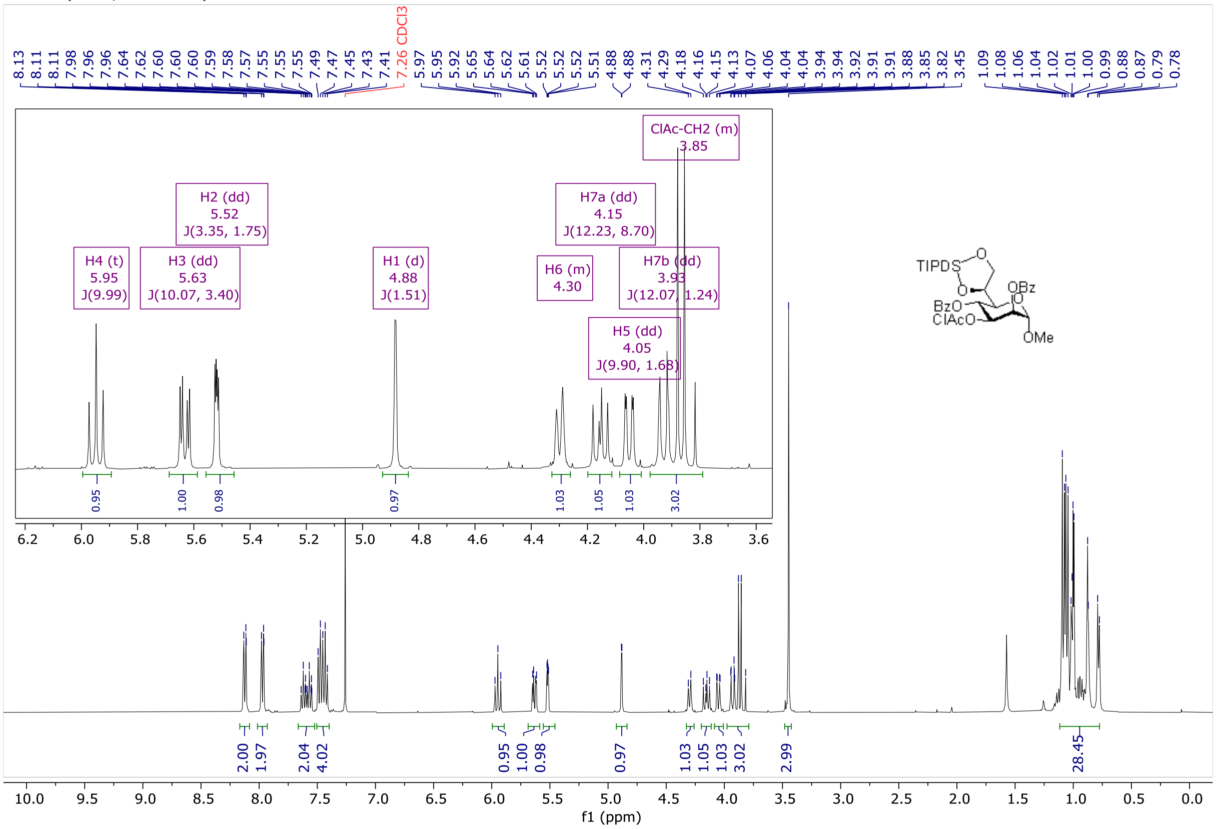

13C NMR (CDCl3, 176 MHz)

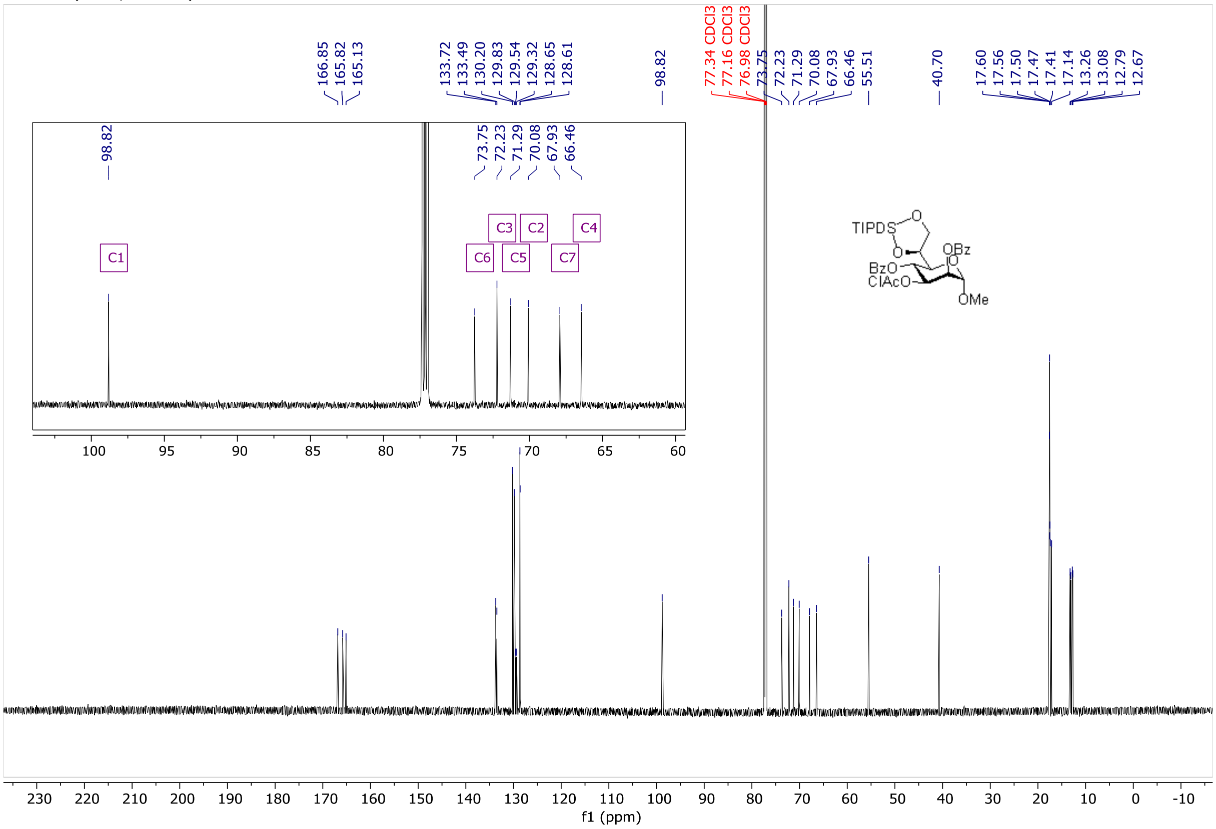

C.2.3. Tol-4-yl 2,3-di-*O*-benzoyl-4-*O*-chloroacetyl-6,7-*O*-(1,1,3,3-tetraisopropyl-1,3-disiloxane-1,3-diyl)-1-thio-L-*glycero*-α-D-*manno*-heptopyranoside (12)

1H NMR (CDCl3, 400 MHz)

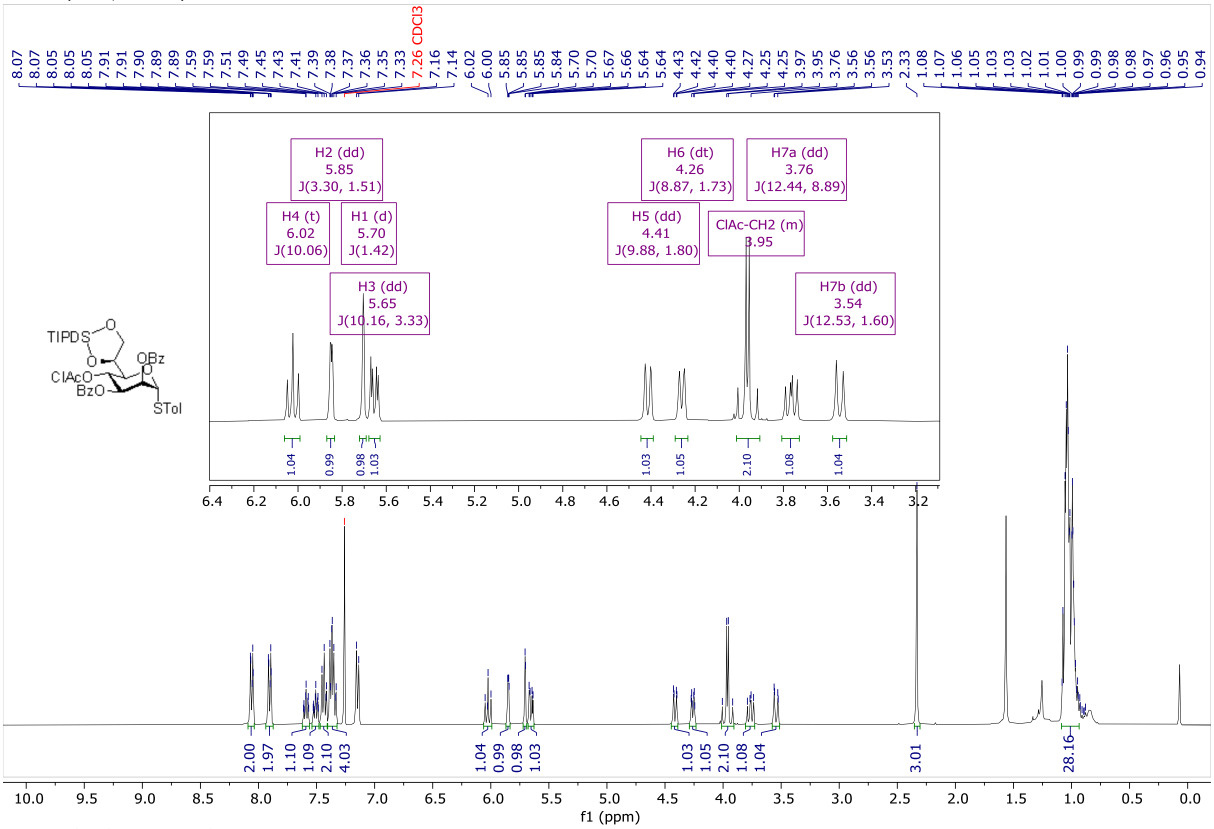

13C NMR (CDCl3, 100.62 MHz)

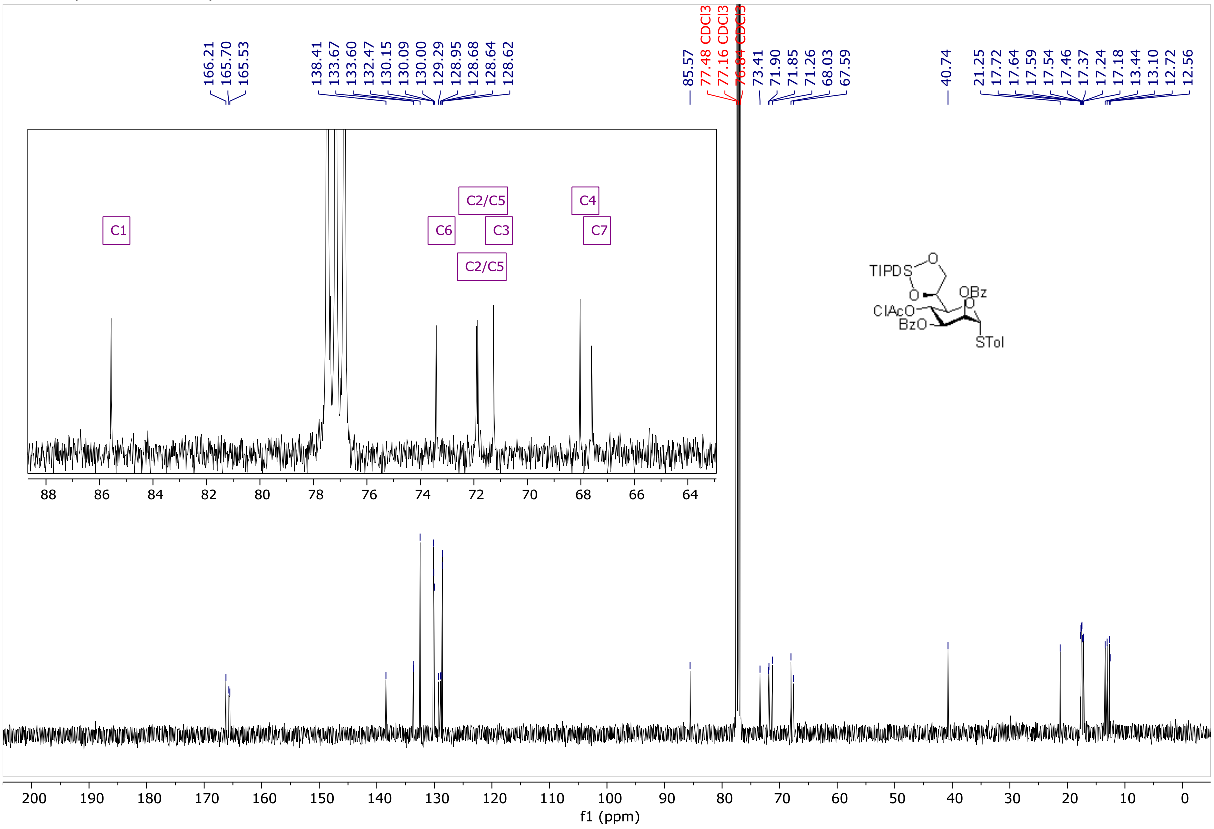

C.2.4. Tol-4-yl 2,4-di-*O*-benzoyl-3-*O*-chloroacetyl-6,7-*O*-(1,1,3,3-tetraisopropyl-1,3-disiloxane-1,3-diyl)-1-thio-L-*glycero*-α-D-*manno*-heptopyranoside (13)

1H NMR (CDCl3, 400 MHz)

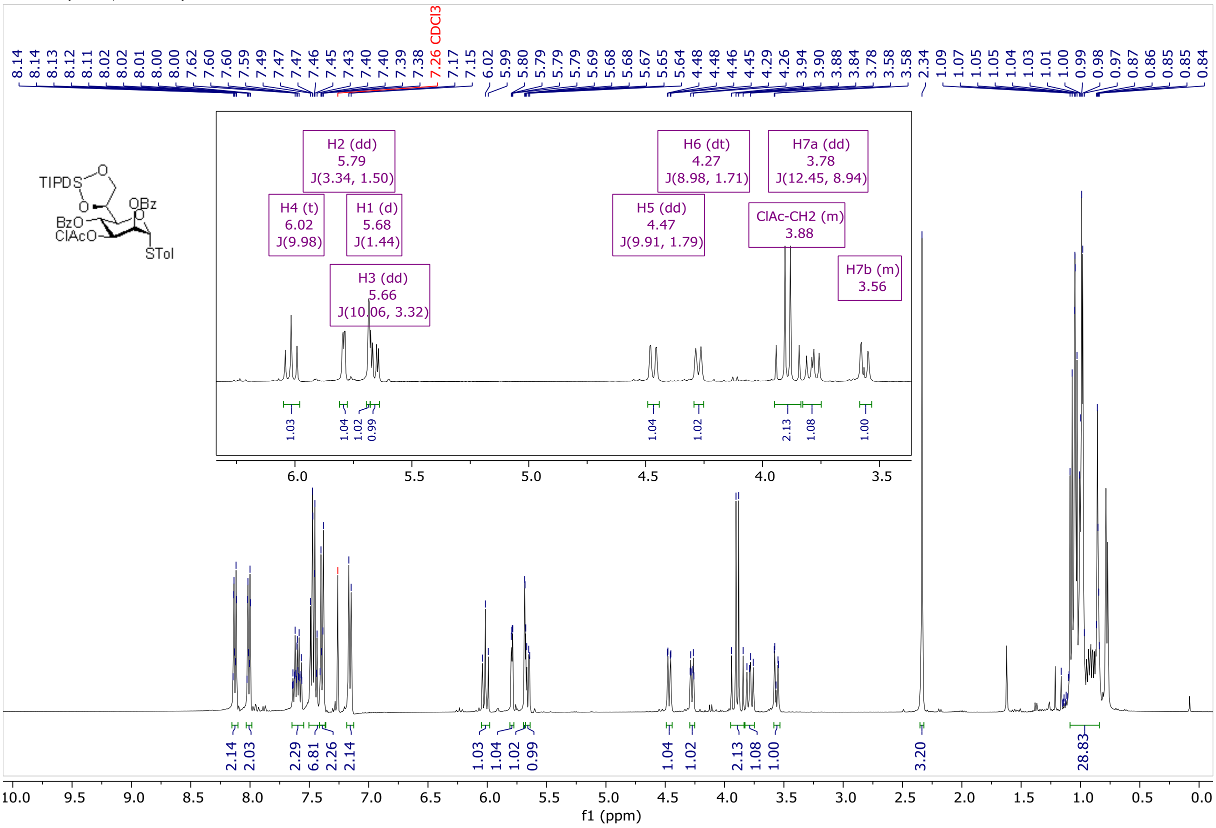

13C NMR (CDCl3, 101 MHz)

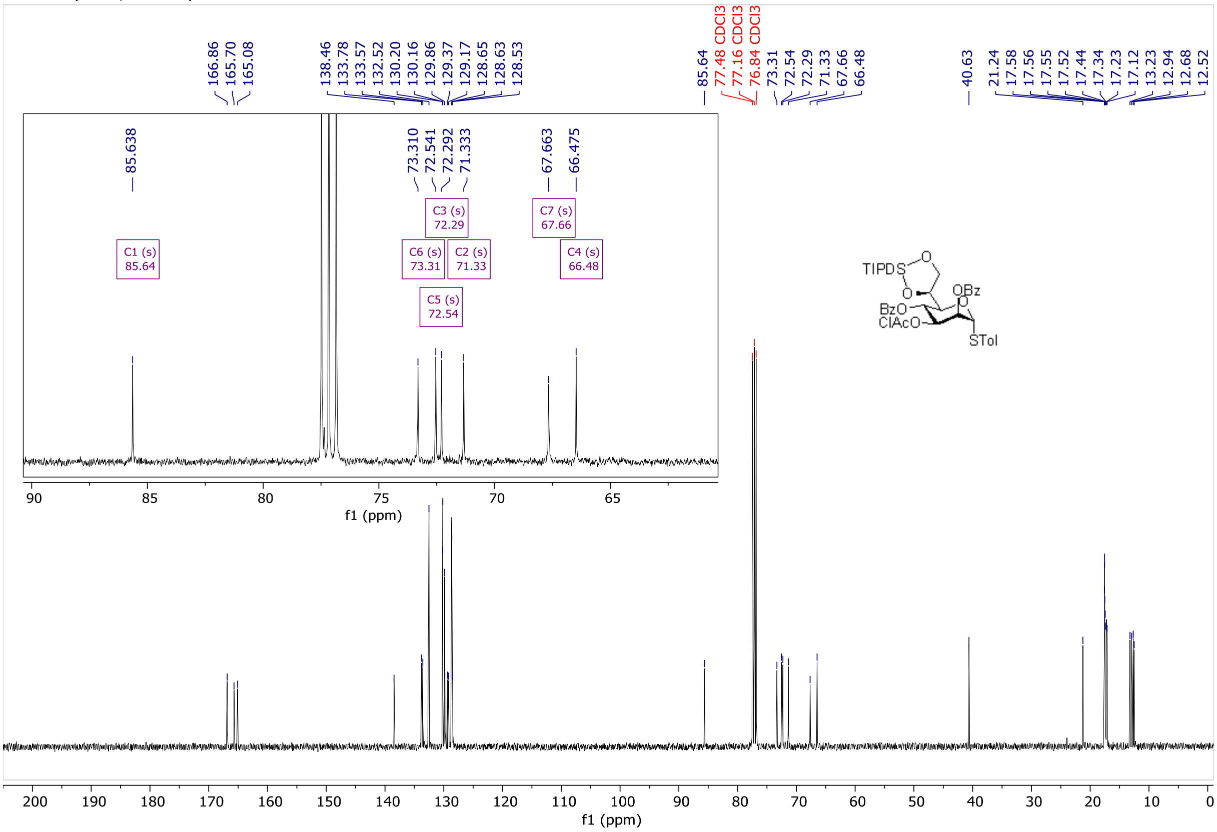

COSY (CDCl3, 400.13 MHz)

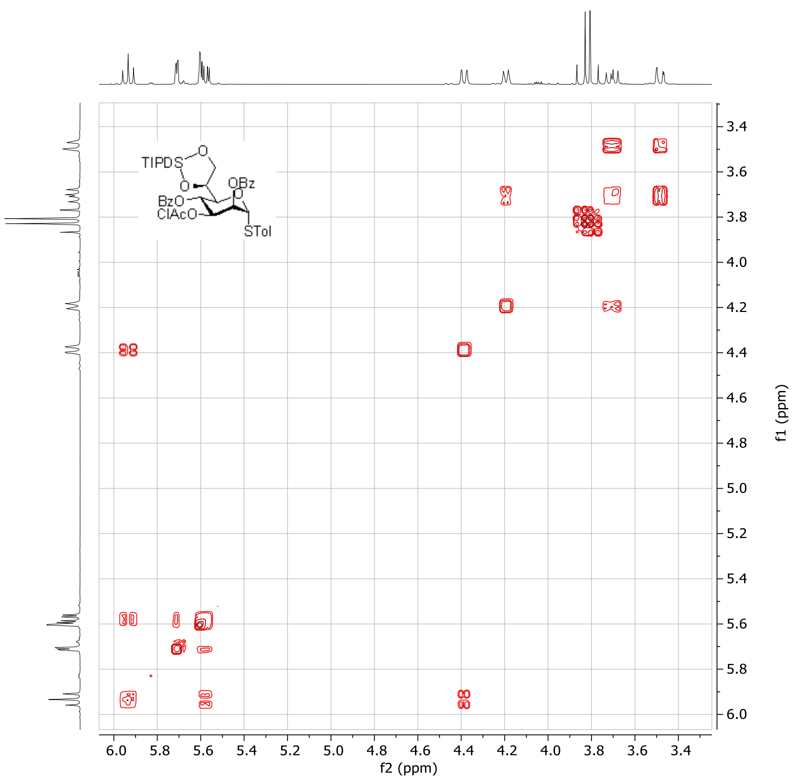

HSQC (CDCl3, 400 MHz)

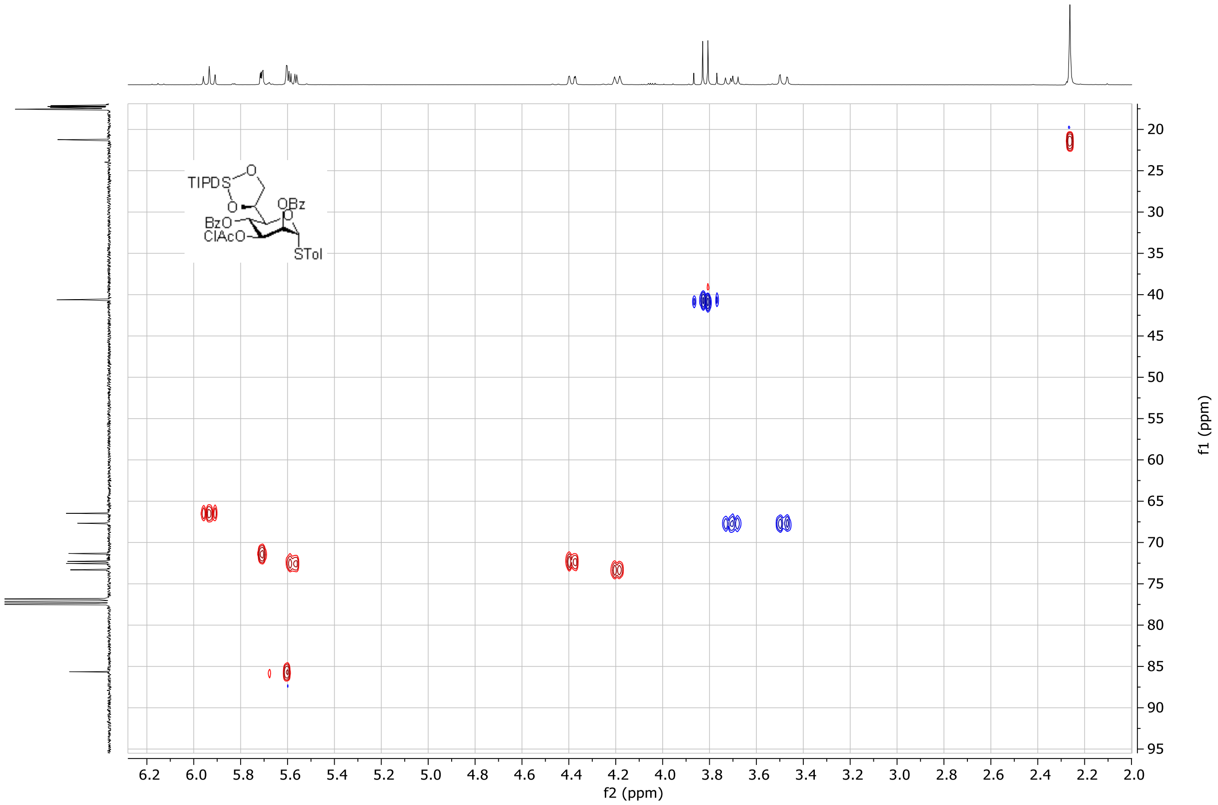

C.2.5. Tol-4-yl 2,3-*O*-(2-chloro-1-ethoxyethyliden-1,1-diyl)-6,7-*O*-(1,1,3,3-tetraisopropyl-1,3-disiloxane-1,3-diyl)-1-thio-L-*glycero*-α-D-*manno*-heptopyranoside – Isomere A (14a)

1H NMR (CDCl3, 400 MHz)

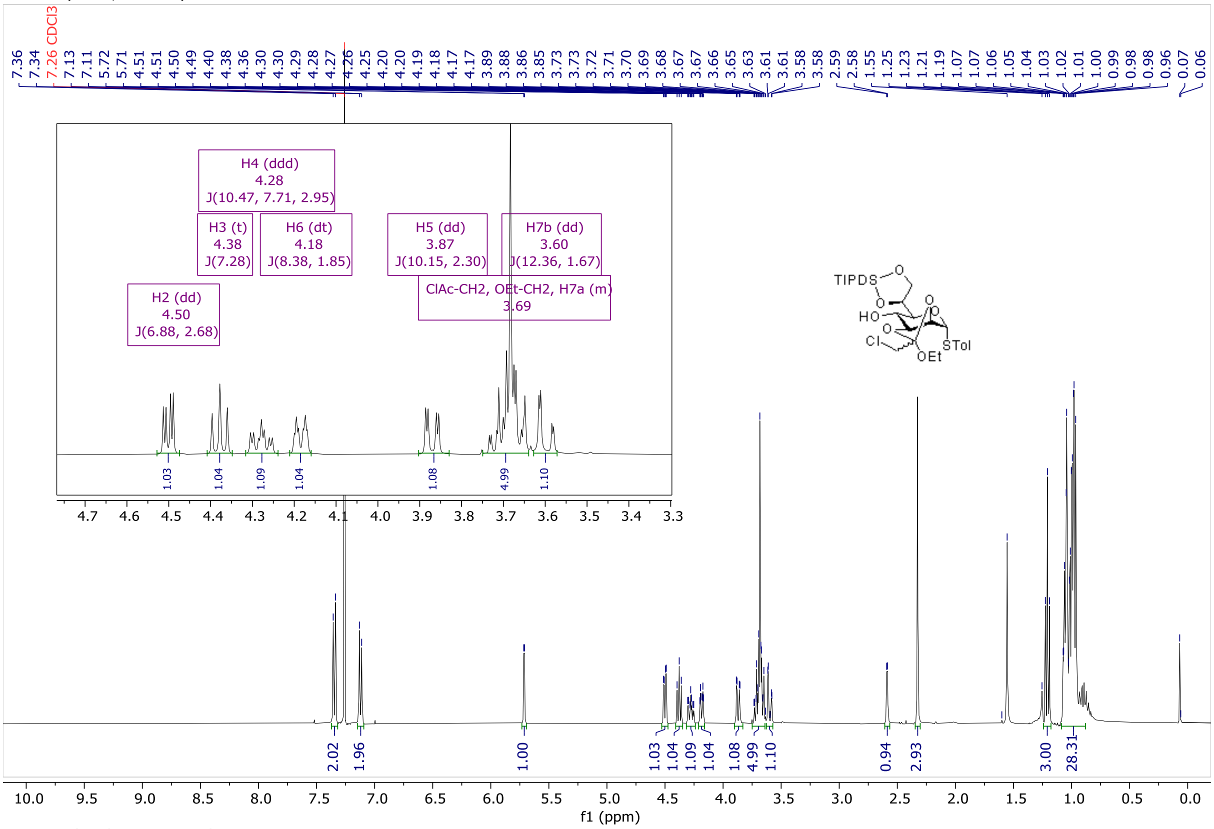

13C NMR (CDCl3, 100.62 MHz)

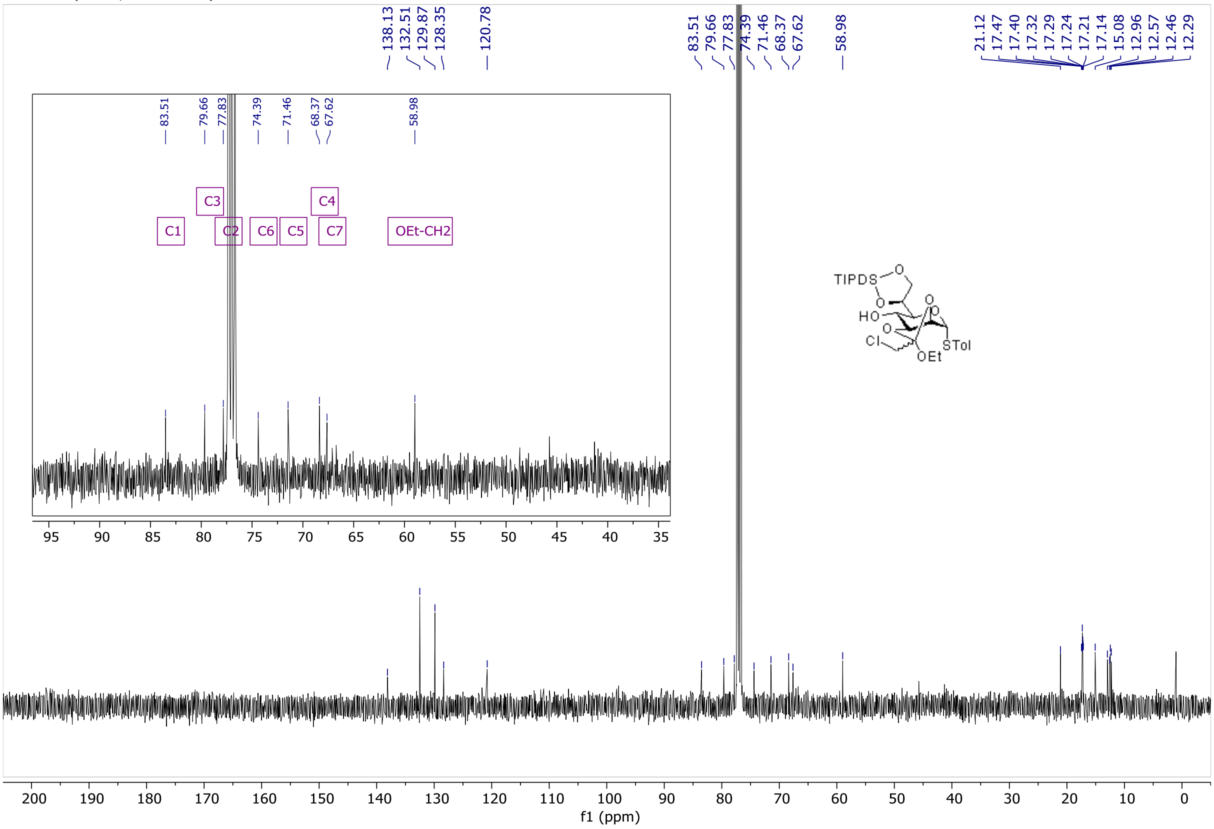

C.2.6. Tol-4-yl 2,3-*O*-(2-chloro-1-ethoxyethyliden-1,1-diyl)-6,7-*O*-(1,1,3,3-tetraisopropyl-1,3-disiloxane-1,3-diyl)-1-thio-L-*glycero*-α-D-*manno*-heptopyranoside – Isomere B (14b)

1H NMR (CDCl3, 400 MHz)

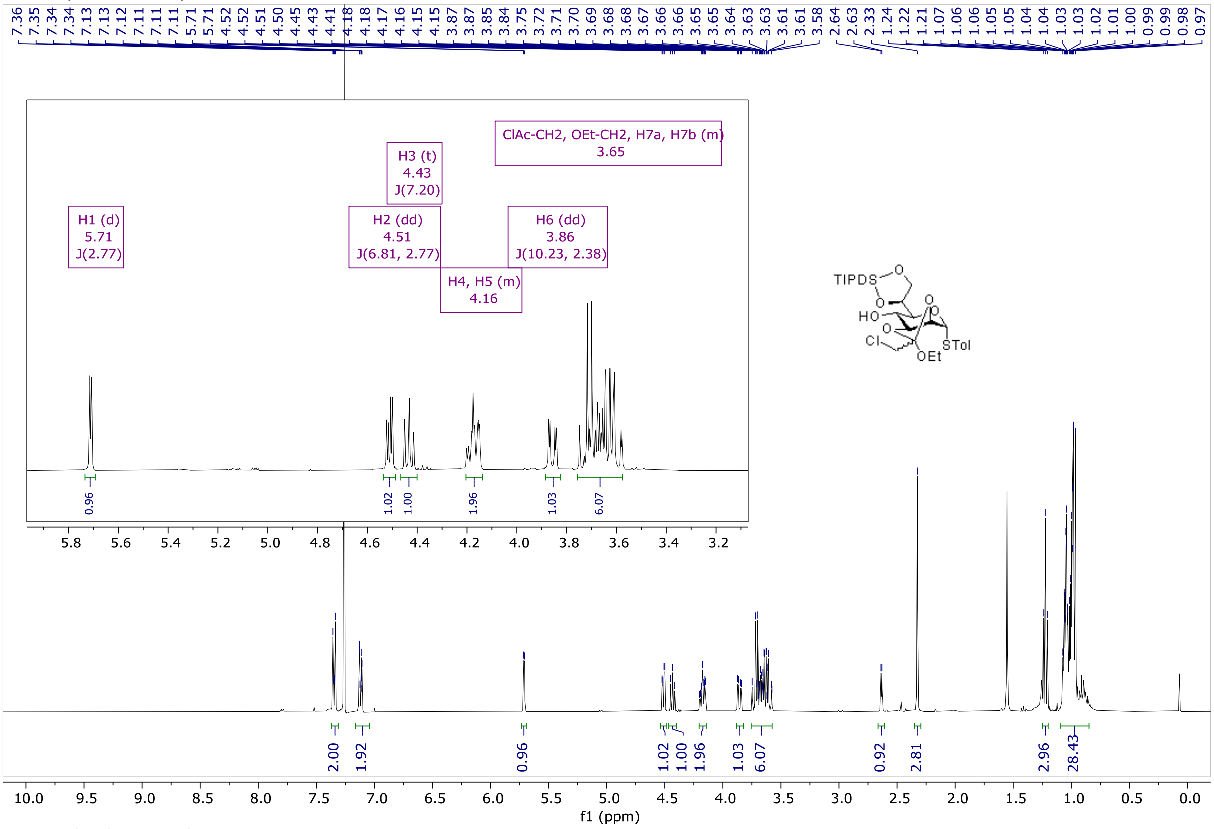

13C NMR (CDCl3, 101 MHz)

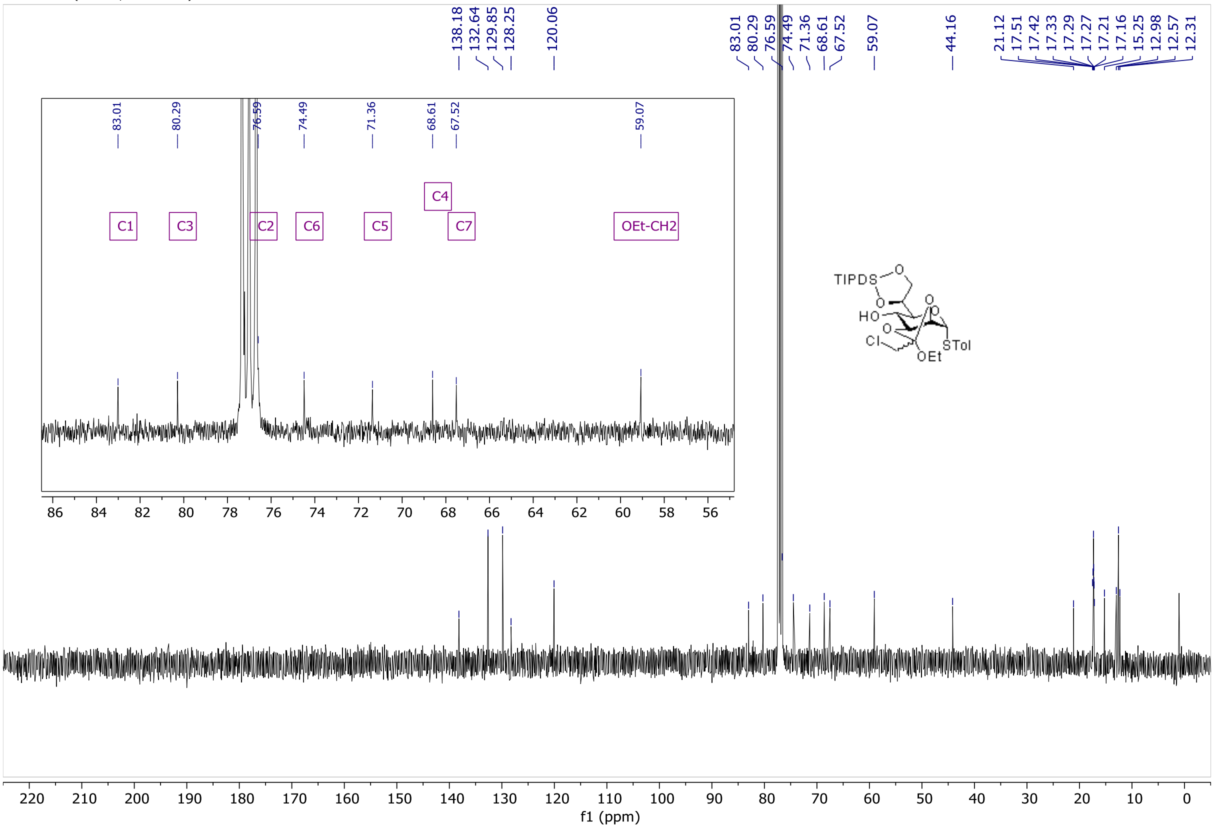

C.2.7. Tol-4-yl 4-*O*-benzoyl-2,3-*O*-(2-chloro-1-ethoxyethyliden-1,1-diyl)-6,7-*O*-(1,1,3,3-tetraisopropyl-1,3-disiloxane-1,3-diyl)-1-thio-L-*glycero*-α-D-*manno*-heptopyranoside – Isomere A (15a)

1H NMR (CDCl3, 600 MHz)

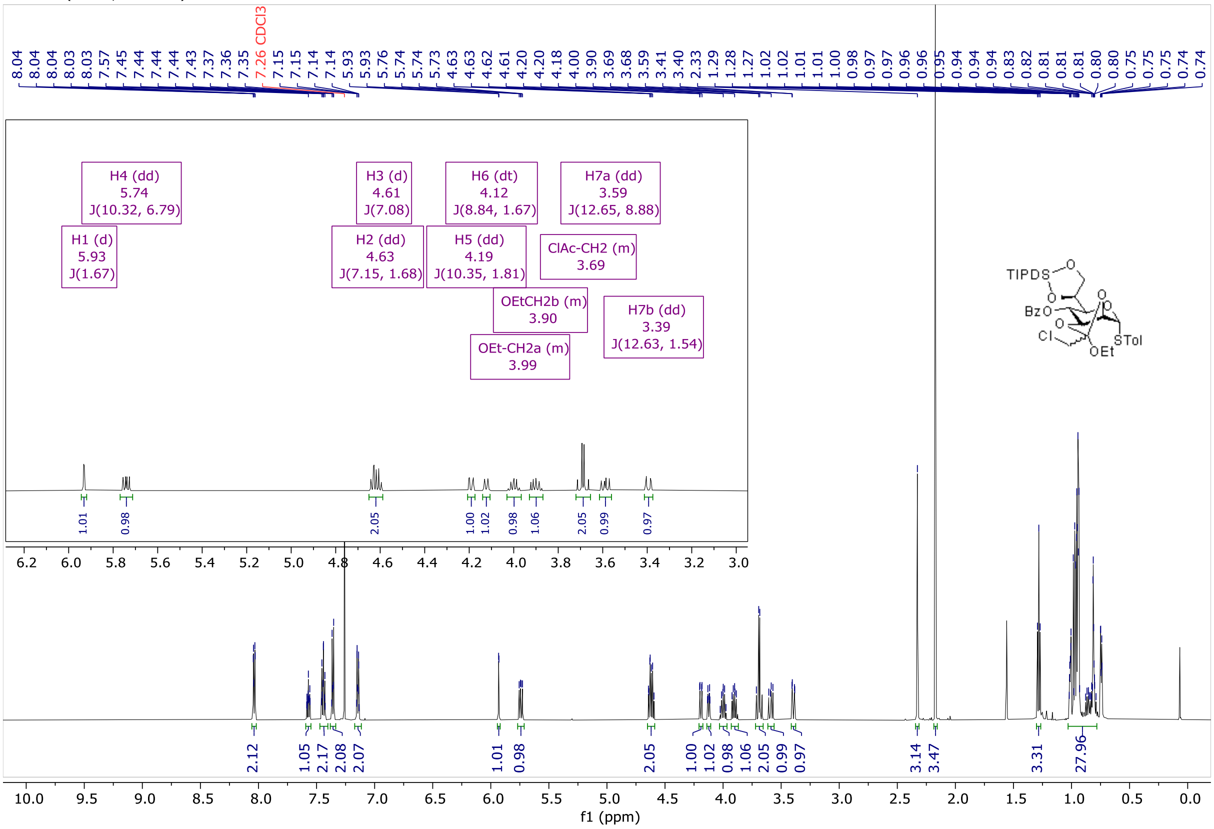

13C NMR (CDCl3, 151 MHz)

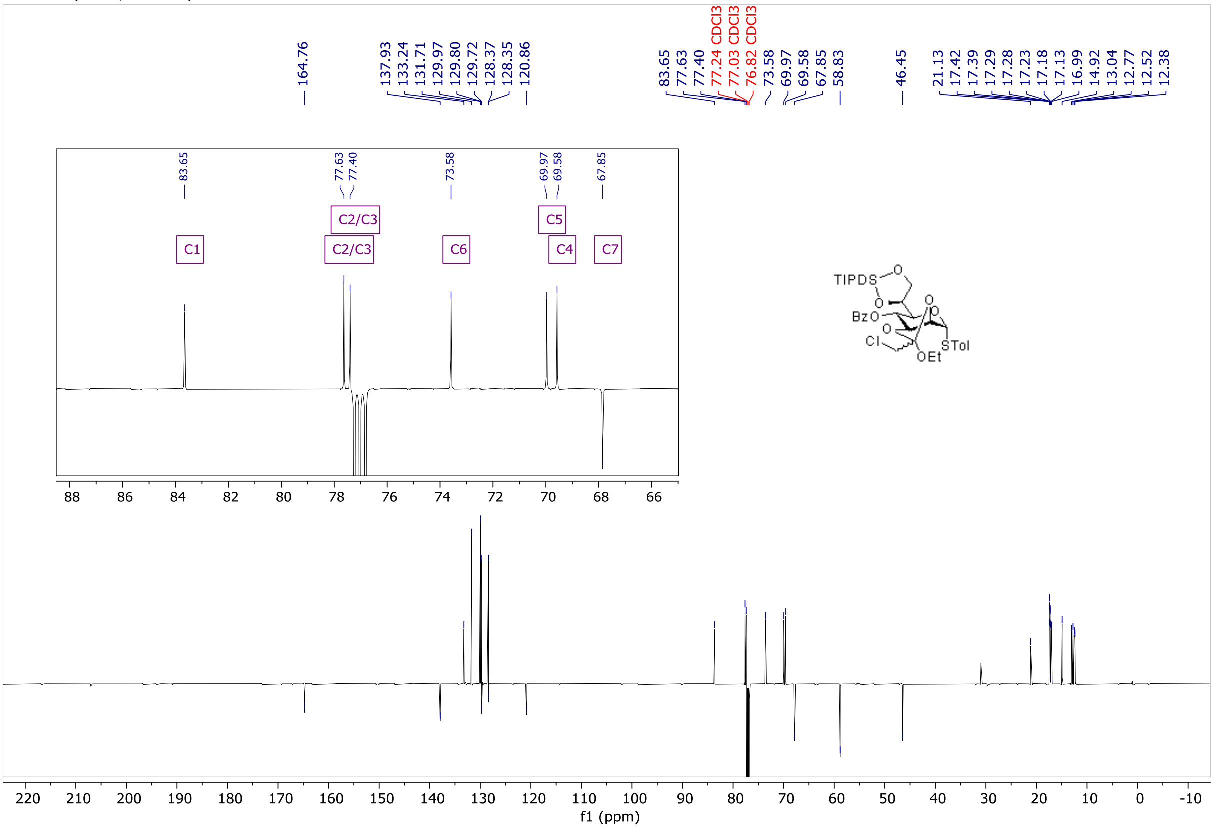

COSY (CDCl3, 600 MHz)

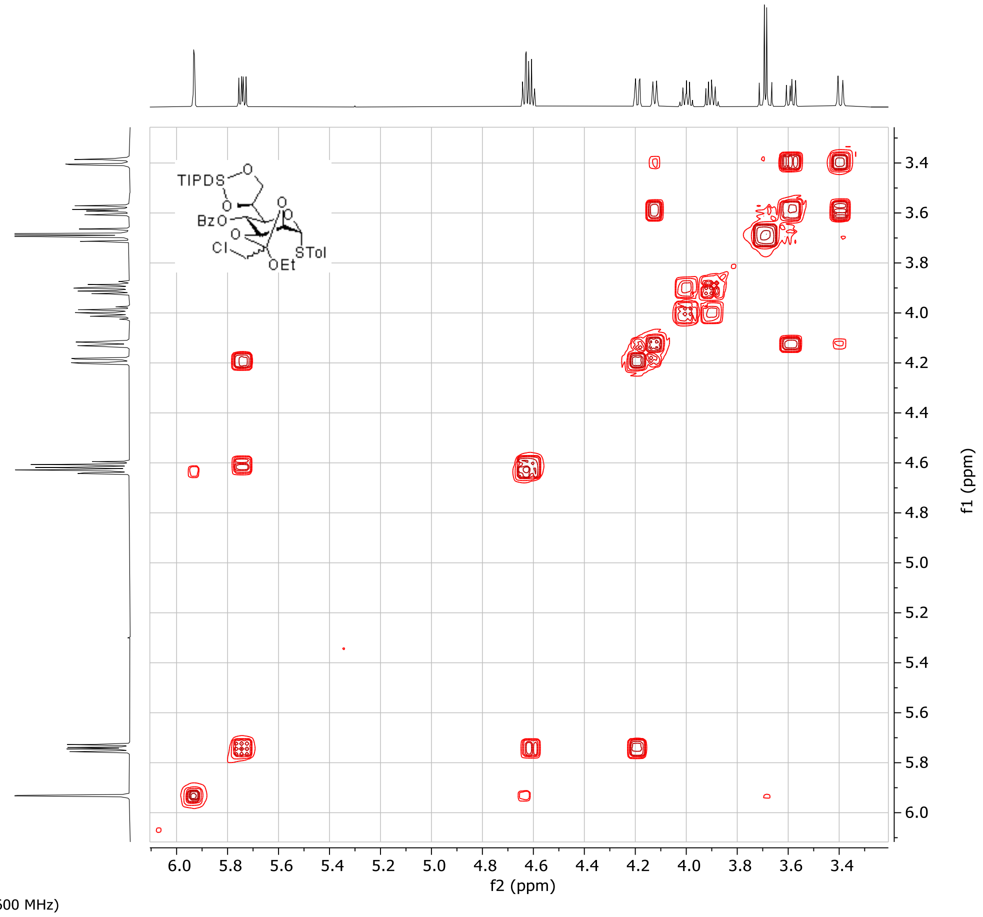

HSQC (CDCl3, 600 MHz)

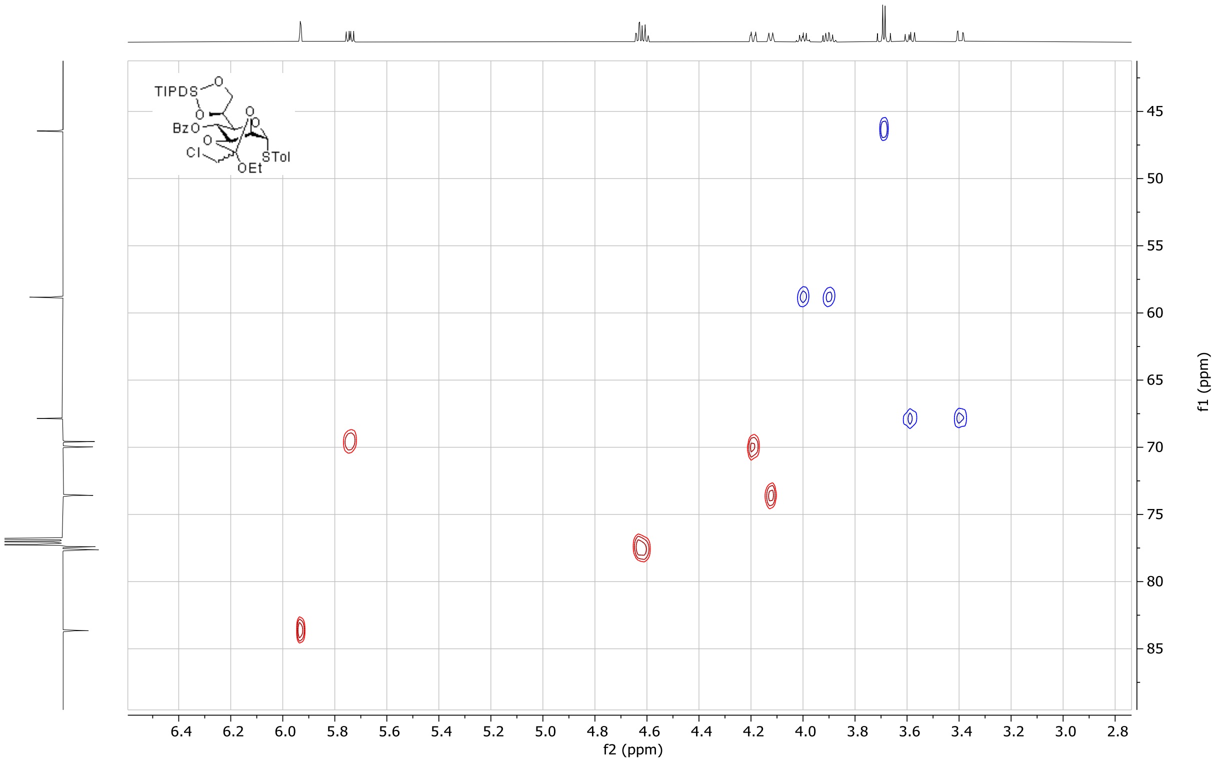

C.2.8. Tol-4-yl 4-*O*-benzoyl-2,3-*O*-(2-chloro-1-ethoxyethyliden-1,1-diyl)-6,7-*O*-(1,1,3,3-tetraisopropyl-1,3-disiloxane-1,3-diyl)-1-thio-L-*glycero*-α-D-*manno*-heptopyranoside – Isomere B (15b)

1H NMR (CDCl3, 600 MHz)

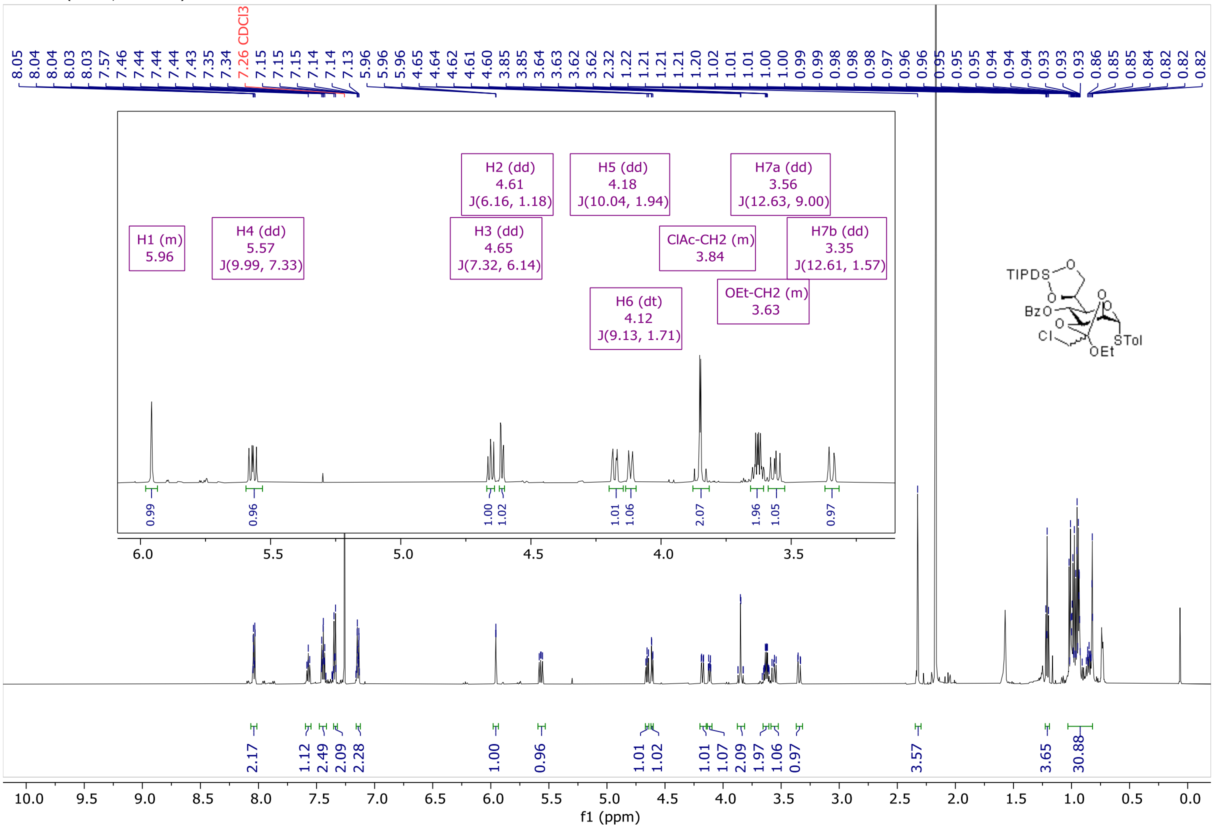

13C NMR (CDCl3, 151 MHz)

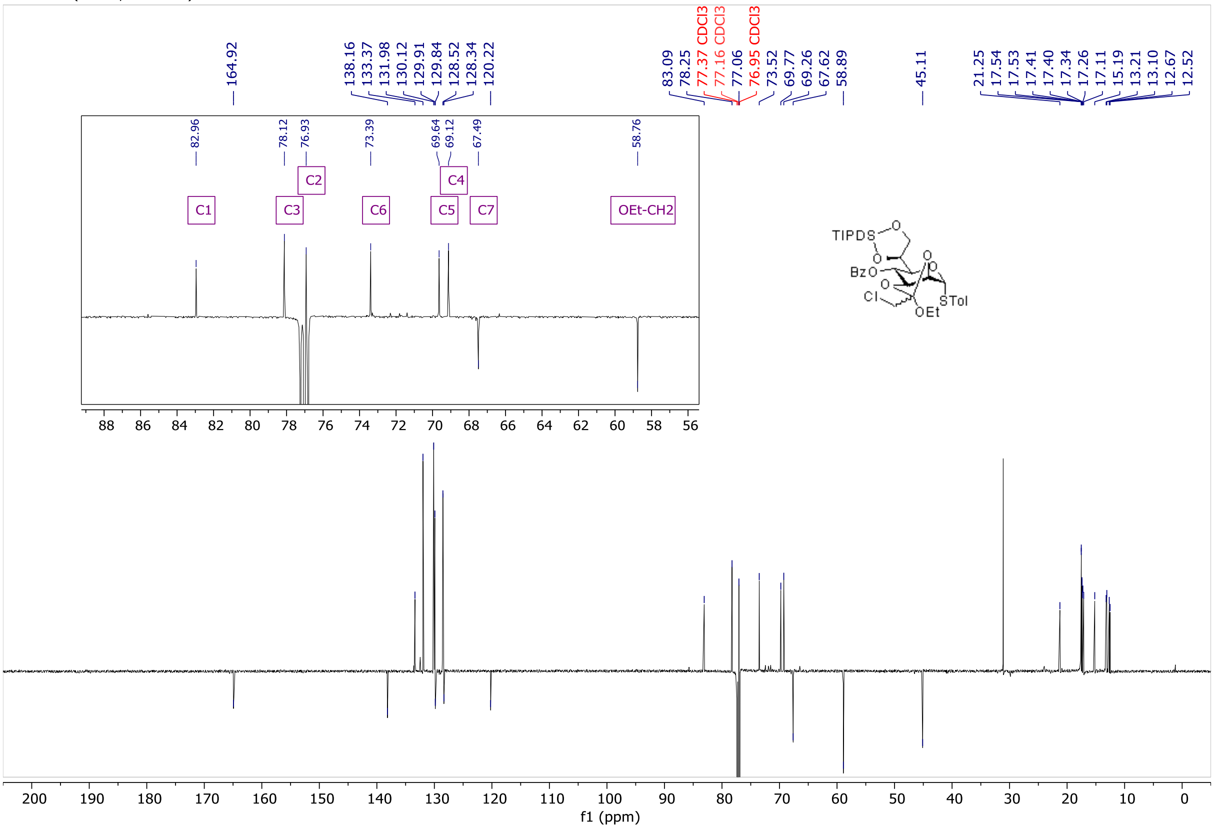

COSY (CDCl3, 600 MHz)

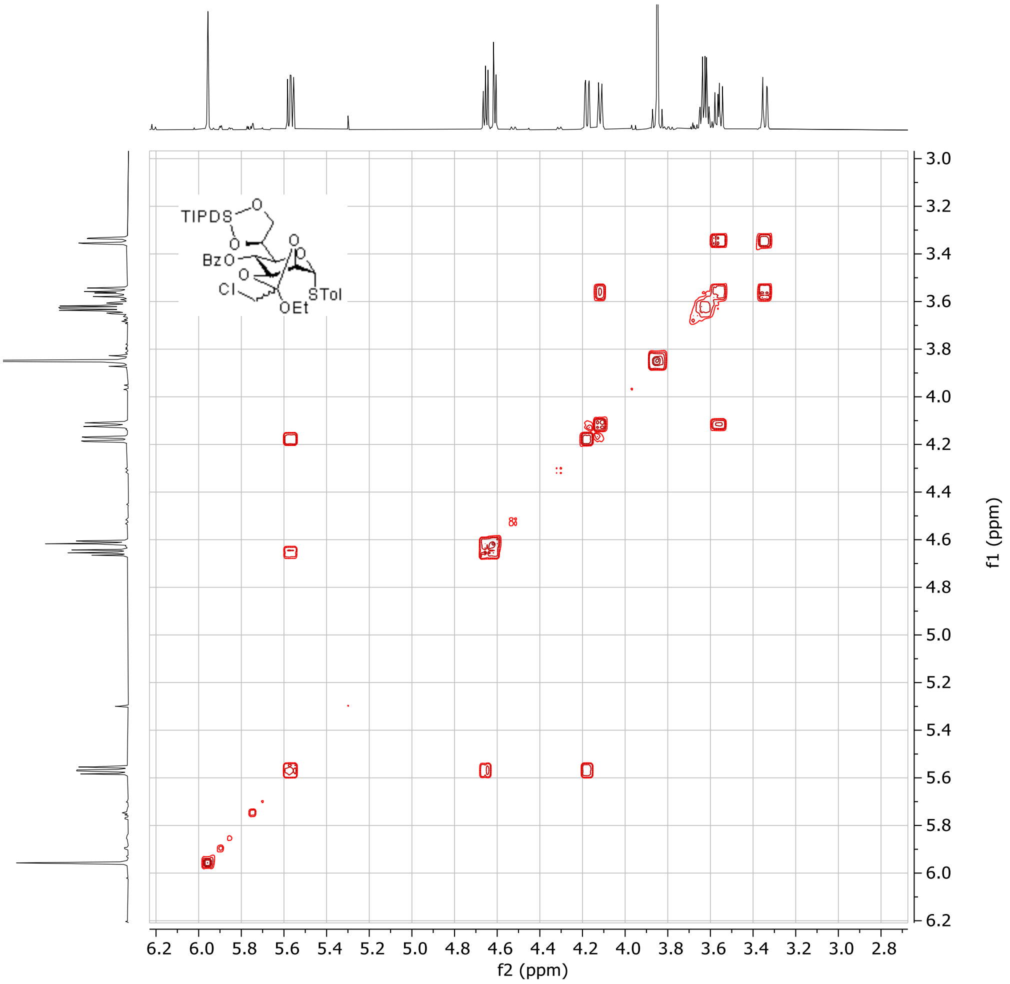

HSQC (CDCl3, 600 MHz)

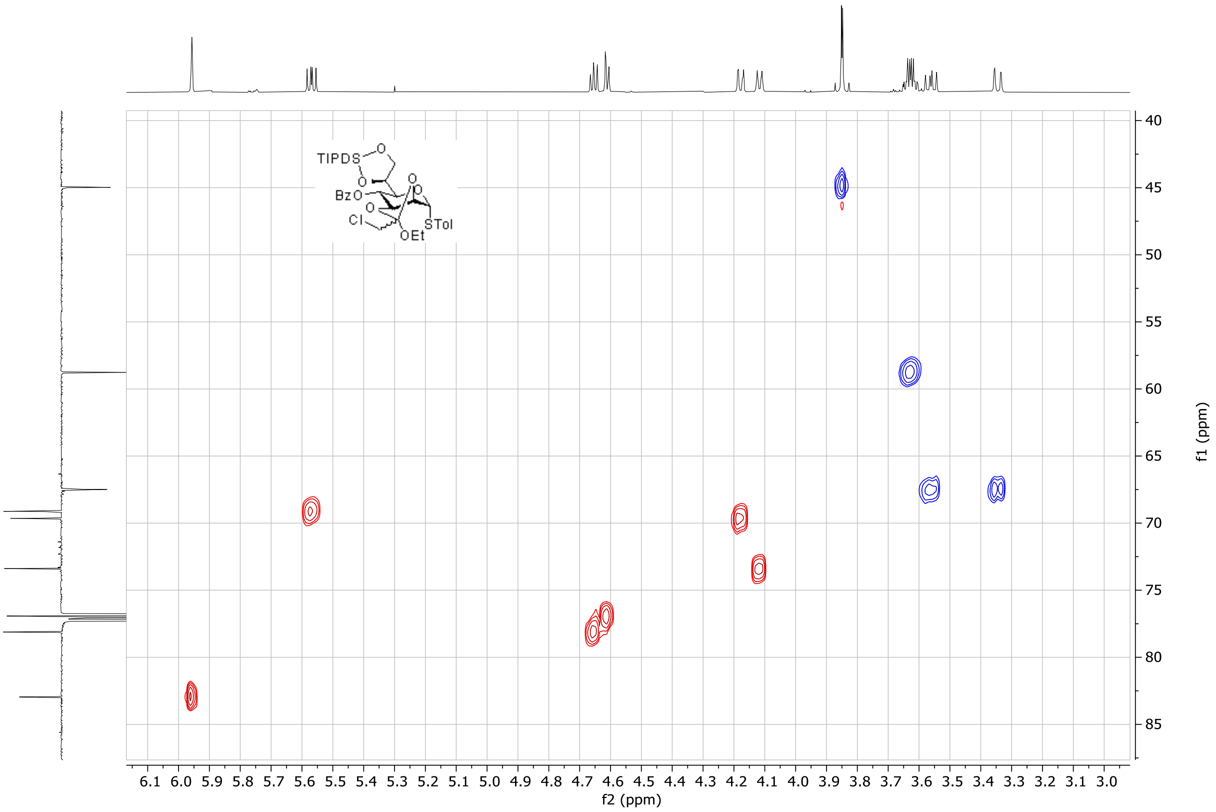

C.2.9. Tol-4-yl 2,3-di-*O*-benzoyl-4-*O*-chloroacetyl-6,7-*O*-(1,1,3,3-tetraisopropyl-1,3-disiloxan-1,3-diyl)-1-thio-D-*glycero*-α-D-*manno*-heptopyranoside (16)

1H NMR (CDCl3, 400 MHz)

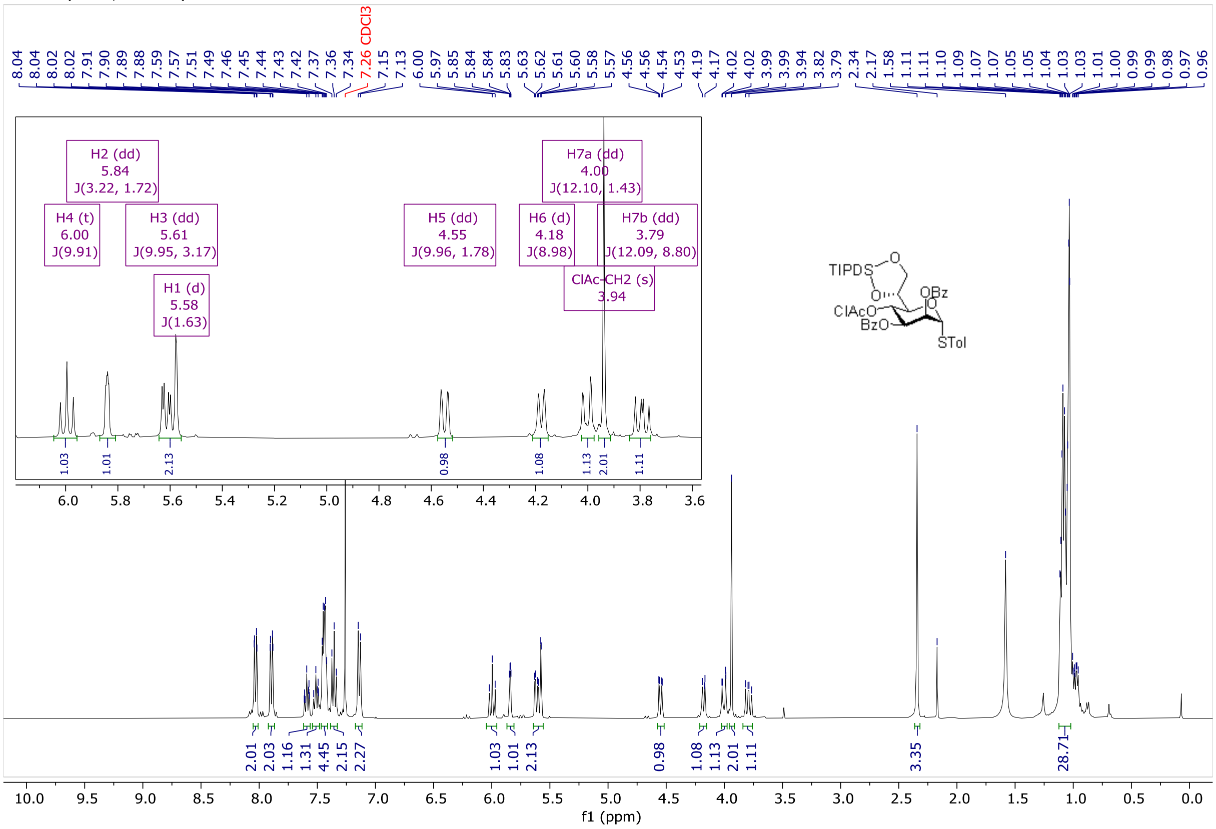

13C NMR (CDCl3, 101 MHz)

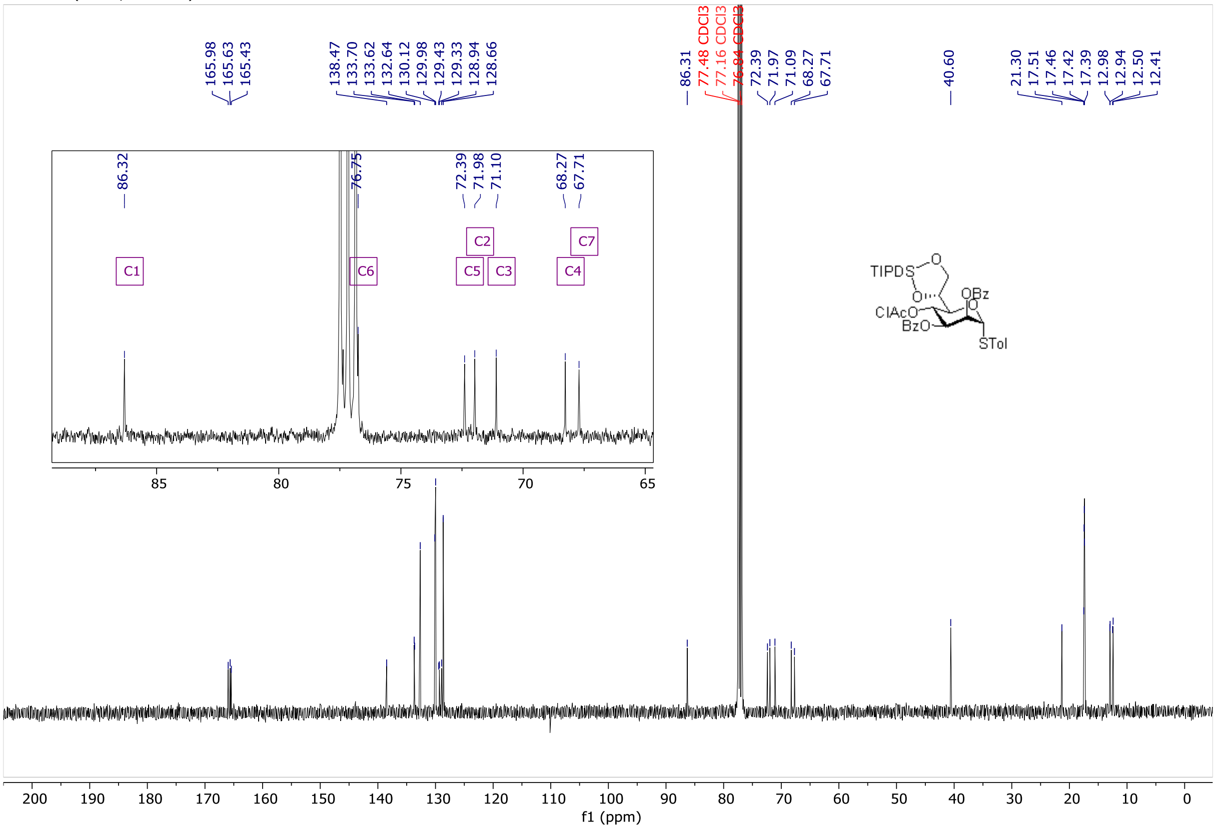

C.2.10. Tol 4-yl 3-*O*-benzoyl-6,7-*O*-(1,1,3,3-tetraisopropyl-1,3-disiloxan-1,3-diyl)-1-thio-L-*glycero*-α-D-*manno*-heptopyranoside (18)

1H NMR (CDCl3, 400 MHz)

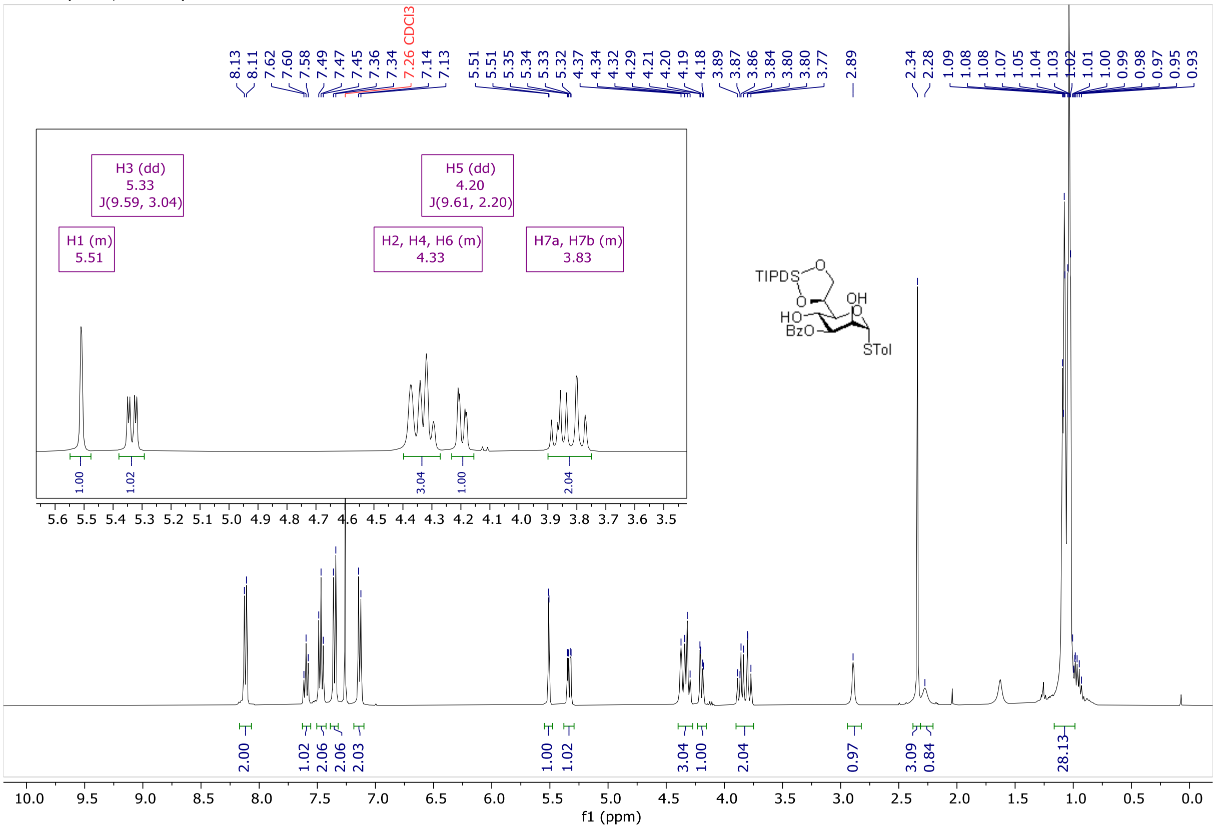

13C NMR (CDCl3, 101 MHz)

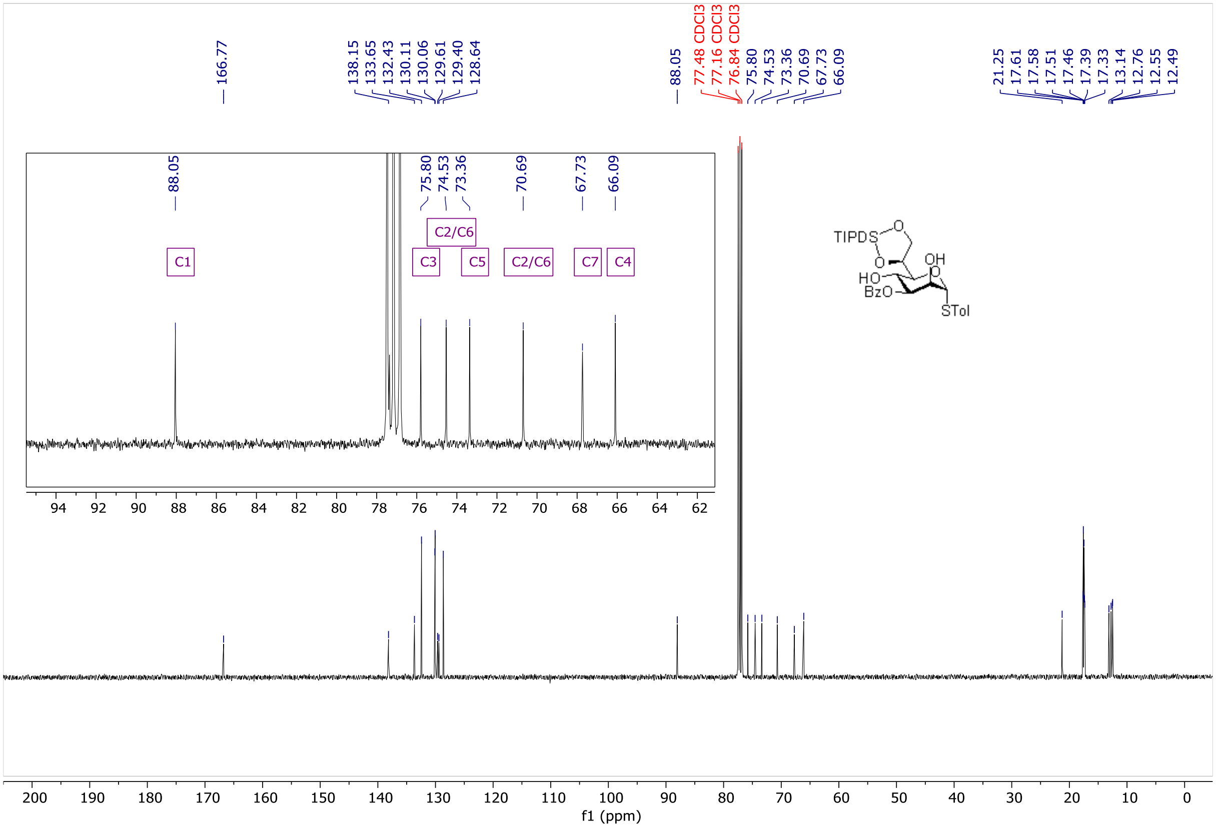

C.2.11. Tol 4-yl 3-*O*-benzoyl-2-*O*-chloroacetyl-6,7-*O*-(1,1,3,3-tetraisopropyl-1,3-disiloxan-1,3-diyl)-1-thio-L-*glycero*-α-D-*manno*-heptopyranoside (19)

1H NMR (CDCl3, 400 MHz)

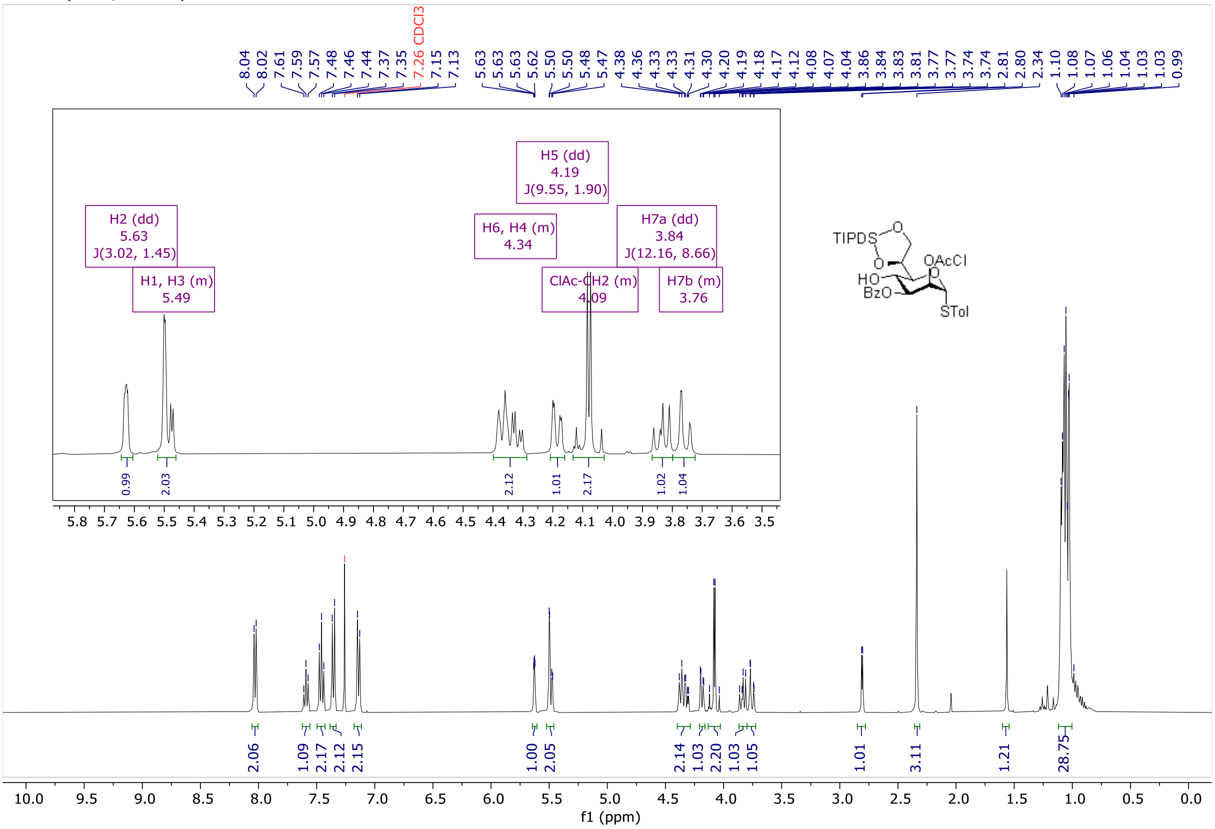

13C NMR (CDCl3, 101 MHz)

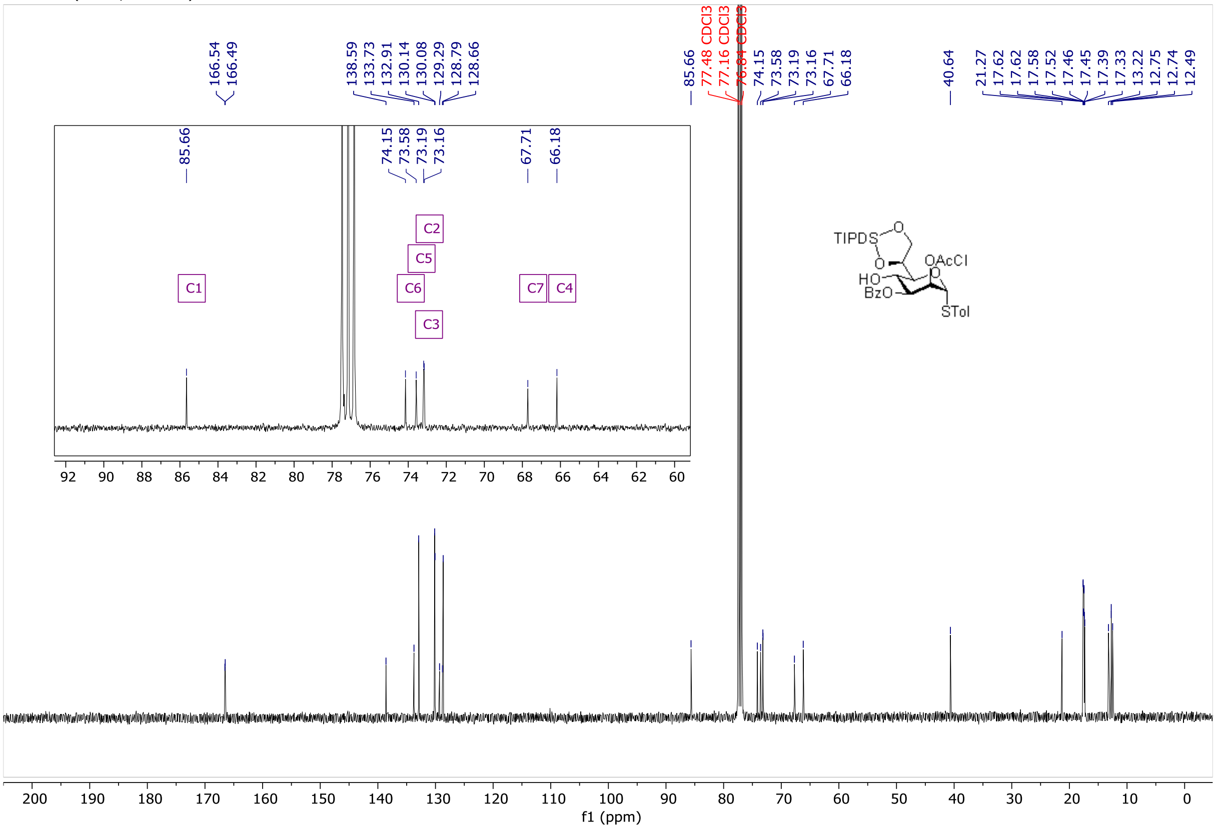

C.2.12. Tol 4-yl 3-*O*-benzoyl-2,4-*O*-dichloroacetyl-6,7-*O*-(1,1,3,3-tetraisopropyl-1,3-disiloxan-1,3-diyl)-1-thio-L-*glycero*-α-D-*manno*-heptopyranoside (by-product of 19)

1H NMR (CDCl3, 400 MHz)

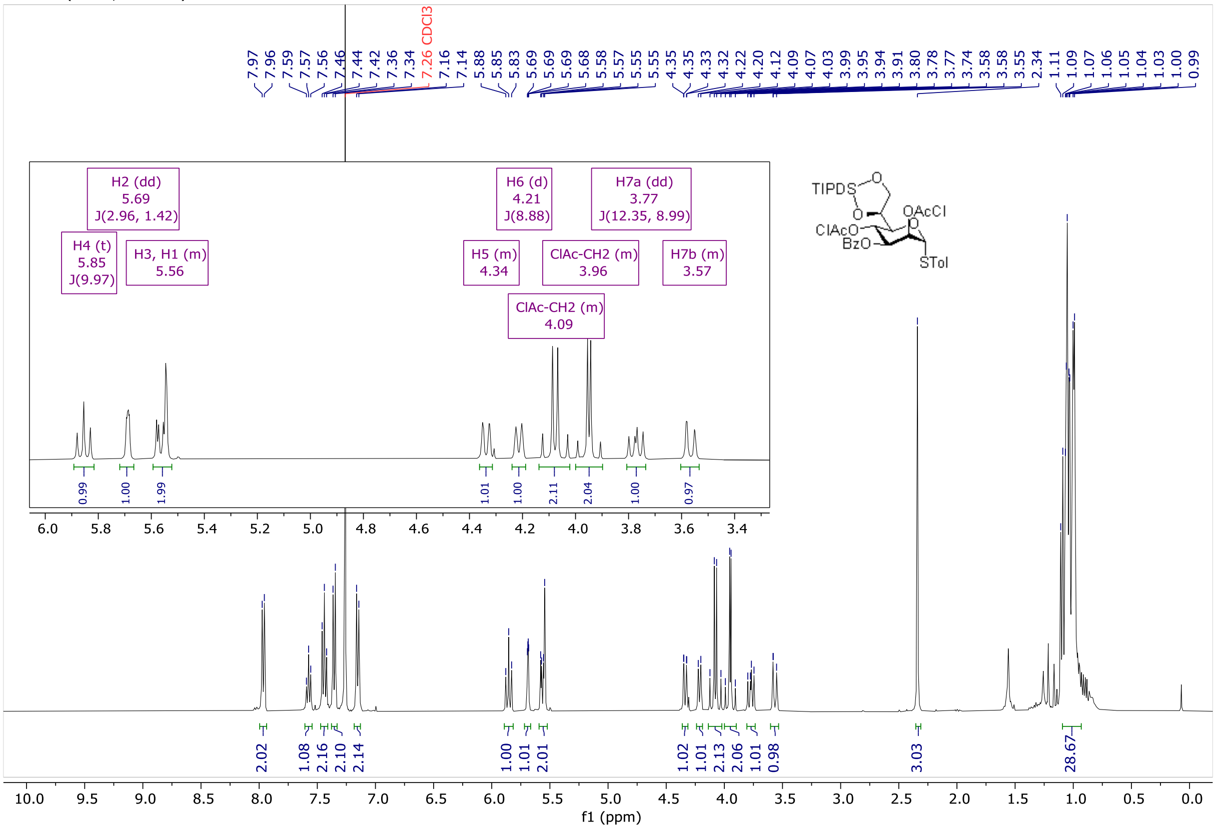

13C NMR (CDCl3, 101 MHz)

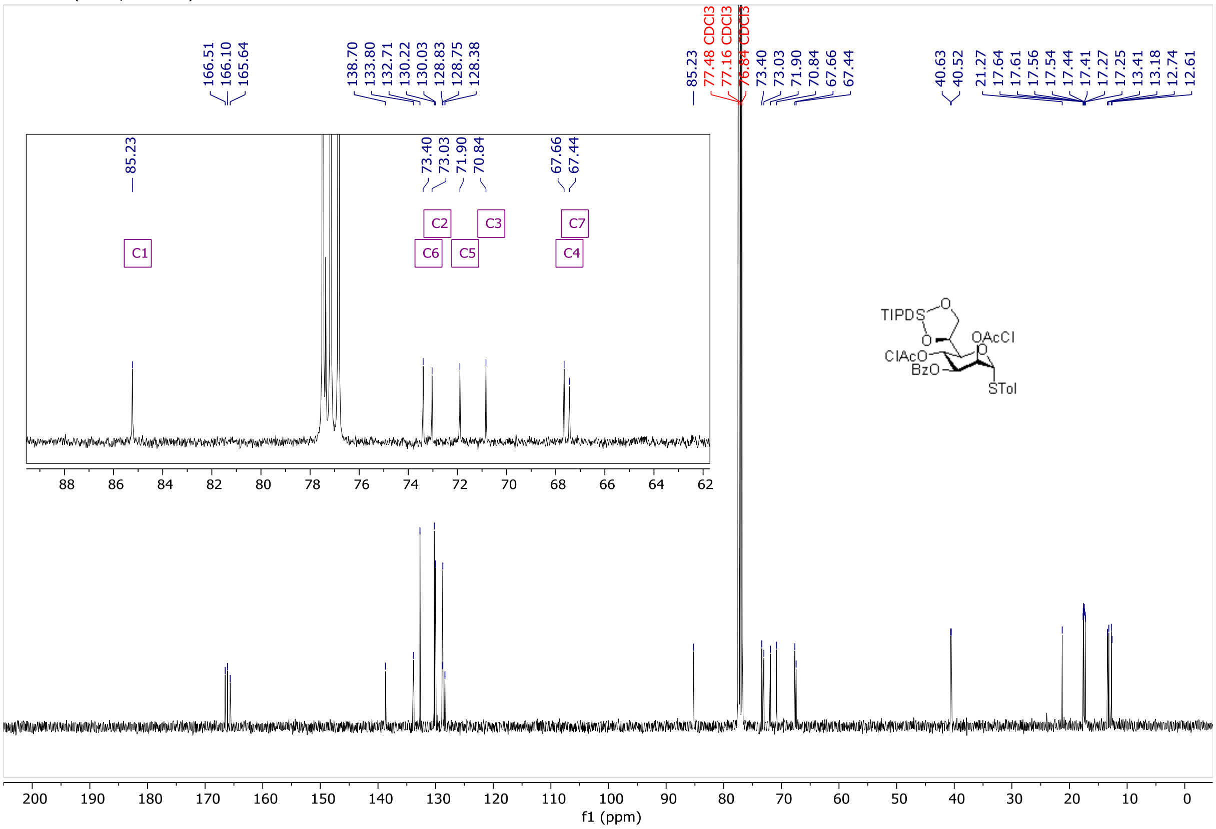

C.2.13. Tol-4-yl 3,4-di-*O*-benzoyl-2-*O*-chloroacetyl-6,7-*O*-(1,1,3,3-tetraisopropyl-1,3-disiloxan-1,3-diyl)-1-thio-L-*glycero*-α-D-*manno*-heptopyranoside (17)

1H NMR (CDCl3, 600 MHz)

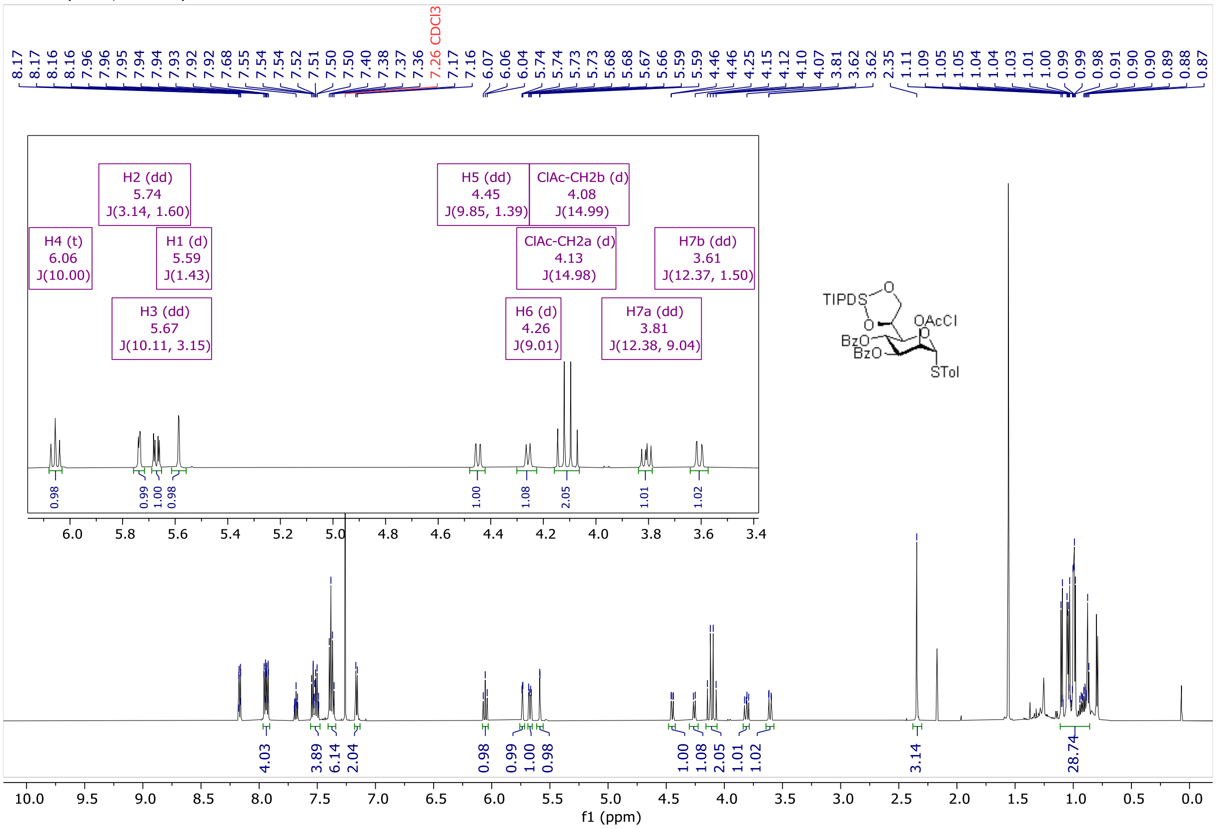

13C NMR (CDCl3, 151 MHz)

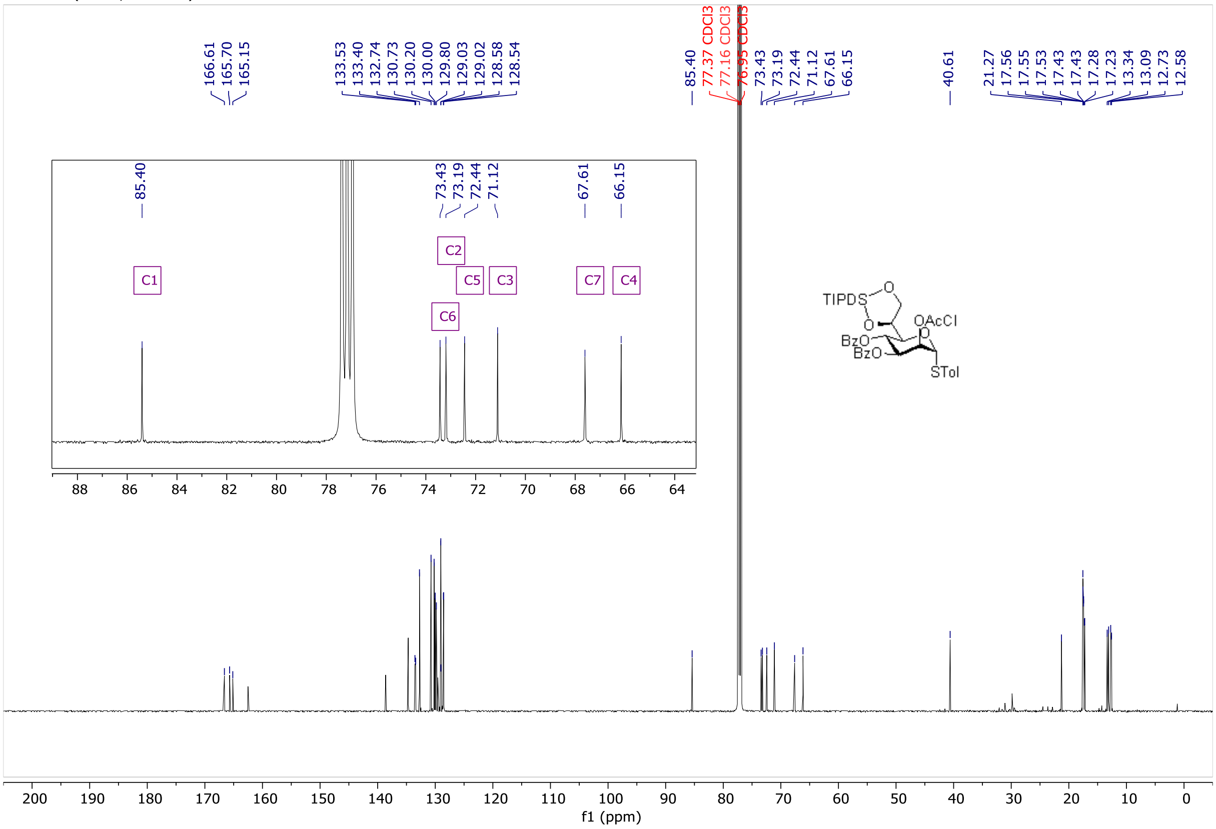

C.2.14. Tol-4-yl 2,3,4-tri-*O*-benzoyl-6,7-*O*-(1,1,3,3-tetraisopropyl-1,3-disiloxane-1,3-diyl)-1-thio-L-*glycero*-α-D-*manno*-heptopyranoside (21)

1H NMR (CDCl3, 600 MHz)

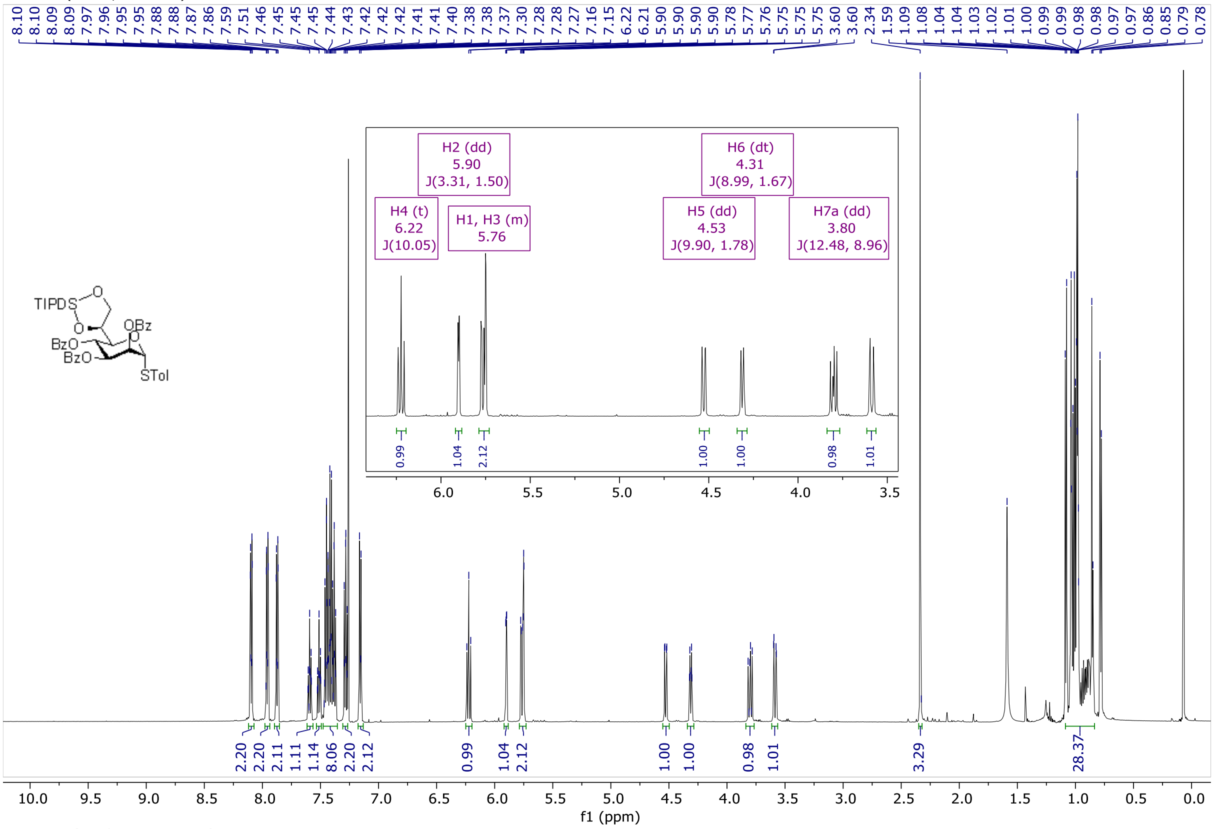

13C NMR (CDCl3, 100.62 MHz)

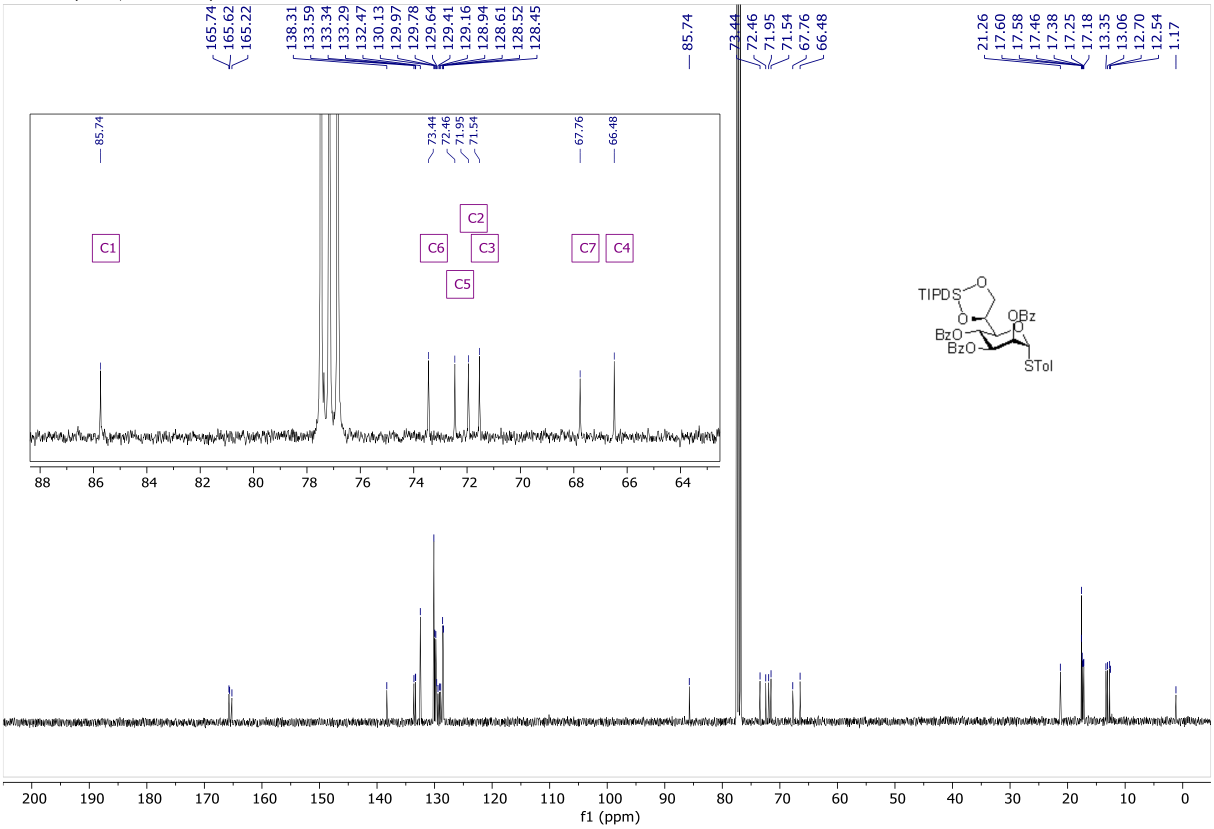

C.2.15. Tol-4-yl 2,3,4-tri-*O*-benzoyl-6,7-*O*-(1,1,3,3-tetraisopropyl-1,3-disiloxane-1,3-diyl)-1-thio-D-*glycero*-α-D-*manno*-heptopyranoside (29)

1H NMR (CDCl3, 400 MHz)

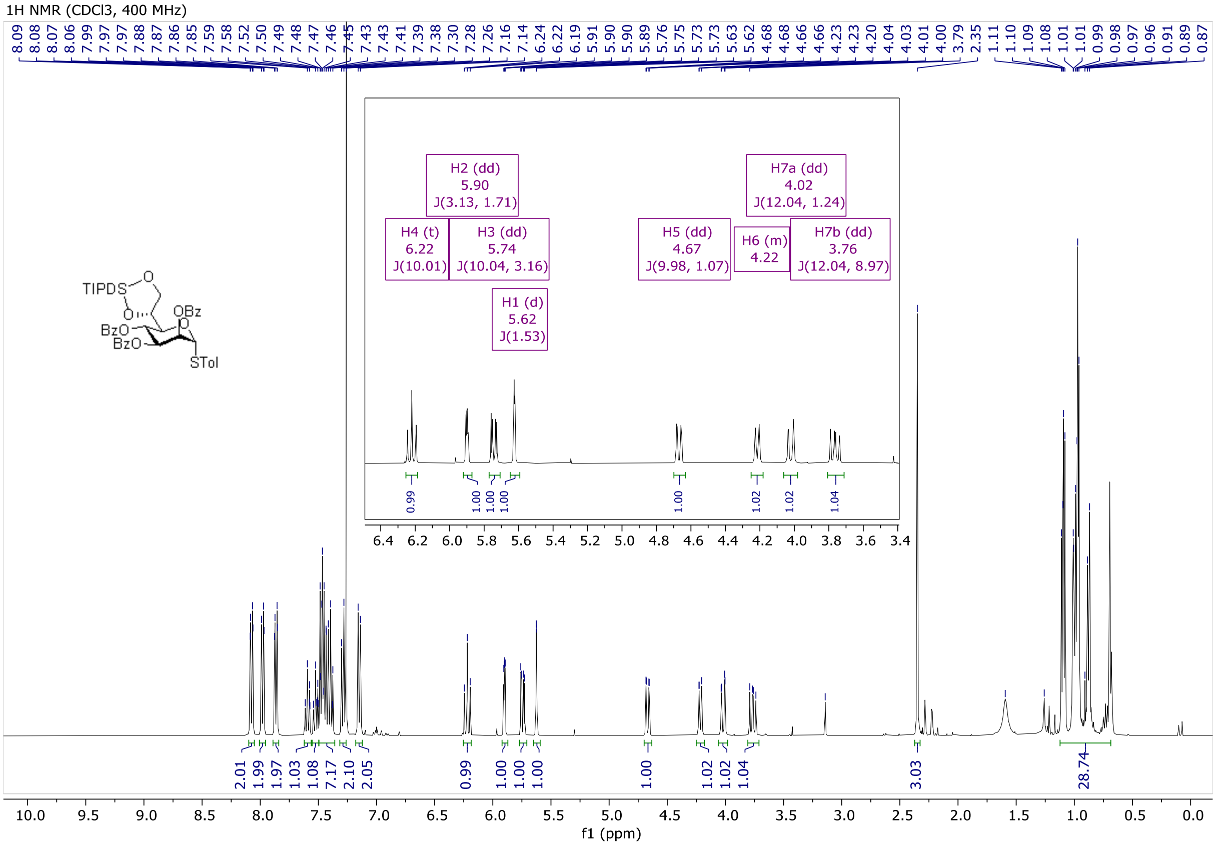

13C NMR (CDCl3, 101 MHz)

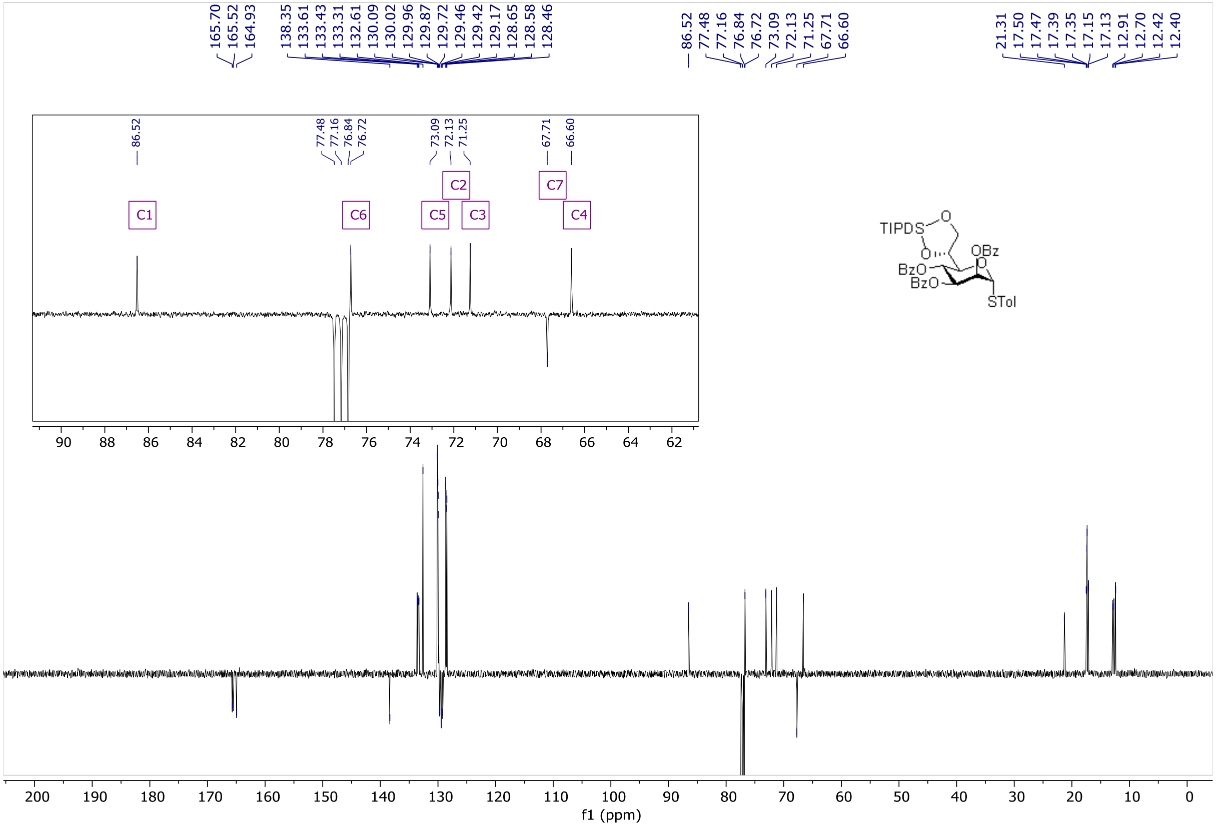

C.2.16. Tol-4-yl 2,3,4-tri-*O*-acetyl-6,7-*O*-(1,1,3,3-tetraisopropyl-1,3-disiloxane-1,3-diyl)-1-thio-D-*glycero*-α-D-*manno*-heptopyranoside (31)

1H NMR (CDCl3, 400 MHz)

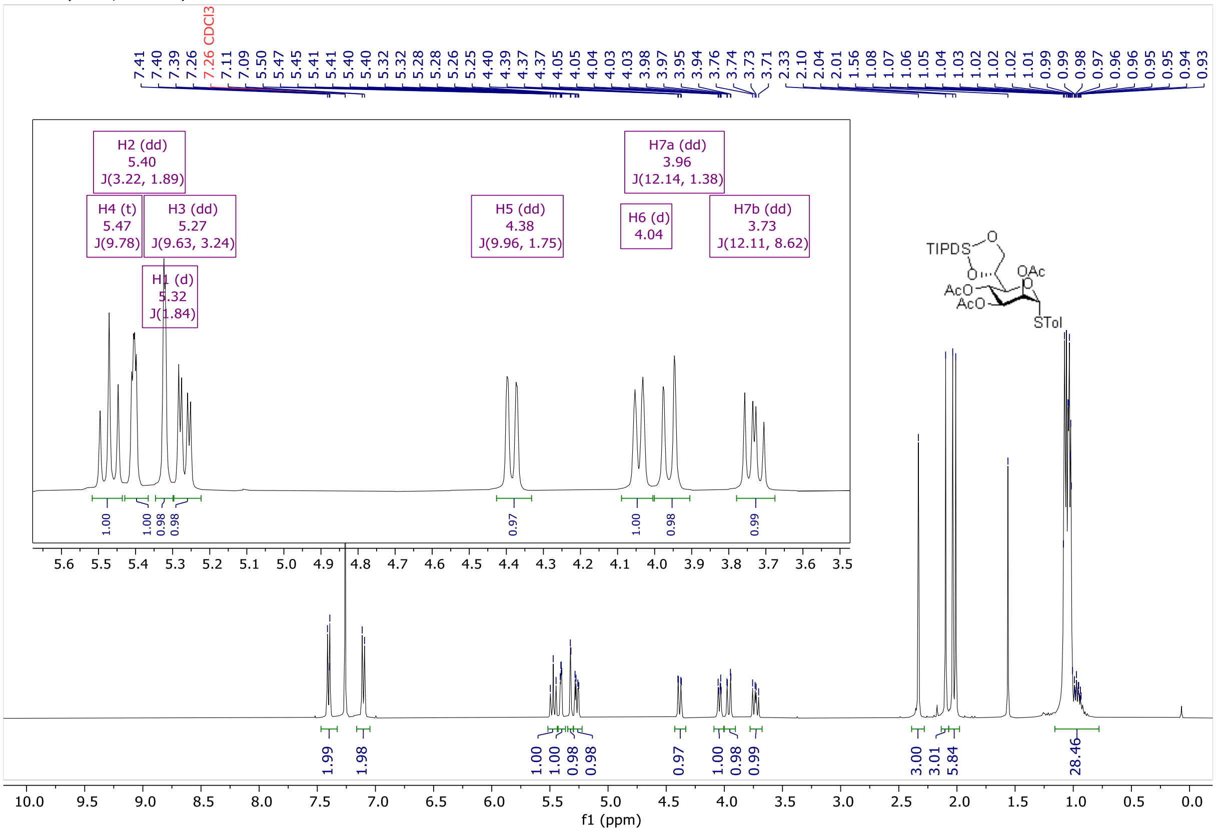

13C NMR (CDCl3, 101 MHz)

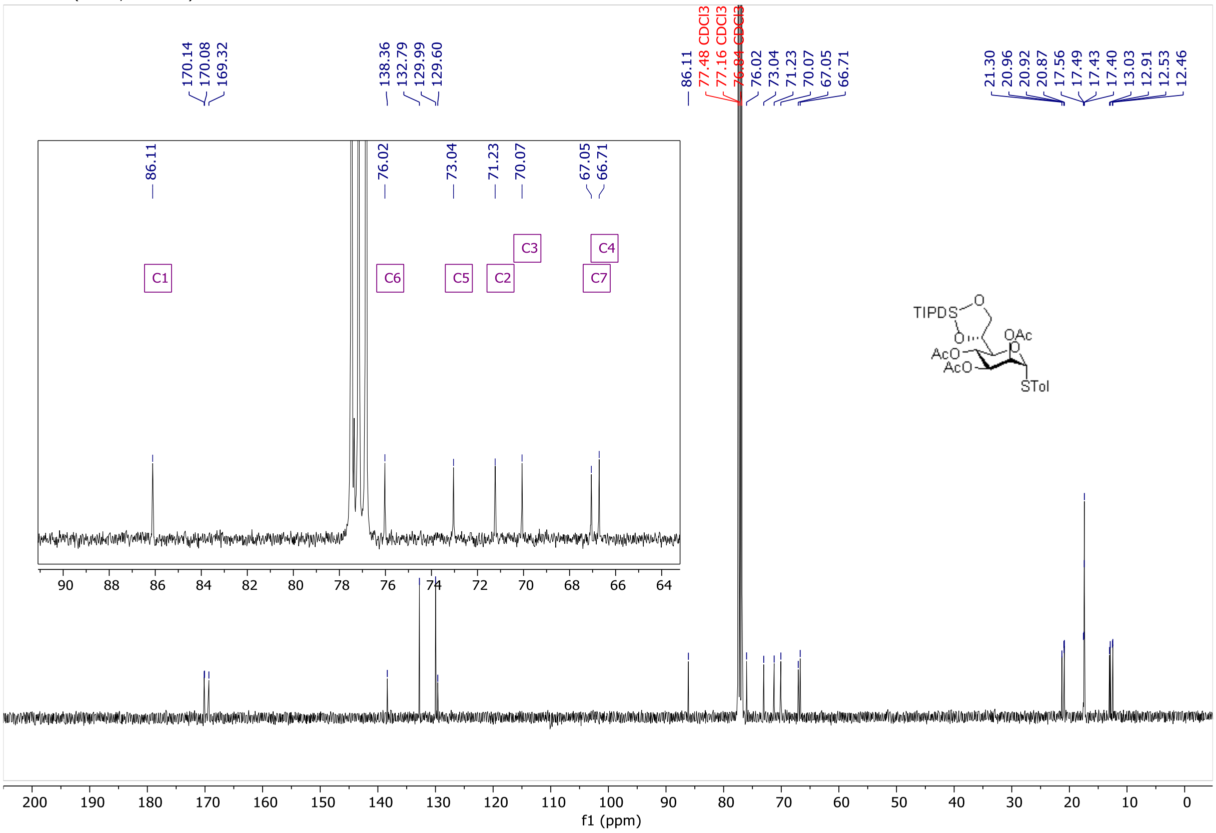

C.3. Regioselective partial cleavage of TIPDS

C.3.1. Methyl 2,4-di-*O*-benzoyl-3-*O*-chloroacetyl-6-*O*-(3-fluoro-1,1,3,3-tetraisopropyl-1,3-disiloxane-1-yl)-L-*glycero*-α-D-*manno*-heptopyranoside (20)

1H NMR (CDCl3, 400 MHz)

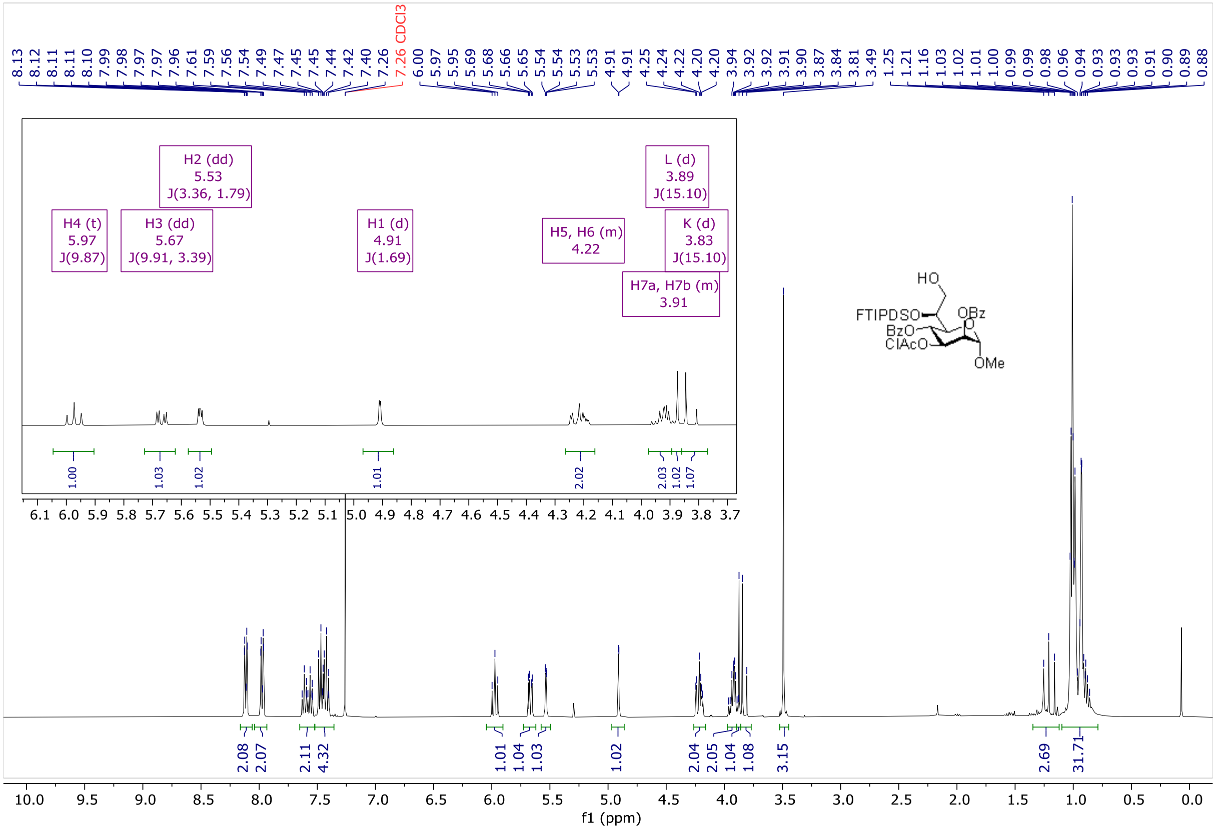

13C NMR (CDCl3, 101 MHz)

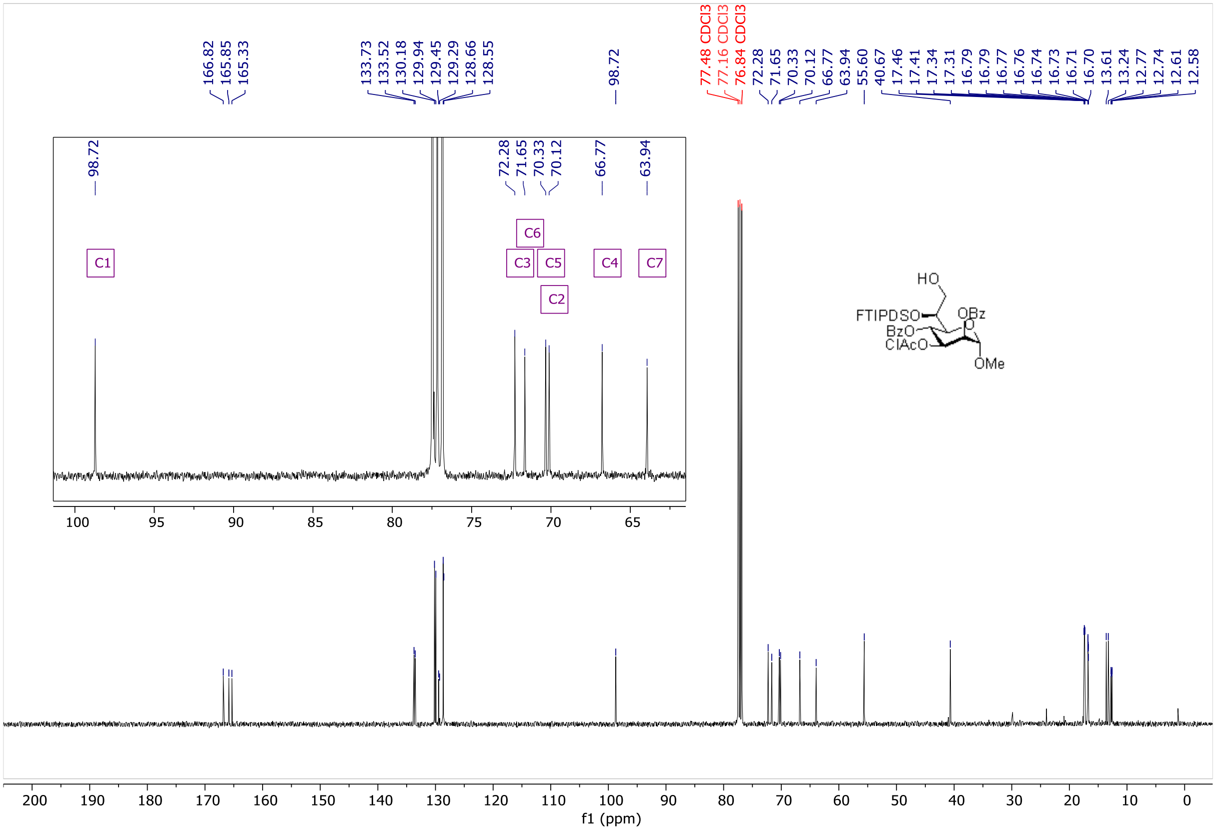

C.3.2. Tol-4-yl 2,3,4-tri-*O*-benzoyl-6-*O*-(3-fluoro-1,1,3,3-tetraisopropyl-1,3-disiloxane-1-yl)-1-thio-L-*glycero*-α-D-*manno*-heptopyranoside (22)

1H NMR (CDCl3, 400 MHz)

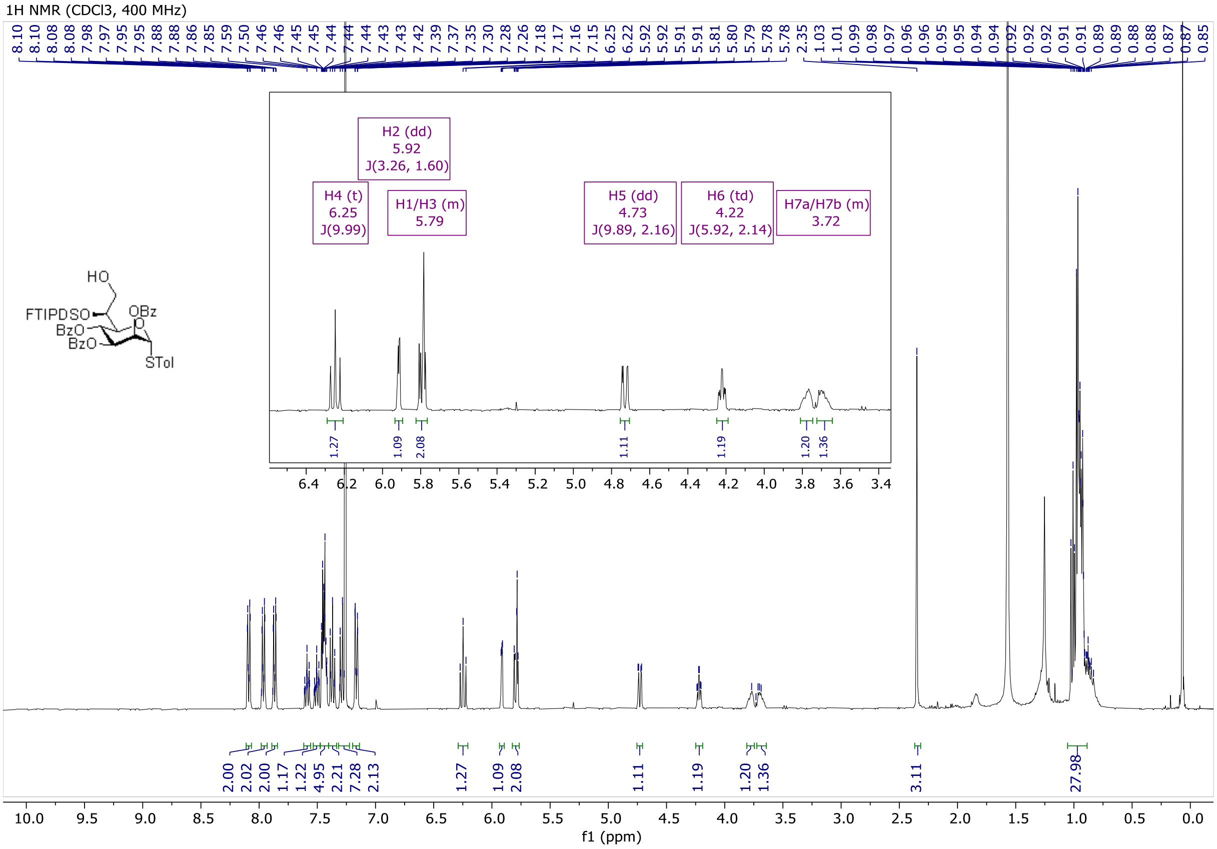

13C NMR (CDCl3, 101 MHz)

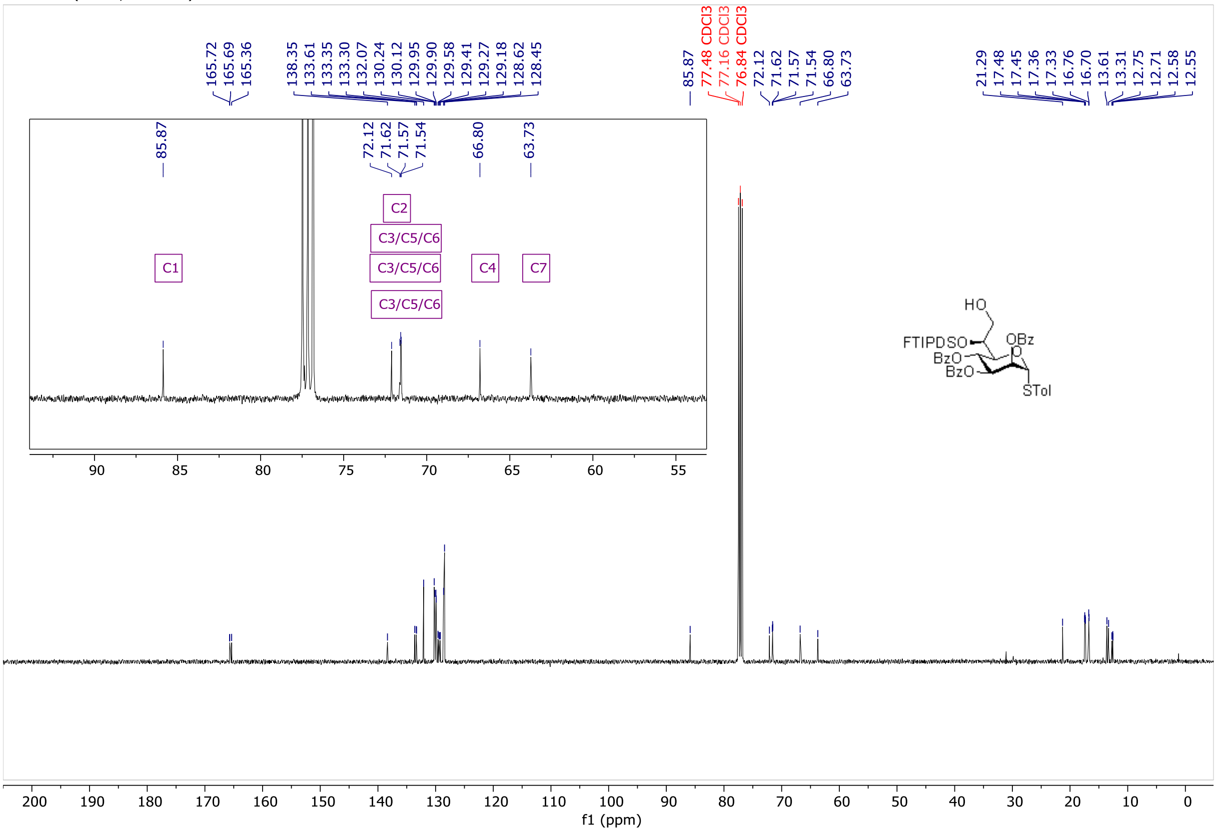

C.3.3. Tol-4-yl 2,3,4-tri-*O*-benzoyl-1-thio-L-*glycero*-α-D-*manno*-heptopyranoside (22a)

1H NMR (CDCl3, 400 MHz)

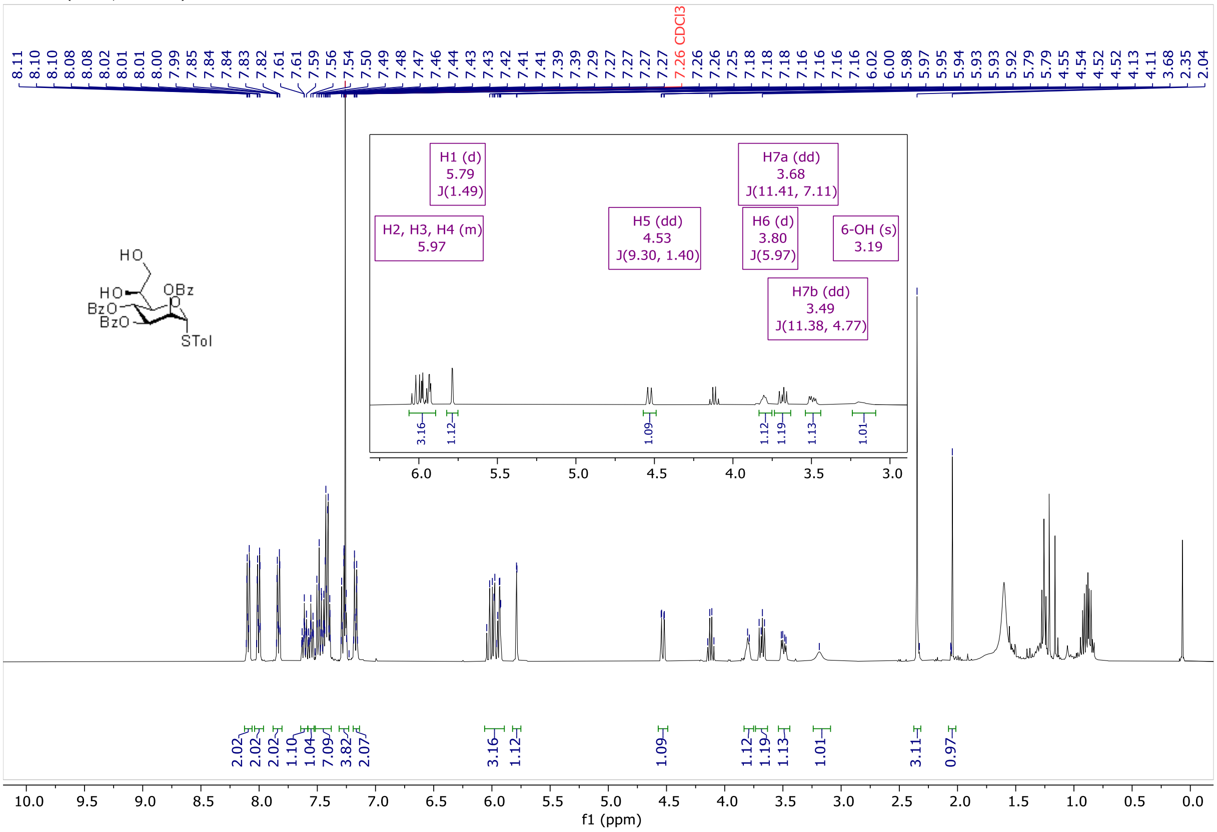

13C NMR (CDCl3, 101 MHz)

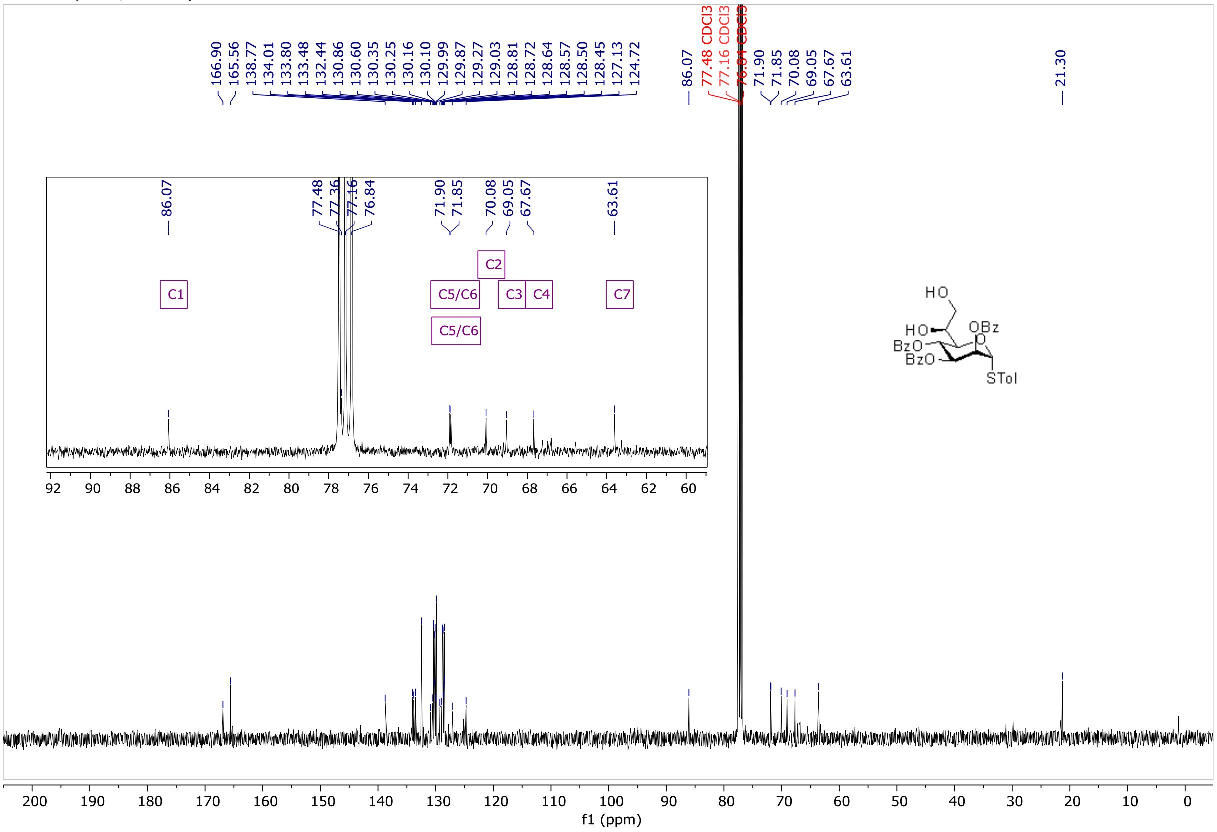

C.3.4. Tol-4-yl 2,3,4-tri-*O*-acetyl-6-*O*-(3-fluoro-1,1,3,3-tetraisopropyl-1,3-disiloxane-1-yl)-1-thio-L-*glycero*-α-D-*manno*-heptopyranoside (24)

1H NMR (CDCl3, 400 MHz)

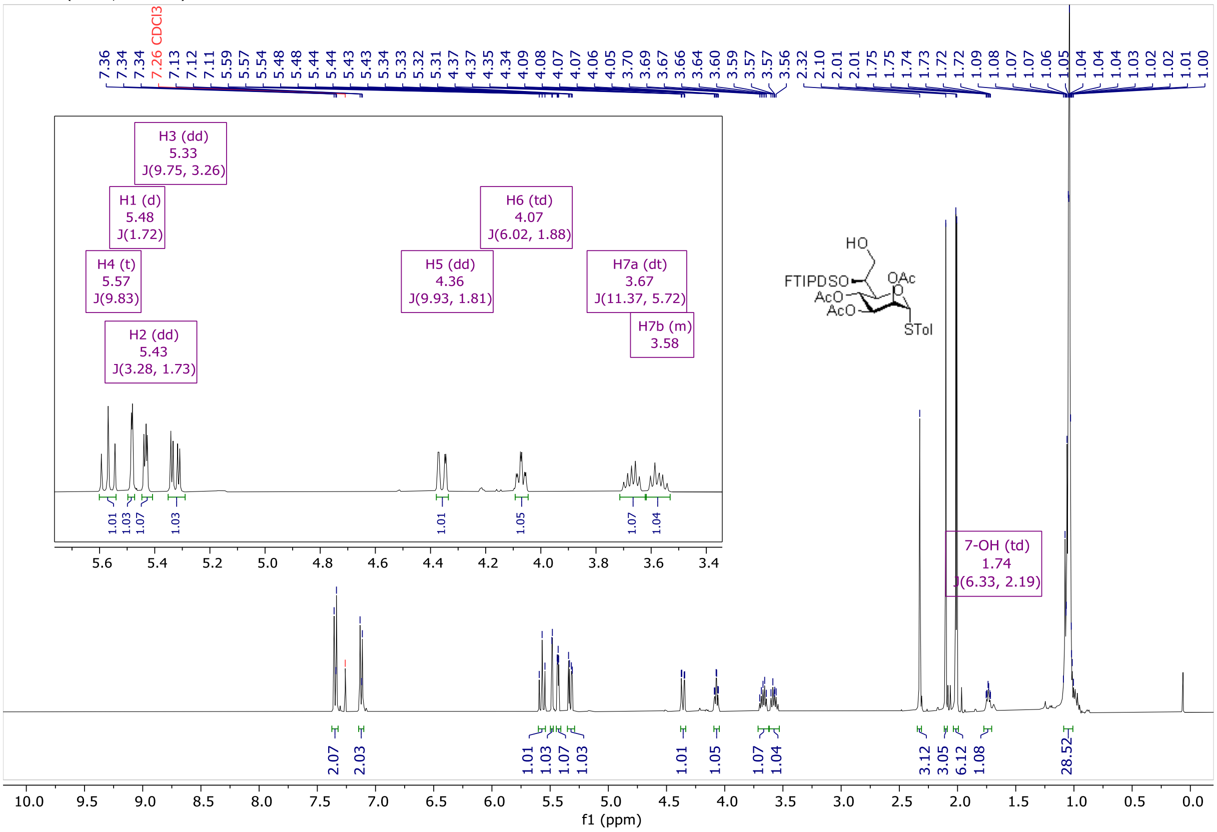

13C NMR (CDCl3, 101 MHz)

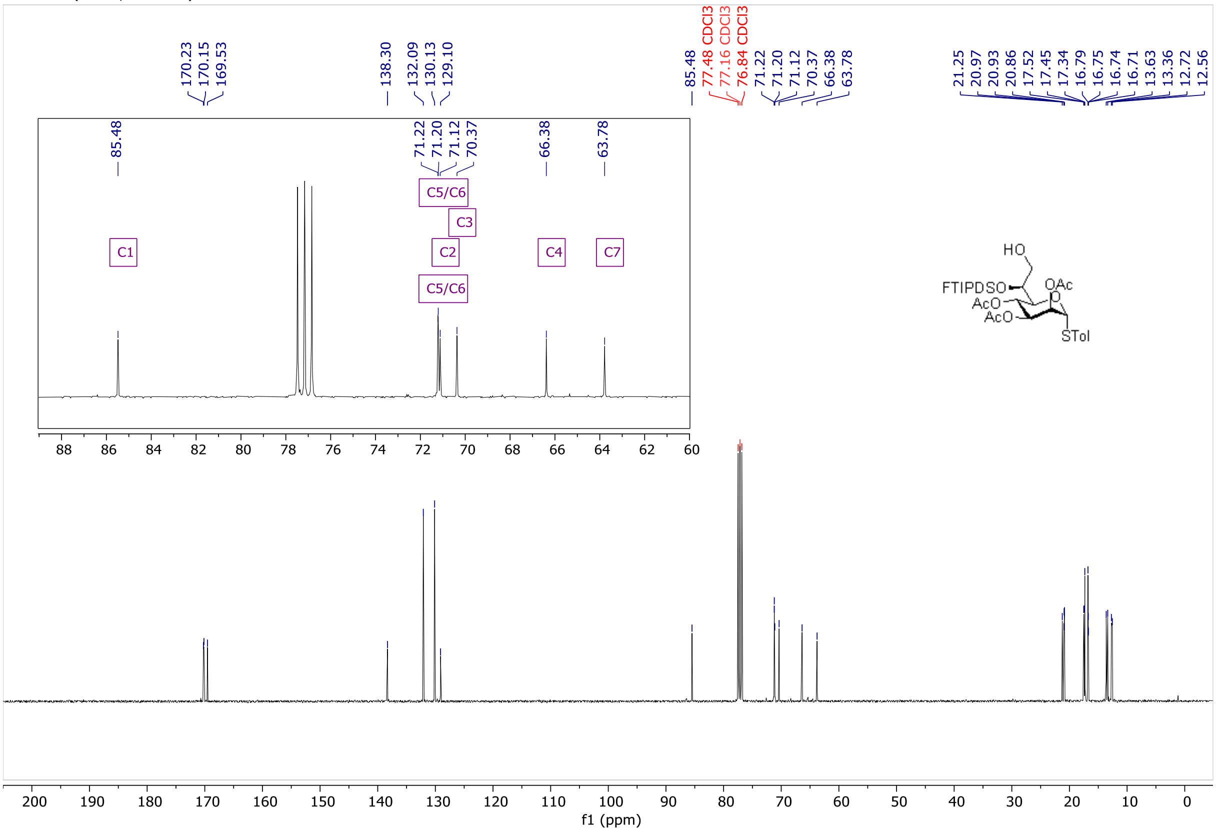

C.3.5. Tol-4-yl 2,3,4-tri-*O*-acetyl-1-thio-L-*glycero*-α-D-*manno*-heptopyranoside (24a)

1H NMR (CDCl3, 600 MHz)

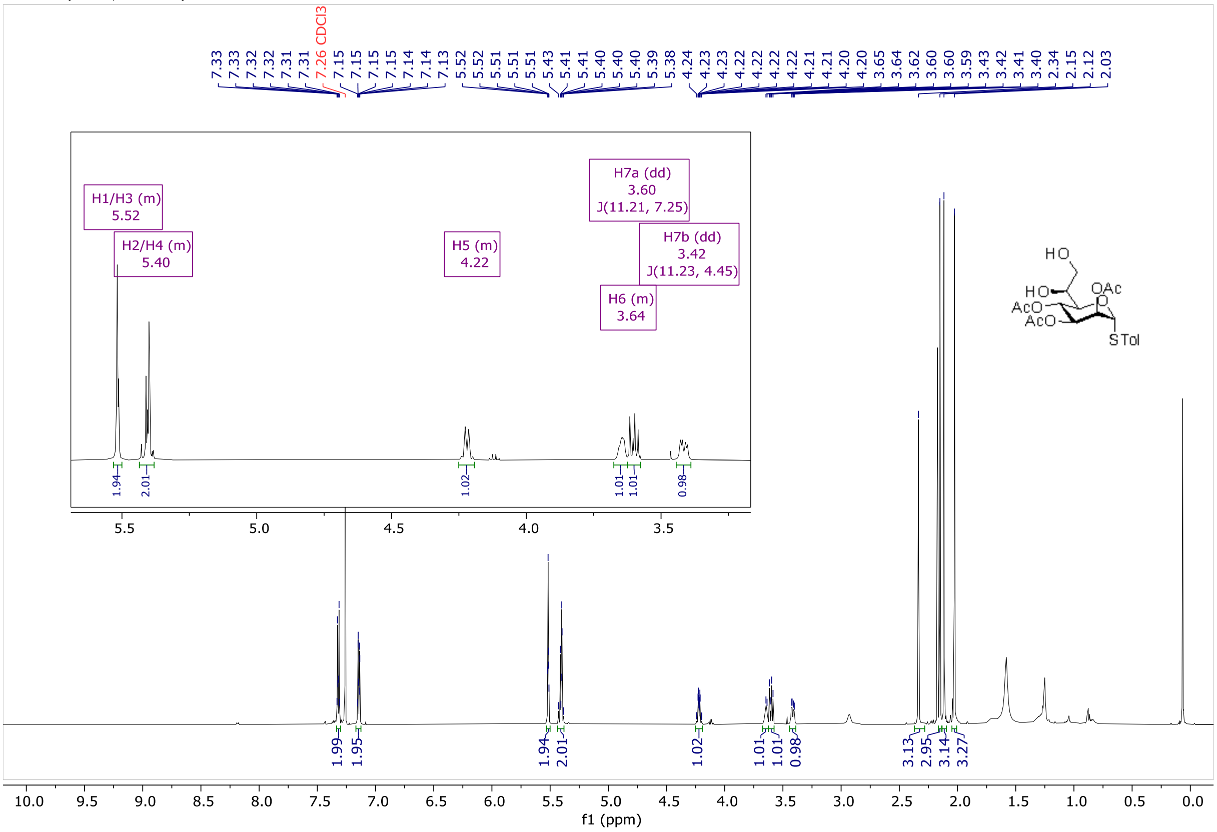

13C NMR (CDCl3, 151 MHz)

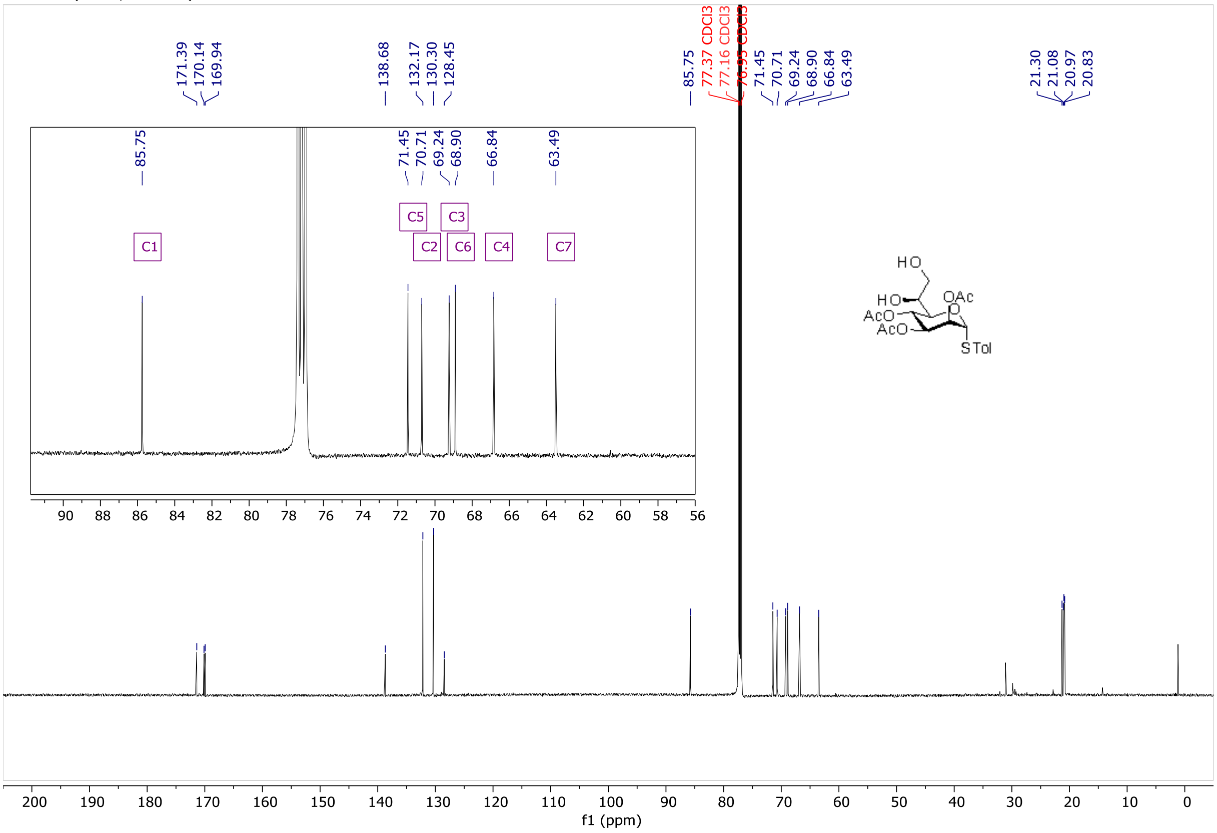

C.3.6. Tol-4-yl 2,4-di-*O*-benzoyl-3-*O*-chloroacetyl-6-*O*-(3-fluoro-1,1,3,3-tetraisopropyl-1,3-disiloxane-1-yl)-1-thio-L-*glycero*-α-D-*manno*-heptopyranoside (26)

1H NMR (CDCl3, 400 MHz)

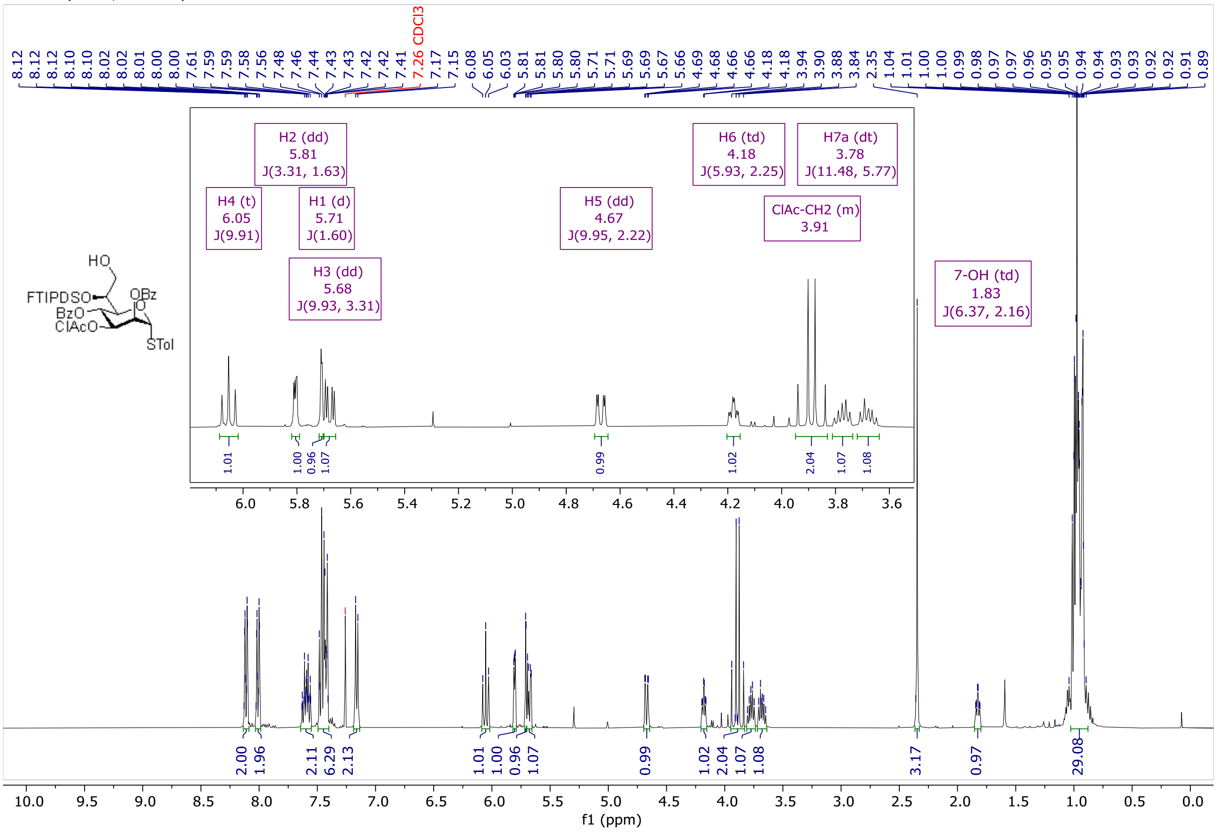

13C NMR (CDCl3, 101 MHz)

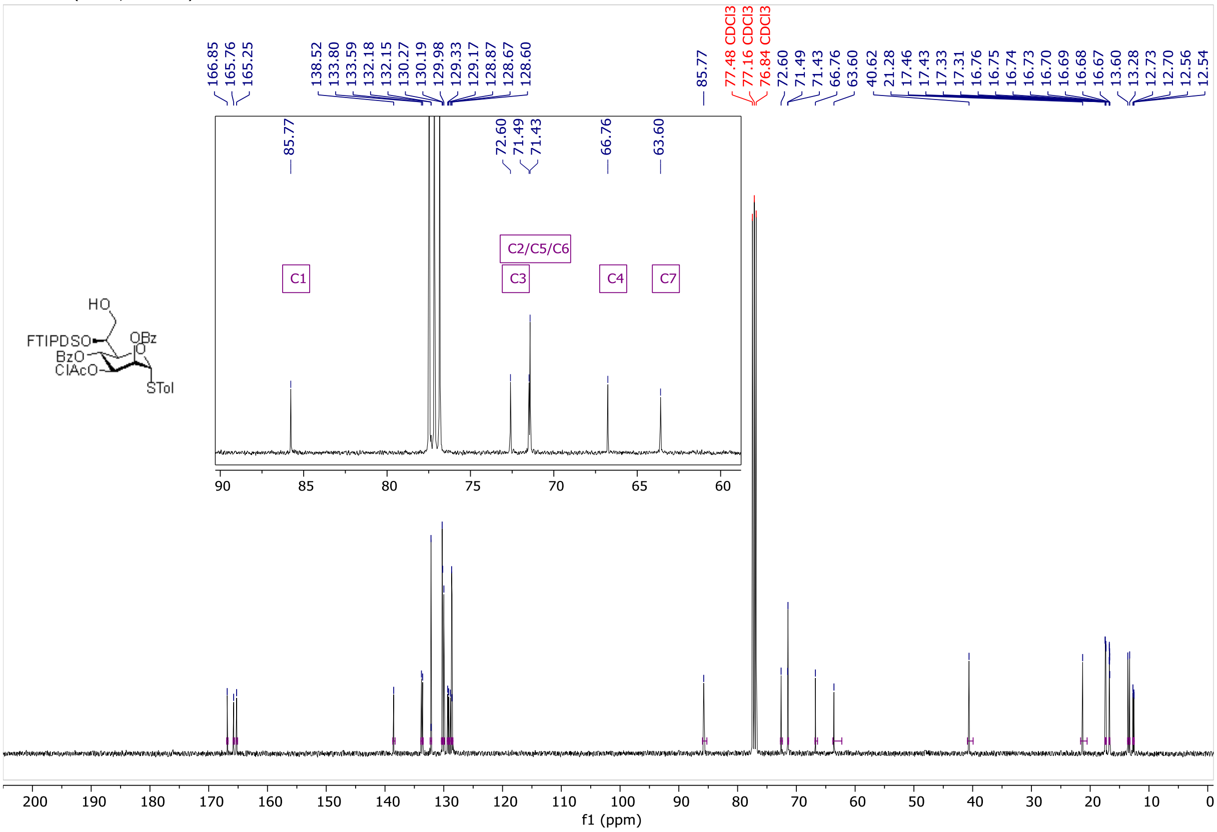

COSY (CDCl3, 400 MHz)

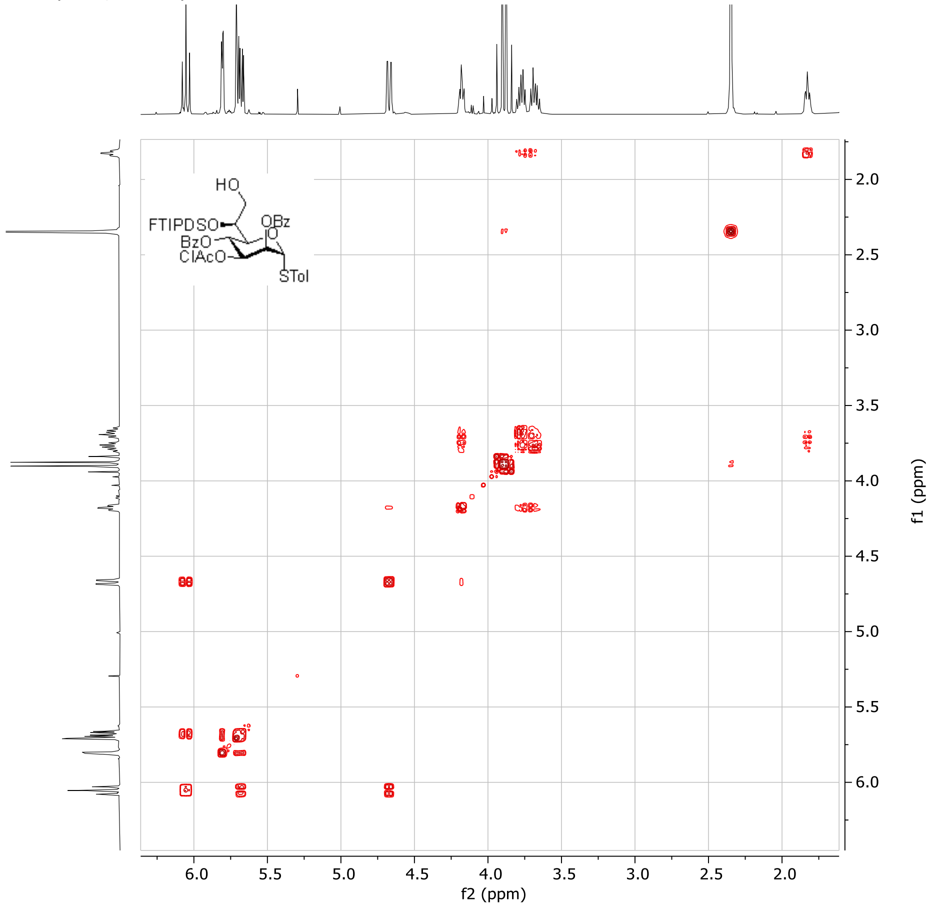

HSQC (CDCl3, 400 MHz)

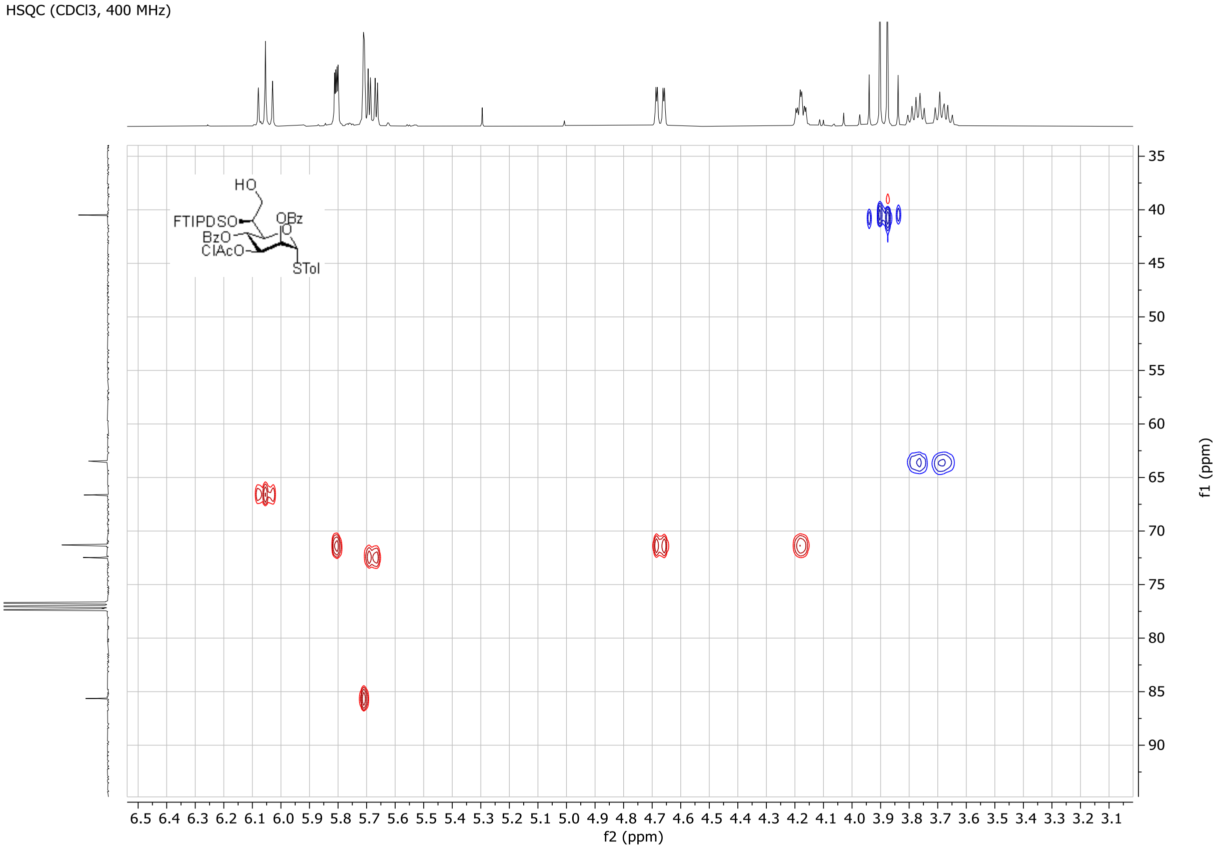

C.3.7. Tol-4-yl 2,3-*O*-(2-chloro-1-ethoxyethyliden-1,1-diyl)- 6-*O*-(3-fluoro-1,1,3,3-tetraisopropyl-1,3-disiloxane-1-yl)-1-thio-L-*glycero*-α-D-*manno*-heptopyranoside (27)

1H NMR (CDCl3, 600 MHz)

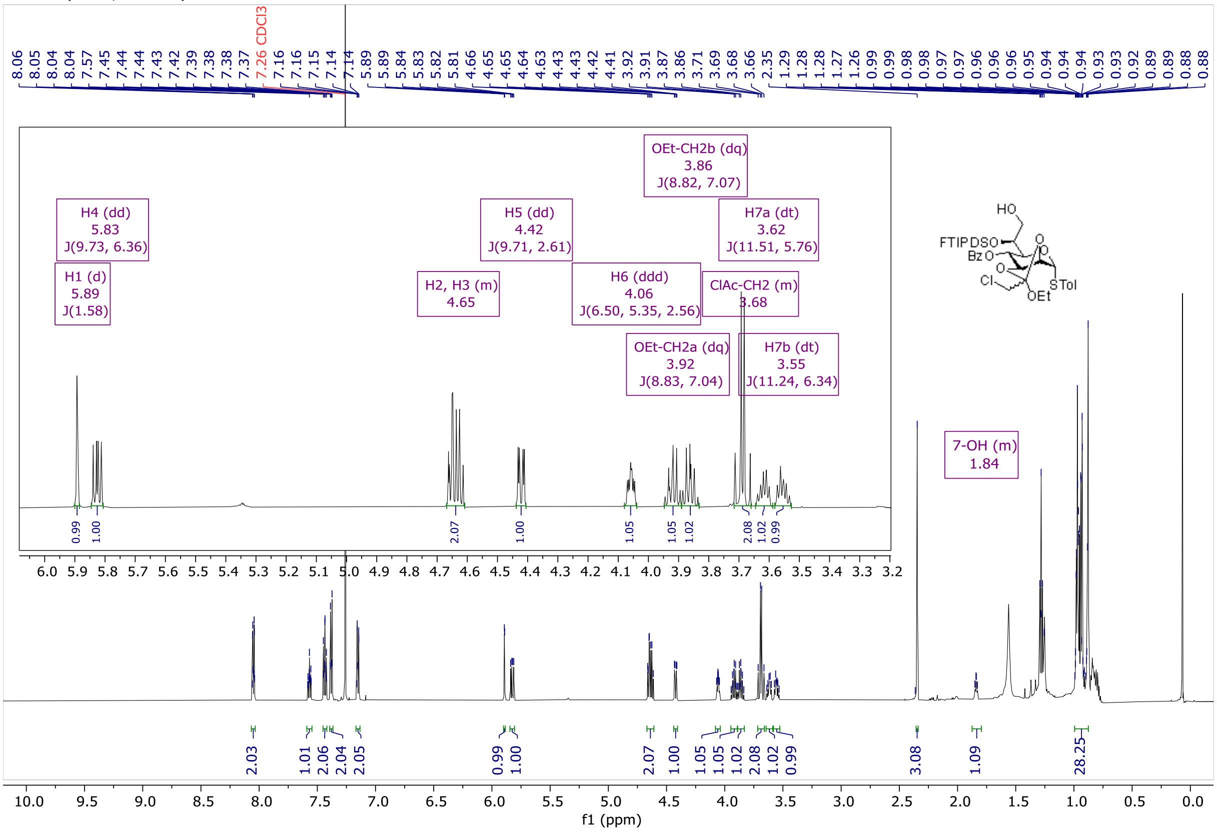

13C NMR (CDCl3, 151 MHz)

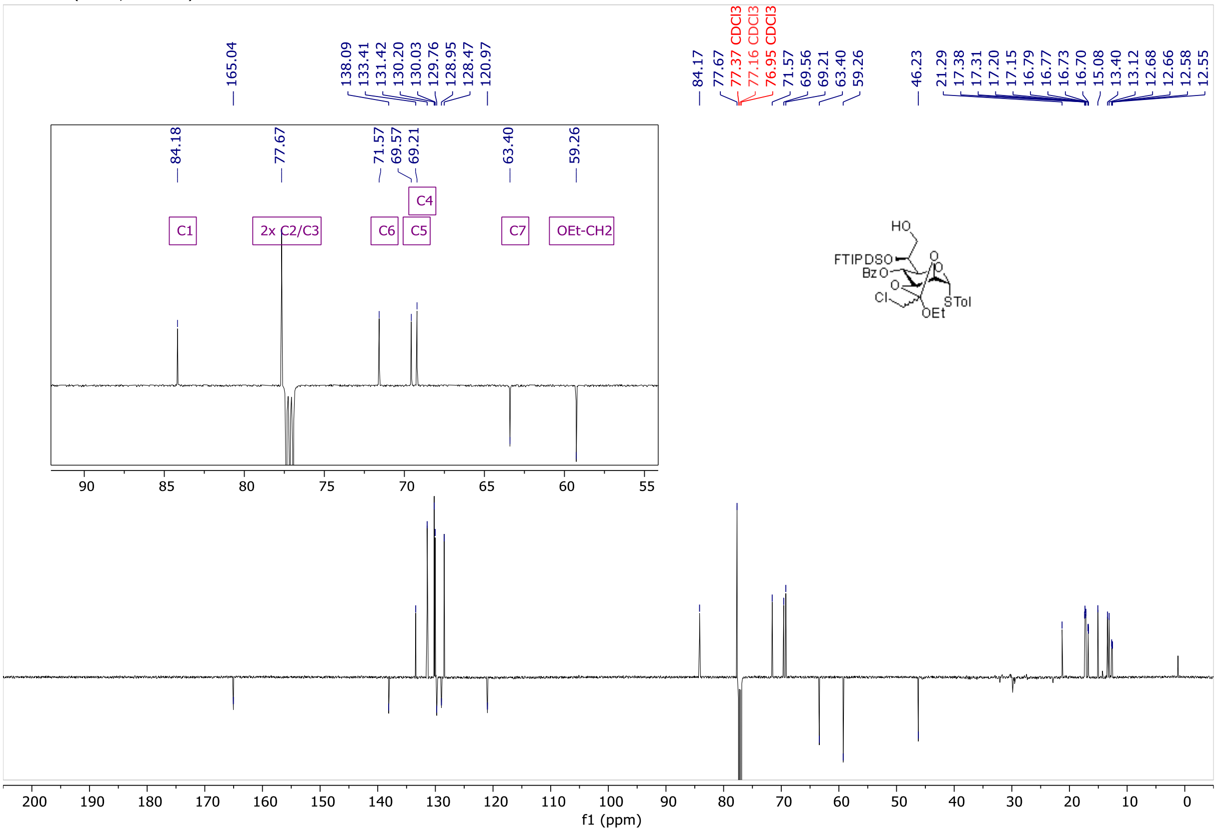

C.3.8. Tol-4-yl 3,4-di-*O*-benzoyl-2-*O*-chloroacetyl-6-*O*-(3-fluoro-1,1,3,3-tetraisopropyl-1,3-disiloxane-1-yl)- 1-thio-L-*glycero*-α-D-*manno*-heptopyranoside (28)

1H NMR ($CDCl_3$, 600 MHz)

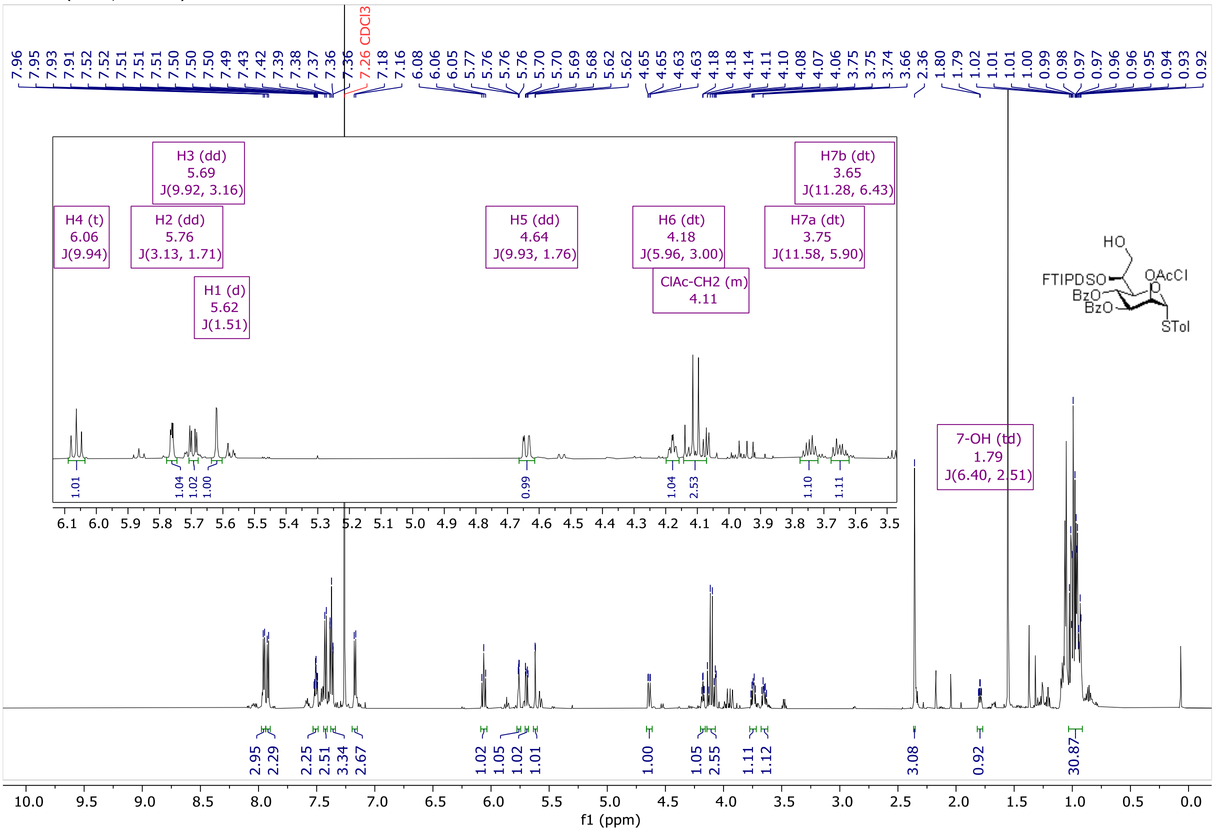

13C NMR ($CDCl_3$, 151 MHz)

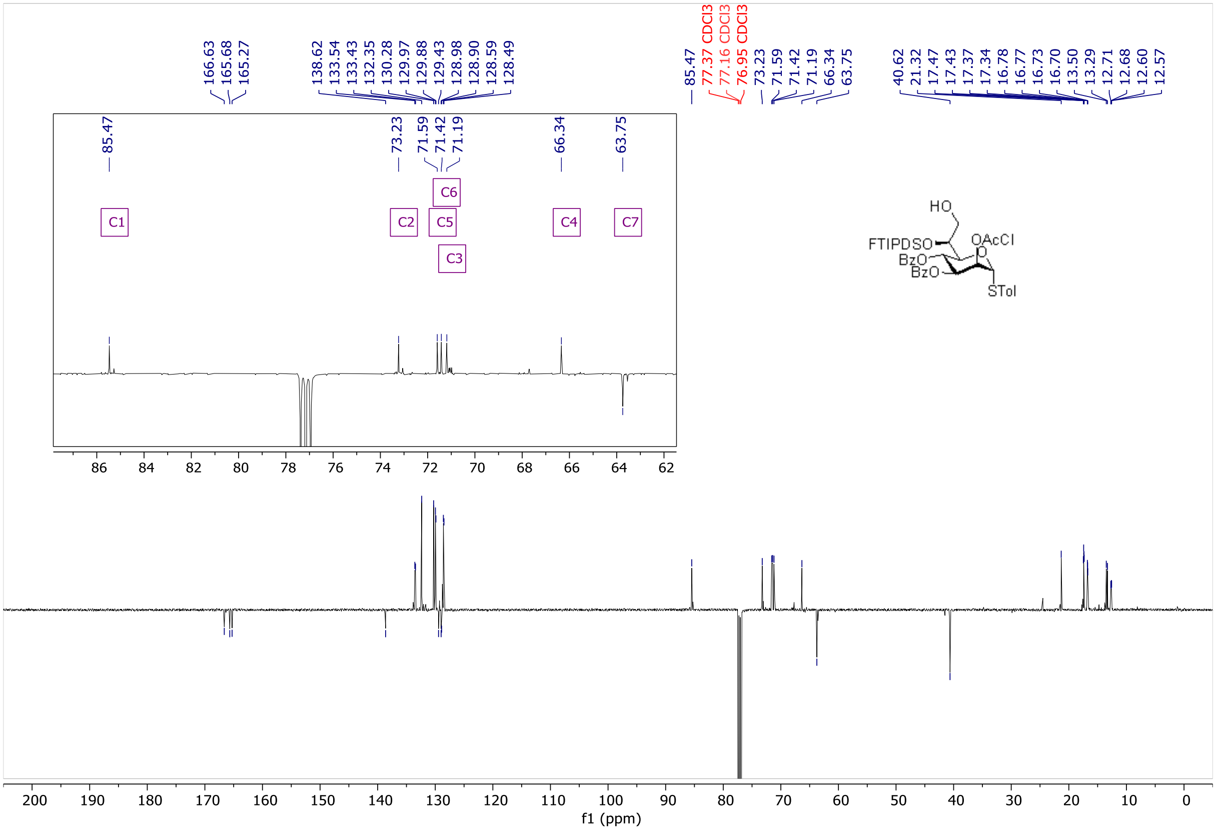

C.3.9. Tol-4-yl 2,3,4-tri-*O*-benzoyl-6-*O*-(3-fluoro-1,1,3,3-tetraisopropyl-1,3-disiloxane-1-yl)- 1-thio-D-*glycero*-α-D-*manno*-heptopyranoside (30)

1H NMR (CDCl3, 400 MHz)

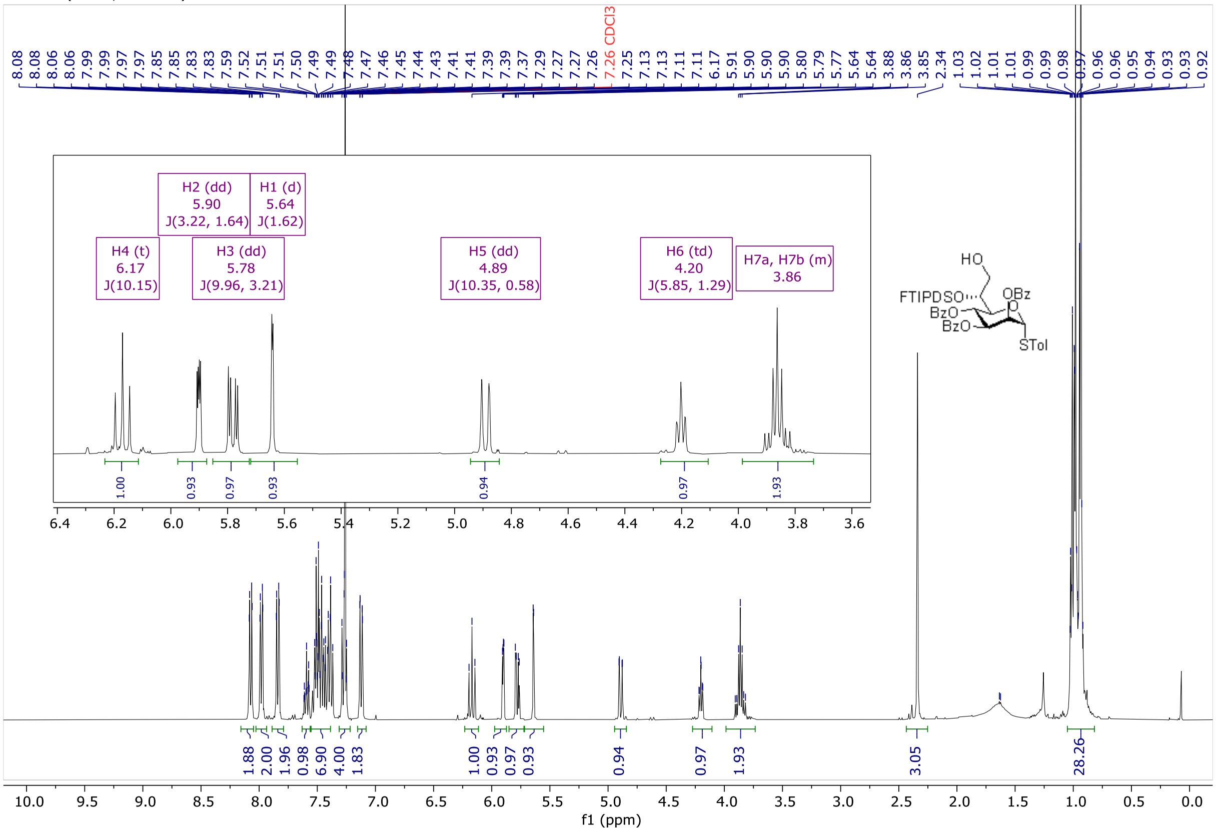

13C NMR (CDCl3, 101 MHz)

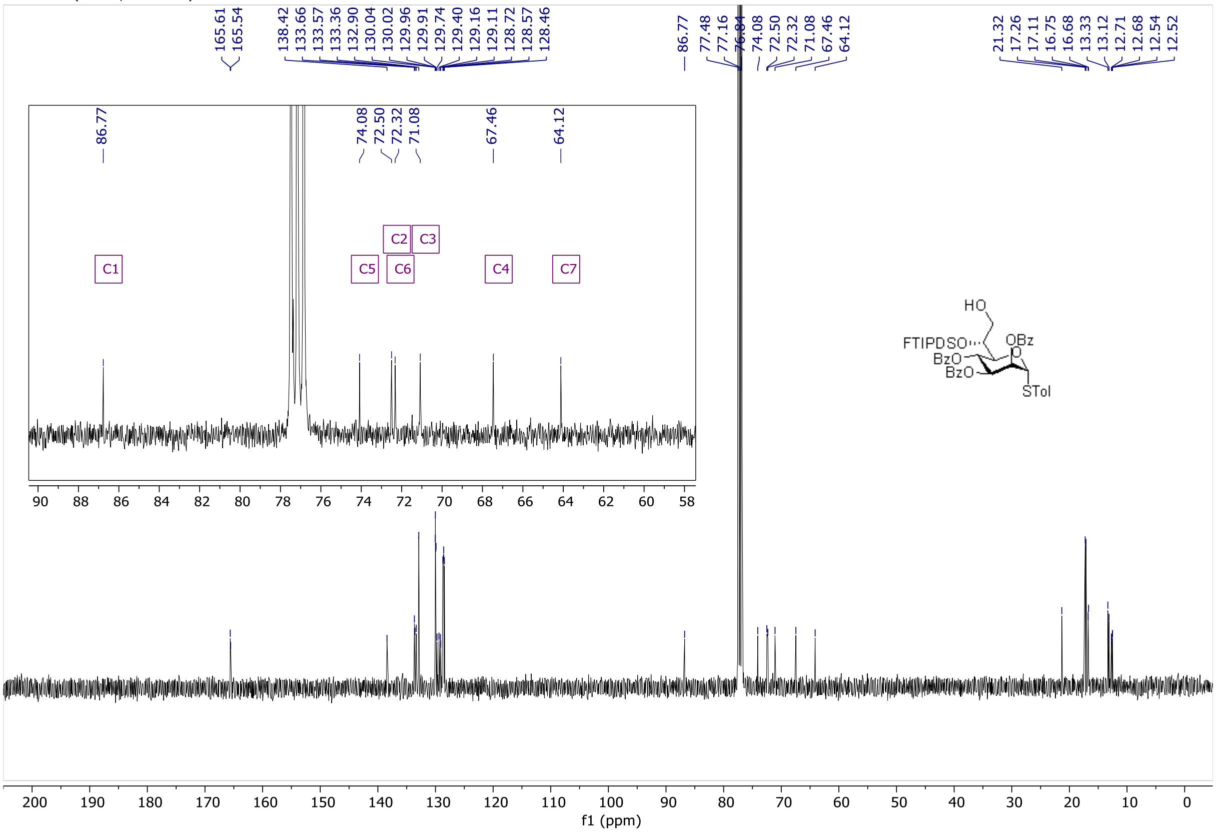

C.3.10. Tol-4-yl 2,3,4-tri-*O*-acetyl-6-*O*-(3-fluoro-1,1,3,3-tetraisopropyl-1,3-disiloxane-1-yl)-1-thio-D-*glycero*-α-D-*manno*-heptopyranoside (32)

1H NMR (CDCl3, 400.13 MHz)

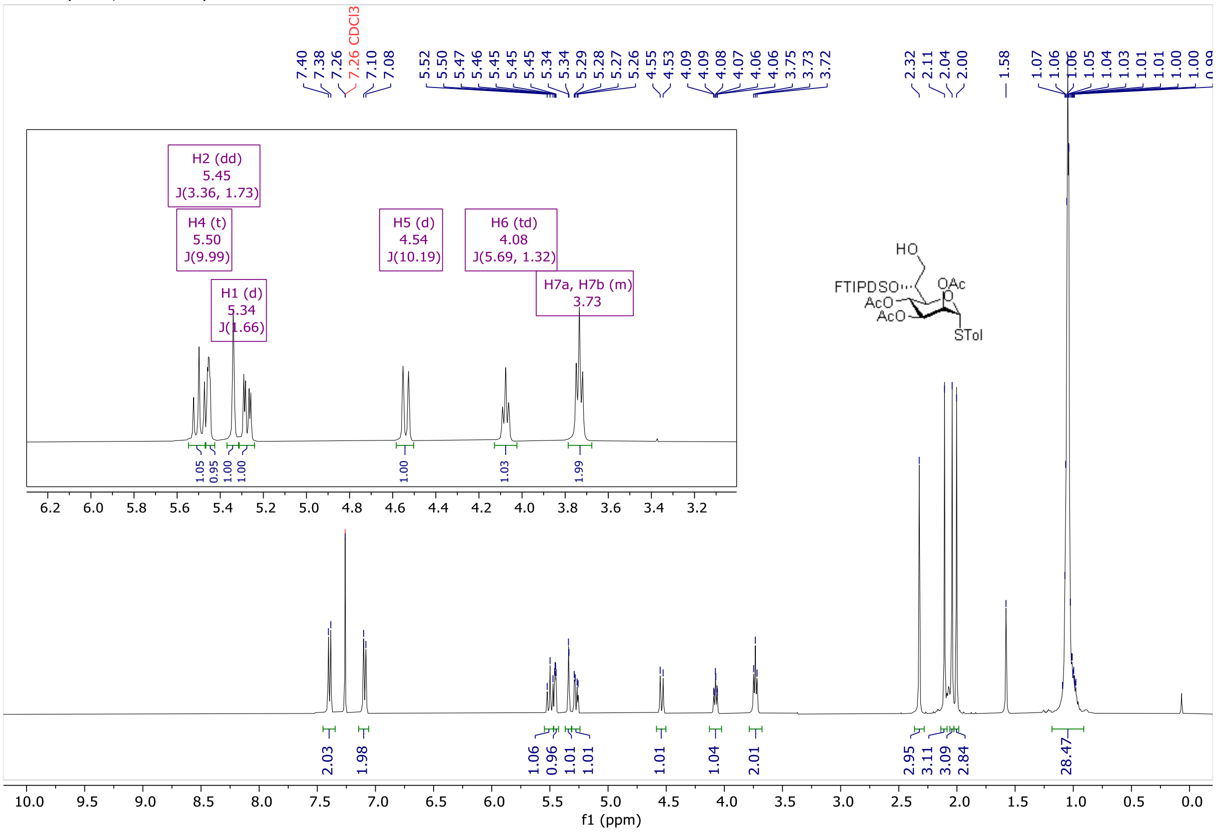

13C NMR (CDCl3, 101 MHz)

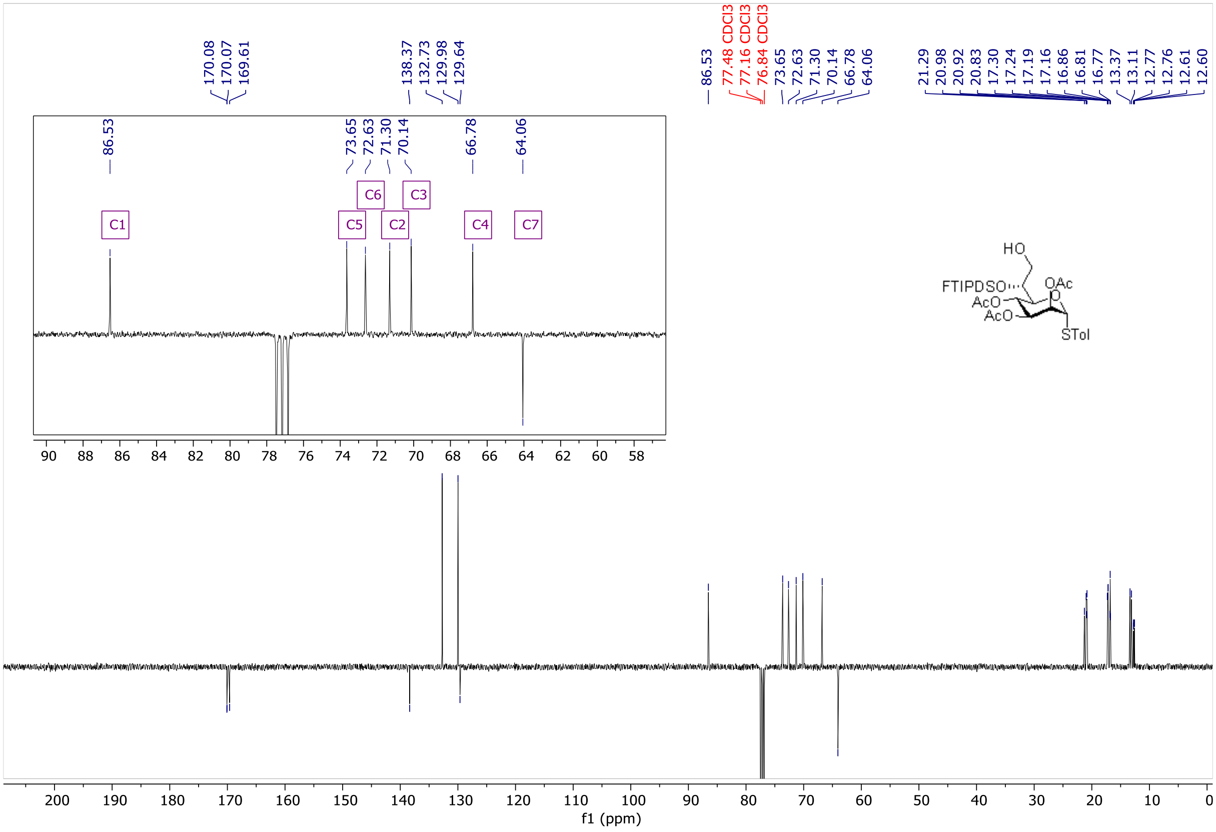

C.3.11. Tol-4-yl 2,3-di-*O*-benzoyl-4-*O*-chloroacetyl-6-*O*-(3-fluoro-1,1,3,3-tetraisopropyl-1,3-disiloxane-1-yl)- 1-thio-D-*glycero*-α-D-*manno*-heptopyranoside (33)

1H NMR (CDCl3, 400 MHz)

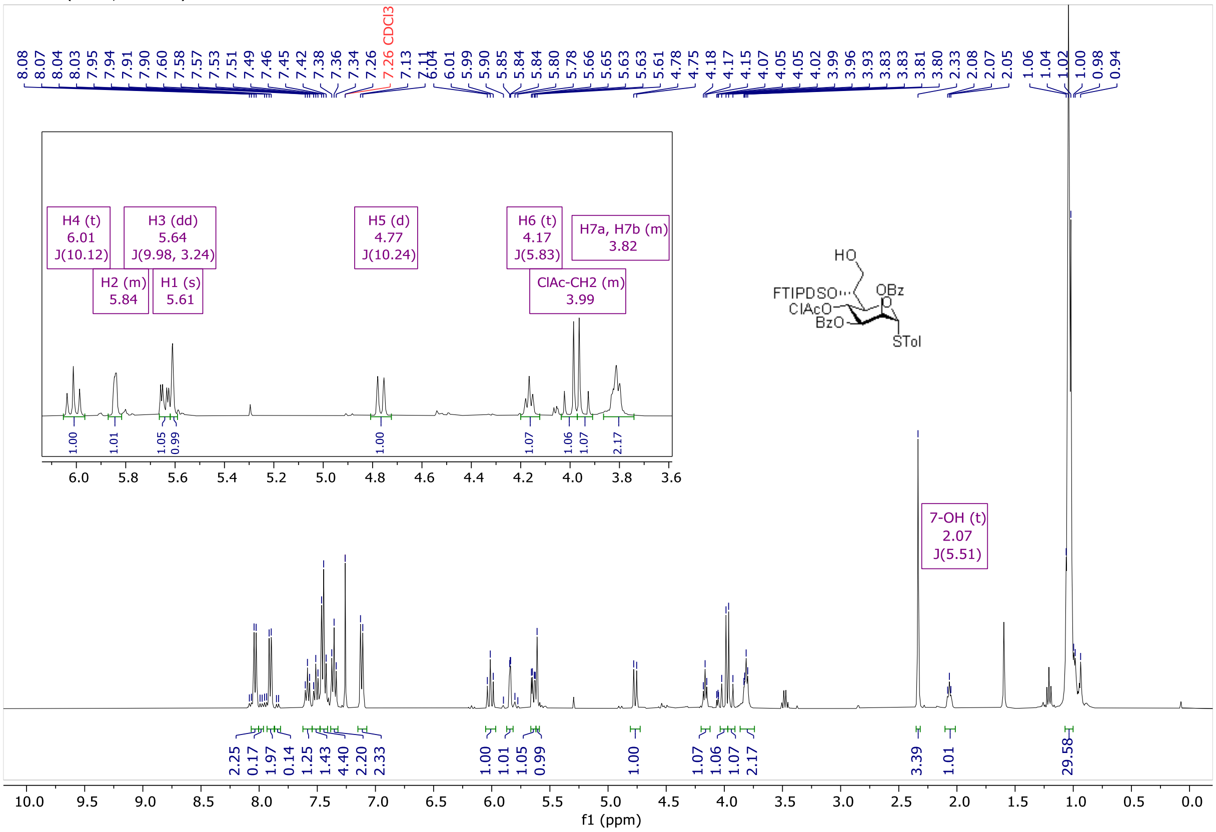

13C NMR (CDCl3, 101 MHz)

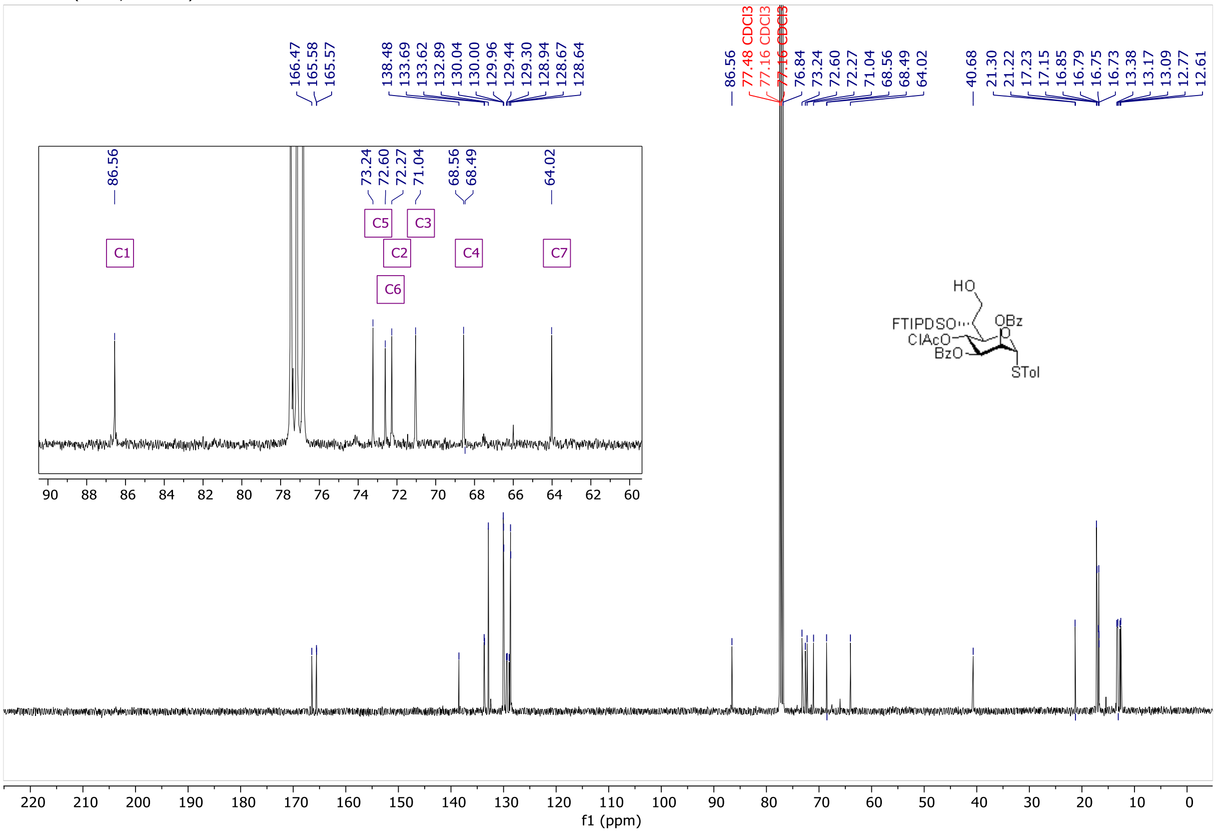

Supplement: Supplementary file 1 [file Data_Sheet_1.PDF]
